# Dual-modal piezotronic transistor for highly sensitive vertical force sensing and lateral strain sensing

**DOI:** 10.1038/s41467-023-41983-3

**Published:** 2023-10-09

**Authors:** Rui Ge, Qiuhong Yu, Feng Zhou, Shuhai Liu, Yong Qin

**Affiliations:** 1 School of Advanced Materials and Nanotechnology, Xidian University, Xi’an, Shaanxi 710071 China; 2 Institute of Nanoscience and Nanotechnology, School of Materials and Energy, Lanzhou University, Lanzhou, Gansu 730000 China; 3 Henan Key Laboratory of Photoelectric Energy Storage Materials and Applications, School of Physics and Engineering, Henan University of Science and Technology, Luoyang, Henan 471000 China; 4 State Key Laboratory of Solid Lubrication, Lanzhou Institute of Chemical Physics, Chinese Academy of Sciences, Lanzhou, 730000 China

**Keywords:** Sensors, Nanowires

## Abstract

Mechanical sensors are mainly divided into two types (vertical force sensing and lateral strain sensing). Up to now, one sensor with two working modes is still a challenge. Here, we demonstrate a structural design concept combing a piezoelectric nano/microwire with a flexible polymer with protrusions that enables a dual-modal piezotronic transistor (DPT) with two working modes for highly sensitive vertical force sensing and lateral strain sensing. For vertical force sensing, DPT exhibits a force sensitivity up to 221.5 N^−1^ and a minimum identifiable force down to 21 mN, corresponding to a pressure sensitivity of 1.759 eV/MPa. For lateral strain sensing, DPT can respond to a large compression strain (~5.8%) with an on/off ratio up to 386.57 and a gauge factor up to 8988.6. It is a universal design that can integrate vertical force sensing and lateral strain sensing into only one nanodevice, providing a feasible strategy for multimodal devices.

## Abstract (web summary)

Developing mechanical sensors with two working modes for detecting vertical force and lateral strain is challenging. Here, Ge et al. report a piezotronic transistor with protrusions that enable dual-modal functionality and improve sensing performance.

## Introduction

Force/strain sensors with capacity of converting mechanical signals into electrical signals have played a significant role in the existing industry and many emerging fields, including electronic skin, human-machine communication, health monitoring, and Internet of Things^1–7^. According to different working principles, force/strain sensors can be mainly classified into piezoresistive^6^, capacitive^5,8^, piezoelectric^3,9–11^, and piezotronic^4^ types, etc. Among them, the piezotronic sensors employ a brand-new regulation mechanism (piezotronic effect^1^), that is, the use of the force/strain-induced piezoelectric polarization charges and corresponding potentials produced at the interfaces so as to linearly regulate the interface barrier height (Supplementary Note 1, Supplementary Fig. 1) and exponentially tune the interface carrier transport^1^. Since its natural exponential control of output signal by input signal, the piezotronic force/strain sensor shows inherent high sensitivity in sensing mechanical stimuli^1,12^. Up to now, its applications have covered many innovative electronics, such as tactile imaging^4^, high-sensitive strain sensor^13^, light nano-antenna^14^, pressure optoelectronics^15^, optofluidic logic computation^16^ and so on.

**Fig. 1:**
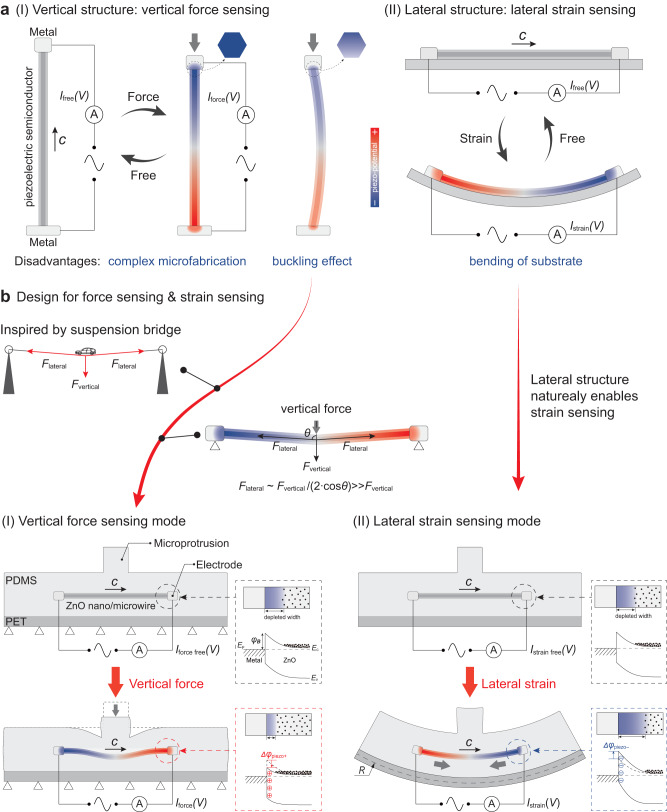
Device design and working mechanism of dual-modal piezotronic transistor (DPT). **a** Schematic diagrams of two traditional structures of piezotronic transistors illustrating the mechanism of (I) the vertical structure for vertical force sensing and (II) the lateral structure for lateral strain sensing. Buckling effect occurred in vertical structure decreases the piezoelectric potential and leads to an uneven potential distribution at terminals, which has negative impact on piezotronic effect. **b** Design of the DPT with two working modes for (I) vertical force sensing and (II) lateral strain sensing. The mode for vertical force sensing is inspired by suspension bridge, in which the vertical force is transformed and amplified to a lateral force that elongates the nano/microwire and avoids the problem of buckling effect in traditional vertical structure. This makes DPT have a lateral structure but can sense vertical force with a high sensitivity. The corresponding energy band diagrams for the piezotronic modulation of Ag/*n*-ZnO Schottky junction at the contact interfaces are shown in the boxes with dotted lines. ${E}_{{{{{{\rm{C}}}}}}}$ and ${E}_{{{{{{\rm{V}}}}}}}$ are respectively the conduction and valence bands of the ZnO; the dotted line is the Fermi level (${E}_{{{{{{\rm{F}}}}}}}$) of the electrodes; $\Delta {\varphi }_{{{{{{\rm{piezo}}}}}}+}$ and $\Delta {\varphi }_{{{{{{\rm{piezo}}}}}}-}$ indicate the change in Schottky barrier height induced by positive and negative piezoelectric polarization charges, respectively.

The core functional unit in a piezotronic sensor is a piezotronic transistor, which is a force/strain-sensitive unit^17^. Analogous to transistors controlled by electrical ‘gate’ signals, piezotronic transistors are usually composed of Schottky junctions formed between piezoelectric semiconductors and metals, which can be controlled by external mechanical signals. In recent years, important progress of piezotronic transistors have been made particularly in exploring effective strategies to improve performance, for example, introducing tunneling mechanism^13,18^, alloy structure^19^, internal holes^20^, and new materials with high piezoelectric coefficients^21^ or special geometries^22^. Nevertheless, the basic structures of piezotronic transistors still remain the same with the early piezotronic devices, which can be divided into two types: vertically driven structure^4,22^ and laterally driven structure^23,24^. The vertical structure is a three-dimensional structure^4^ including a single-crystal piezoelectric semiconductor sandwiched by two electrodes, which not only requires complex microfabrication but also may introduce a buckling effect (right, Fig. 1a-I, leading to decreased and uneven piezoelectric potential at two terminals and detrimental to piezotronic effect)^22^. This structure is only utilized to directly detect vertical force or pressure without bending substrates, whose typical representatives are tactile imaging^4^ and pressure optoelectronics^15^. The lateral structure is fairly two-dimensional and can be easily fabricated but only applied to detect lateral strain (Fig. 1a–II)^13,25^, which is adopted in the recently proposed ultra-sensitive strain sensors based on piezotronic tunneling junction^13^ and the strain-controlled power devices^26^. These two structures can detect vertical force or lateral strain separately, while not meeting the demand of actual applications that require the same sensor to switch between two working modes. How to make above two structures compatible and integrate their merits is of great significance to the development in the new principles and potential applications of piezotronic sensors. Therefore, it is highly desired to find a new scheme to realize piezotronic transistor possessing two modes for vertical force sensing and lateral strain sensing.

In this work, we reported a dual-modal piezotronic transistor (DPT) combining a piezoelectric nano/microwire with a flexible polymer with protrusions that enables one device with two working modes for both highly sensitive vertical force sensing and lateral strain sensing. For vertical force sensing mode, DPT possesses a high force sensitivity of 221.55 N^−1^ and a minimum identifiable force of 21 mN. Its corresponding pressure sensitivity is about 1.759 eV/MPa, more than 16.5 times of previously reported piezotronic transistors^4,21,27–33^. Meanwhile, for lateral strain sensing mode, DPT exhibits high performance with a high gauge factor of 8988.6 and an on/off ratio of 386.57 while undergoing a response of up to 5.8% compressive strain. This proposed piezotronic transistor with two sensing modes is a step taken in both functional expansion and performance improvement (Supplementary Note 2, Supplementary Figs. 2–6), which will push forward the design of functional piezotronic electronics and provide a feasible strategy for multimodal devices.

## Results

### Structure design and working mechanism of DPT

The detailed fabrication process of DPT is described in the **Methods** (Supplementary Note 3, Supplementary Figs. 7–8). As shown in Fig. 1b–I, the DPT has a lateral structure with an individual *n*-ZnO nano/microwire clamped by Ag electrodes at two terminals, which is entirely encapsulated in the polydimethylsiloxane (PDMS) on a flexible PET substrate. The ZnO nano/microwire was synthesized by chemical vapor deposition, possessing a hexagonal geometry with a good wurtzite crystallinity (Supplementary Note 4, Supplementary Figs. 9–10). As the key piezoelectric semiconductor in DPT, the synthesized ZnO nano/microwire exhibits a good piezoelectricity (${d}_{33}$ ~ 12 pm/V) with polarization *c*-axis along its length (Supplementary Note 5-6, Supplementary Figs. 11–12 and Supplementary Table 1).

The DPT possesses a structural design inspired by suspension bridge (Fig. 1b, Supplementary Fig. 5), in which the vertical force is transformed and amplified to a lateral force that can greatly elongate the nano/microwire and avoid the problem of buckling effect in traditional vertical structure. Rather than the compression of the vertical nano/microwire in traditional vertical structure, it is the elongation of the lateral nano/microwire that is utilized in vertical force sensing mode of DPT. This makes DPT have a lateral structure but can sense vertical force with structural advantages of high sensitivity in principle (Supplementary Note 2). The soft nature of PDMS avoids local extensive stress. In addition, to realize a sensitive detection of a vertical force, a microprotrusion made of PDMS is introduced just above the ZnO nano/microwire (upper, Fig. 1b–I). It can effectively convert a compression deformation induced by the vertical force into a downward bending of ZnO nano/microwire (lower, Fig. 1b–I). Since both ends of the nano/microwire are clamped, a tensile strain along the piezoelectric *c*-axis of the nano/microwire can be induced by the downward bending. As illustrated in Fig. 1b–I, if the polarization *c*-axis of nano/microwire points to the right, negative and positive piezoelectric polarization charges and corresponding potentials will be respectively generated at the left and right ends of the ZnO nano/microwire. According to the theory of piezotronic effect^34^, (taking the contact of the right end of the nano/microwire as an example) the positive piezoelectric polarization charges at the right end of the nano/microwire induced by the applied force will reduce the height of the Schottky barrier (red dotted box in Fig. 1b–I) and hence enhance the carrier transport of Ag/*n*-ZnO interface. Benefiting from the advanced structural design (including suspension-bridge-inspired structure and introduction of microprotrusion) and avoiding the problem of buckling effect, the DPT in the ‘vertical force sensing mode’ can realize vertical force sensing without bending the substrate and have the potential to detect vertical force with high sensitivity (Supplementary Note 2, Supplementary Figs. 2–6).

In addition, DPT also has another working mode for strain sensing by bending the substrate through controlling the radius of curvature $R$ of the substrate (Supplementary Note 7, Supplementary Fig. 13). As shown in Fig. 1b–II, when the substrate is bent downward, the ZnO nano/microwire will be subjected to a compressively strain. Positive and negative piezoelectric polarization charges will be respectively generated at the left and right ends of the ZnO nano/microwire, which can modulate the interface barrier height (blue dotted box in Fig. 1b–II) and the electrical transport of DPT. In this ‘lateral strain sensing mode’, the DPT can directly sense lateral strain through the bending of the flexible substrate, which is similar to the traditional piezotronic transistor for strain sensing^24^ (Fig. 1a–II). So, in terms of functional expansion, DPT intrinsically possesses two working modes that enable vertical force sensing and lateral strain sensing, respectively; in terms of performance improvement, the design of DPT has potential to sense vertical force with high sensitivity in theory.

### Structural optimization of DPT

**Fig. 2:**
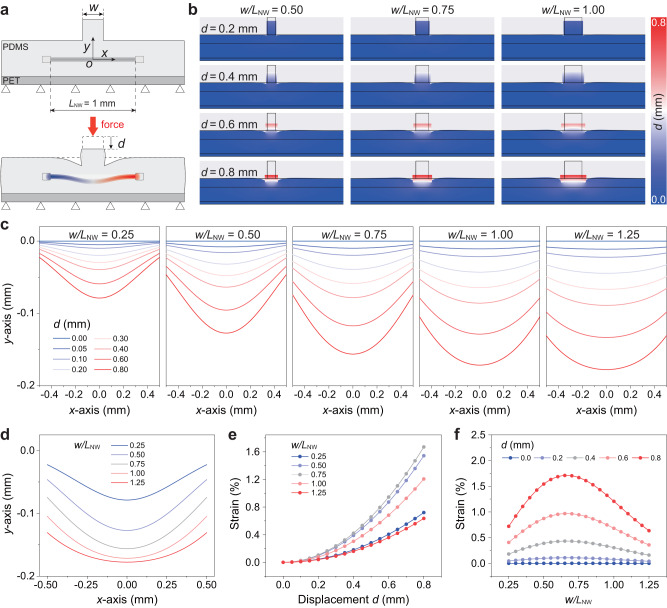
Structural optimization of dual-modal piezotronic transistor (DPT) by the theoretical simulation. **a** Schematic diagram of the finite element method (FEM) model of DPT without (top) and with (bottom) a vertical force. ${L}_{{{{{{\rm{NW}}}}}}}$, $w$ and $d$ represent the length of the ZnO nano/microwire, the width of the microprotrusion and the downward displacement of the microprotrusion’s upper surface, respectively. **b**, **c** Displacement distribution profiles of DPT (**b**) and the corresponding outlines of bended nano/microwires (**c**) with varies of structure ratios $w/{L}_{{{{{{\rm{NW}}}}}}}$ in response to different displacements $d$ obtained from the FEM simulation. **d** Outlines of nano/microwires in the DPT with various structure ratios $w/{L}_{{{{{{\rm{NW}}}}}}}$ under constant displacement ($d$ = 0.8 mm). **e** The calculated strain of nano/microwire with various structure ratios $w/{L}_{{{{{{\rm{NW}}}}}}}$ as a function of displacement $d$. **f** The curves of nano/microwire strain versus structure ratio $w/{L}_{{{{{{\rm{NW}}}}}}}$ under different displacements $d$ derived from (**e**).

In order to optimize the DPT structure to improve its sensitivity to a vertical stimulus, the strain of ZnO nano/microwire induced by various sizes of microprotrusions has been investigated through the method of finite element simulation in Fig. 2 and Supplementary Note 8. The detailed model used in the simulation is illustrated in Fig. 2a (Supplementary Fig. 14a–c, Supplementary Table 2), in which ${L}_{{{{{{\rm{NW}}}}}}}$, $w$ and $d$ represent the length of ZnO nano/microwire, the width and the downward displacement of the upper surface of microprotrusion, respectively. The displacement distribution profiles in Fig. 2b and Supplementary Fig. 14d exhibit the shape change of the DPTs with various microprotrusion structure ratios $w/{L}_{{{{{{\rm{NW}}}}}}}$ under different displacements $d$. The corresponding outlines of bending nano/microwires in different cases in Fig. 2b are plotted in Fig. 2c. It can be found that the downward displacement tends to bend the nano/microwire as the ratio $w/{L}_{{{{{{\rm{NW}}}}}}}$ decreases, while makes the nano/microwire move down as a whole with the increase of the ratio. This can be also revealed in Fig. 2d, illustrating the nano/microwire outlines in the DPTs with various microprotrusion widths $w$ under the same displacement ($d$ = 0.8 mm).

Furthermore, by simulating the change of the ZnO nano/microwire length from Fig. 2c, the strain of ZnO nano/microwire can be approximately evaluated (Supplementary Note 7 and Supplementary Note 9). Figure 2e summarizes the curves of nano/microwire strain versus displacement $d$, which indicates that the nano/microwire strain increases monotonously with the increase of the displacement, and increases fastest when the ratio $w/{L}_{{{{{{\rm{NW}}}}}}}$ is around 0.75. To make the DPT possess higher sensitivity, the optimal structure of the microprotrusion is required to be investigated, which is able to produce greater strain of the nano/microwire and more piezoelectric polarization charges in response to external vertical stimuli. The plots of ZnO nano/microwire strain as a function of the structure ratio $w/{L}_{{{{{{\rm{NW}}}}}}}$ are shown in Fig. 2f. It can be found that the DPT will have the maximum strain response when the microprotrusion structure ratio $w/{L}_{{{{{{\rm{NW}}}}}}}$ is about 0.665. It should be noted that the possible unbalanced deformation (buckling) of the microprotrusion under compression is not considered in the above simulations. Actually, the stability of the microprotrusion with the structure ratio $w/{L}_{{{{{{\rm{NW}}}}}}}$ of about 0.66 exhibits good in experiments, and no obvious buckling occurs. Additional simulations for DPT structural optimization are also carried out in Supplementary Note 8, Supplementary Figs. 15–26 and Supplementary Table 3.

### DPT in vertical force sensing mode

**Fig. 3:**
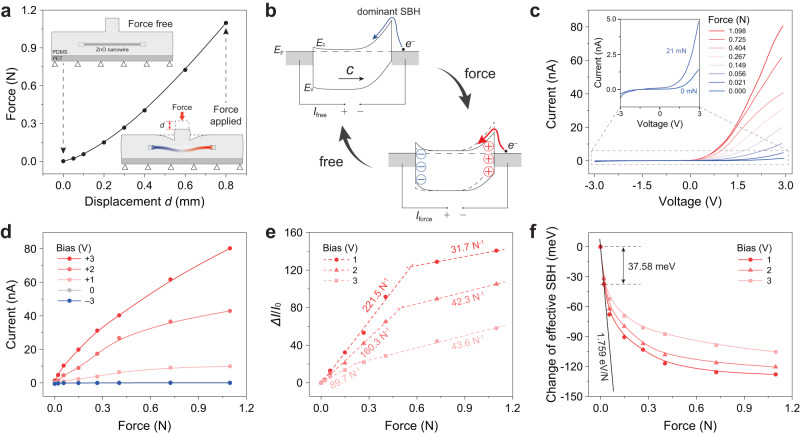
Piezotronic modulation of electrical characterization in the dual-modal piezotronic transistor (DPT) for vertical force sensing. **a** The measured calibration curve of the relationship between the vertical force and downward displacement $d$. Inset: schematic diagrams of the DPT for vertical force sensing without and with the applied force. **b** Energy band diagrams of the metal-semiconductor-metal (MSM) structure with a dominant Schottky barrier (SB) showing the piezotronic behavior in the DPT without (left) and with (right) the vertical force under reverse bias. **c** Force-dependent *I*-*V* characteristics of DPT under the sweeping bias between −3 V and +3 V. Inset: the enlarged *I*-*V* curves with applied forces of 0 and 21 mN. As the force increases, the forward current increases drastically. **d** Current as a function of the applied force under bias of −3 V, 0 V, 1 V, 2 V and 3 V. **e** Curves of current change ratio $(\Delta I/{I}_{0})$ versus force under various biases. The corresponding sensitivities at two force regions are given by the slopes of curves in the plots. **f** Change of effective Schottky barrier height (SBH) derived from (**c**) as a function of applied force. The highest-pressure sensitivity, 1.759 eV/MPa was obtained under a small force (<~21 mN) at the bias of 1 V.

According to the aforementioned simulations, the DPT with optimal structure was fabricated (Supplementary Note 10, Supplementary Fig. 27) to study its performance for vertical force sensing. In order to apply a precise vertical force and explore the approximate force range of DPT, we accurately control the force on the DPT by controlling the downward displacement *d* of the upper surface of the microprotrusion. The specific displacement is applied by a high-precision actuator, and the force is measured by a force sensor placed under the DPT. Experimental setup is detailed in Supplementary Note 11 and Supplementary Fig. 28. The applied force as a function of displacement is firstly calibrated and shown in Fig. 3a. It can be found that a nonlinear relationship between the force and the displacement conforms the mechanical characteristics of the DPT.

Prior to the force-induced piezotronic modification of *I*-*V* characteristics, the corresponding energy band diagrams of DPT with force free and force applied are illustrated in Fig. 3b. When the DPT is free, the electrical transport of the device will be mainly regulated by the reverse-biased Schottky barrier (SB) or the dominant SB that possesses a higher barrier height (upper, Fig. 3b). In this case, electrons require a high energy to leap over the dominant SB, resulting in a small current. As the DPT is subjected to a vertical force, the microprotrusion will convert the force-induced compression deformation into a downward bending of the ZnO nano/microwire, thereby generating piezoelectric polarization charges and corresponding piezoelectric potentials at both ends of the nano/microwire. According to the piezotronic theory^34^, for the Schottky barrier formed by *n*-type semiconductors and metals, the positive and negative piezoelectric polarization charges will respectively reduce and increase the energy band profile. As the case illustrated in Fig. 3b, the positive piezoelectric polarization charges produced at interface will reduce the dominant Schottky barrier height, which in turn enables electrons to only require lower energy to cross the barrier, and thus forms a big current. By observing the change in current, the vertical force applied on the DPT can be sensed.

Supplementary Fig. 28 shows an electrical measurement system used to exact a sweeping voltage to the DPT and simultaneously measure its electrical transport. Based on this measurement system, we investigated the vertical force-dependent *I*-*V* curves of DPT in Fig. 3c. When no force is applied, the forward current is larger than the reverse current (inset of Fig. 3c), revealing that the Schottky barriers at two ends of ZnO nano/microwire are different. As the force increases, the forward current increases dramatically, while the reverse current remains at a small magnitude, indicating that the Schottky barrier on the right side of the energy band in Fig. 3b plays a dominant role. It should be noted that the above judgment is based on the right-pointed *c*-axis direction of the ZnO nano/microwire, which can be confirmed by the method of nanogenerator test (Supplementary Note 6 and Supplementary Fig. 12). To further analyze the piezotronic modification of the carrier transport, the currents under bias of −3 V, 0 V, 1 V, 2 V and 3 V as a function of the force are plotted in Fig. 3d. It can be seen that the magnitude of the current changing with the applied force is gradually enhanced as the bias increases; whereas the increasing trend of current becomes slower with the increased force.

To deeply investigate the regulation mechanism of the vertical force on the electrical transport, curves of $\Delta I/{I}_{0}=\frac{\left({I}_{{{{{{\rm{force}}}}}}}-{I}_{0}\right)}{{I}_{0}}$ versus force at different bias are shown in Fig. 3e, in which ${I}_{{{{{{\rm{force}}}}}}}$ and ${I}_{0}$ represent the current with force applied and force free, respectively. We find that $\Delta I/{I}_{0}$ increases approximately linear with the force, but each curve is divided into two sections with different slopes. In detail, the slope of $\Delta I/{I}_{0}$ versus the force is big under a small-force range (e.g. < 0.6 N under bias of 1 V) while the slope is small as the force is relatively large (e.g. >0.6 N under bias of 1 V). The main reason for this phenomenon is that when the force is small, the Schottky barrier at the right end in Fig. 3b plays a dominant role, and the reduction of its barrier height makes the current increase rapidly; however, as the force gradually increases to a critical value (e.g. 0.6 N under bias of 1 V), the Schottky barrier at the left end (Fig. 3b) increases, while the dominant Schottky barrier at the right end (Fig. 3b) continues to decrease; these two effects offset each other, which makes the current growth begin to slow down. This gradually slowing trend is consistent with the phenomenon observed in Fig. 3d. Actually, there involve three regulations induced by piezoelectric polarization and bias voltage, and the whole regulation process here is very complicated, which is in-depth discussed in Supplementary Note 12 and Supplementary Figs. 30−32. It is noteworthy that the DPT can achieve a high sensitivity of 221.5 N^−1^ in the measurement range from 0 N to 0.6 N under bias of 1 V. In other words, the gravity of an object weighing about 4 g can change the DPT current by 10 times. In addition, using the method in Supplementary Note 13, we also calculated the change of effective Schottky barrier height (SBH) as a function of the force in Fig. 3f. A remarkably change of effective SBH (~37.58 meV) can be observed as the applied vertical force increases from 0.00 N to about 0.02 N, showing that a small force is enough to cause a substantially decreased effective SBH. Considering the area of the force, the estimated pressure sensitivity is 1.759 eV/MPa of the DPT for force sensing, which is a key parameter in piezotronics and is 16.5 times more than that of piezotronic transistors based on traditional vertical structure^4,27^ (Supplementary Table 4). This high performance in vertical force sensing can be attribute to the advanced structural design (containing suspension-bridge-inspired structure and introduction of microprotrusion) of amplifying the vertical force to the tensile force and the perfect solution to the problem of buckling effect in traditional vertical structure (Supplementary Note 2). Meanwhile, although the effective SBH change will continue to decrease as the force further increases from 0.02 N to 1.00 N, its magnitude will be lower than that of the range from 0.00 N to about 0.02 N, which indicates that the rapidly increasing current and the high sensitivity of DPT mainly occurs under a small vertical force. This weakened modulation occurred in the large force range can be attributed to the obviously offsetting effect of piezotronic modification of two Schottky barriers as the applied force is large enough, as detailly discussed in Supplementary Note 14 and Supplementary Fig. 33.

### DPT in lateral strain sensing mode

**Fig. 4:**
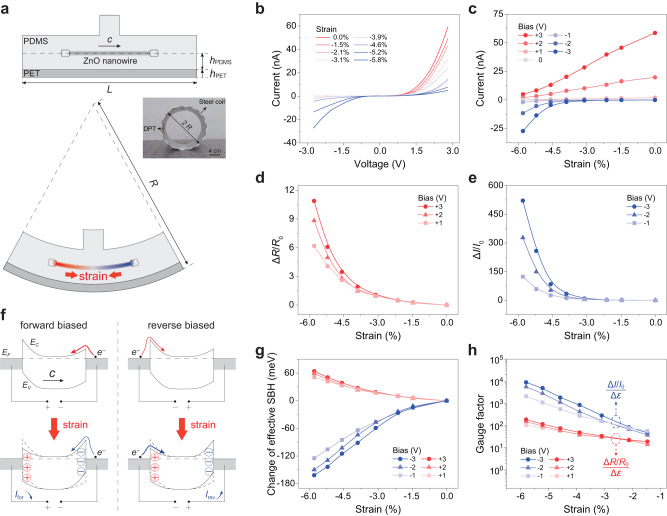
Piezotronic modulation of electrical characterization in the dual-modal piezotronic transistor (DPT) for lateral strain sensing. **a** Schematic diagrams of the DPT showing the driving mode for lateral strain sensing to evaluate the axial strain ($\varepsilon$) of the nano/microwire. The thicknesses of the lower layer PDMS and the PET substrate are ${h}_{{{{{{\rm{PDMS}}}}}}}$ and ${h}_{{{{{{\rm{PET}}}}}}}$; the length and the bending radius of the PET substrate are $L$ and $R$, respectively. Inset: Photo image of controlling the strain applied on DPT by controlling the radius of the steel coil. **b** Strain-dependent *I*-*V* characteristics of DPT under the sweeping bias between −3 V and +3 V. With the increase of compressive strain, the reverse current increases rapidly, while the forward current decreases, which exhibits the asymmetric modulation of electrical transport by the piezotronic effect. **c** Current as a function of applied strain under bias of −3, −2, −1, 0, 1, 2 and 3 V. **d**, **e** Resistance change ratio ($\Delta R/{R}_{0}$) (**d**) and current change ratio ($\Delta I/{I}_{0}$) (**e**) versus strain under various forward and reverse biases, respectively. **f** Energy band diagrams showing the strain-induced piezotronic behavior in the DPT under opposite bias. ${I}_{{{{{{\rm{for}}}}}}}$ and ${I}_{{{{{{\rm{rev}}}}}}}$ represent forward and reverse current, respectively. **g** Change of effective Schottky barrier height (SBH) as a function of strain as forwardly and reversely biased derived from the *I*-*V* curves in (**b**). **h** Gauge factor (GF) as a function of strain to evaluate the strain-sensing performance of DPT. The highest GF is about 8988.6.

Next, we investigated the performance of DPT as a strain sensor along the lateral direction. Figure 4a shows the structure of DPT and the corresponding driving mode for lateral strain sensing (Supplementary Note 7), which is similar to the traditional lateral structure of piezotronic transistor^24^ (Fig. 1a–II). It utilizes the bending of the substrate (a certain curvature radius *R*) to compress the ZnO nano/microwire and introduce piezoelectric polarization charges at two ends. The applied strain on DPT is controlled by changing the radius of the steel coil (inset of Fig. 4a).

Figure 4b shows the lateral strain-dependent *I*-*V* characteristics of DPT. Owing to the existence of the lower layer PDMS with a thickness of ${h}_{{{{{{\rm{PDMS}}}}}}}$, the strain induced in the nano/microwire by bending substrate is considerably large compared with the traditional structure (Fig. 1a–II). It can be clearly observed that both the forward and reverse current change remarkably with the applied strain. With the increase of compressive strain, the forward current decreases, while the reverse current increases. This asymmetric strain-controlled *I*-*V* characteristic indicates that the piezotronic effect is the dominant modulation principle of DPT (Supplementary Note 15, Supplementary Figs. 34−36), rather than some other effects like piezoresistive effect. In addition, the current variations of DPT with the lateral strain under different biases are also plotted in Fig. 4c. It is obvious that the DPT current tends to change toward a negative current as the compressive strain increases. Through the analysis of Fig. 4c and the consideration of DPT’s asymmetric modulation (Supplementary Fig. 35), we can obtain the resistance response of DPT as forwardly biased (corresponding to the positive voltage, Fig. 4d), and the current response of DPT as reversely biased (corresponding to the negative voltage, Fig. 4e). Figure 4f illustrates the corresponding energy band profiles of DPT under opposite biases with strain free and strain applied. Normally, the reverse-biased Schottky barrier will play a dominant role, just as shown in Fig. 4f. For example, when the DPT is forwardly biased (left, Fig. 4f), the reverse-biased Schottky barrier is the right one, which plays a dominant role in the carrier transport of DPT. Its barrier height will increase as the strain-induced negative piezoelectric polarization charges are produced, leading to a decreased current. The similar analysis is also adaptable to the case when the DPT is reversely biased (right, Fig. 4f). It is worth noting that, although the reverse-biased Schottky barrier plays a dominant role in the absence of strain, the large lateral strain induced in the DPT (owing to ${h}_{{{{{{\rm{PDMS}}}}}}}$) causes significant change in both Schottky barrier heights, that one increases and the other decreases, eventually leading to an offsetting effect on the carrier transport of DPT. To evaluate this offsetting effect, we calculated the change of effective Schottky barrier height (SBH) as a function of the strain (Fig. 4g), indicating the decreased effective SBH under reverse bias and the increased effective SBH under forward bias. It can be found that the opposite regulation of the piezotronic effect on the interface barriers is almost linearly related to the strain, particularly in the high strain range from about −2% to −6%.

In order to evaluate the performance of DPT for lateral strain sensing, we derived the gauge factor, which is defined as the ratio of relative change in current or resistance to the strain (Supplementary Note 16), of DPT under different biases in Fig. 4h. When the lateral strain is about −5.8% and the bias is −3 V, the highest gauge factor of DPT can reach 8988.6, which is a relatively high value in strain sensors (Supplementary Note 17, Supplementary Fig. 37 and Supplementary Table 6).

### Dynamic current response of DPT to external strain and force

**Fig. 5:**
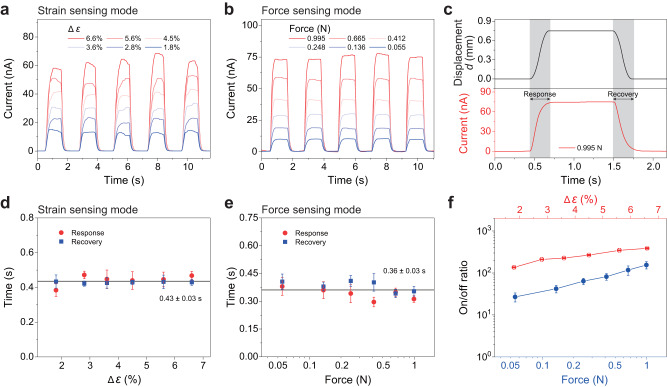
Dynamic current response of dual-modal piezotronic transistor (DPT) to periodic mechanical stimuli (lateral strain and vertical force). **a**, **b** *I*-*t* curves of the current response to 5 on/off loading cycles under different strain changes (**a**) and forces (**b**) at a fixed bias of 3 V for strain sensing and force sensing, respectively. **c** Detailed current response-recovery curve exhibiting the real-time current change with the displacement-controlled force of 0.995 N in (**b**). The corresponding response time and recovery time are shown by the gray regions. **d**, **e** Statistical response time and recovery time of DPT in the lateral strain sensing mode (**d**) and the vertical force sensing mode (**e**) derived from (**a**) and (**b**), respectively. The error bars denote standard deviations of the average value. **f** On/off ratio of the DPT in its two working modes for lateral strain sensing and vertical force sensing derived from the current statistics in (**a**), (**b**). The error bars in (**d**–**f**) denote standard deviations of the mean.

We also measured the dynamic response characteristics of DPT to periodic lateral strain and vertical force in two working modes in Fig. 5. The periodic strain and force were imposed on the DPT by the high-precision actuator, and meanwhile the dynamic response was measured by the electrical measurement system. In Fig. 5a,b, different series of *I*-*t* curves exhibit the current response to the stimuli of 5 on/off cycles with different magnitudes of strain and force, respectively. The currents both respond very strongly as soon as stimulated by strain or force, and immediately returned to the initial state, demonstrating the excellent temporal response performance of DPT in sensing strain or force. As can be seen, while the amplitude of the current response monotonically increases with the strain or force, the current signals remain nearly identical under the same stimulus and no obvious signal attenuation are observed throughout the test, indicating the repeatable and stable response characteristics of DPT. According to the detailed current response process of DPT plotted in Fig. 5c, we can evaluate the response time and recovery time. Figure 5d,e gives the response time and recovery time of DPT for lateral strain sensing and vertical force sensing, respectively. In the lateral strain sensing mode, the response time of DPT slightly increases with the increased strain and the recovery time remains essentially unchanged, which have an average value of about 0.43 s. And in the vertical force sensing mode, the response and recovery time of DPT are both about 0.36 s. These values of time are dominated by the response time of the measurement apparatus and the loading speed of the strain or force, where the output current keeps well in step with the external stimulus, as shown in Fig. 5c. The fast response (without hysteresis) demonstrates that the sensor can switch rapidly at loading and unloading, which ensures the ability of real-time tracking of the applied strain or force. In addition, the on/off ratio (defined in Supplementary Note 18) of the DPT in its two working modes have also been calculated and summarized in Fig. 5f. It can be found that the on/off ratio can be enhanced by increasing the stimulating signal. The maximum switching on/off ratio of DPT can reach 154.86 to vertical force and 386.57 to lateral strain. In conclusion, the DPT can work in two modes and exhibits reliable and rapid response as a strain sensor or force sensor. It should be noted that DPT cannot decouple the two sensing signals, which is the problem to be solved in the future research (Supplementary Note 19, Supplementary Fig. 38).

## Discussion

In summary, a dual-modal piezotronic transistor with a structure design is proposed and developed to achieve a sensitive sensor with two modes for vertical force sensing and lateral strain sensing. For force sensing in the vertical direction, DPT exhibits a high sensitivity of 221.5 N^-1^ and a high pressure sensitivity of 1.759 eV/MPa, particularly in detecting a small force. As a macroscopic device, an object weighing only 4 g can make the DPT current change 10 times. Furthermore, for strain sensing along the lateral direction, DPT shows high sensitivity with on/off ratio of 386.57 and gauge factor of 8988.6 at a strain of about -5.8%. By integrating vertical force sensing and lateral strain sensing into one nanodevice with a lateral structure, DPT provides more possibilities in the piezotronics and some other piezoelectric nanodevices, which will benefit for the application design of advanced piezoelectric materials.

## Methods

### Growth of ZnO nano/microwires

The synthesis of ZnO nano/microwires were implemented in a conventional tube furnace via chemical vapor deposition (CVD) process. The starting materials were ZnO (2.000 g) and graphite (0.400 g) powder, which were uniformly mixed in an agate mortar. The resulting mixture was transferred in an alumina boat and then placed in the center region of the tube furnace. Afterwards, the system was heated to 1150 °C for 40 min and then to 1250 °C for 40 min, under the flow of Ar (240 sccm) and O_2_ (12 sccm). Eventually, the grown ZnO nano/microwires were collected near the outlet of the furnace tube.

### Fabrication of dual-modal piezotronic transistor (DPT)

First, PET films (150 μm in thickness) were ultrasonicated and rinsed with acetone, ethanol and deionized water prior to dry cleaning by nitrogen gas. Then, semidry PDMS mixture (Sylgard 184-Dow Corning, the base and curing agent in the *wt*. ratio of 10:1) was transferred onto the PET substrate, followed by curing at 100 °C for 1 h on a hotplate. The PET film with a PDMS layer (∼825 μm) was accordingly obtained for use as the supporting substrate. After that, a ZnO nano/microwire was placed laterally on the PDMS layer of the flexible substrate under an optical microscope, while the substrate could be cut into the desired dimensions (30 mm × 10 mm) based on the alignment of the ZnO nano/microwire. To constitute a metal-semiconductor-metal structure, the source/drain Ag electrodes were deposited on the ZnO nano/microwire by RF-magnetron sputtering, with both ends of the nano/microwire clamped and connected to copper wires by silver paste to improve the interface property. Subsequently, another PDMS thin layer (∼500 μm) as encapsulation was applied to wrap around the nano/microwire and then baked at 100 °C for 1 h. Finally, a small piece of PDMS with dimension of 1 mm × 1 mm × 1 mm was bonded to the top PDMS layer above the center of the nano/microwire, forming microprotrusion for force transfer. Diagram of fabrication is given in Supplementary Note 3.

### Electrical connection of dual-modal piezotronic transistor (DPT)

A data acquisition card (National Instruments) is used for data acquisition and input the data to the computer. A synthesized function generator (Stanford Model DS 345) is applied to produce a drive voltage on DPT. With the help of shielded junction box, the voltage signal is also imported into the data acquisition card. Meanwhile, a low-noise current preamplifier (Stanford Model SR570) is used to measure the current flow through the DPT and also import the current value into the data acquisition card. In the specific test process for vertical force sensing, a linear actuator is controlled by the computer to apply a displacement on the DPT, and a force sensor under the substrate readouts the force applied on DPT at the same time. The image/illustration of electrical connection and corresponding circuit diagram are provided in Supplementary Note 11 and Supplementary Fig. 28. Note that the potential damping effects from components constituting the DPT (such as PDMS) actually have little effect on the accuracy of the force measurement (Supplementary Note 11, Supplementary Fig. 29).

### Reporting summary

Further information on research design is available in the Nature Portfolio Reporting Summary linked to this article.

### Supplementary information

Supplementary Information

Peer Review File

Reporting Summary

## Data Availability

The data that support the findings of this study are available from the corresponding author upon request.
