# Supplementary material for: Dual-modal piezotronic transistor for highly sensitive vertical force sensing and lateral strain sensing
Source: Nat Commun. 2023 Oct 9;14:6315. doi: 10.1038/s41467-023-41983-3 (PMC10562489; doi:10.1038/s41467-023-41983-3)
Supplement: Supplementary file 1 — Supplementary Information [file 41467_2023_41983_MOESM1_ESM.pdf]

Supplementary Information for
Dual-modal piezotronic transistor for highly sensitive vertical force sensing and lateral strain sensing

Rui Ge[1,2†], Qiuhong Yu[2,3†], Feng Zhou[4], Shuhai Liu[2], Yong Qin[2]**

[1]*School of Advanced Materials and Nanotechnology, Xidian University, Xi'an, Shaanxi 710071, China*
[2]*Institute of Nanoscience and Nanotechnology, School of Materials and Energy, Lanzhou University, Lanzhou, Gansu 730000, China*
[3]*Henan Key Laboratory of Photoelectric Energy Storage Materials and Applications, School of Physics and Engineering, Henan University of Science and Technology, Luoyang, Henan 471000, China*
[4]*State Key Laboratory of Solid Lubrication, Lanzhou Institute of Chemical Physics, Chinese Academy of Sciences, Lanzhou 730000, China*

†These authors contributed equally to this work.
**Corresponding author E-mail: liushuhai1991@live.cn, qinyong@lzu.edu.cn*

This PDF file includes:
Supplementary Notes 1-19:
Supplementary Note 1 | Piezotronic linear regulation on interface barrier height
Supplementary Note 2 | Design idea of DPT for performance improvement and functional expansion (Novelty of DPT)
Supplementary Note 3 | Step-by-step fabrication of DPT
Supplementary Note 4 | Material characterization
Supplementary Note 5 | Piezoelectricity in wurtzite ZnO
Supplementary Note 6 | Piezoelectric nanogenerator measurement to determine the polarity of the ZnO nano/microwire
Supplementary Note 7 | Driving mode and calculation method of strain on DPT
Supplementary Note 8 | Simulations for the structural optimization of the DPT
Supplementary Note 9 | Calculation of the strain in simulations
Supplementary Note 10 | Method to realize optimal microprotrusion structure ratio of 0.66
Supplementary Note 11 | Experimental setup and electrical measurement
Supplementary Note 12 | Three regulations induced by piezoelectric polarization and bias voltage
Supplementary Note 13 | Calculation of the change of effective Schottky barrier height
Supplementary Note 14 | Weakened piezotronic modulation in the DPT for vertical force sensing
Supplementary Note 15 | Definition of the change ratio in piezotronic transistors

Supplementary Note 16 | Definition of the gauge factor in piezotronic transistors
Supplementary Note 17 | Comparison of DPT with some other sensors
Supplementary Note 18 | Definition of the on/off ratio
Supplementary Note 19 | Crosstalk between two sensing signals in DPT and its possible solution

Supplementary Figures 1-38:

Supplementary Fig. 1 | Ideal metal-semiconductor Schottky contact with the presence of piezoelectric charges at an applied voltage $V = 0$ (thermal equilibrium).
Supplementary Fig. 2 | A long-standing challenge in piezotronics.
Supplementary Fig. 3 | Problem of buckling effect in vertical structure of piezotronic transistors.
Supplementary Fig. 4 | Structural evolution of piezotronic transistors to solve the problem of buckling effect.
Supplementary Fig. 5 | Our original design idea of dual-modal piezotronic transistor (DPT).
Supplementary Fig. 6 | Novelty of the dual-modal piezotronic transistor (DPT).
Supplementary Fig. 7 | Schematic diagram showing the fabrication process of the DPT.
Supplementary Fig. 8 | Schematic illustration of the ZnO nano/microwire alignment.
Supplementary Fig. 9 | Scanning electron microscopy (SEM) and transmission electron microscopy (TEM) images of the ZnO nano/microwires.
Supplementary Fig. 10 | XRD analysis of the ZnO nano/microwires.
Supplementary Fig. 12 | Piezoelectric nanogenerator (PENG) measurement to determine the c-axis orientation of the ZnO nano/microwire.
Supplementary Fig. 13 | Driving mode and calculation method of strain on DPT.
Supplementary Fig. 14 | Schematic illustration of the dual-modal piezotronic transistor (DPT) and the 2D simulation model of DPT in the finite element method (FEM) for the structural optimization.
Supplementary Fig. 15 | Simulated shapes of DPTs with various structure ratios (w/L_{NW}) under different pressures.
Supplementary Fig. 16 | Simulated results of the nano/microwire deformation in DPTs with various structure ratios (w/L_{NW}) under uniform pressures.
Supplementary Fig. 17 | The relationship between the downward displacement (d) of the top edge of microprotrusion and the applied pressure in DPTs with various structure ratios (w/L_{NW}) for vertical force sensing obtained from the FEM simulation.
Supplementary Fig. 18 | Impact of the material properties of PDMS on the performance of the DPT in vertical force sensing mode.
Supplementary Fig. 19 | Impact of the material properties of PDMS on the relationship between the pressure and the downward displacement.
Supplementary Fig. 20 | Impact of the PET substrate on the performance of the DPT in vertical force sensing mode.
Supplementary Fig. 21 | Influence of the thickness of bottom PDMS layer on the performance of the DPT for vertical force sensing by FEM simulations.
Supplementary Fig. 22 | Impact of the thickness of top PDMS layer on the performance

of the DPT for vertical force sensing by FEM simulations.
Supplementary Fig. 23 | Impact of the PDMS thickness ratio of the top layer to the total on the DPT performance for vertical force sensing by FEM simulations.
Supplementary Fig. 24 | The FEM simulation results for the DPTs with different microprotrusion heights in vertical force sensing mode.
Supplementary Fig. 25 | Impact of the microprotrusion height on the DPT performance for vertical force sensing by the FEM simulation.
Supplementary Fig. 26 | Impact of the structure of the microprotrusion on the performance of the DPT for lateral strain sensing by the FEM simulation.
Supplementary Fig. 27 | Dimension of DPTs and method to achieve the structure ratio of 0.66.
Supplementary Fig. 28 | Experimental setup.
Supplementary Fig. 29 | The relationship of the vertical force on DPTs measured in different positions.
Supplementary Fig. 30 | Energy bands of device under different bias and different force.
Supplementary Fig. 31 | Influence of the image force induced by high bias on piezotronic modification of energy bands.
Supplementary Fig. 32 | Change of energy bands and Redistribution of voltage drops induced by high bias.
Supplementary Fig. 33 | Piezotronic modulation of the dominant Schottky barrier height (SBH) on electrical transport.
Supplementary Fig. 34 | Strain-induced symmetric modulation of electrical transport by piezoresistive effect.
Supplementary Fig. 35 | Strain-induced asymmetric modulation of electrical transport by piezoelectric charges (piezotronic effect).
Supplementary Fig. 36 | Resistance change ratio $(\Delta R/R_0)$ and current change ratio $(\Delta I/I_0)$ used to characterize the piezotronic device under forward bias and reverse bias.
Supplementary Fig. 37 | Sensing performance of the DPT.
Supplementary Fig. 38 | A possible method to solve the problem of crosstalk in DPT.

Supplementary Tables 1-6:

Supplementary Table 1 | Summary of piezoelectric coefficients from some piezoelectric materials
Supplementary Table 2 | Material parameters of the mechanical properties for PET, PDMS and ZnO in the finite element method (FEM) simulations.
Supplementary Table 3 | Summary of Young's moduli (E) of the PDMS samples with different base/agent ratios.
Supplementary Table 4 | Summary of pressure sensitivity of piezotronic sensors
Supplementary Table 5 | Comparison of sensitivity of force/pressure sensors
Supplementary Table 6 | Comparison of strain sensing works

References 1-127.

Supplementary Note 1 | Piezotronic linear regulation on interface barrier height

The metal-semiconductor contact is an important component in electronic devices, which can be simplified in terms of the charge distribution as shown in **Supplementary Fig. 1a** in the presence of a Schottky barrier [1-4]. The semiconductor side is assumed to be *n*-type, and the surface states and other anomalies are ignored for simplicity. Under strain, the generated piezoelectric charges at the interface not only change the height of the Schottky barrier, but also change its depletion layer width. Different from the method of changing the Schottky barrier height by introducing dopants at the semiconductor side, the piezopotential can be continuously tuned by strain for a fabricated device. Here, the piezoelectric potential distribution and related electric field in the Schottky junction can be calculated by simplifying the Poisson equation as follows:

$$-\frac{d^2\psi_i}{dx^2} = \frac{dE}{dx} = \frac{\rho(x)}{\varepsilon_s} = \frac{1}{\varepsilon_s}\left[qN_D(x) - qn(x) + qp(x) + q\rho_{piezo}(x)\right] \tag{1}$$

in which $N_D(x)$ is the donor concentration, $\rho_{piezo}(x)$ is the polarization charge density. Then W_{Dn} is defined as the width of the depletion layers on the *n*-type semiconductor side, and the electric fields in **Supplementary Fig. 1b** at different positions can be expressed by combining these equations:

$$E(x) = -\frac{q[N_D(W_{Dn} - x) + \rho_{piezo}(W_{piezo} - x)]}{\varepsilon_s}, \text{for } 0 \le x \le W_{piezo} \tag{2}$$

$$E(x) = -\frac{qN_D}{\varepsilon_s}(W_{Dn} - x), \text{for } W_{piezo} \le x \le W_{Dn} \tag{3}$$

Thus, the maximum electric field (E_m) at the interface (x=0) is given by

$$|E_m| = \frac{q(N_D W_{Dn} + \rho_{piezo} W_{piezo})}{\varepsilon_s} \tag{4}$$

In addition, the potential distribution $\psi_i(x)$ in **Supplementary Fig. 1c** can be expressed as:

$$\psi_i(x) = \psi_i(0) + \frac{q[N_D\left(W_{Dn} - \frac{x}{2}\right)x + \rho_{piezo}(W_{piezo} - \frac{x}{2})x]}{\varepsilon_s},$$

$$\text{for } 0 \le x \le W_{piezo}$$

(5)

$$\psi_i(x) = \psi_i(W_{piezo}) - \frac{qN_D}{\varepsilon_S}\left(W_{Dn} - \frac{W_{piezo}}{2}\right)W_{piezo} + \frac{qN_D}{\varepsilon_S}\left(W_{Dn} - \frac{x}{2}\right)x,$$
$$\text{for } W_{piezo} \le x \le W_{Dn}$$

(6)

Then, the built-in potential ψ_{bi} can be calculated as

$$\psi_{bi} = \frac{q}{2\varepsilon_s}[\rho_{piezo}W_{piezo}^2 + N_D W_{Dn}^2]$$

(7)

From the above equations, the change in the built-in potential derives from the piezoelectric charges generated through the applied strain. Here, the sign of the local piezoelectric charges is determined by tensile or compressive strain. It should be noted that the built-in potential without piezopotential can be expressed as

$$\psi_{bi0} = \frac{q}{2\varepsilon_s} N_D W_{Dn}^2$$

(8)

If positive piezoelectric charges or potential is generated at interface by strain, the sign of the local piezoelectric charges is a positive value, and the built-in potential will change a value as follows

$$\psi_{bi} - \psi_{bi0} = -\frac{q\rho_{piezo}W_{piezo}^2}{2\varepsilon_s}$$

(9)

In other words, the built-in potential (of Schottky barrier formed between metal and *n*-type semiconductor just as set before) will increase as positive charges generated at interface.

Obviously, the piezoelectric potential can effectively tune the band of the junction corresponding to the Fermi levels. From equations (1) - (9), we can obtain the Fermi level (E_F) with a piezopotential at the interface of the Schottky junction as follow:

$$E_F = E_{F0} - \frac{q^2\rho_{piezo}W_{piezo}^2}{2\varepsilon_s}$$

(10)

in which E_{F0} is the Fermi level in the absence of a piezopotential.

Here, the piezoelectric charges are assumed to function as a perturbation to the conduction band edge E_C at the interface of the Schottky junction. Thus, the change $\Delta\varphi_{piezo}$ in the Schottky barrier height (SBH) arising from the tuning of the piezoelectric charges can be calculated as

$$\varphi_{Bn} = \varphi_{Bn0} - \frac{q^2 \rho_{piezo} W_{piezo}^2}{2\varepsilon_s}$$

(11)

Thus, we can obtain that the density of the piezoelectric charges (ρ_{piezo}) linearly regulates the change of barrier height ($\Delta\varphi_{piezo}$).

$$\varphi_{Bn} - \varphi_{Bn0} = \Delta\varphi_{piezo} = -\frac{q \rho_{piezo} W_{piezo}^2}{2\varepsilon_S}$$

(12)

in which φ_{Bn} and φ_{Bn0} are the Schottky barrier height with and without the presence of piezoelectric charges, respectively.

Furthermore, the piezoelectric coefficient matrix of wurtzite structure ZnO can be written as [1, 2]

$$(e)_{ijk} = \begin{bmatrix} 0 & 0 & 0 & 0 & e_{15} & 0 \\ 0 & 0 & 0 & e_{15} & 0 & 0 \\ e_{31} & e_{31} & e_{33} & 0 & 0 & 0 \end{bmatrix}$$

(13)

As the major polarization axis of ZnO is the *c*-axis, normally in wurtzite crystals, the longitudinal direction of a ZnO nano/microwire is along the *c*-axis. Based on the piezoelectric effect, the piezoelectric polarization of the ZnO nano/microwire at the contact in this case can be expressed as

$$P_z = e_{33} s_{33} = q \rho_{piezo} W_{piezo}$$

(14)

in which e_{33} is the piezoelectric constant, s_{33} represents the strain produced along the *c*-axis of ZnO nano/microwire.

From equation (14), it can be concluded that there is a linear correlation between piezoelectric polarization (P_z) and strain (s_{33}), as well as the density of piezoelectric

polarization charges (ρ_{piezo}).

So,

$$P_z \propto s_{33} \propto \rho_{piezo} \tag{15}$$

According to equation (12), we can obtain that

$$\Delta\varphi_{piezo} \propto \rho_{piezo} \propto s_{33} \tag{16}$$

Thus, the force/strain-induced piezoelectric polarization charges and corresponding potentials produced at the interfaces linearly regulate the interface barrier height.

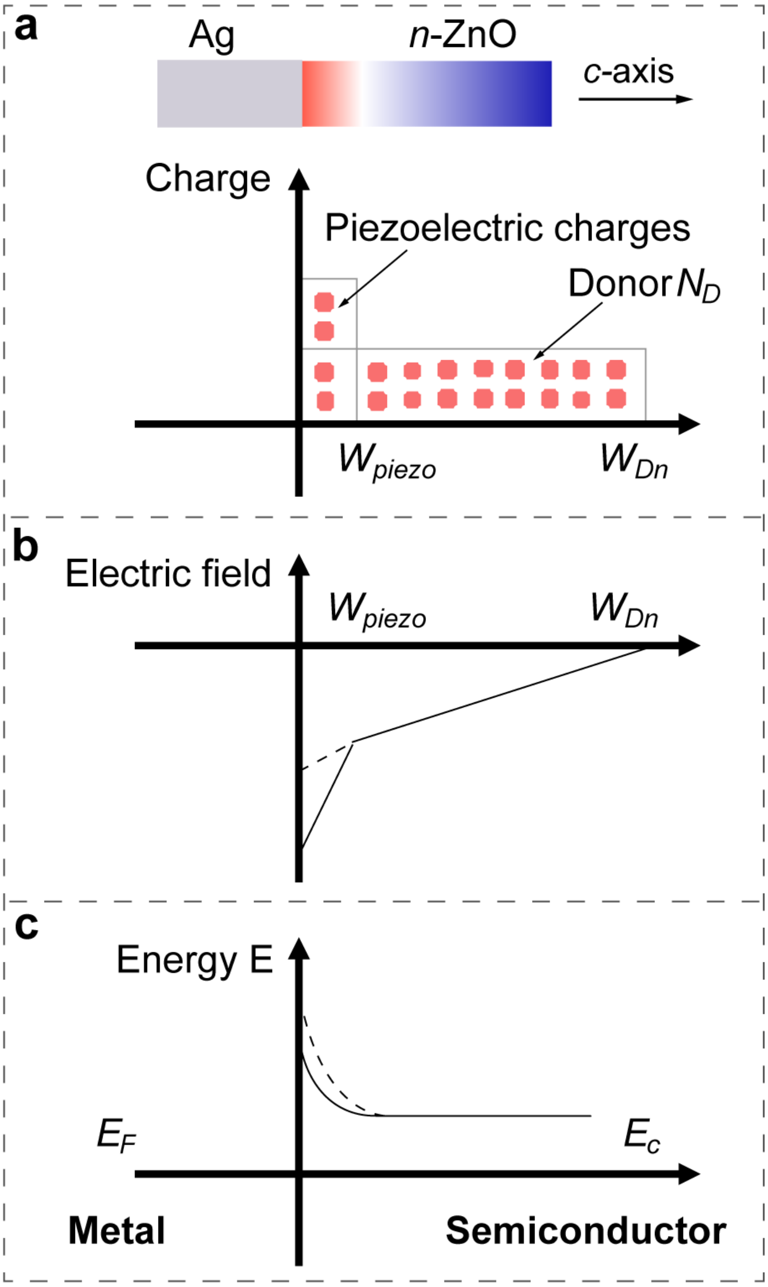

Supplementary Fig. 1 | Ideal metal-semiconductor Schottky contact with the presence of piezoelectric charges at an applied voltage $V = 0$ (thermal equilibrium). a, Space charge distribution. **b** and **c**, Electric field (**b**) and energy band diagram (**c**) in the presence of piezoelectric charges. Dashed lines indicate the electric field and energy band in the absence of piezoelectric charges and the solid lines indicate the cases when a piezopotential is present in the semiconductor.

Supplementary Note 2 | Design idea of DPT for performance improvement and functional expansion (Novelty of DPT)

The design of DPT with two sensing modes is a qualitative leap in both functional expansion and performance improvement. In order to clarify the novelty of DPT in a clear and detailed way, we will discuss (1) the challenges long-term faced in piezotronics, and (2) the structural evolution of the piezotronic transistors in the past to solve the problems/challenges, and (3) the original design idea of DPT according to our thinking about the key point of the challenges in piezotronics, and (4) the achievements regarding both the performance improvement and functional expansion of piezotronic transistors realized by the innovative design of DPT.

(1) Challenges long-term faced in piezotronics

Piezotronic transistor, utilizing piezoelectric polarizations to control the interface barrier height thus to achieve the active interaction between electronics and external stimuli, is a kind of new principle device and exhibits great potential in mechanical sensing [3]. In principle, piezotronic transistors should possess inherent high sensitivity in sensing mechanical stimuli due to its natural exponential control of output signal by input signal. However, currently, piezotronic transistors still face many problems toward applications both in terms of functional integration and performance improvement.

As shown in **Supplementary Fig. 2**, a long-standing challenge is that the huge gap between the two typical structures (lateral structure and vertical structure) makes piezotronic transistor can only measure lateral strain or can only measure vertical force, which cannot meet the demand of actual applications that require the same sensor to switch between two working modes. Although there have been many reports of multidirectional, high-sensitivity strain/pressure sensors in recent years, it is still a big challenge for piezotronic transistor integrate vertical force sensing and lateral strain sensing into one device.

Another long-term challenge or problem affecting sensing performance, associated with piezotronic transistor from the invention to the present, is that the

traditional vertical structure of piezotronic transistor has a nano/microwire buckling effect upon vertical compressive force, which seriously limits the performance of piezotronic transistors. Due to this problem of buckling effect of nano/microwires (which will be detailly described and discussed in the following section (2)), the pressure sensitivity of piezotronic transistors is always kept within about 100 meV/MPa.

These two dilemmas have long existed since 2007 and have hindered the development and application of the piezotronic transistor. Actually, the design of DPT in this work is to simultaneously overcome above two challenges by absorbing the advantages of the traditional lateral structure but avoiding the disadvantages of the traditional vertical structure, thus to enable piezotronic transistor with two working modes and enhanced performance, which makes a qualitative leap in both function and performance of piezotronic transistors.

The key to successfully overcome the above two challenges lies in the vertical force sensing mode of DPT. Next, we will give priority to the structural evolution of the traditional vertical structure for vertical force sensing and the long-term existing problem (negative influence of buckling effect on piezotronic effect), and then derive our original design idea on the vertical force sensing mode of DPT step by step.

(2) Structural evolution of piezotronic transistors to solve the problem of buckling effect

The earliest design of using piezotronic effect to sense vertical force is a vertical structure composed of a vertical aligned wurtzite ZnO single crystal nano/microwire sandwiched and Schottky contacted by two electrodes (**Supplementary Fig. 3a** and Figure 1a-I). Since ZnO is a semiconductor possessing piezoelectricity, positive and negative piezoelectric polarization charges and potentials will be generated at the upper and the lower ends respectively upon a vertical compressive force according to the polarization axis (*c*-axis) shown in **Supplementary Fig. 3b**. These vertical-force-induced piezoelectric charges/potentials can control the electrical transport by modulating the Schottky barrier heights at interfaces formed between ZnO and electrodes. As a result, we can achieve the detection of the vertical force by measuring

the change of the electrical transport. This is the basic principle of the earliest design for vertical force sensing.

However, this structure has an unavoidable problem. When a vertical compressive force is applied on the vertical nano/microwire, the nano/microwire will withstand a compressive deformation along its length direction accompanied by bending deformation, which is known as buckling effect as illustrated in **Supplementary Fig. 3c**. We can imagine that a large part of the vertical force is consumed to bend the nano/microwire. As a result, the net compressive deformation along the nano/microwire and the corresponding generated piezoelectric charges/potentials at two ends of the nano/microwire both decreases as the buckling effect occurs, which has a negative influence on piezotronic modification on the electrical transport.

Additionally, the buckling effect in the vertical structure will also cause an uneven piezoelectric potential distribution at the ends of the nano/microwire as simulated in the enlarged inset of **Supplementary Fig. 3c** due to the bending nano/microwire with one side stretched and the other compressed. This leads a nonuniform piezotronic modulation on the interface Schottky barrier. Because the interface electrical transport is mainly determined by the region with the lowest barrier height, the nonuniform modulation caused by the buckling effect will also have a negative influence on the piezotronic modification on the electrical transport.

Due to the negative impact of the buckling effect (**Supplementary Fig. 3d**), the early piezotronic transistors based on vertical nano/microwires (**Supplementary Fig. 4a**) for vertical force sensing exhibits low pressure sensitivities (CdSe nano/microwire [5]: 0.677 meV/MPa; ZnO nano/microwire [6]: 0.083-0.422 meV/MPa; GaN nano/microwire [7]: 0.212-0.240 meV/MPa).

In order to weaken the buckling effect, nano/microwire clusters encapsulated by PDMS are used in 2012 as the core unit of piezotronic transistor (**Supplementary Fig. 4b**) [4]. In this design, the nano/microwire is bounded by the PDMS and the nano/microwires around, which is not easy to be bent upon a vertical compressive force. The piezotronic transistor based on this structural improvement achieves a pressure sensitivity up to 33.39 meV/MPa. However, the nano/microwires in the nano/microwire

clusters are not completely vertical, and there still exists buckling effect when subjected to the vertical compressive force.

In 2017, a design of replacing vertical nano/microwire or nano/microwire cluster with nanoplatelet is proposed to solve the problem of buckling effect as shown in **Supplementary Fig. 4c** [8]. Since the nanoplatelet has an aspect ratio (thickness-to-diameter) of about 0.42, it will not be buckle as the vertical nano/microwire subjected to a vertical force. The piezotronic transistor based on ZnO nanoplatelet exhibits pressure sensitivity of 60.97~78.23 meV/MPa, which is greatly improved from the ones based on vertical nano/microwires or nano/microwire clusters. However, due to nanoplatelets' small size both in thickness and diameter, it is very difficult to fabricate functional devices with furtherly enhanced performance. So, this design provides a possible solution to the problem of buckling effect; while it is not a feasible scheme for the time being.

The problem of the buckling effect in vertical nano/microwires, which weakens the piezotronic effect, is still not properly solved, and still restricts the performance of piezotronic transistors so far. Finding schemes to avoid the problem of buckling effect in nano/microwire-based piezotronic transistor is important for their applications in sensing.

(3) Original design idea of DPT according to our thinking about the key point of the challenges in piezotronics

From the above introduction about structural evolution of piezotronic transistors, we can find that the buckling effect mainly derived from the compression bending of the nano/microwire caused by the vertical compressive force (**Supplementary Fig. 5a**). A question (**Supplementary Fig. 5b**) arises, that is, can we utilize the elongation of nano/microwires rather than the compression of nano/microwires to achieve the detection of vertical force.

Based on this question and inspired by the suspension bridge (**Supplementary Fig. 5c**), we designed a structure composed of a lateral nano/microwire with two ends constrained by electrodes as illustrated in **Supplementary Fig. 5d-I**. If the vertical

force is applied on the middle of the lateral nano/microwire, the nano/microwire will be bent downward and elongated. In this way, we can use the elongation of lateral nano/microwire to sense the applied vertical force, and perfectly solve the problem of the buckling effect in the vertical structure. This is the prototype design of DPT.

It should be noted that the downward bending here is totally different from previously discussed bending of vertical nano/microwire in the problem of the buckling effect. The vertical force induced downward bending of the lateral nano/microwire here is to elongate the nano/microwire, thus to generate piezoelectric charges/potentials. As a contrast, the vertical compressive force induced bending deformation (buckling effect) of the vertical nano/microwire decreases the net compressive deformation along the nano/microwire, weakening the generated piezoelectric charges/potentials.

In addition to the above advantage of avoiding the problem of buckling effect, this new design has another advantage. As shown in **Supplementary Fig. 5d-I**, based on a very simple model, we can find that this newly designed structure should have high response in the detection of the vertical force. As a small vertical force F_{vertical} is applied on the middle of the nano/microwire, a very large tensile force $F_{lateral}$ approximately equaling to $F_{vertical}/(2 \cdot \cos\theta)$ (θ is the angle between nano/microwire and vertical line) would be produced along the length of the nano/microwire. Since θ approaches 90°, $F_{lateral}$ ($\approx F_{vertical}/(2 \cdot \cos\theta)$) is a very large value. Although this estimation model is very rough, it is qualitatively estimated that a small vertical force can indeed cause the nano/microwire to tighten and produce a very large tensile force along the nano/microwire.

Our earliest device structure is illustrated in **Supplementary Fig. 5d-II**. However, this structure is very easy to be damaged. Even a very small vertical force will cause the nano/microwire to be broken into two sections. Then, in order to avoid very large local stresses particularly at the positions of the force point and electrodes, we used PDMS to wrap the nano/microwire and designed the structure shown in **Supplementary Fig. 5e**. The soft nature of PDMS allows the nano/microwire to sustain a large enough tensile stress, which can avoid the nano/microwire fracturing when subjected to very large local stresses.

To further improve the performance, as illustrated in **Supplementary Fig. 5f**, a microprotrusion is applied to make the vertical force mainly concentrate on the middle part of the nano/microwire, thus more effectively elongating the nano/microwire rather than pressing the whole nano/microwire down.

Through the above thinking about the key point of the challenge in piezotronics and the exploration of the new design, we designed DPT possessing four features:

I. it has a basic principle of utilizing the response of lateral nano/microwire to vertical force to realize the vertical force sensing;

II. it utilizes elongation of nano/microwires (rather than compression of nano/microwires) to sense vertical force, which properly solves the problem of buckling effect in traditional vertical structure.

III. the structure of DPT inspired by suspension bridge can amplify the vertical force to the tensile force along the length of the nano/microwire;

IV. the soft nature of PDMS avoids extensive local stress, and the microprotrusion makes the vertical force concentrate on the middle of the nano/microwire, thus more effectively elongating the nano/microwire under the action of vertical force.

(4) Achievements regarding both the performance improvement and functional expansion of piezotronic transistors realized by the innovative design of DPT

The new design of DPT promotes the piezotronic transistor with both merits of performance improvement and functional expansion, as illustrated in **Supplementary Fig. 6**.

① *Performance improvement: high pressure sensitivity*

Based on the new design of DPT, we successfully achieve the piezotronic transistor with a pressure sensitivity of 1.759 eV/MPa, which is 16.5 times more than that of previously reported piezotronic transistors.

Comparing the differences and analyzing the progressive relationships between the DPT in this work and the piezotronic transistors in previous works, we can find that the high performance of DPT is not a simple structural optimization or parameter increasement, but results from the perfect solution to the problem of buckling effect

encountered by the development of piezotronics transistors in the past, and advanced structural design (containing suspension-bridge-inspired structure and introduction of microprotrusion) of amplifying the vertical force to the tensile force along the length of the nano/microwire.

② *Functional expansion: two working modes*

Fortunately, through previous discussions about the structural evolution in piezotronic transistors (Section (2)) and our research idea of DPT (section (3)), the design of DPT realizes the detection of vertical force with ultrahigh performance based on a new lateral structure; Meanwhile, the new design itself has a lateral structure similar to the traditional piezotronic transistor shown in **Figure 1a-II**, which makes the new design of DPT naturally suitable for lateral strain sensing. As a result, DPT with the new lateral structure that enables two working modes, one is vertical force sensing and the other is lateral strain sensing. DPT inherits all the advantages of the traditional lateral structure to detect the lateral strain, and can also detect the vertical force without the problem of buckling effect existed in the traditional vertical structure.

The design of DPT successfully overcomes the dilemma that piezotronic transistor can only be used for vertical force sensing or only be used for lateral strain sensing, and for the first time enables piezotronic transistor to possess two sensing functionalities and more possibilities in practical application, which would subvert the structural design of traditional piezotronic transistors and provide new ideas for new principle devices in the field of piezotronics to promote the application of piezotronic effect.

In summary, the design of DPT is a qualitative leap in both performance improvement and functional expansion in piezotronics (**Supplementary Fig. 6**). This is a big step in the field of piezotronics.

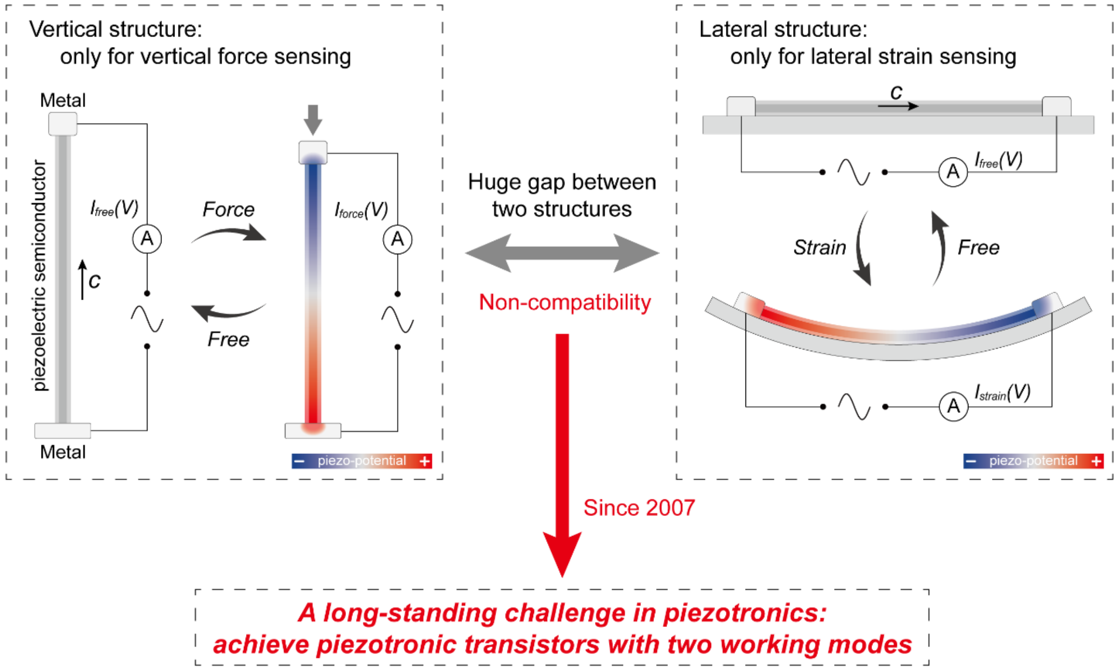

Supplementary Fig. 2 | A long-standing challenge in piezotronics. The left dotted box shows the traditional vertical structure of piezotronic transistors in the past composed of a vertical aligned nano/microwire sandwiched by two electrodes, which can only be used for vertical force sensing; while the right dotted box shows the traditional lateral structure of piezotronic transistors in the past possessing a lateral nano/microwire clamped by two electrodes, which can only be used for lateral strain sensing. Since 2007, the incompatibility of the two structures (vertical structure and lateral structure) of piezotronic transistor has made it impossible to achieve a same piezotronic transistor to switch between two working modes, which is a long-standing challenge in the field of piezotronics.

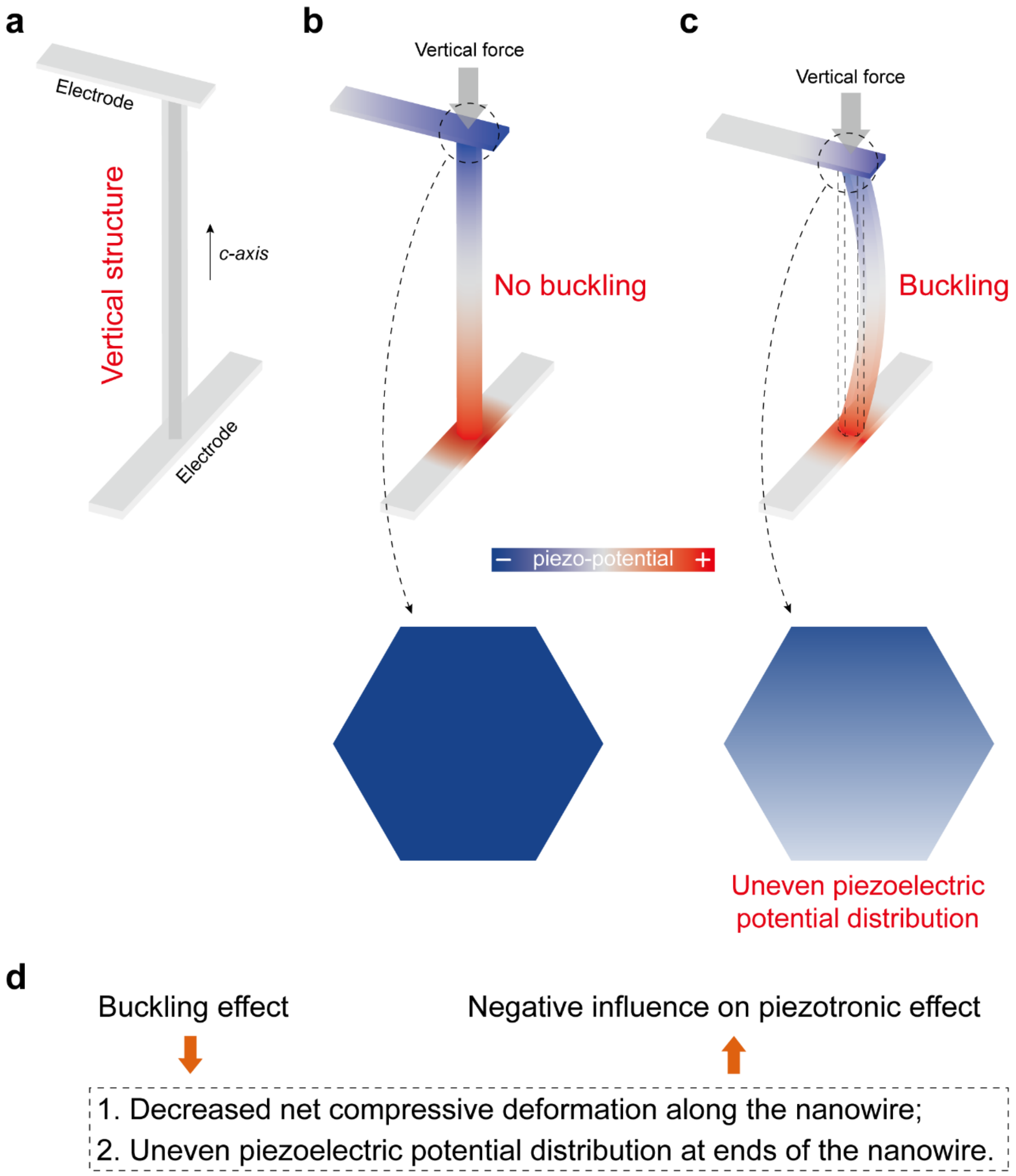

Supplementary Fig. 3 | Problem of buckling effect in vertical structure of piezotronic transistors. a, A piezotronic transistor with a vertical structure composed of a vertical aligned nano/microwire sandwiched by two electrodes. **b** and **c**, Illustrations of vertical force induced piezoelectric potential distributions without (**b**) and with (**c**) considering the buckling effect. The enlarged insets are illustrations of the potential distributions on the upper end of the nano/microwire. **d**, The buckling effect will lead to a decreased net compressive deformation along the nano/microwire and an uneven piezoelectric potential distribution at the ends of the nano/microwire, which has negative influence on piezotronic effect.

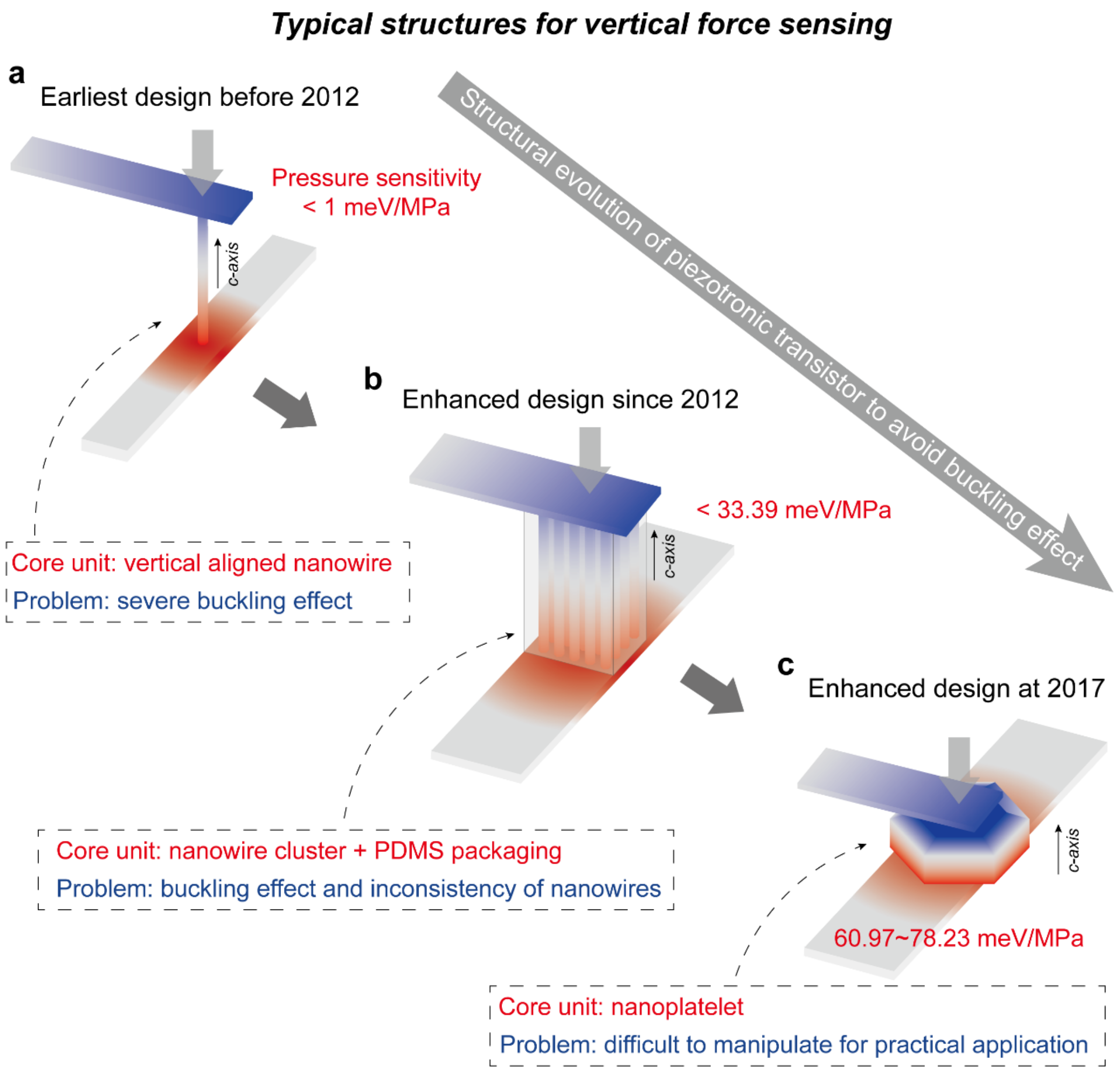

Supplementary Fig. 4 | Structural evolution of piezotronic transistors to solve the problem of buckling effect. a, The earliest design of piezotronic transistor for vertical force sensing. It is based on the vertical aligned nano/microwire but possesses a problem of severe buckling effect, which is not conducive to piezotronic effect. **b**, The enhanced design of piezotronic transistor based on nano/microwire cluster encapsulated by PDMS. Bounded by the PDMS and the nano/microwires around, vertical nano/microwire in the cluster is not easy to be bent (buckling effect) upon a vertical compressive force, which can enhance the performance to a certain extent. The still existing buckling effect and the inconsistency of nano/microwires hinder the further improvement of performance. **c**, A design based on nanoplatelet to solve the problem of buckling effect. It is difficult to manipulate the nanoplatelets for practical application.

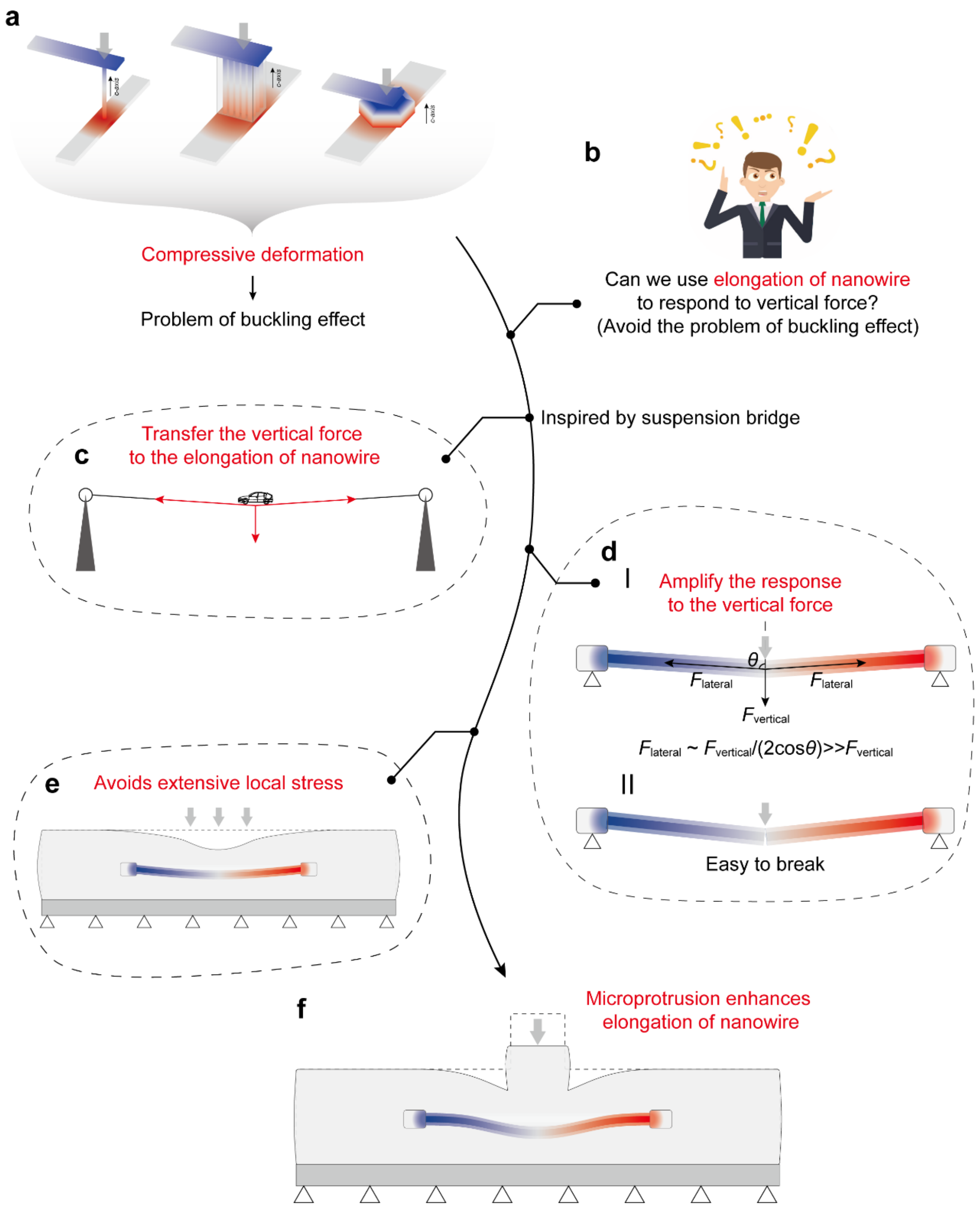

Supplementary Fig. 5 | Our original design idea of dual-modal piezotronic transistor (DPT). a, Traditional vertical structures with a common feature of utilizing compressive deformation caused by vertical force to sense the vertical force. This sensing principle is easy to induce the problem of buckling effect. **b**, A question raised after investigating the structural evolution of traditional piezotronic transistors to avoid the problem of buckling effect: can we use elongation of nano/microwire to respond to vertical force? **c**, A suspension-bridge-inspired structure that can transfer the vertical

force to the elongation of wire, and thus achieve elongation of nano/microwire to respond to vertical force. **d**, The earliest structure of DPT that can amplify the response to the vertical force. Directly applying the vertical force on the lateral nano/microwire can easily lead to the fracture of the nano/microwire. **e**, Using PDMS to encapsulate the lateral nano/microwire to avoid extensive local stress. **f**, Final design of DPT. Introduction of the microprotrusion makes the vertical force concentrate on the middle of the nano/microwire, thus more effectively elongating the nano/microwire under the action of vertical force.

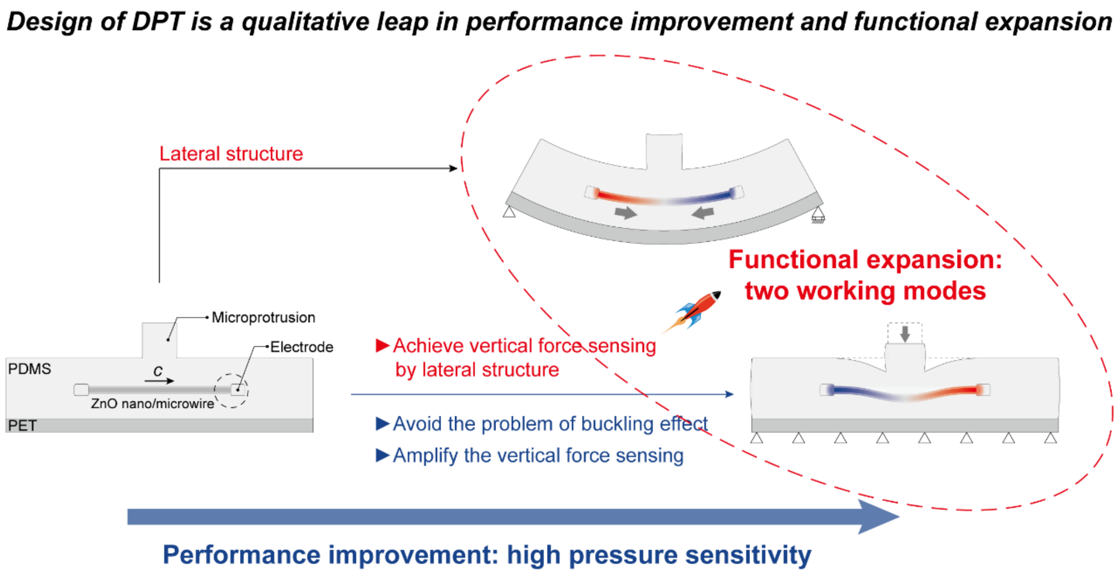

Supplementary Fig. 6 | Novelty of the dual-modal piezotronic transistor (DPT). In terms of performance improvement (blue font marked), DPT possesses high pressure sensitivity resulting from the perfect solution to the problem of buckling effect and advanced structural designs containing the suspension-bridge-inspired structure and introduction of microprotrusion that can amplify the vertical force sensing. In terms of functional expansion (red font marked), DPT enables one piezotronic transistor with two working modes for vertical force sensing and lateral strain sensing, which benefits from the new lateral structure design of DPT.

Supplementary Note 3 | Step-by-step fabrication of DPT

The DPT device is constructed on the flexible PET substrate (0.15 mm in thickness) with a soft adherent layer of PDMS. Before the preparation of PDMS layer, a clean PET film (50×50 mm^2) should first be tightly attached to the bottom of the lab dish. The mixture of PDMS (a prepolymer and a curing agent default mixing weight ratio 10:1) is then poured onto the PET substrate (**Supplementary Fig. 7a**), followed by 30 min of degassing in vacuum desiccator. When the thermal curing process is done, the PDMS layer with uniform thickness (~0.825 mm) would be formed on a PET film.

For the formation of the device, a single ZnO nano/microwire (its length is usually larger than 1.5 mm) could be physically picked up and transferred onto the as-fabricated PDMS-PET substrate with tweezers (**Supplementary Fig. 7b**). Owing to the geometrical shape, the ZnO nano/microwire would lay down on the flat surface of the substrate, while the growth direction of the ZnO nano/microwire (the piezoelectric polarization *c*-axis) is naturally parallel to the flat substrate horizontally.

Then, the PDMS-PET substrate together with the ZnO nano/microwire would be placed on the microscope stage (with calibration ruler under the substrate) as shown in **Supplementary Fig. 8a**. By rotating the PDMS-PET substrate with the ZnO nano/microwire, we can align the nano/microwire very accurately with the calibration ruler under the microscope (**Supplementary Fig. 8b**). Furtherly cut the substrate into the desired shape with dimension of 30 mm × 10 mm based on the calibration ruler (**Supplementary Fig. 8c**). In this way, the alignment of ZnO nano/microwire length's direction with the PDMS substrate's length direction is achieved.

To realize Schottky contact, the source/drain Ag electrodes are deposited on both ends of the nano/microwire by RF-magnetron sputtering, which is furtherly fixed by silver paste, to improve the interface property. Furthermore, the additional 0.5 mm of PMDS layer was also deposited on the ZnO nano/microwire as a top dielectric layer after curing at 100 °C for 1 hour (**Supplementary Fig. 7c**). Finally, the as-prepared PDMS microprotrusion could be mechanically released from the mold, and then carefully transferred and bonded onto the top of the device under the microscope,

considering the transparent encapsulation of the whole device (**Supplementary Fig. 7d**).

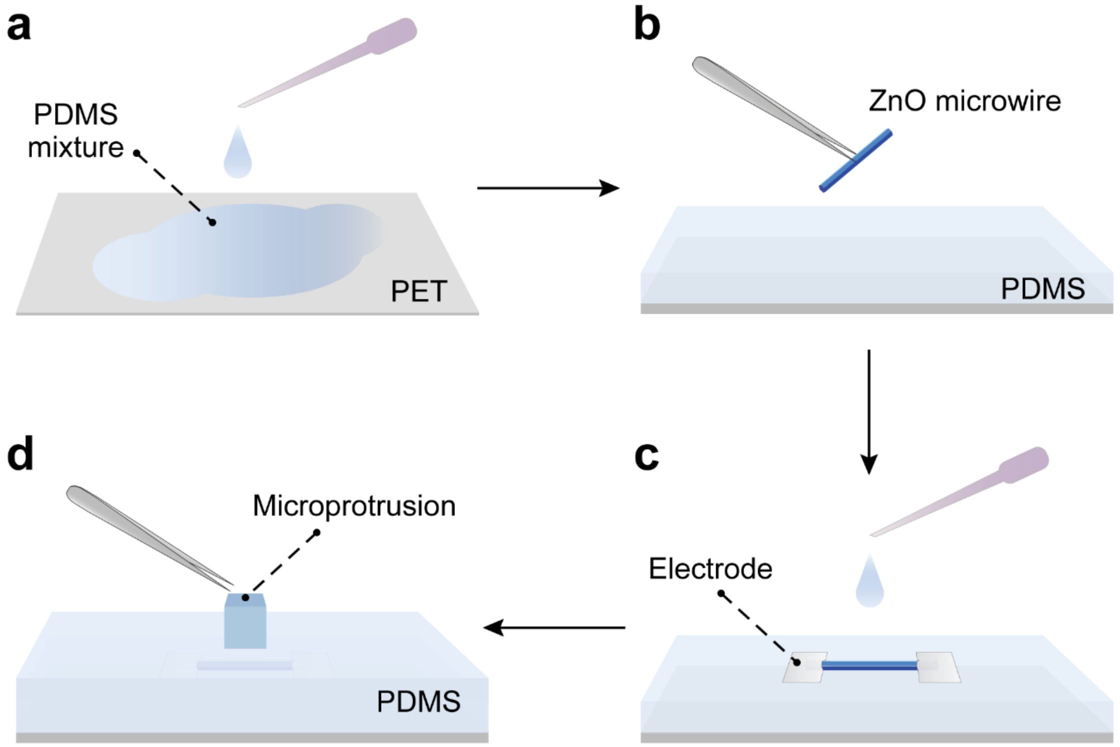

Supplementary Fig. 7 | Schematic diagram showing the fabrication process of the DPT. a, Preparation of PDMS layer on clean PET film. **b**, Transferring a single ZnO nano/microwire on PDMS-PET substrate. **c**, Deposition of Ag source/drain electrodes and PDMS encapsulation layer. **d**, Assembling DPT with PDMS microprotrusion.

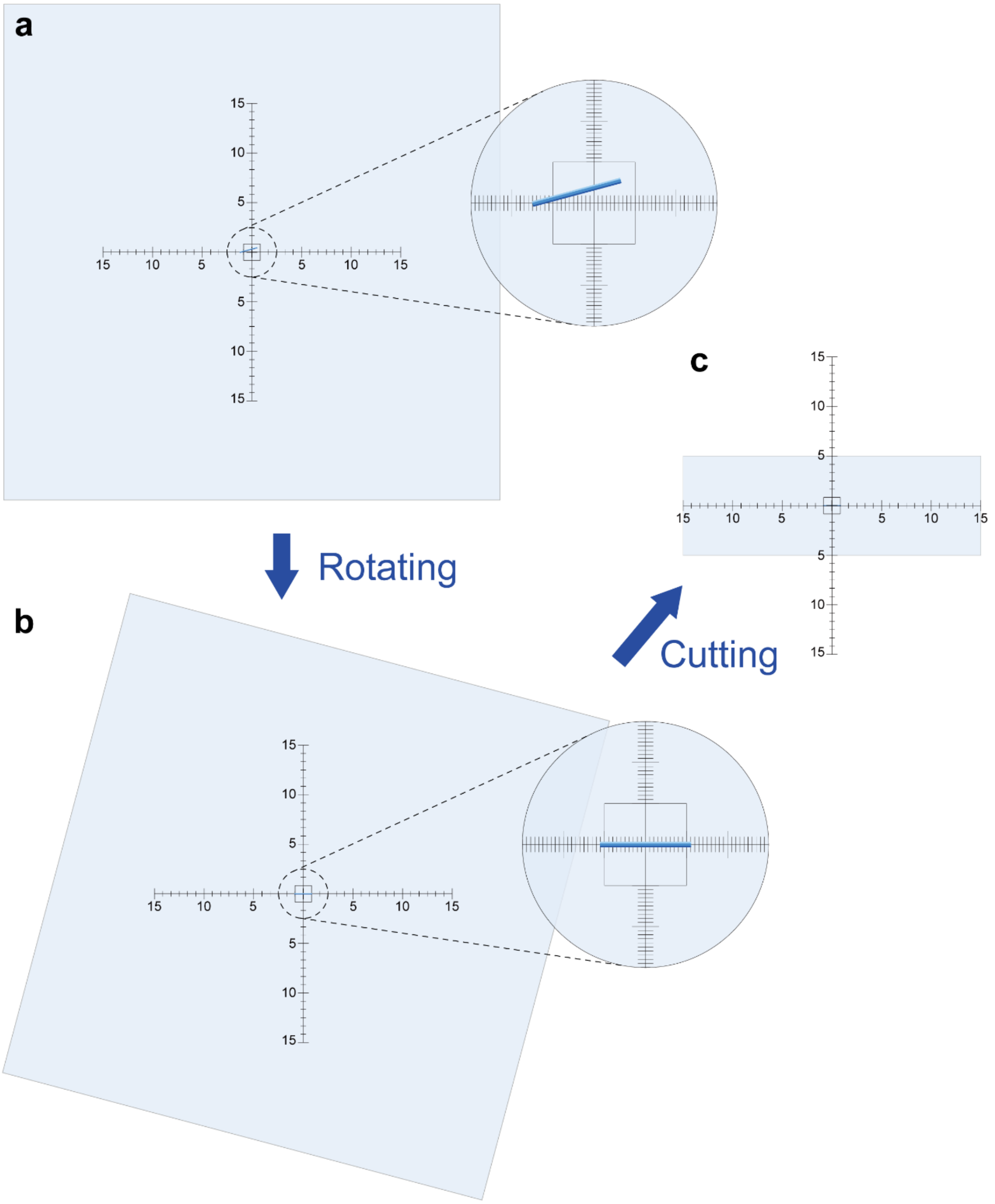

Supplementary Fig. 8 | Schematic illustration of the ZnO nano/microwire alignment. a, The PDMS-PET substrate is placed on the calibration ruler under microscope. **b**, Aligning the nano/microwire to the calibration ruler by rotating the PDMS-PET substrate. **c**, Cutting the substrate with aligned ZnO nano/microwire into the desired shape based on the calibration ruler. The insets show the enlarged images of the central region indicated by dashed lines.

Supplementary Note 4 | Material characterization

The morphology and structure of the synthesized ZnO nano/microwires were characterized by FE-SEM (Apreo HiVac, FEI) and TEM (JEM-2100F, JEOL), as shown in **Supplementary Fig. 9**. It can be found that the ZnO nano/microwire possesses a clean surface and a typical hexagonal geometry, which indicates that the wurtzite-structured nano/microwire grows along the *c*-axis. The X-ray diffraction (XRD) spectrum of the ZnO nano/microwires was also performed by X-ray diffractometer (D8 ADVANCE, Bruker) in **Supplementary Fig. 10**, confirming the monocrystalline nature of the nano/microwires with wurtzite structure.

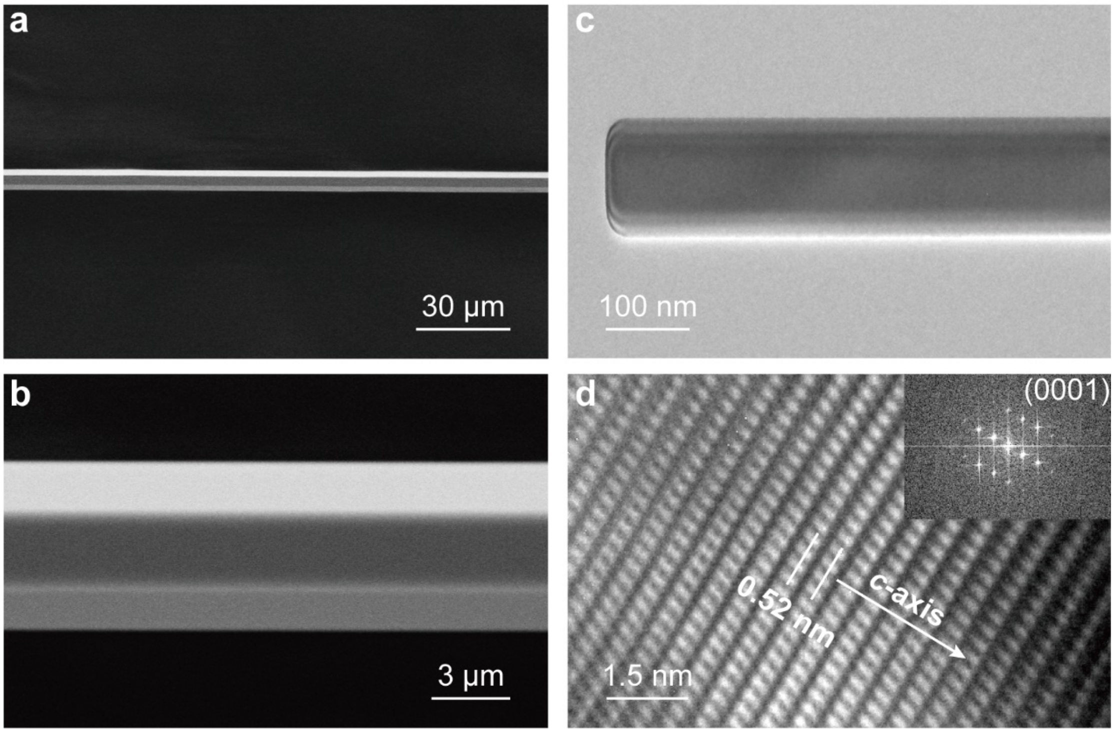

Supplementary Fig. 9 | Scanning electron microscopy (SEM) and transmission electron microscopy (TEM) images of the ZnO nano/microwires. a and **b**, SEM images of a ZnO microwire used in this work with the diameter of about 6.5 μm. **c** and **d**, TEM and HRTEM images of the as-synthesized ZnO nanowire. The inset in (**d**) shows the selective area electron diffraction (SAED) pattern from the nanowire.

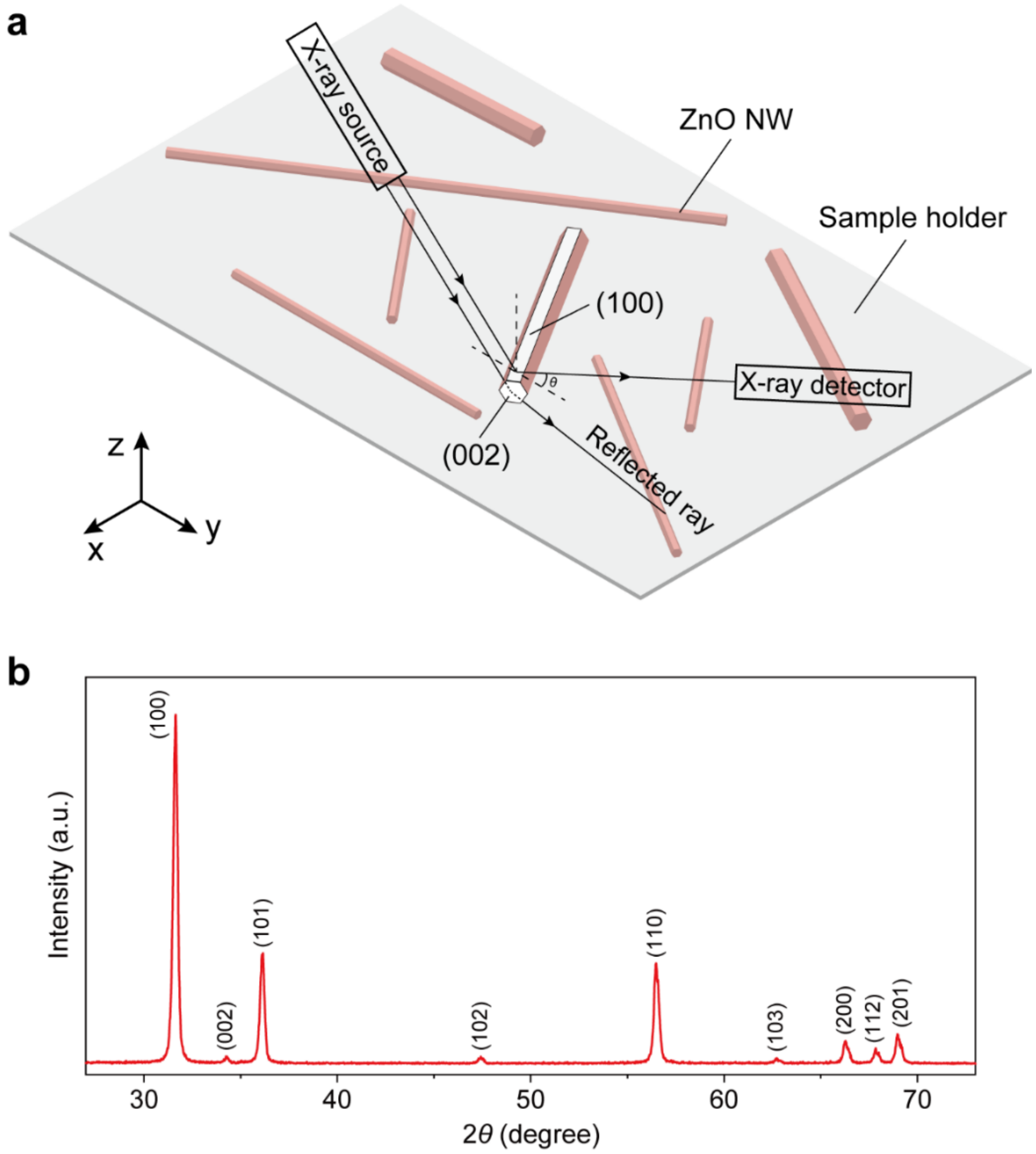

Supplementary Fig. 10 | XRD analysis of the ZnO nano/microwires. a, Schematic diagram of the experiment setup for XRD measurement of the ZnO nano/microwires. **b**, The XRD spectrum of the ZnO nano/microwires. The corresponding characteristic peaks are (100), (002), (101), (102), (110), (112) and (201), which fully match those in previous reports related to ZnO nano/microwire [9]. The reason why the (100) peak is higher than the (002) peak is mainly because the nano/microwires are lying on the sample holder, so that the (100) crystal plane is parallel to the plane where the sample holder is located.

Supplementary Note 5 | Piezoelectricity in wurtzite ZnO

The piezotronic transistor in our experiment is typically designed by using the wurtzite ZnO nano/microwires, whose crystal structure is illustrated in **Supplementary Fig. 11a**. When subjected to axial strain, the crystal with non-centrosymmetric structure will generate piezoelectric polarization charges within thickness of one to two atomic layers at both terminals in the ZnO nano/microwire along *c*-axis, which is usually the growth direction of the nanowire [10]. The piezoelectricity of ZnO is the key factor that directly determines the performance of the piezotronic transistor. Here, we utilized AFM (Cypher ES, Asylum Research) with PFM mode to investigate the piezoelectricity of the ZnO microwire. According to the slopes of the two linear fitting curves to the corresponding piezoresponse amplitude versus the tip voltage applied during the PFM measurement, the effective piezoelectric coefficient d_{33} of the ZnO microwire is approximately equal to 12 pm/V (**Supplementary Fig. 11b**), which is a relatively high value among piezoelectric materials (**Supplementary Table 1**). With the excellent property of vertical piezoelectricity, the ZnO microwire is an ideal candidate for the dual-modal piezotronic transistor (DPT).

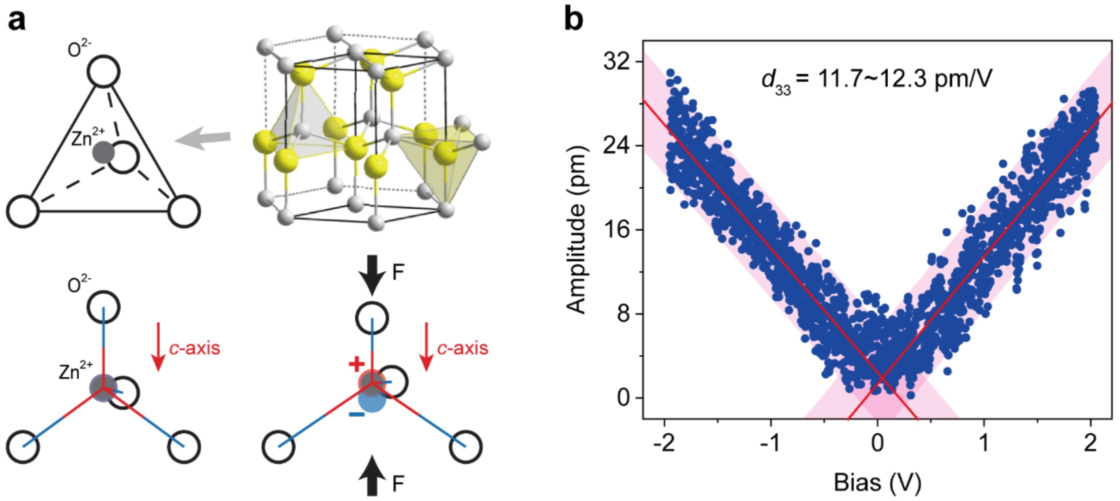

Supplementary Fig. 11 | Piezoelectricity in wurtzite-structured ZnO nano/microwire. a, Schematic diagram of piezoelectric polarization in ZnO with wurtzite structure. **b**, Piezoresponse measurement of the ZnO microwire by PFM, indicating an effective piezoelectric coefficient d_{33} of 11.7~12.3 pm/V.

Supplementary Table 1 | Summary of piezoelectric coefficients from some piezoelectric materials

Materials	Morphology	Piezo. Coefficient d_{33} (pm/V)	Reference
ZnO	Microwire	11.7~12.3	This work
ZnO	Bulk	12.4±1.1	[11]
ZnO	Bulk	~9.93	[12]
ZnO	Microtube	16.98±0.5	[13]
ZnO	Nanopillar	7.5±0.6	[14]
ZnO	Nanorod	4.41±1.73	[15]
ZnO	Nanorod	0.4-9.5	[16]
ZnO	Nanobelt	14.3-26.7	[12]
ZnO	Nanoplatelet	18.9-22.5	[8]
ZnO	Nanosheet	~23.7	[17]
AlN	Film	5.1±0.1	[18]
GaN	Bulk	2.0±0.1	[19]
GaN	Film	3.1±0.1	[18]
GaN	Nanowire	~12.8	[20]
CdS	Bulk	~9.71	[21]
CdS	Nanosheet	~16.4*	[22]
MoS_2	Nanosheet	1.35±0.24	[23]
α-$GaPO_4$	Nanosheet	7.5±0.8	[24]
α-In_2Se_3	Nanoflake	0.34-5.6	[25]
$NaNbO_3$	Nanowire	0.85-4.26	[26]
$KNbO_3$	Nanowire	~7.9	[27]
PZT	Nanowire	~114	[28]
TMCM-$MnCl_3$	Film	~185	[29]

*The effective d_{33} was later multiplied by 2, resulting in a piezoelectric constant of 32.4 pm/V.

Supplementary Note 6 | Piezoelectric nanogenerator measurement to determine the polarity of the ZnO nano/microwire

Piezopotential (piezoelectric potential) generated by the strain-induced piezoelectric polarization charges in piezoelectric semiconductors forms the basis of the piezotronics and the piezoelectric nanogenerator (PENG) [30]. In order to determine the direction of the polarization *c*-axis of the ZnO nano/microwires used in this work, the PENG measurements were carried out here.

As for a PENG based on a single ZnO nano/microwire with a right-pointed *c*-axis direction as illustrated in **Supplementary Fig. 12a**, when the flexible substrate bends downward to induce compressive strain inside the piezoelectric microwire, the electrons in the external circuit will get repelled by the negative piezoelectric charges and the corresponding negative piezopotential created at the *n*-ZnO/Ag interface on the right side of the ZnO nano/microwire, leading to the positive output current from left to right. And when the substrate is released, the disappearance of the piezopotential will drive the electrons accumulated near the left electrode to flow back in the opposite direction. In other words, an alternating current will be generated by bending and releasing the PENG periodically [31]. Therefore, the direction of the *c*-axis of the nano/microwire can be inferred by looking at whether a positive or a negative current peak occurs first. **Supplementary Fig. 12b** shows a typical experimental data of a PENG measurement. As can be seen, a positive current peak occurs first followed by a negative current peak. According to the aforementioned standard of judgment, the *c*-axis direction of the ZnO microwire can be confirmed to point right.

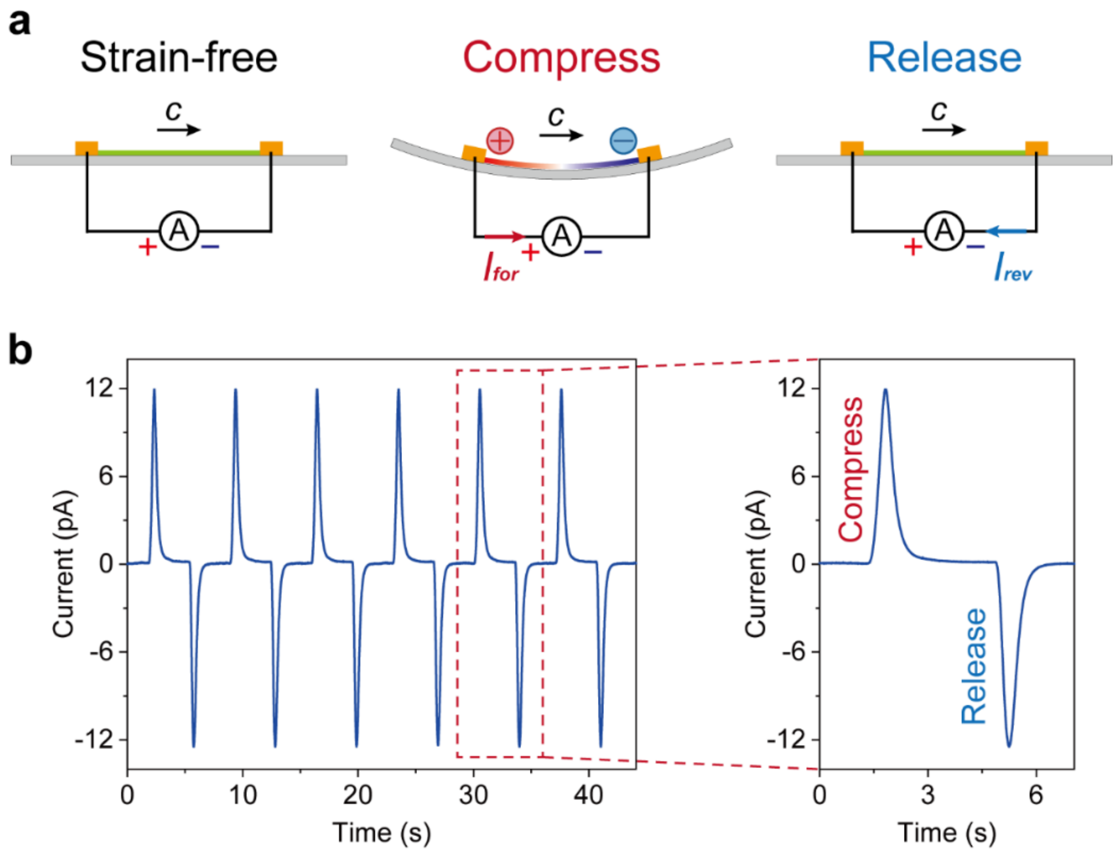

Supplementary Fig. 12 | Piezoelectric nanogenerator (PENG) measurement to determine the *c*-axis orientation of the ZnO nano/microwire. a, Schematic diagrams of the mechanism for the PENG based on a single nano/microwire upon compressive strain. **b**, The current output of PENG during the compress-release cycles to determine the *c*-axis direction of the ZnO nano/microwire.

Supplementary Note 7 | Driving mode and calculation method of strain on DPT

In order to accurately calibrate the strain applied to the nano/microwires, we designed the driving mode of the strain, and applied finite element method (FEM) simulation to obtain the strain in consideration of the PDMS Poisson 's ratio of 0.49.

As shown in **Supplementary Fig. 13a**, we fix the DPT onto the inner surface of a steel coil, and then apply strain to the DPT by changing the diameter of the steel coil. In this case, we can confirm that the shape of the PET is a circular arc. By measuring the distance change ΔL between the two ends of the PET or the radius of the steel coil in this case, combined with the FEM simulation, we can get a relatively accurate strain. It should be noted that the boundary condition of PET is a circular arc in the FEM simulation. **Supplementary Fig. 13b** shows the calibrated strain that is applied on DPT in the strain sensing mode by the calculation method using steel coil and FEM simulation.

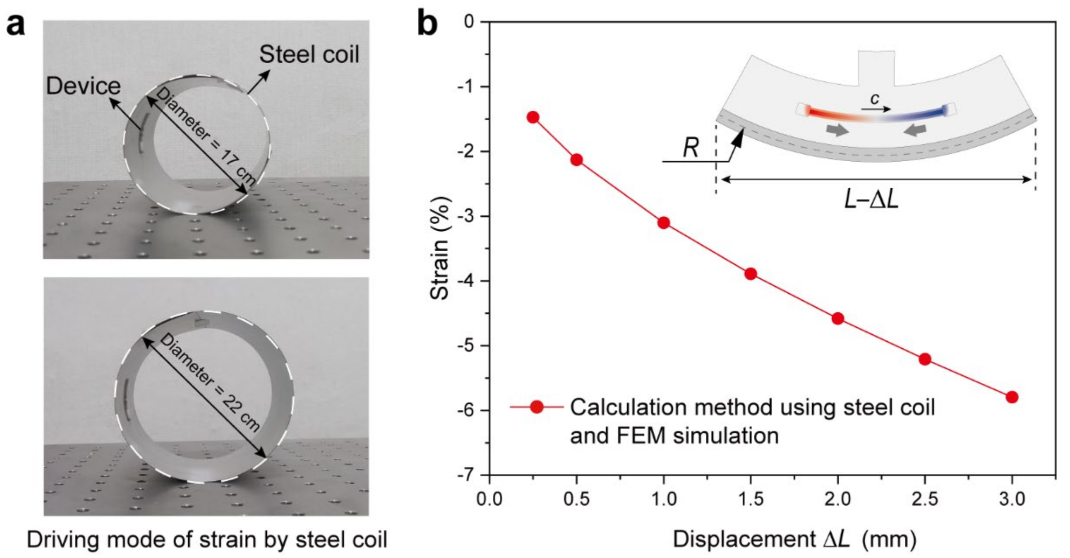

Supplementary Fig. 13 | Driving mode and calculation method of strain on DPT. **a**, Driving mode of strain by steel coil on DPT for lateral strain sensing. **b**, Calibrated strain as a function of the displacement (distance change) ΔL by using steel coil and FEM simulation.

Supplementary Note 8 | Simulations for the structural optimization of the DPT

In addition to the simulations in **Figure 2** of the manuscript, we also carried out some other simulations for the structural optimization of the DPT as follows.

(1) Detailed information of the finite element method (FEM) simulation

The schematic representation of the FEM simulation in the structural optimization of the DPT is shown in **Supplementary Fig. 14**. As illustrated in the schematic diagram of the exploded view layout (**Supplementary Fig. 14a**) and the side view (xy plane) of the DPT in vertical force sensing mode (**Supplementary Fig. 14b**), a ZnO nano/microwire is embedded into two PDMS layers on a PET substrate, with a microprotrusion bonded onto the top PDMS layer just above the ZnO nano/microwire. Because of the extremely small diameter of the ZnO nano/microwire (usually in the micron scale, such as about 6.5 μm) compared with the size of the microprotrusion (in the millimeter scale) and the PDMS layer (in the centimeter scale) in the direction of the z-axis (perpendicular to the xy plane), the influence of the morphology of ZnO nano/microwire on the deformation induced by the vertical force can be negligible in this work. Considering the above structural characteristics, it is reasonable for us to construct a 2D finite element model in plane stress condition using the software COMSOL Multiphysics to simulate the loading process of the vertical force and the corresponding shape change of the DPTs with various structure parameters.

As shown in **Supplementary Fig. 14c**, the model is constructed by two PDMS layers on a PET substrate, with a square PDMS as the microprotrusion just above the embedded ZnO nano/microwire. For convenience, the diameter of the ZnO nano/microwire can be ignored in the FEM model as considering its extremely small diameter compared with the thicknesses of PDMS and PET. Thus, we assume that a ZnO nano/microwire with length $L_{NW} = 1\ mm$ exists at the middle of the interface between the two PDMS layers. In order to investigate the influence of the microprotrusion structure ratio (w/L_{NW}) on the ZnO nano/microwire deformation upon a vertical force and the sensitivity of the DPT, we model the microprotrusion with width (w) in the range from 0.25 mm to 1.25 mm, while the height remains constant

($h_{protrusion} = 1\ mm$). **Supplementary Fig. 14d** gives the cases with w/L_{NW} of 0.25 and 1.25. Besides, the length of PDMS layers and PET substrate is $L = 30\ mm$; and the thicknesses of the top PDMS layer, the bottom PDMS layer and the PET substrate are $h_{PDMS,top} = 0.5\ mm$, $h_{PDMS,bottom} = 0.825\ mm$ and $h_{PET} = 0.15\ mm$, respectively. Under the boundary condition that the bottom edge of the PET substrate is fixed constraint, the vertical force on the top edge of the microprotrusion is applied by pressing a certain downward displacement (d) as shown in **Supplementary Fig. 14b**. Additionally, the main parameters of the material properties for the mechanical simulation are taken from the material library database in the software of COMSOL Multiphysics as well as the reported values in the literature (**Supplementary Table 2**).

Supplementary Table 2 | Material parameters of the mechanical properties for PET, PDMS and ZnO in the finite element method (FEM) simulations.

Materials	Young's modulus, $\boldsymbol{E}$	Poisson's ratio, $\boldsymbol{\nu}$	Reference
PET	4 GPa	0.35	[32]
PDMS	750 kPa	0.49	[33]
ZnO	210 GPa	0.33	[34]

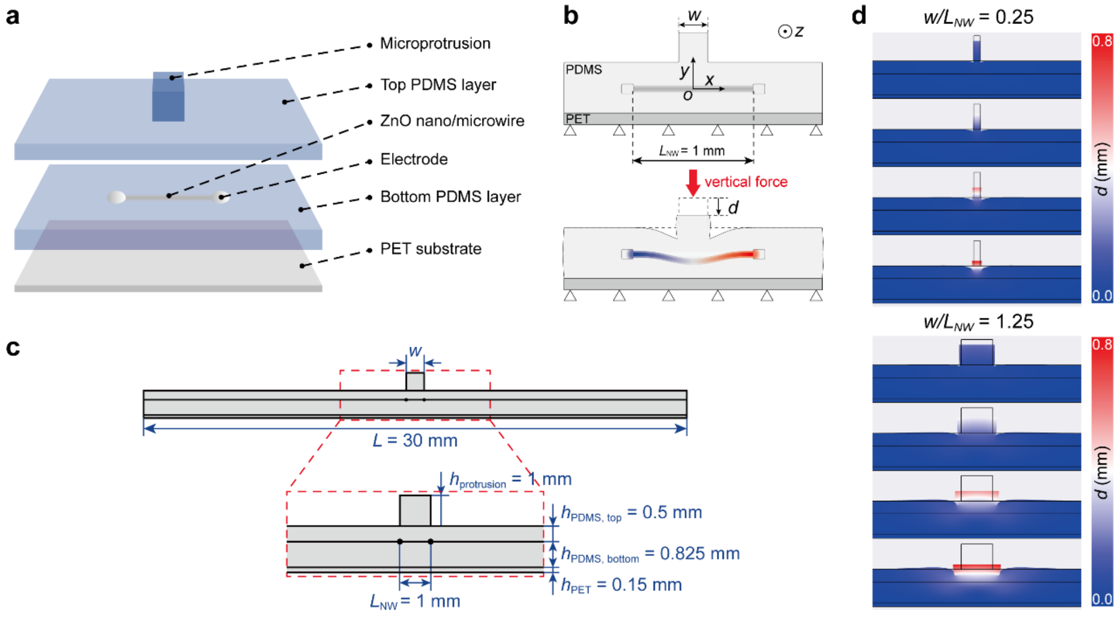

Supplementary Fig. 14 | Schematic illustration of the dual-modal piezotronic transistor (DPT) and the 2D simulation model of DPT in the finite element method (FEM) for the structural optimization. a and **b,** Schematic illustrations of the exploded view layout (**a**) and the side view at the *xy* plane (**b**) of the DPT in vertical force sensing mode. The hollow triangle symbol represents the fixed constraint. **c**, Schematic illustration of the FEM model of the DPT with the detailed structure parameters. Black points represent ends of the ZnO nano/microwire. **d**, The corresponding displacement distribution profiles of DPT with various structure ratios (w/L_{NW} = 0.25 and 1.25) in simulating the deformation against with different vertical displacements *d*.

(2) Influence of the microprotrusion structure ratio (w/L_{NW}) on the performance of the DPT under the same external force

To better understand the structural characteristics of the microprotrusion in DPT, we systematically investigate the downward displacement (d) of the top edge of microprotrusion and the nano/microwire deformation together with the strain induced by different external vertical force in DPTs with different microprotrusion structure ratio (w/L_{NW}). As previously discussed in (1), the influence of the geometry of the microprotrusion along *z*-axis on the nano/microwire deformation under vertical force

can be approximately neglected. So, the external force evenly applied to the top surface of the microprotrusion can be denoted as the contact pressure ($P = F/S$) through keeping the loaded area (or the area of the top surface) unchanged, to avoid interference of the loaded area (S) and other insignificance factors.

In order to further confirm the optimal structure ratio of the microprotrusion and give a convincing demonstration, we simulated the mechanical deformation process of the ZnO nano/microwire in DPTs with various microprotrusion widths (w) under uniform pressure as illustrated in **Supplementary Fig. 15a**. Under the application of the loading pressure varying from 0.15 MPa to 0.6 MPa on the top edge of microprotrusion as shown in **Supplementary Fig. 15b**, the calculated spatial distribution of displacement profiles would clearly demonstrate the shape change of the DPTs with different microprotrusion structure ratios (w/L_{NW}) in the range of 0.25~1.25.

Furthermore, the corresponding outlines of the bended nano/microwires under different conditions in **Supplementary Fig. 15b** could be obtained and plotted in **Supplementary Fig. 16a**. It can be seen from **Supplementary Fig. 16b** that with the application of the pressure of 0.6 MPa, a small structure ratio (for example, $w/L_{NW} = 0.25$) of the microprotrusion tends to induce the bending deformation of the nano/microwire; while a large structure ratio (for example, $w/L_{NW} = 1.25$) is more likely to cause the overall displacement of the nano/microwire. Note that the lengths of these bended nano/microwires can be estimated by the integrals derived from **Supplementary Fig. 16a**, which are subsequently used to evaluate the strain of ZnO nano/microwires ($\varepsilon = \Delta l/l_0$). As demonstrated by the calculated curves of the nano/microwire strain as a function of the applied pressure in **Supplementary Fig. 16c**, the nano/microwire strain increases monotonously with the pressure increasing from 0 to 0.6 MPa, while the structure ratio around 0.75 may induce the relatively high strain level in the nano/microwire and thus a relatively excellent sensitivity. In order to further analyze the relationship between the microprotrusion structure ratio and the nano/microwire deformation, the nano/microwire strain as a function of the structure ratio under five different levels of pressure (0, 0.15, 0.30, 0.45, and 0.60 MPa) are simulated in a finer gradient of the structure ratio ranged from 0.25 to 1.25 and

summarized in **Supplementary Fig. 16d**. It can be found that the strain level of the nano/microwire can reach a maximum value at a structure ratio of about 0.65~0.70 under a given pressure, which is in good agreement to the previous simulation results with application of the downward displacement (d). Also, we can find that no matter how much the loading pressure is applied, the optimal microprotrusion both possesses the structure ratio of about 0.65~0.70.

Additionally, in order to explain why the results (the optimal microprotrusion possessing a structural ratio w/L_{NW} of about 0.65~0.70) obtained by the two simulation approaches by calculating the downward displacement and calculating the applied pressure are consistent with each other, we also studied the relationship between the displacement and the pressure. As shown in **Supplementary Fig. 17a**, the simulation results indicate that there is a good linear relationship between the downward displacement and the pressure applied on the top edge of the microprotrusion, as similar trends can be observable in DPTs with different microprotrusion structure ratios. It is this linearity that makes the two results obtained by the two simulation approaches consistent. As a consequence, it is an appropriate approach to quantitatively apply the vertical force on the DPT with the application of the downward displacement, considering that the displacement can be accurately controlled by a high-precision actuator in the experimental section.

Furtherly, as shown in **Supplementary Fig. 17b**, the downward displacement (d) increases with the structure ratios (w/L_{NW}) slightly, and then reaches a saturated value ($d = 0.92\ mm$) under a given pressure of 0.6 MPa applied on the microprotrusion. It is worth noting that the maximum downward displacement of the top edge of microprotrusion occurs when the structure ratios is about 1.00~1.10 rather than 0.65~0.70. Actually, in vertical force sensing mode of DPT, it is not that the larger the displacement is, the better, because the larger structural ratio will make the nano/microwire move down as a whole rather than produce strain. The above contents provide a better understand the structural characteristics of the microprotrusion in DPT.

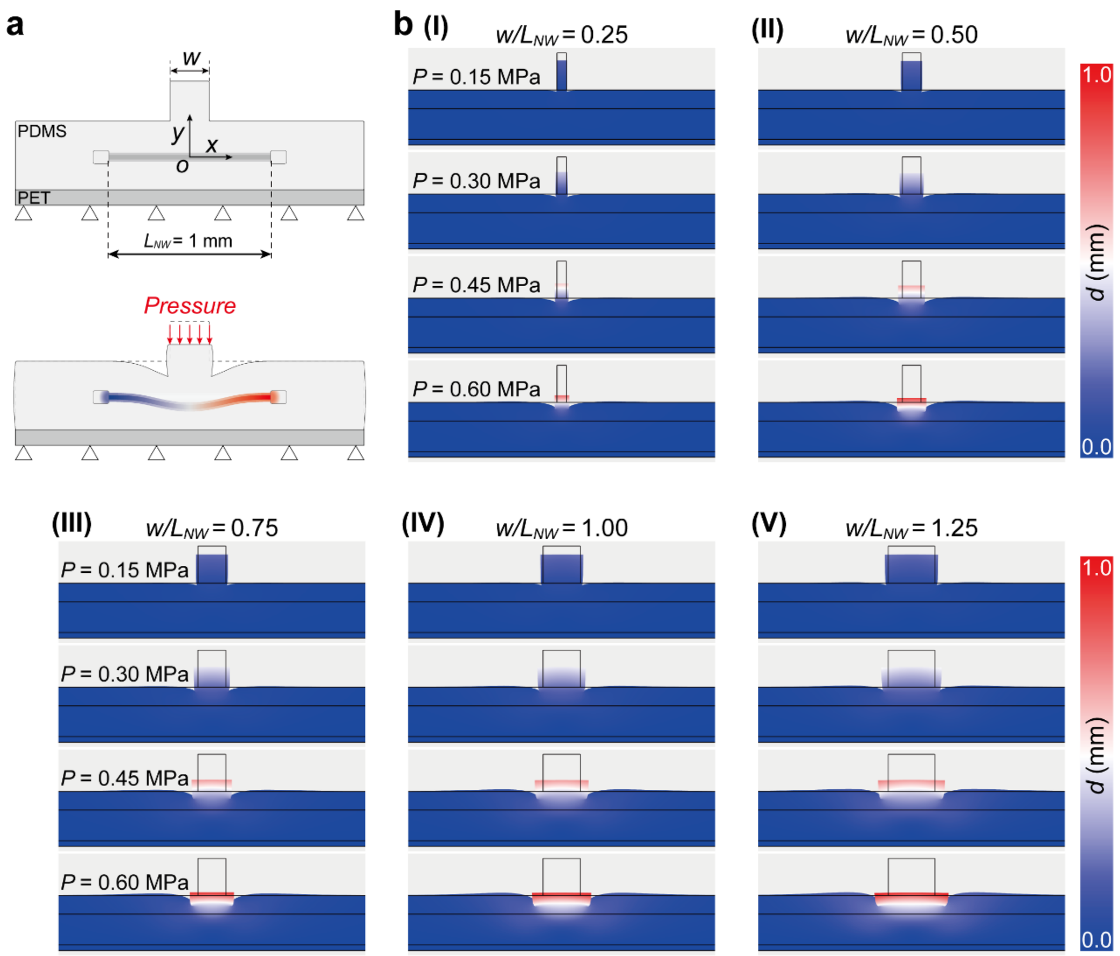

Supplementary Fig. 15 | Simulated shapes of DPTs with various structure ratios (w/L_{NW}) under different pressures. a, Schematic illustration of the side view at the *xy* plane of the DPT unloaded (top) and loaded (bottom) with pressure. L_{NW} and w represent the length of the ZnO nano/microwire and the width of the microprotrusion, respectively. **b**, Calculated displacement distribution profiles of the DPTs with varies of the microprotrusion structure ratios (w/L_{NW}) at different applied pressures. The loads are applied as the uniform pressure on the top edge from 0.15 MPa to 0.6 MPa and the structure ratios (w/L_{NW}) of the microprotrusion are set as 0.25~1.25. The solid line represents the original shape of DPTs before the pressure is applied. Colors represent the value of the downward deformation after compression.

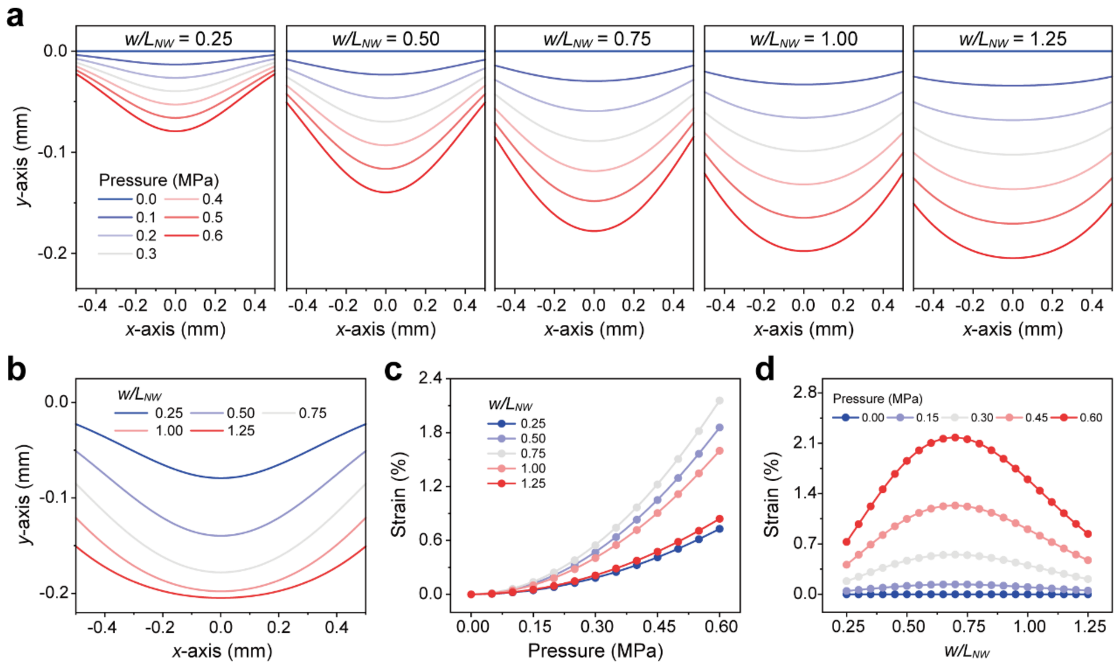

Supplementary Fig. 16 | Simulated results of the nano/microwire deformation in DPTs with various structure ratios (w/L_{NW}) under uniform pressures. a, Outlines of the bended nano/microwires in DPTs with various structure ratios (w/L_{NW}) from 0.25 to 1.25 in response to different applied pressures. **b**, The corresponding outlines of nano/microwires of the DPTs with various structure ratios (w/L_{NW}) at a constant pressure of 0.6 MPa. **c**, The calculated strain of the nano/microwires in DPTs with various structure ratios (w/L_{NW}) as a function of the pressure. **d**, The curves of nano/microwire strain versus structure ratio (w/L_{NW}) under different applied pressures.

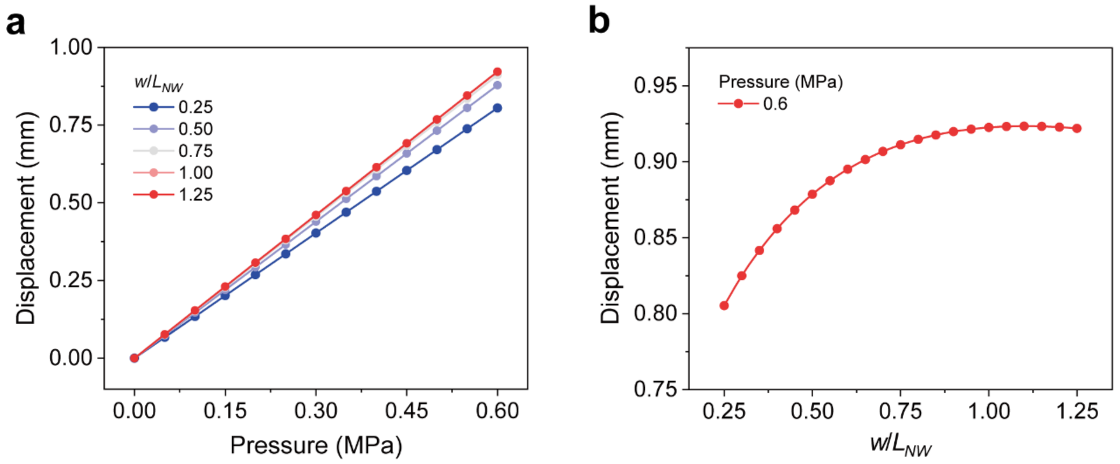

Supplementary Fig. 17 | The relationship between the downward displacement (d) of the top edge of microprotrusion and the applied pressure in DPTs with various structure ratios (w/L_{NW}) for vertical force sensing obtained from the FEM simulation. a, The corresponding downward displacement (d) of the top edge of the microprotrusion as a function of the applied pressure in DPTs with various structure ratios (w/L_{NW}). **b**, The curve of downward displacement (d) versus structure ratio (w/L_{NW}) under a constant pressure of 0.6 MPa.

(3) Influence of the material properties on the performance of the DPT

The impacts of the materials on DPT have been further studied. Considering the extremely small size of ZnO nano/microwire compared with the PDMS and PET, we focus on the influences of the PDMS and PET as follows.

① *Impact of the material properties of PDMS*

As the main material in the overall structure of DPT, the mechanical properties of polydimethylsiloxane (PDMS) are directly related to the nano/microwire deformation macroscopically. PDMS, one of the most widely used elastomer materials due to its excellent flexibility, optical transparency, chemical inertness, biocompatibility and simple fabrication, demonstrates promising applications in fields such as wearable electronics, soft robots, microelectromechanical systems (MEMS), biomedicine, microfluidics, and many others [35, 36]. Uniquely, the inherent mechanical property of PDMS, namely Young's modulus, can be further controllable through changing the

degree of PDMS network's cross-linking, which can be achieved, for instance, by modifying the ratio of prepolymer to curing agent, curing time and temperature [36, 37]. Conventionally, the main property of PDMS used in the FEM (finite element method) modeling is the Young's modulus (E), which are set to be 750 kPa [33].

Here we carefully utilize the FEM simulation delving into the possible influence of the material properties (such as Young's modulus) of PDMS on the performance of the DPT for vertical force sensing. According to previous reports, modifying the cross-linker concentration and the baking time will effectively modulate the Young's modulus of PDMS (Sylgard 184) in a large range from 50 kPa to about 4 MPa [38, 39]. Based on the experimental data from the relevant literatures, the following **Supplementary Table 3** gives a summary of the mechanical properties (Young's modulus E), which corresponded to a series of PDMS samples with the mixtures of Sylgard 184 base and curing agent in the wt. ratios of 5:1, 10:1, 15:1, 20:1, 25:1 and 30:1 [40].

Supplementary Table 3 | Summary of Young's moduli (E) of the PDMS samples with different base/agent ratios [40].

Sample, Base/agent ratio	Young's modulus, $\boldsymbol{E}$
PDMS, 5:1	3 MPa
PDMS, 10:1	2 MPa
PDMS, 15:1	1.5 MPa
PDMS, 20:1	0.8 MPa
PDMS, 25:1	0.45 MPa
PDMS, 30:1	0.15 MPa

Then, we fit the given parameters to the FEM model to simulate the mechanical deformation process of the DPTs in vertical force sensing mode. **Supplementary Fig. 18a** shows the simulated shapes of the DPTs with a constant microprotrusion structure ratios w/L_{NW} of 1.00 and PDMS possessing different Young's moduli (E, ranged from

0.15 MPa to 3 MPa) under different downward displacement (d) of the top edge of microprotrusion. As can be seen, there is almost no difference in the shape change of the DPTs under the same downward displacement. By summarizing the data in **Supplementary Fig. 18a**, we can obtain the relationship between the nano/microwire strain and the downward displacement in DPTs with different Young 's moduli of PDMS (**Supplementary Fig. 18b**), and the curves of the nano/microwire strain as a function of the Young 's modulus of PDMS under different downward displacement (**Supplementary Fig. 18c**). Note that under the same downward displacement (d), the strain level of the nano/microwire just exhibit a slight increase due to the Young's modulus variation of PDMS, as shown in **Supplementary Fig. 18b** and **Supplementary Fig. 18c**. It can be seen that using PDMS with different Young's moduli, we can approximately control the nano/microwire strain by controlling the downward displacement of the top edge of microprotrusion.

Also, the plots of the corresponding pressure applied on the DPT as a function of the displacement (d) are shown in **Supplementary Fig. 19**. With the Young's modulus of PDMS ranging from 0.15 MPa to 3 MPa, the range of loading pressure can be dynamically changed from the low-pressure range (0~72 kPa) to the high-pressure range (0~2.1 MPa) as shown in **Supplementary Fig. 19a**. A strong linearity can be found between the loading pressure and the displacement. Moreover, it can be seen clearly from **Supplementary Fig. 19b** that the material properties of PDMS have a notable effect on the pressure of the DPT, since the applied pressure linearly increases with the Young's modulus (E) under the same displacement (d). These findings suggest that we can effectively expand the pressure working range via tuning the Young's modulus of PDMS to optimize the sensing capability of DPT, which demonstrates its feasibility in broadening the application scenarios.

It should be noted that we use the microprotrusion here with a structural ratio of 1.00 to simulate as a demonstration, mainly considering the mechanical stability of the microprotrusion particularly when the PDMS Young 's modulus is relatively small (for example, 0.15 MPa); in fact, for DPTs with other structural ratios, the conclusions derived from the above discussions remain unchanged.

② *Impact of the material properties of PET*

Ploy(ethylene terephthalate) (PET) is also a key material for DPTs. We also investigate the influence of the material properties of PET on the sensing performance of the DPT. It is well-known that PET film is a common polymer material with good strength, flexibility, electrical insulation, and chemical stability, which is widely used in industrial applications such as photographic films, flexible electrical circuits, and packaging materials [32,41]. In the FEM simulation, the Young's modulus ($E = 4$ GPa) of PET reported in the previous paper is adopted [32].

Supplementary Fig. 20a-c show the displacement distribution profiles obtained from the FEM simulation under different downward displacements (d), which exhibit the deformation of the DPTs with PET substrate (**Supplementary Fig. 20a**), without substrate (**Supplementary Fig. 20b**) and with PDMS substrate (**Supplementary Fig. 20c**), respectively. As demonstrated by the corresponding outlines of nano/microwires in **Supplementary Fig. 20d** and **Supplementary Fig. 20e**, the existence of PET substrate has a negligible effect on the nano/microwire deformation in vertical force sensing mode, since the nano/microwire strain will remain essentially unchanged in the DPTs with and without PET substrate (**Supplementary Fig. 20g** and **Supplementary Fig. 20h**). This mainly results from the fact that the deformation of PET substrate is almost imperceptible under the application of vertical force and the quite high hardness of PET ($E = 4$ GPa). Hence, the material properties of PET substrate should not contribute to the sensing performance of the DPT. In contrast, upon application of the substrate material that much softer than PET (i.e., PDMS), the increase in the thickness of bottom PDMS layer would induce a larger bending deformation and thus yield a larger strain level of the nano/microwire in the DPT (**Supplementary Fig. 20f** and **Supplementary Fig. 20i**). The maximum strain of the nano/microwire in the DPT with PDMS substrate can reach 1.36%, whereas that of the nano/microwire with PET substrate is about 1.21% under the same displacement of 0.8 mm, as shown in **Supplementary Fig. 20g** and **Supplementary Fig. 20i**.

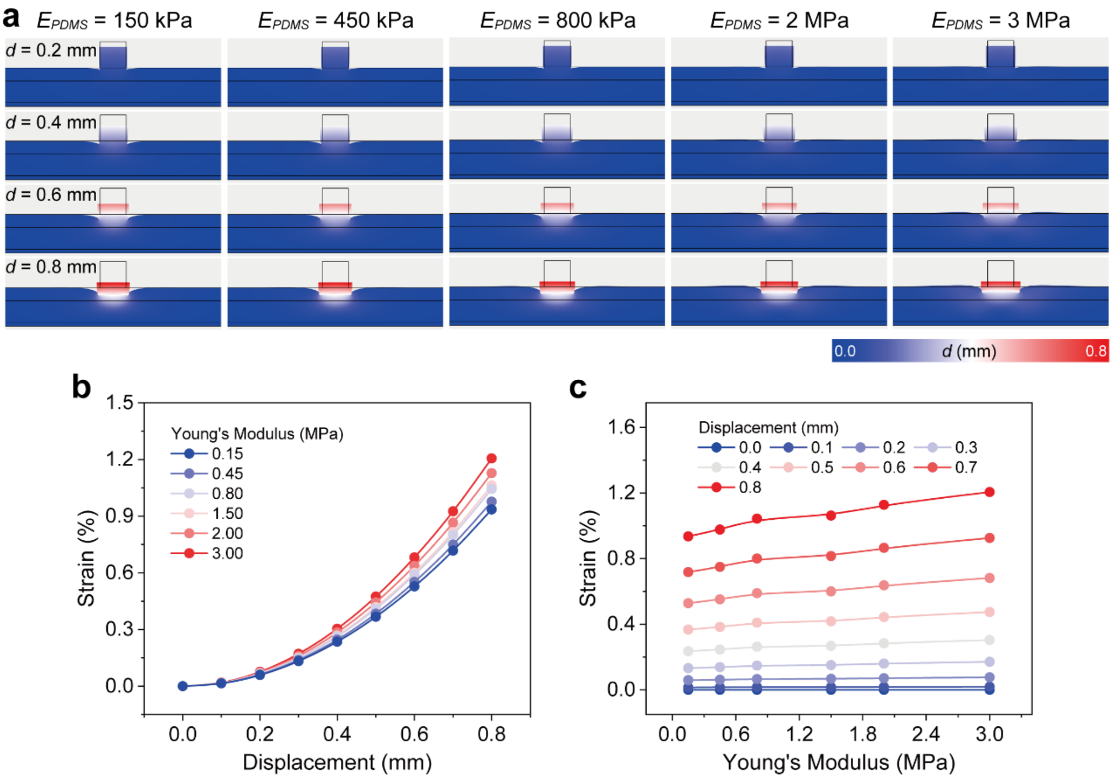

Supplementary Fig. 18 | Impact of the material properties of PDMS on the performance of the DPT in vertical force sensing mode. a, Simulated shapes of DPTs fabricated by PDMS with various Young's moduli (E) under different downward displacement (d). The microprotrusion structure ratio in the simulations is 1.00. **b**, Calculated strain of nano/microwires in the DPTs with various Young's moduli of PDMS as a function of the downward displacement (d). **c**, The curves of nano/microwire strain versus Young's modulus (E) under different downward displacements (d).

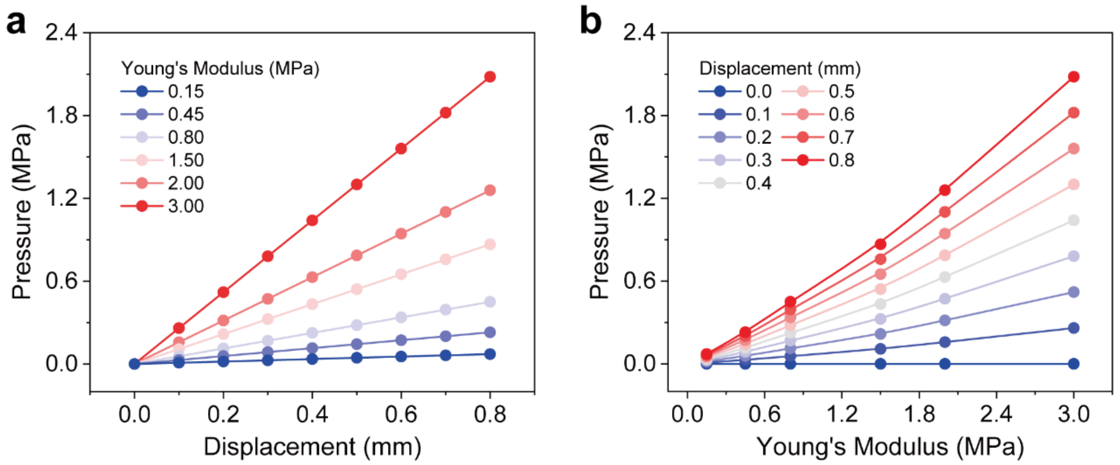

Supplementary Fig. 19 | Impact of the material properties of PDMS on the relationship between the pressure and the downward displacement. a, Pressure applied on the top edge of the microprotrusion as a function of the downward displacement (d) in the DPTs fabricated by PDMS with various Young's moduli (E). **b**, The relationship between the applied pressure and the Young's modulus of PDMS in response to different downward displacements.

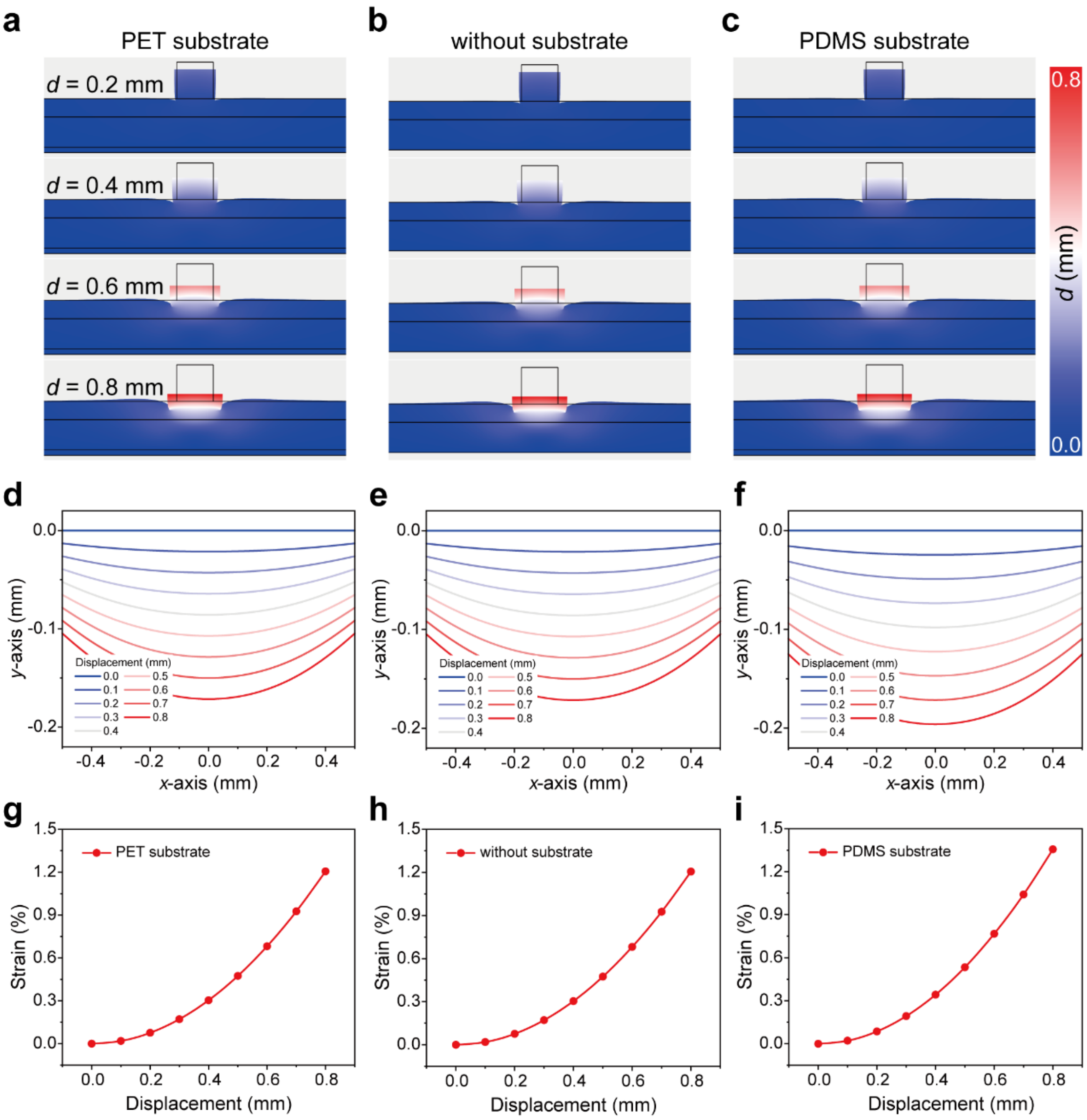

Supplementary Fig. 20 | Impact of the PET substrate on the performance of the DPT in vertical force sensing mode. a-c, Displacement distribution profiles of the DPTs with PET substrate (**a**), without PET substrate (**b**) and with PDMS substrate (**c**) in response to different displacements (*d*) obtained from the FEM simulations. **d-f**, The corresponding outlines of bended nano/microwires in the DPTs with PET substrate (**d**), without PET substrate (**e**) and with PDMS substrate (**f**) under different displacements. **g-i**, The calculated nano/microwire strain as a function of displacement in the DPTs with PET substrate (**g**), without PET substrate (**h**) and with PDMS substrate (**i**), respectively.

(4) Influence of the PDMS thickness on the performance of the DPT

According to the structural design of the DPT, the thickness of PDMS layer will directly affect the sensitivity to the vertical stimulus on the bending deformation of the nano/microwire, which also plays a crucial role in improving the sensing performance. The additional PDMS layer is introduced before the transfer of ZnO nano/microwire to protect the lateral nano/microwire from breaking when subjected to the vertical force. Therefore, its main function is to provide a soft substrate as buffer layer for the downward bending of nano/microwires in the vertical force sensing mode. Another additional function is to produce greater strain on the nano/microwire when the substrate bends. In order to delve into the PDMS thickness effect in the DPT for vertical force sensing, the FEM simulations have been performed with a series of controlled experiments.

① *Influence of the bottom PDMS thickness on the vertical force sensing performance*

To investigate the influence of the thicknesses of PDMS layers on the sensing performance of the DPT, the finite element method (FEM) simulation is established to analyze the structure of the DPT in vertical force sensing mode. **Supplementary Fig. 21a** schematically shows the side view (xy plane) of the DPT with two soft PDMS layers, in which $h_{PDMS,bottom}$ and $h_{PDMS,top}$ represent the thicknesses of the bottom PDMS layer (as substrate) and the top PDMS layer (as encapsulation), respectively. Meanwhile, other structural parameters are set as constant values, such as the microprotrusion (1 mm in width and 1 mm in height), the ZnO microwire (1 mm in length) and the PET substrate (0.15 mm in thickness and 30 mm in length).

With a constant thickness of the top PDMS layer ($h_{PDMS,top} = 0.5\ mm$), the displacement distribution profiles of the DPTs and the corresponding outlines of bended nano/microwires in response to different displacements (d) applied on the DPTs with various thicknesses of the bottom PDMS layers ($h_{PDMS,bottom}$) ranged from 0.425 mm to 1.625 mm are demonstrated in **Supplementary Fig. 21b** and **Supplementary Fig. 21c**, respectively. It can be obviously observed in **Supplementary Fig. 21c** that the magnitude of the overall displacement of the nano/microwire in the vertical direction (the y-axis) gradually increases with thicker bottom PDMS. Also, it can be found that

the downward bending degree of the nano/microwire increases as the bottom PDMS thickness increases. As expected, the tendency of decrease in the bending deformation and the overall displacement of the nano/microwire with respect to the reduced thickness of the bottom PDMS layer should be attributed to the enhanced substrate clamping effect in the thinner PDMS layer. The obtained curves of nano/microwire strain versus downward displacement (d) with the thickness in the range of 0.225 mm to 1.625 mm are plotted in **Supplementary Fig. 21d**, which also indicates that the strain level of the nano/microwire increases with the bottom PDMS thickness. Under the given displacement ($d = 0.8\ mm$), the nano/microwire strain increases approximately linearly and then tends to a saturated value (~1.6%) when the thickness of the bottom PDMS layer exceeds 1.225 mm, as shown in **Supplementary Fig. 21e**. It is worth noting that although the strain increases with the increase of the bottom PDMS thickness, the thickness should not be too large considering the practical application of the device.

② *Influence of the top PDMS thickness on the vertical force sensing performance*

The influence of the top PDMS layer on the vertical force sensing performance is also studied by using the FEM model of the DPT with a constant thickness of the bottom PDMS layer ($h_{PDMS,bottom} = 0.825\ mm$), while the thickness of the top PDMS layer ($h_{PDMS,top}$) is variable from 0.15 mm to 1 mm (**Supplementary Fig. 22**). **Supplementary Fig. 22b** shows the displacement distribution profiles obtained under different downward displacements (d), which indicates the device deformation with the top PDMS layers at the thicknesses ($h_{PDMS,top}$) of 0.15 mm, 0.50 mm and 1.00 mm, respectively. It is worth noting that a thinner encapsulation layer (i.e., the top PDMS layer) leads to a diminution of the distance between the nano/microwire and the microprotrusion, and hence induces a larger bending deformation of the microwire under vertical force condition, as shown in **Supplementary Fig. 22c**.

Supplementary Fig. 22d gives the calculated strain of the bended nano/microwire as a function of the downward displacement (d) in the DPTs with different thicknesses of the top PDMS layers ($h_{PDMS,top}$). It can be seen clearly from **Supplementary Fig.**

22e that when a 0.15-mm-thick PDMS layer is employed for encapsulation, the maximum strain level of the microwire under the downward displacement d of 0.8 mm can reach about 3.0%, which then begins to drop dominantly with increasing thickness of the top PDMS layer. On the other hand, the top PDMS layer is introduced as encapsulation for insulation and protection, which should achieve a robust thickness in order to maintain the physical stability of the ZnO nano/microwire. Therefore, utilizing the PDMS layer with appropriate thickness can effectively control the force transfer process to induce the sufficiently large deformation in the nano/microwire, while maintaining high sensitivity as well as excellent reliability of the DPT for vertical force sensing.

③ *Influence of the top/bottom PDMS layer thickness ratio on the vertical force sensing performance*

The FEM model of the DPT with various thicknesses of the top PDMS layers ($h_{PDMS,top}$) ranging from 0.1 mm to 1.0 mm are constructed (**Supplementary Fig. 23**), while keeping the total PDMS thickness fixed ($h_{PDMS} = 1.325\ mm$). With application of the displacement boundary conditions (d) on the top edge of the microprotrusion, the side views of the DPTs in the initial state and the vertical force sensing mode are schematically shown in **Supplementary Fig. 23a**. As demonstrated by the FEM simulation results in **Supplementary Fig. 23b**, it is evident that the PDMS thickness ratio of the top layer to the total ($h_{PDMS,top}/h_{PDMS}$) would not affect the overall mechanical property and the corresponding displacement distribution of the DPT, which could be attributed to the identical thickness of the total PDMS layer. Nevertheless, due to the influence of the position of the nano/microwire, the DPTs with various thickness ratios of the PDMS layers ($h_{PDMS,top}/h_{PDMS}$) will induce different magnitudes of bending deformation in the nano/microwires, which continuously decline with the increase of thickness ratio ($h_{PDMS,top}/h_{PDMS}$), as shown in **Supplementary Fig. 23c**. It can be also seen clearly from the calculated strain curves of the nano/microwires in **Supplementary Fig. 23d** and **Supplementary Fig. 23e** that, under the application of a certain displacement ($d = 0.8\ mm$), the nano/microwire

strain will decrease from 3.63% at the thickness ratio of $h_{PDMS,top}/h_{PDMS} = 0.08$ down to only 0.05% at the ratio of $h_{PDMS,top}/h_{PDMS} = 0.75$, which is drastically reduced by ~98.5%. Therefore, it is expected to induce much higher strain level in the nano/microwire with the relatively small thickness ratio ($h_{PDMS,top}/h_{PDMS}$), when the position of the nano/microwire is closer to the local region surrounding the deformed microprotrusion (**Supplementary Fig. 23b**).

In summary, the introduction of the bottom PDMS layer is mainly to meet the needs of vertical force sensing of DPT, which can improve the nano/microwire strain caused by external force or strain to a certain extent; the introduction of the top PDMS is mainly to encapsulate and protect the nano/microwire, with the best thickness to improve the vertical force sensing performance of DPT. Apart from above effect of the PDMS layer on the device performance, it is found that the lack of top and bottom PDMS layers will cause the local stress of the device to be too large and very easy to damage through a large number of experiments. Therefore, a very important function of the top and bottom PDMS layers together is actually to protect the lateral nano/microwire from breaking when subjected to the vertical force.

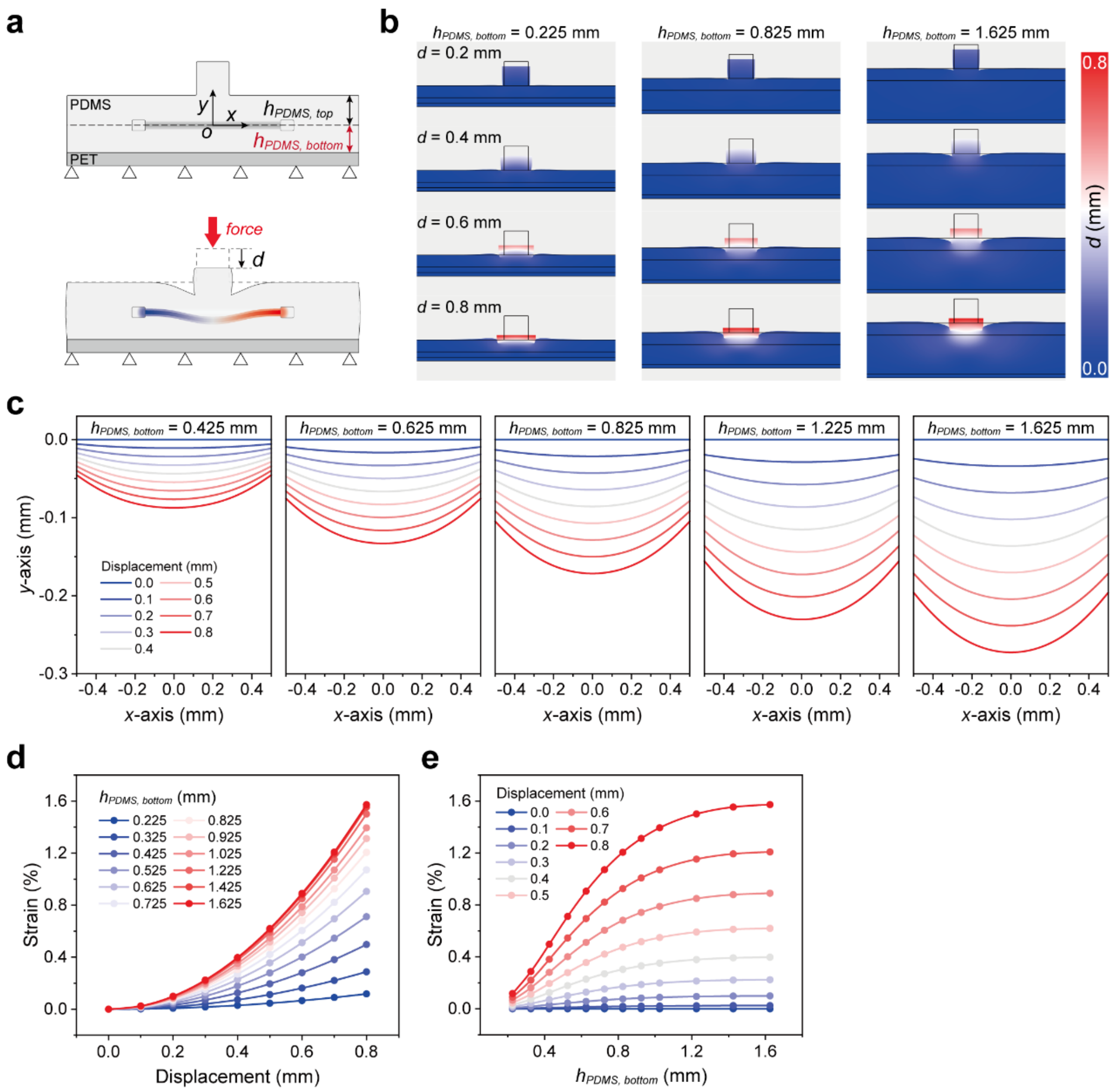

Supplementary Fig. 21 | Influence of the thickness of bottom PDMS layer ($h_{PDMS,bottom}$) on the performance of the DPT for vertical force sensing by FEM simulations. a, Schematic illustration of the DPT with the detailed structure of two PDMS layers in vertical force sensing mode. $h_{PDMS,top}$ and $h_{PDMS,bottom}$ represent the thicknesses of the top PDMS layer and the bottom PDMS layer, respectively. **b** and **c**, Displacement distribution profiles of the DPTs (**b**) and the corresponding outlines of bended microwires (**c**) with various thicknesses of bottom PDMS layers ($h_{PDMS,bottom}$) in response to different downward displacements (d). **d**, The calculated strain of nano/microwires of the DPTs with different thicknesses of bottom PDMS layers ($h_{PDMS,bottom}$) as a function of displacement (d). **e**, The curves of microwire strain versus thickness of bottom PDMS layer ($h_{PDMS,bottom}$) under different displacements (d).

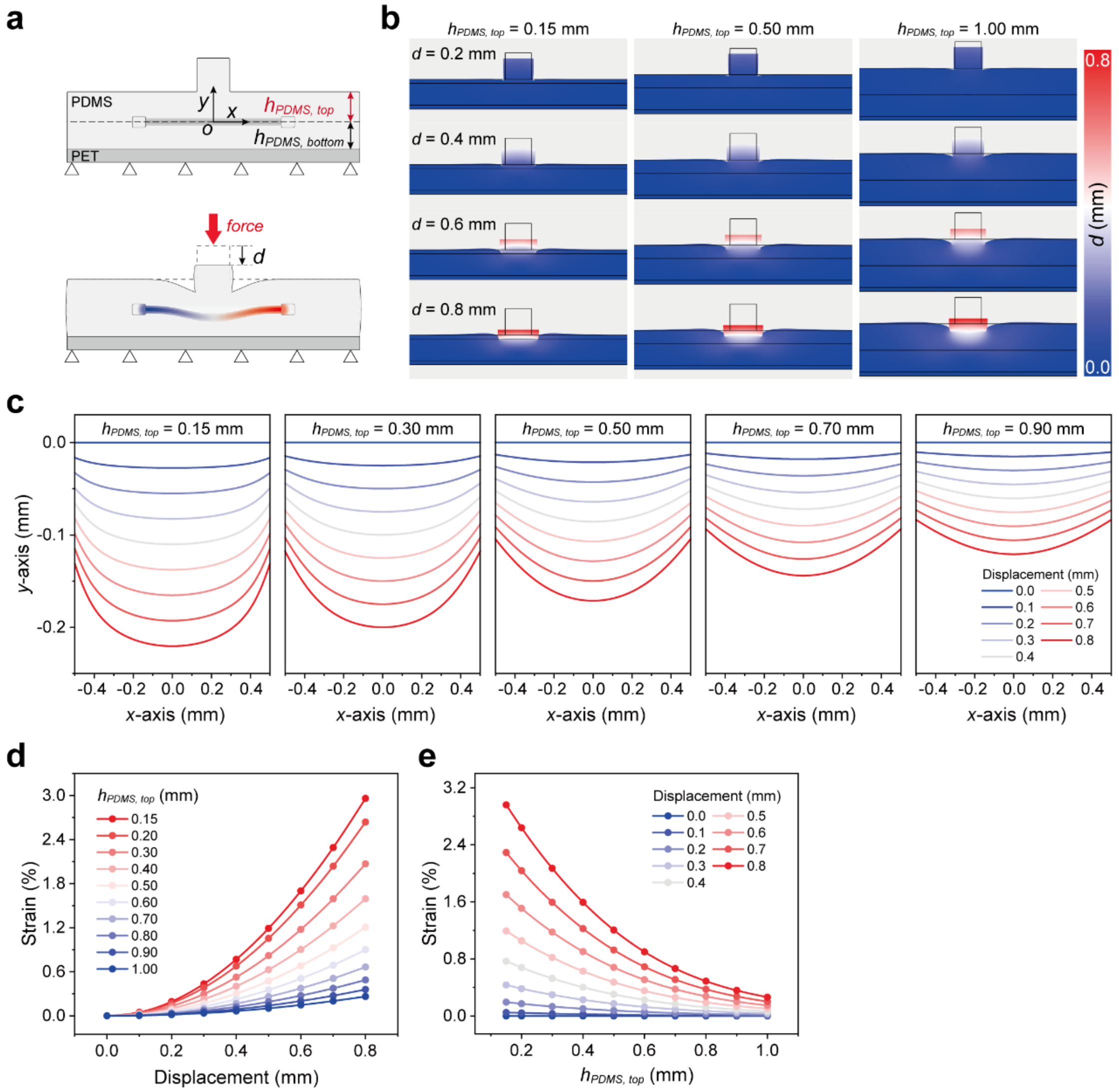

Supplementary Fig. 22 | Impact of the thickness of top PDMS layer ($h_{PDMS,top}$) on the performance of the DPT for vertical force sensing by FEM simulations. a, Schematic illustration of the DPT with the detailed structure of two PDMS layers in vertical force sensing mode. $h_{PDMS,top}$ and $h_{PDMS,bottom}$ represent the thicknesses of the top PDMS layer and the bottom PDMS layer, respectively. **b** and **c**, Displacement distribution profiles of the DPTs (**b**) and the corresponding outlines of bended microwires (**c**) with various thicknesses of top PDMS layers ($h_{PDMS,top}$) in response to different downward displacements (d). **d**, The calculated strain of microwires of the DPTs with different thicknesses of top PDMS layers ($h_{PDMS,top}$) as a function of displacement (d). **e**, The curves of microwire strain versus thickness of top PDMS layer ($h_{PDMS,top}$) under different displacements (d).

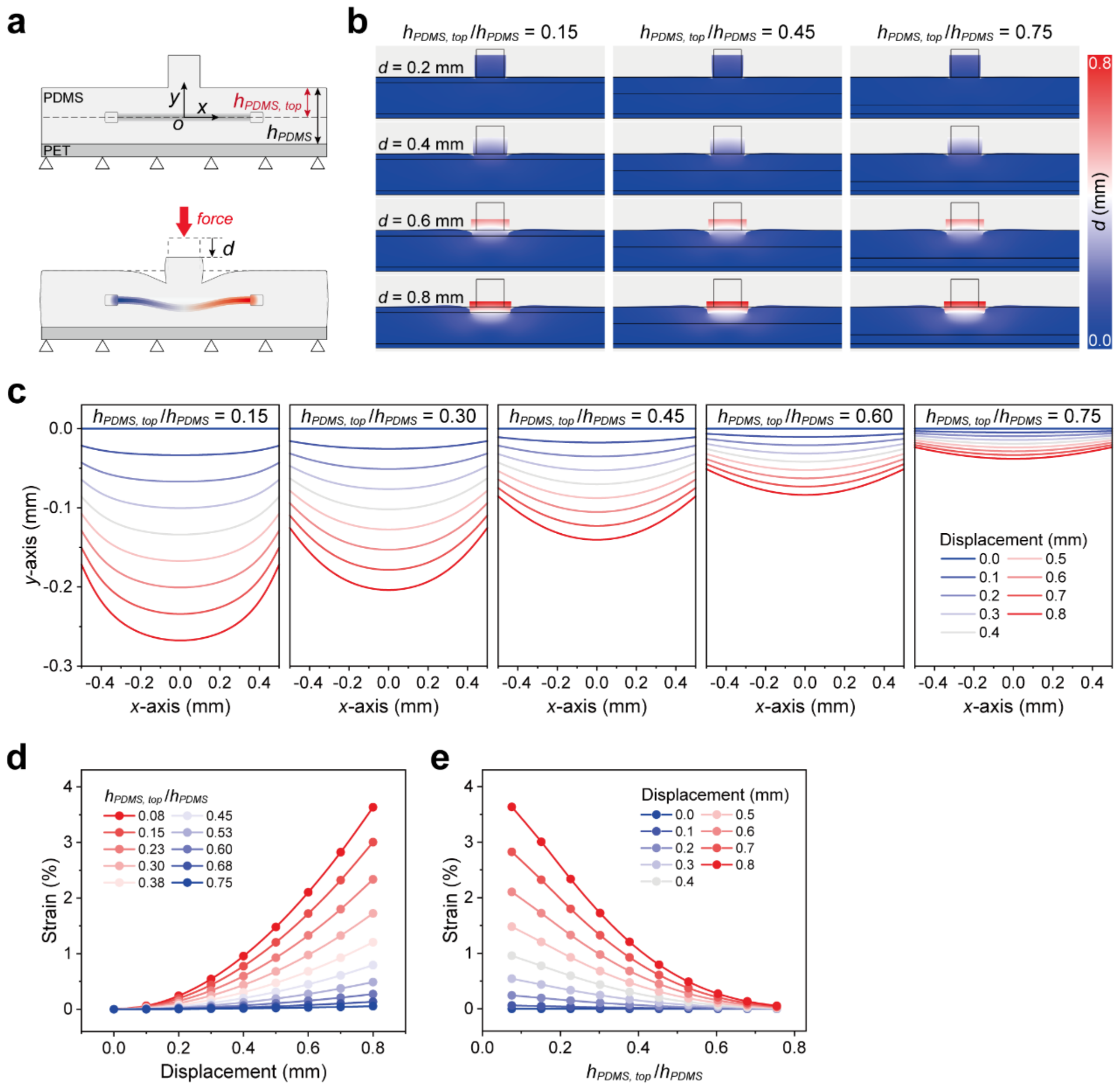

Supplementary Fig. 23 | Impact of the PDMS thickness ratio of the top layer to the total ($h_{PDMS,top}/h_{PDMS}$) on the DPT performance for vertical force sensing by FEM simulations. a, Schematic illustration of the DPT with the detailed structure of two PDMS layers in vertical force sensing mode. $h_{PDMS,top}$ and h_{PDMS} represent the thicknesses of the top PDMS layer and the total PDMS layer, respectively. **b** and **c**, Displacement distribution profiles of the DPTs (**b**) and the corresponding outlines of bended nano/microwires (**c**) with various thickness ratios of the PDMS layers ($h_{PDMS,top}/h_{PDMS}$) in response to different displacements (d). **d**, The calculated strain of nano/microwires of the DPTs with different PDMS thickness ratios ($h_{PDMS,top}/h_{PDMS}$) as a function of displacement (d). **e**, The curves of nano/microwire strain versus PDMS thickness ratio ($h_{PDMS,top}/h_{PDMS}$) under different displacements (d).

(5) Influence of the height of the PDMS microprotrusion on the performance of the DPT

① *Influence of the PDMS microprotrusion height on the vertical force sensing performance*

Considering the extremely small diameter of the ZnO nano/microwire (usually in the micron scale, such as about 6.5 μm) compared with the length of the PDMS microprotrusion (millimeter scale), the influence of the microprotrusion length on the microwire deformation under vertical force condition is negligible in this work. However, the height (also can be called thickness) of the PDMS microprotrusion ($h_{protrusion}$) actually has an important impact on the performance of the DPT for vertical force sensing. Here, the microprotrusions with various heights ranging from 0.2 mm to 2.0 mm are introduced in the FEM models of the DPTs while keeping other structural parameters unchanged. We model the microprotrusion with width $w = 1\ mm$, and the structure ratio remains constant ($w/L_{NW} = 1$). Besides, the thicknesses of the PET substrate, the top and bottom PDMS layers are set as $h_{PET} = 0.15\ mm$, $h_{PDMS,top} = 0.5\ mm$ and $h_{PDMS,bottom} = 0.825\ mm$, respectively.

For the DPT in vertical force sensing mode, the applied force can be controlled by changing the downward displacement (d) on the top edge of the microprotrusion. Note that the DPT with higher microprotrusion could allow it to adapt to much larger downward displacement (d), whereas the displacements (d) are relatively limited for those with smaller microprotrusion heights ($h_{protrusion}$). So, we define the ratio between the downward displacement (d) and the microprotrusion height ($h_{protrusion}$) as a new parameter named downward displacement level ($d/h_{protrusion}$) in the loading process for the DPT with different microprotrusion heights. As demonstrated by the FEM analysis results in **Supplementary Fig. 24**, the DPT with the microprotrusion height ($h_{protrusion}$) of 0.25 mm will exhibit extremely small bending deformation of the nano/microwire with application of a low displacement range ($d = 0{\sim}0.2\ mm$). When the microprotrusion height ($h_{protrusion}$) rises up to 2.0 mm, larger range of the downward displacement ($d = 0{\sim}1.6\ mm$) will lead to an obvious increase in the overall displacement of the nano/microwire and then induce a larger bending

deformation (**Supplementary Fig. 24b**).

Supplementary Fig. 25a shows the typical strain curves of the nano/microwires in the DPTs with varying microprotrusion heights ($h_{protrusion}$) from 0.25 mm to 2.0 mm, which indicates that the nano/microwire strain shows a conspicuous monotonic increase as a function of the displacement level ($d/h_{protrusion}$). It can be seen from **Supplementary Fig. 25b** that, when the height of the microprotrusion is relatively small ($h_{protrusion} \leq 1.0\ mm$), higher microprotrusion could induce larger bending deformation in the nano/microwire, corresponding to a rapidly increased strain level. However, as the microprotrusion height further increases, a substantial amount of the downward displacement (d) would be inevitably dissipated to compress the microprotrusion with the additional height, which causes the strain growth of the bended nano/microwire to slow down gradually (**Supplementary Fig. 25b**).

Moreover, to evaluate the mechanical properties of the DPTs with different microprotrusion heights ($h_{protrusion}$), we systematically investigate the corresponding pressure applied on the DPTs under different levels of displacements (d). It is worth noting that the DPT exhibits a good linear relationship between the applied pressure and the displacement level ($d/h_{protrusion}$) for all cases, while the maximum measurable pressure rises rapidly at first and the trend is subsequently moderated with the increase of the microprotrusion height, as shown in **Supplementary Fig. 25c-d**. On the other hand, when a relatively small pressure (0.2 MPa) is applied, the strain level of the nano/microwire will decrease drastically due to a slight increase in the microprotrusion height, falling below 0.18% from 0.22% (**Supplementary Fig. 25e**). It can be obviously observed in **Supplementary Fig. 25f** that the tendency of decrease in the bending deformation of the microwire should be attributed to the decline of the induced displacement level with increasing microprotrusion height under a pressure of 0.2 MPa, indicating the small pressure is insufficient to drive the microprotrusion with a large height to bend the nano/microwire.

Therefore, these findings suggest the increase of the microprotrusion height ($h_{protrusion}$) could achieve a larger pressure response range but a much lower sensitivity with the minimum detectable pressure.

② *Influence of the PDMS microprotrusion structure on the lateral strain sensing performance*

For the traditional piezotronic transistor with the lateral structure, the induced strain in the ZnO nano/microwire can be directly sensed through the bending of the flexible substrate. In fact, considering that the ratio of the microprotrusion area to the substrate one is negligible, the microprotrusion in the DPT should not be necessary factors that affect the overall deformation of the device in lateral strain sensing mode. To evaluate the influence of the introduction of the microprotrusion structure on the mechanical property and the sensing performance, further simulations are carefully performed to qualitatively verify the sensitivity of the DPT in response to the lateral strain.

The typical 2D finite element models are constructed for the simulations with the consideration of the microprotrusion structure. As the demonstration of the loading process in the DPT for lateral strain sensing, we create the proper boundary condition to the neutral layer in the PET substrate to induce the bending deformation. Here we introduce an assumption that the length of the substrate (L) remains unchanged while the distance between the two ends of the substrate ($L - \Delta L$) decreases, leading to various curvature of the compressible PDMS layers in the model (**Supplementary Fig. 26a**). It can be quantitatively observed that the upper part of the PDMS layer at the center position could be gradually compressed as the increasing curvature of the substrate, as demonstrated by the strain distribution (ε_{11}) profiles under different bending conditions in **Supplementary Fig. 26b** and **Supplementary Fig. 26c** for the DPTs with and without the microprotrusion, respectively. Note that in comparison to the piezotronic transistor without the microprotrusion (**Supplementary Fig. 26e**), the existence of the microprotrusion in the DPT would create the localized high magnitude strain around the surface of the PDMS layer, which is more inclined to induce the inhomogeneous deformation of the nano/microwire under bending, as shown in **Supplementary Fig. 26d**. Subsequently, the calculated strain curves of the nano/microwires corresponding to the DPTs with and without the microprotrusion under the same loading conditions are obtained in **Supplementary Fig. 26f**. It can be found that when subjected to the bending deformation at the same curvature, the

nano/microwire strain for the DPT with the microprotrusion may slightly increase in the simulated strain range (0%~4%) compared to that without the microprotrusion, which should benefit from the stress concentration behavior around the microprotrusion. The above results validate that, as expected, the addition of the microprotrusion would not significantly affect the sensitivity of the DPT to the lateral strain.

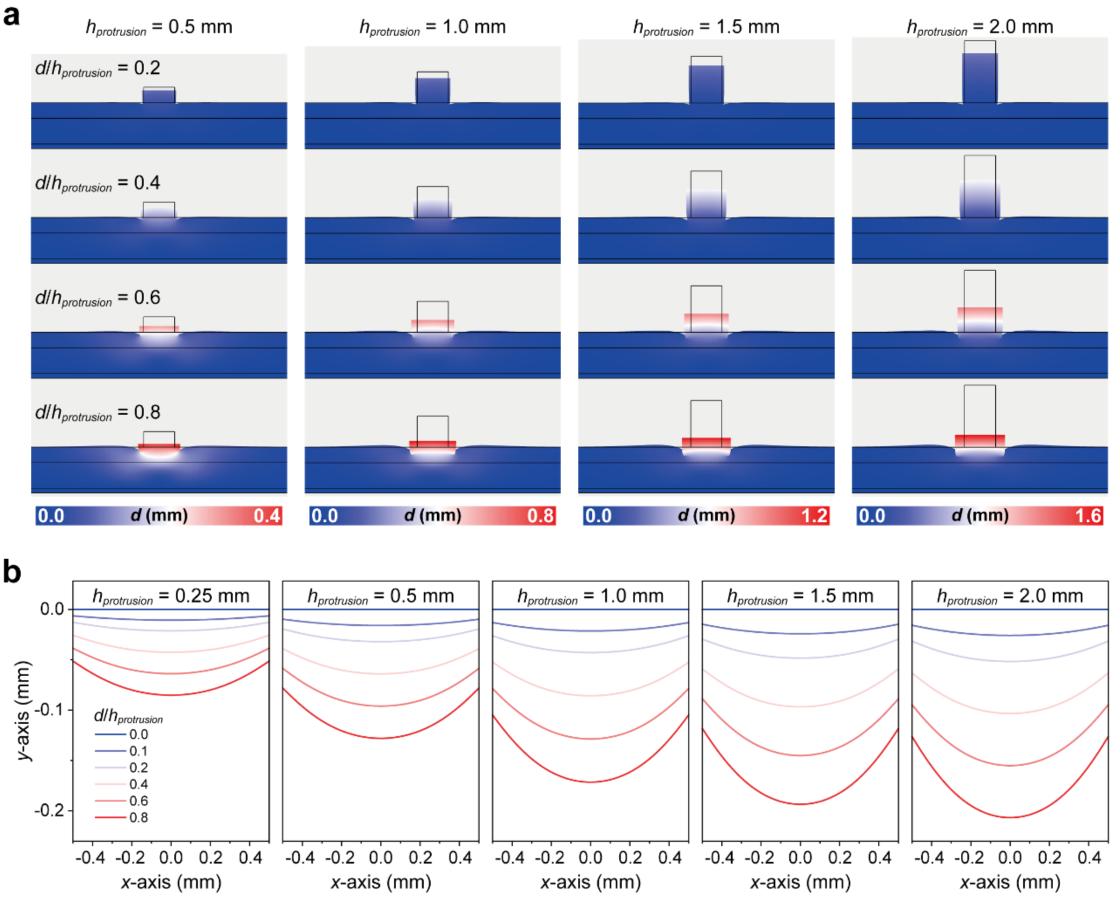

Supplementary Fig. 24 | The FEM simulation results for the DPTs with different microprotrusion heights ($h_{protrusion}$) in vertical force sensing mode. a and b, Displacement distribution profiles of the DPTs (**a**) and the corresponding outlines of bended nano/microwires (**b**) with various heights of the microprotrusions ($h_{protrusion}$) ranging from 0.25 mm to 2.00 mm under different downward displacement levels ($d/h_{protrusion}$ = 0~0.8).

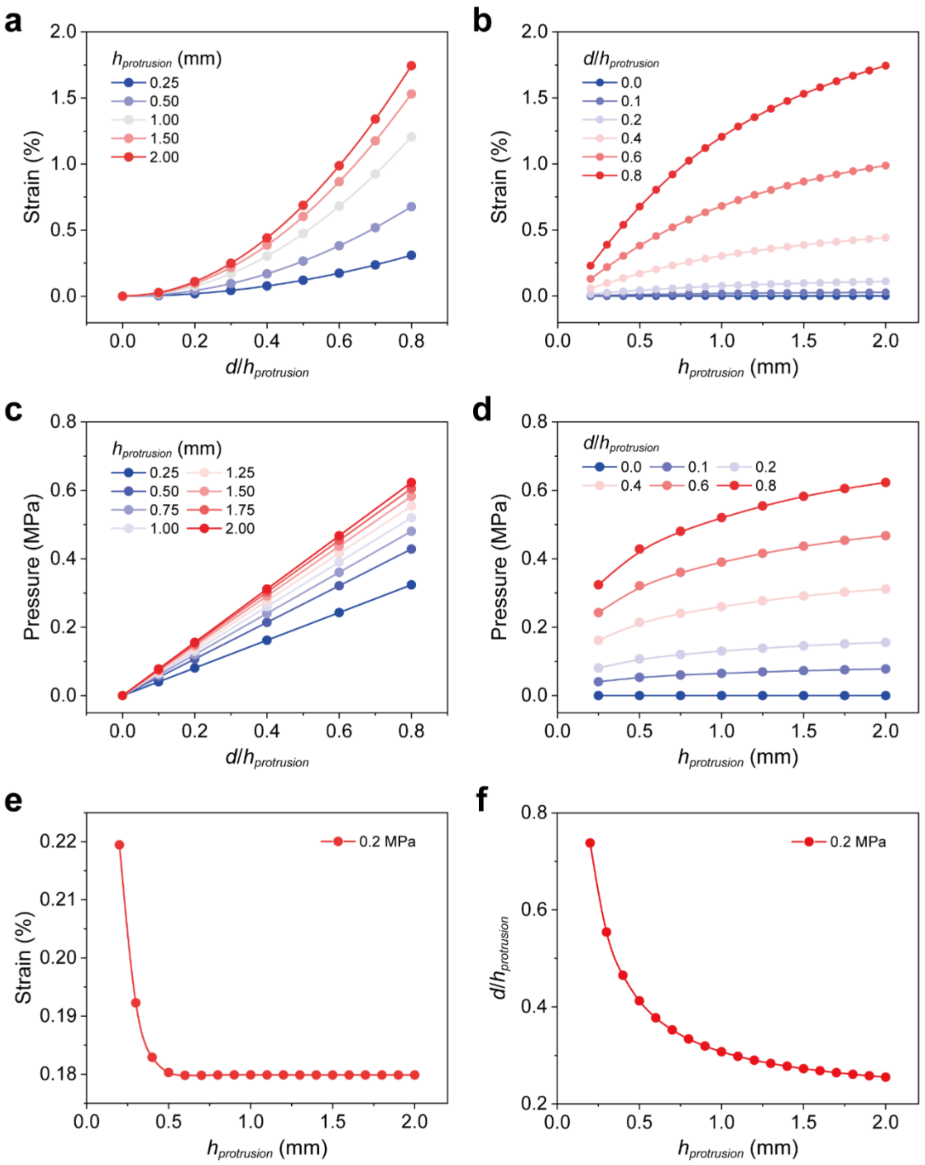

Supplementary Fig. 25 | Impact of the microprotrusion height on the DPT performance for vertical force sensing by the FEM simulation. a, Curves of nano/microwire strain versus the displacement level ($d/h_{protrusion}$) for DPTs with various microprotrusion heights ($h_{protrusion}$). **b**, Dependence of the simulated strain with respect to the microprotrusion height under different displacement levels. **c**, Corresponding pressure applied on the top edge of the microprotrusion as a function of the displacement level with different microprotrusion heights. **d**, The relationship between the applied pressure and the microprotrusion height in response to different displacement levels. **e** and **f**, Dependence of the calculated strain (**e**) and the corresponding displacement level (**f**) with respect to the microprotrusion height under a pressure of 0.2 MPa.

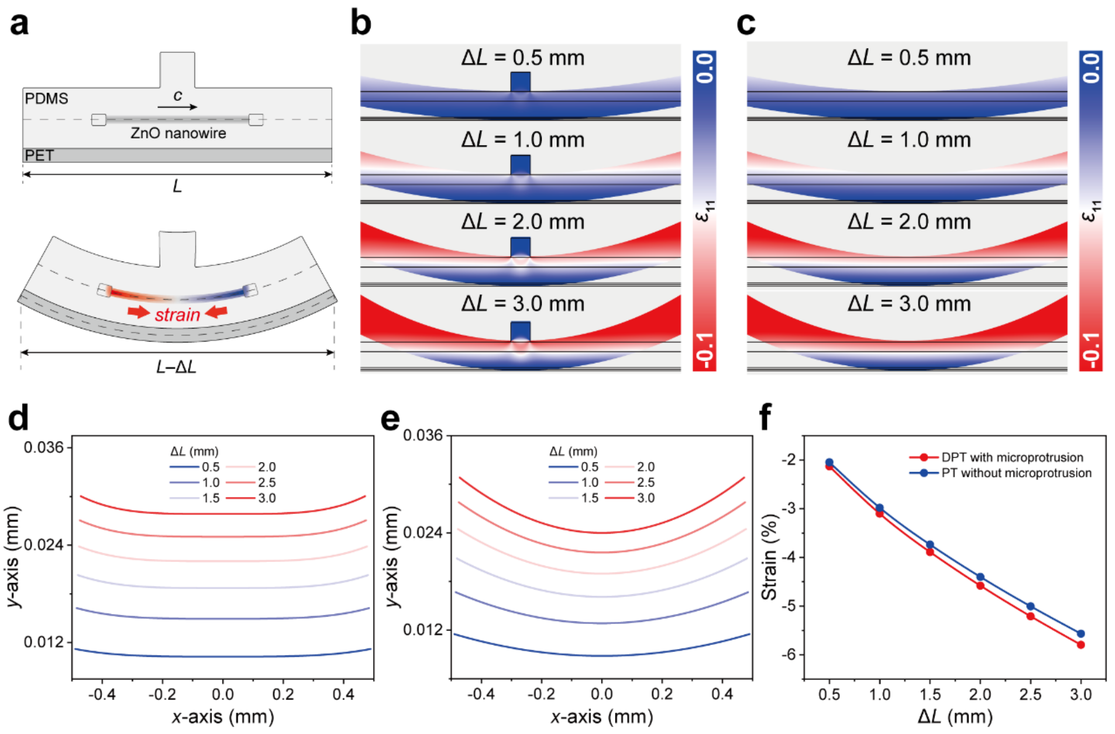

Supplementary Fig. 26 | Impact of the structure of the microprotrusion on the performance of the DPT for lateral strain sensing by the FEM simulation. a, Schematic diagrams of the side view of the DPT in lateral strain sensing mode. The original length of the substrate is L; the distance change between the two ends of the bending substrate is ΔL. **b** and **c**, Strain distribution (ε_{11}) profiles under bending of the DPT with (**b**) and without (**c**) the microprotrusion in response to different distance changes ΔL. **d** and **e**, Corresponding outlines of bended nano/microwires in the case with (**d**) and without (**e**) the microprotrusion under bending, respectively. **f**, Calculated nano/microwire strain as a function of the distance change (ΔL) applied under lateral strain condition for the DPT with and without the microprotrusion.

Supplementary Note 9 | Calculation of the strain in simulations

In order to estimate the bending deformation of ZnO nano/microwire in response to the applied force, we establish a 2D finite element model (FEM) of the DPT under plane stress condition, and assume that a nano/microwire with original length $L_{NW} = 1\ mm$ is embedded in the interface between two PDMS layers. According to the FEM simulation result, we can obtain the information relating to the mechanical deformation of the DPT under vertical force condition, such as the outline curve of bended nano/microwire in the displacement distribution profile. For the convenience of calculation, we will not consider the negligible effect of the diameter of the ZnO nano/microwire in the FEM simulations.

With application of the definite integral, if a smooth curve with equation $y = f(x),\ a \le x \le b$, is traversed exactly once as x increases from a to b, then its length l can be determined from the following formula:

$$l = \int_a^b \sqrt{1 + \left(\frac{dy}{dx}\right)^2}\, dx$$

(17)

Considering the very small size of the nano/microwire compared to PDMS and PET, we can estimate the change of the nano/microwire length (Δl) based on the integral for the curve length derived from the outline of bended nano/microwire. So, the axial strain of the nano/microwire along length direction can be calculated as

$$\varepsilon = \frac{\Delta l}{l_0} = \frac{l - l_0}{l_0}$$

(18)

where l_0 and l represent the original length of the nano/microwire in the initial state ($l_0 = 1\ mm$) and the calculated length of the nano/microwire under bending, respectively.

Supplementary Note 10 | Method to realize optimal microprotrusion structure ratio of 0.66

The dimensions of the DPT device are shown in **Supplementary Fig. 27a**, where the microprotrusion is manufactured by casting a PDMS pillar with a cross-sectional area S of 1×1 mm^2. In order to achieve the microprotrusion structure ratio w/L_{NW} of 0.66, we firstly matched nano/microwires by manufacturing microprotrusions with different sizes. However, since the microprotrusion is made of PDMS and its size is small (in about 1 millimeter), the error introduced by manufacturing microprotrusions was relatively large. Later, in order to solve this problem, we found that by controlling the size of the mask to accurately control the electrode spacing (distance between source and drain electrodes, deposited by RF-magnetron sputtering on the ZnO nano/microwire) is a more feasible method that possess a higher accuracy. Therefore, we fabricated a PDMS microprotrusion with a cross-sectional area of 1 mm × 1 mm, and then controlled the electrode spacing to achieve various microprotrusion structure ratios w/L_{NW}. **Supplementary Fig. 27b** shows the DPTs with various channel lengths on the ZnO nano/microwires and the corresponding structure ratios w/L_{NW}, through adjusting the accurate distance of the source/drain electrodes. Note that when the electrode spacing of the ZnO nano/microwire (the channel length) is 1.5 mm, the microprotrusion structure ratio w/L_{NW} of the DPT could exactly be the optimal value 0.66, which is thus adopted as the practical parameter in the experimental section.

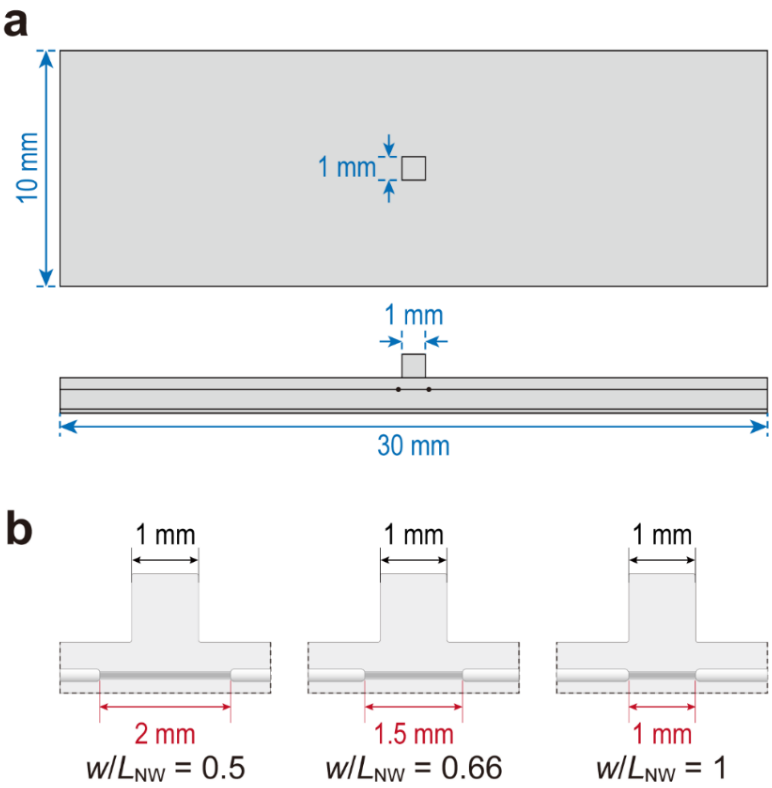

Supplementary Fig. 27 | Dimension of DPTs and method to achieve the structure ratio of 0.66. a, Overview of the DPT with the top view (top) and the side view (bottom). **b**, Schematic illustration of the DPTs with various channel lengths (distances between source and drain electrodes) on the ZnO nano/microwires and the corresponding structure ratios w/L_{NW}.

Supplementary Note 11 | Experimental setup and electrical measurement

The experimental setup and the electrical connection are shown in **Supplementary Fig. 28**. The specific home-made experiment setup together with the optical image of the DPT is shown in **Supplementary Fig. 28a**. The data acquisition card (National Instruments PCI-6259) is used for data acquisition and input the data to the computer. The synthesized function generator (Stanford Model DS 345) is applied to produce a drive voltage on DPT. With the help of shielded junction box, the voltage signal is also imported into the data acquisition card. Meanwhile, the low-noise current preamplifier (Stanford Model SR570) is used to measure the current flow through the DPT and also import the current value into the data acquisition card. In the specific test process for vertical force sensing, a linear actuator is controlled by the computer to apply a displacement on the DPT, and a force sensor under the substrate readouts the force applied on DPT at the same time. The illustration of electrical connection and the circuit diagram are also provided in **Supplementary Fig. 28b** and **Supplementary Fig. 28c**, respectively.

Additionally, in order to quantitatively evaluate the potential influence of the damping effects from PDMS on the force measurement, we also measured the vertical force in different positions. With the configuration as shown in **Supplementary Fig. 29a**, the dynamometer with a flat measurement head was vertically mounted on the linear actuator, which could precisely control the displacement of the microprotrusion surface and directly measure the applied force on the DPT. Meanwhile, the force senor under the substrate could measure the force beneath the DPT. By measuring the force on the microprotrusion (F_1) and the force beneath the DPT (F_2) simultaneously, we can get a relatively accurate force. It should be noted that the force sensor is zeroed after the DPT is placed on it, so F_2 does not include the weight of the DPT. As demonstrated by the test results in **Supplementary Fig. 29b** and **Supplementary Fig. 29c**, the average values of force difference ($\Delta F = F_1 - F_2$) can be controlled within 2 mN and the vertical forces are basically identical with the maximum error $\Delta F/F_2$ less than 3%, which indicates that the PDMS damping effects might actually have little effect on the accuracy of the force measurement.

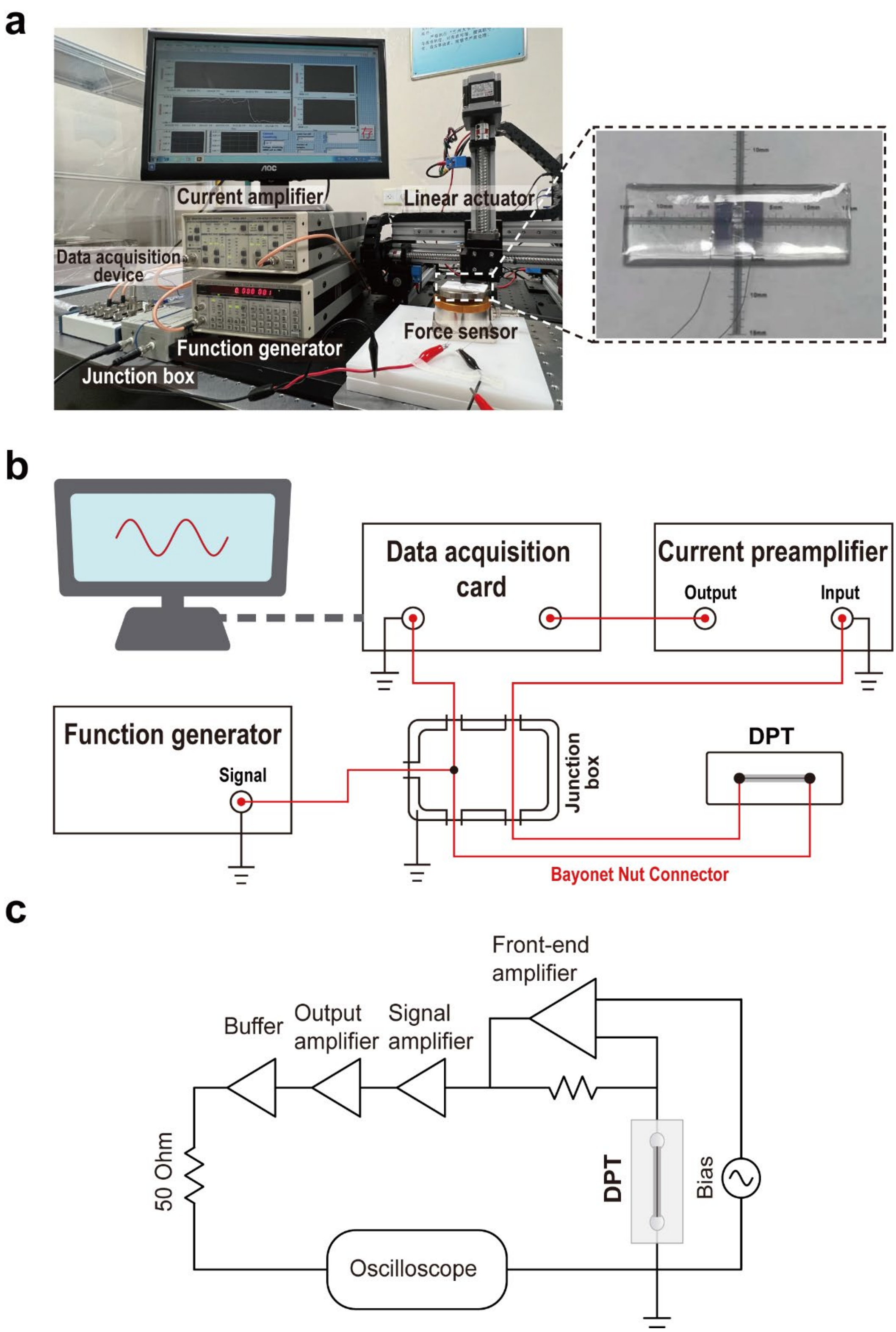

Supplementary Fig. 28 | Experimental setup. a, Home-made experiment setup composed of functional generator, current preamplifier, junction box, data acquisition card and computer. Inset: Optical image of DPT. **b**, Illustration of external electrical connection. **c**, Circuit diagram of measurement.

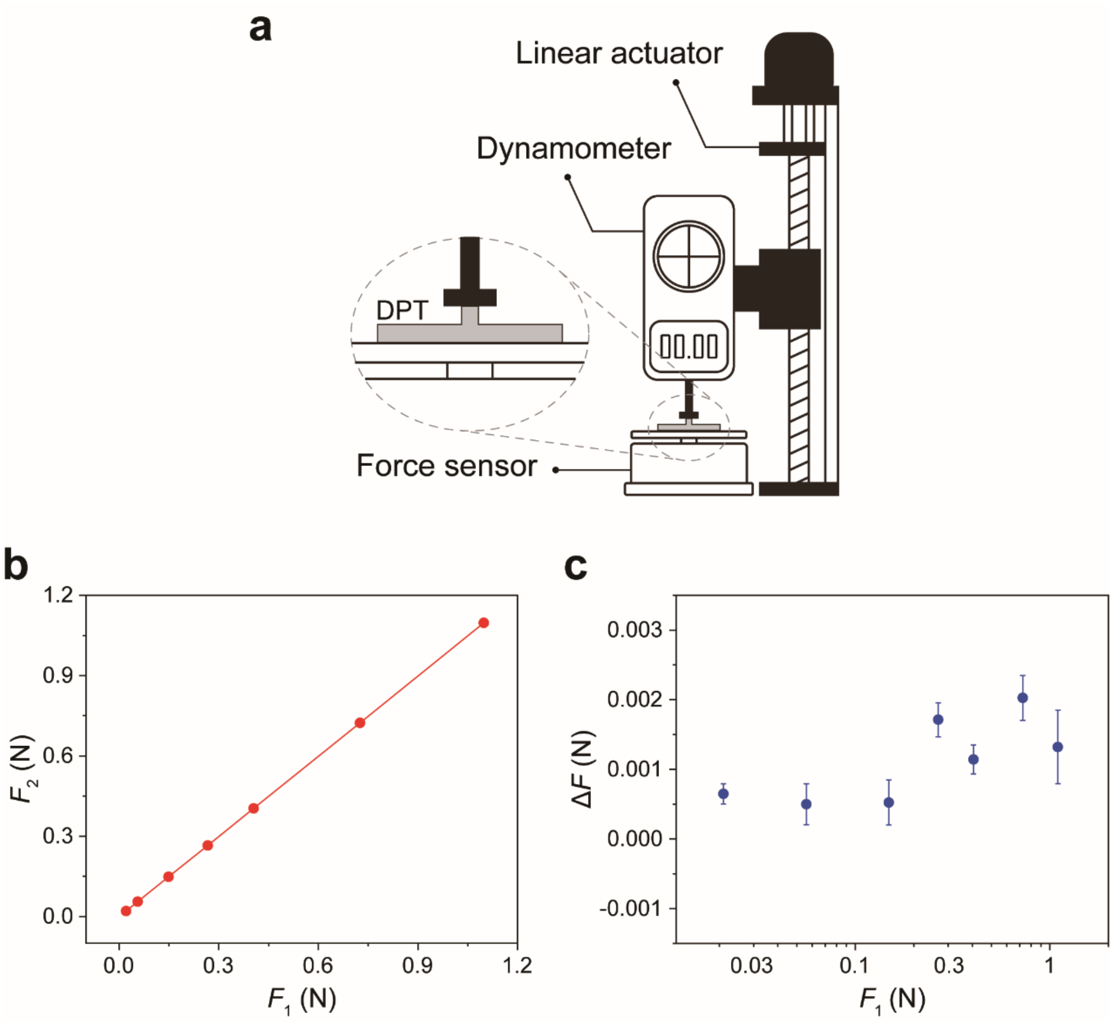

Supplementary Fig. 29 | The relationship of the vertical force on DPTs measured in different positions. a, Schematic illustration of the force measurement system for the DPT in vertical force sensing mode, composed mainly of a force sensor under the substrate of the DPT and a dynamometer vertically mounted on the linear actuator. **b**, Curve of force beneath the DPT (F_2) versus force on the microprotrusion (F_1) measured simultaneously. **c**, Difference value of the measured force ($\Delta F = F_1 - F_2$) from statistics results in **b**. The error bars denote standard deviations of the mean.

Supplementary Note 12 | Three regulations induced by piezoelectric polarization and bias voltage

There involve three regulations induced by piezoelectric polarization and bias voltage, the whole regulation process in piezotronic transistors is very complicated. The three regulations can be labeled as: (1) opposite regulation induced by asymmetric polarization; (2) image force induced by high bias; (3) redistribution of voltage drops induced by high bias. In order to clarify the whole regulation process clearly, we will analyze above three regulations in detail and take the phenomenon* in **Figure 3e** in the manuscript as an example to show what kind of changes in the energy band of the device will be caused by the introduction of piezoelectric polarization and electric field.

*The interesting phenomenon in **Figure 3e**: upon a small force, the slope value (221.5 N^{-1}) at lower bias (+1 V) is much larger than that (89.7 N^{-1}) at higher bias (+3 V); whereas, upon a large force, the slope value (31.7 N^{-1}) at lower bias (+1 V) is slightly smaller than that (43.6 N^{-1}) at higher bias (+3).

(1) Opposite regulation induced by asymmetric polarization

As shown in **Supplementary Fig. 30**, we illustrate the energy bands of device under different bias and different mechanical stimuli. **Supplementary Fig. 30a-c** respectively shows the energy bands without force (**Supplementary Fig. 30a**), under small force (**Supplementary Fig. 30b**) and large force (**Supplementary Fig. 30c**). As can be seen, there are two Schottky barriers (short for SB) in series, through which electrical carriers (mainly electrons in this work) must pass. Here, we define the left one as SB1 and the right one as SB2. As shown in **Supplementary Fig. 30a**, since the SB2 is larger than SB1, the electrical transport characteristics of device are mainly controlled by SB2. In other words, SB2 plays a dominant regulatory role. As the applied force gradually increases (**Supplementary Fig. 30b**), a negative piezoelectric polarization charge is generated at SB1, resulting in an increased Schottky barrier height (SBH1); whereas a positive piezoelectric polarization charge is generated at SB2, resulting in a decreased Schottky barrier height (SBH2). As the force becomes large enough (**Supplementary**

Fig. 30c), the asymmetric polarization and opposite regulation (SBH1 gradually increase, SBH2 gradually decrease) make SB1 and SB2 gradually become similar, and SB2 gradually loses its dominant regulatory role. At this time, SB1 and SB2 both affect the electrical transport, rather than SB1 alone dominates in the case of applying a small force.

We also illustrate the corresponding energy bands of device under low bias (**Supplementary Fig. 30d-f**) and high bias (**Supplementary Fig. 30g-i**). Since SB1 and SB2 are in series, voltage drops of the two barriers ($V_{drop,1}$ for SB1 and $V_{drop,2}$ for SB2) add up to the bias voltage V_{bias}, which can be expressed as following formula (21).

$$V_{bias} = V_{drop,1} + V_{drop,2} \tag{19}$$

Similar to **Supplementary Fig. 30a-c**, the opposite regulation will always make SB2 lower with $V_{drop,2}$ smaller (**Supplementary Fig. 30d-f** under low bias) and SB1 higher with $V_{drop,2}$ larger (**Supplementary Fig. 30g-i** under high bias) as increasing force. This is the reason why the piezotronic modulation gradually decreases with the increase of force under a same bias (as shown in **Figure 3e** in the manuscript).

It should be noted that we only analyzed the piezotronic effect affected by the piezoelectric polarization charge up to now, and did not involve the influences of high bias. Next, we will focus on the analysis of two effects induced by high bias on piezotronic effect as follows.

(2) Image force induced by high bias

On the basis of the aforementioned opposite regulation induced by asymmetric polarization, another effect must be considered as the bias voltage increases, that is, the Schottky effect, especially at SB2 which plays the dominant role when the force is relatively small.

Based on semiconductor theory [42], there will exist an image force at Schottky barrier in the presence of an electric field, which makes the Schottky barrier not that 'sharp' and lead the Schottky barrier height lower. This effect is known as the Schottky

effect or Schottky-barrier lowering. As the voltage bias (electric field) across Schottky barrier increases, the image force will increase and the Schottky barrier height lowering $\Delta\varphi_{\mathrm{image\ force}}$ will become obvious, which can be derived from the following formula.

$$\Delta\varphi_{image\ force} = \sqrt{\frac{qE}{4\pi\varepsilon_s}} \tag{20}$$

in which q is the carrier charge, E represents the electric field at the Schottky barrier, ε_s is the dielectric constant of semiconductor.

Returning to the situation in this work, as shown in **Supplementary Fig. 30e** and **Supplementary Fig. 30h**, under the action of a small force, due to the weak opposite regulation, SB2 maintains its dominant regulatory role no matter under low bias or high bias, and the voltage drop $V_{drop,2}$ on it is approximately equal to the bias voltage V_{bias}. As the bias increases (from **Supplementary Fig. 30e** to **Supplementary Fig. 30h**), a gradually increased electric field falls on SB2, making Schottky effect become obvious.

In order to clarify the influence of image force induced by high bias on the piezotronic regulation, we illustrate the piezotronic modification of the energy band (conductive band) without image force (**Supplementary Fig. 31a**) and with image force (**Supplementary Fig. 31b**). In addition to the previously mentioned image force will reduce the Schottky barrier height, the image force will also cause the barrier to shift away from the interface between the metal and the semiconductor by a distance of x_m (**Supplementary Fig. 31b**). This will cause the piezoelectric polarization charge to stagger from the top position of barrier and reduce the piezotronic effect. In other words, the existence of the image force induced by high bias weakens the regulation of the piezotronic effect on the energy band. This is the main reason why piezotronic regulation weakens with the increase of bias when the force is small. It should be noted that the piezotronic regulation weakening here not only comes from the image force, but also is related to the narrower depletion layer width and the stronger electrostatic shielding of the polarization charges by the free carriers under high bias.

For the case of large force, the image force will also exist. However, due to the obvious opposite regulation, the voltage drop of SB2 is not high (and the electric field

is not high), and the influence of image force is limited. In this case, another effect induced by high bias needs to be considered.

(3) Redistribution of voltage drops induced by high bias

Upon a large force, opposite regulation previously discussed in (1) will make SB2 gradually lose its dominant regulatory role as illustrated in **Supplementary Fig. 30c** or **Supplementary Fig. 30f**. At this time, the SBH1 and SBH2 are appropriate, and their voltage drops ($V_{drop,1}$ and $V_{drop,2}$) are also appropriate. As the bias become high (**Supplementary Fig. 30i**) from low (**Supplementary Fig. 30f**), there is an easily overlooked effect, that is, redistribution of voltage drops between two Schottky barriers induced by high bias, will become important.

Energy bands of two Schottky barriers with similar barrier height in series under different biases are illustrated in **Supplementary Fig. 32**. When the bias tends to 0 V (**Supplementary Fig. 32a**), the forward biased SB1 and the reverse biased SB2 are similar in barrier height, and it can be considered that their voltage drops ($V_{drop,1}$ for SB1 and $V_{drop,2}$ for SB2) are approximately equal to $V_{bias}/2$. In this case, both SB1 and SB2 determinate the electrical transport of device. As the bias gradually increase to a small value (**Supplementary Fig. 32b**), SB1 will decrease, and its voltage drop $V_{drop,1}$ will decrease to a relatively small value $V_{bias}/2 - \Delta$, compared with that ($V_{bias}/2 + \Delta$) of SB2. Since the bias is very low, the difference 2Δ of voltage drops between the two is small, and the voltage drops of the two is approximately equal to $V_{bias}/2$. In this case, SB1 and SB2 still determinate the electrical transport of device together. Therefore, when the bias is low, both SB1 and SB2 control the device transport. Since the opposite regulation exists between SB1 and SB2, the overall performance of piezotronic effect (sensitivity value) is relatively weak.

As the bias furtherly increases to a high value, SBH1 will furtherly decrease (finally tend to 0), and 2Δ is a considerable large value. At this time, the voltage drop of SB2 is $V_{drop,2}$ far larger than $V_{bias}/2$ and tends to V_{bias}. Usually, the value of $V_{drop,2}/(V_{drop,1} + V_{drop,2})$ is more than 75%, and gradually increase with applied bias. In other words, when bias is high, the reverse biased SB2 will play a dominant

regulatory role in controlling the device transport. So, the piezotronic modification of SB2 will totally determine the device, resulting a high overall performance of piezotronic effect (force sensitivity value).

It should be noted that the image force also exists when large force is applied. The influence of image force induced by high bias and the influence of redistribution of voltage drops induced by high bias offset each other, resulting in only a slightly enhanced force sensitivity value at bias of 3 V compared with that at 1 V. Maybe in some case, it is entirely possible that the force sensitivity value at 3V is smaller than or equal to that at 1 V.

Through the above qualitative analysis, the opposite regulation induced by asymmetric polarization, the image force induced by high bias and the redistribution of voltage drops induced by high bias all affect the overall piezotronic regulation process, resulting in the interesting phenomenon. With the increase of bias, what happens to the interface engineering by polarizations including piezoelectric polarization, flexoelectric polarization and ferroelectric polarization? This question is an important and cutting-edge research topic in the field of piezotronics and flexotronics (maybe also in ferroelectric electronics), which has rarely been discussed before. More in-depth investigations are needed in the further.

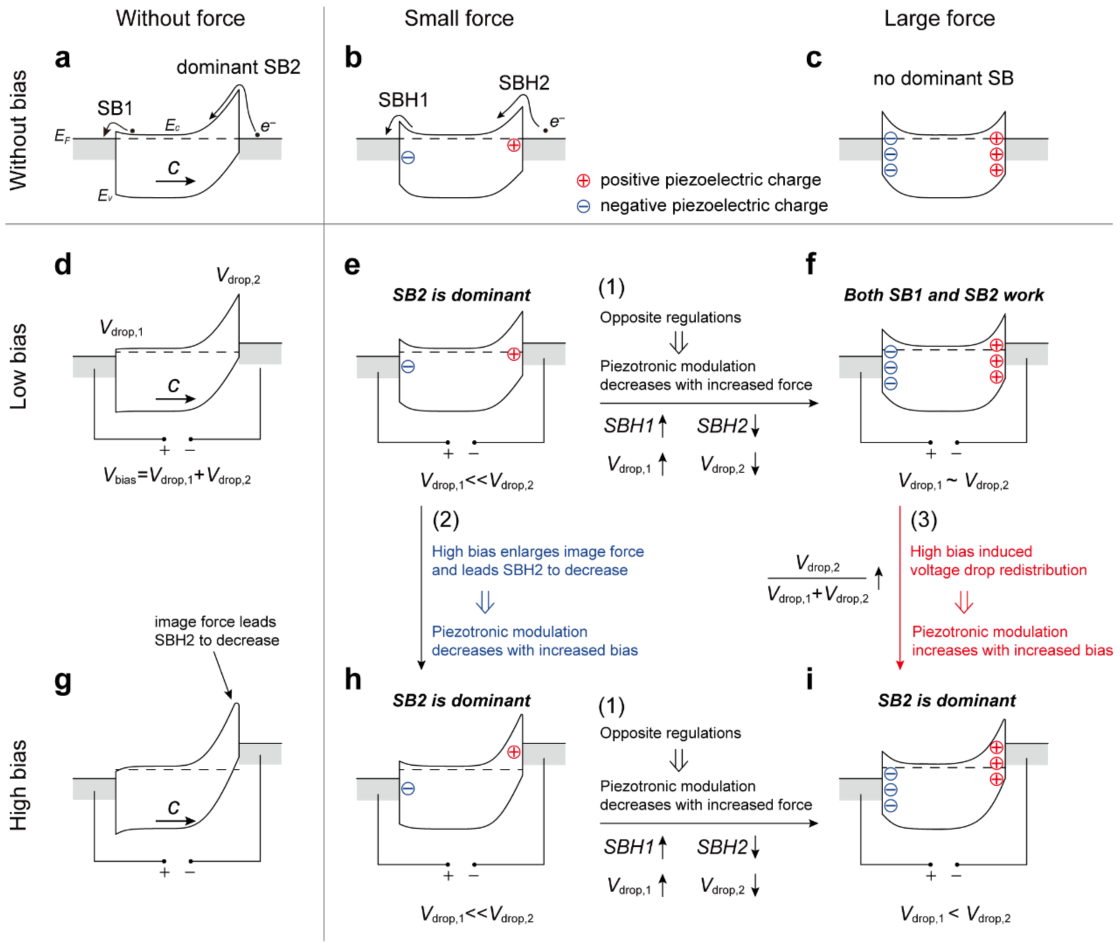

Supplementary Fig. 30 | Energy bands of device under different bias and different force. a-c, The energy bands under no bias without force (**a**), with a small force (**b**) and a large force (**c**). **d-f**, The energy bands under low bias without force (**d**), with a small force (**e**) and a large force (**f**). **g-i**, The energy bands under high bias without force (**g**), with a small force (**h**) and a large force (**i**). The left Schottky barrier, Schottky barrier height and corresponding voltage drop are respectively short for SB1, SBH1 and $V_{drop,1}$. The right Schottky barrier, Schottky barrier height and corresponding voltage drop are respectively short for SB2, SBH2 and $V_{drop,2}$. The bias voltage is V_{bias}.

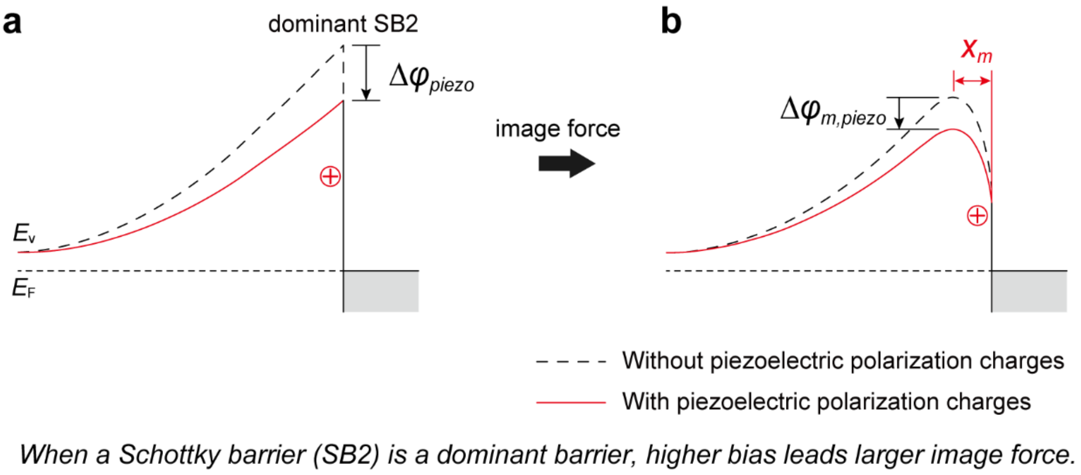

Supplementary Fig. 31 | Influence of the image force induced by high bias on piezotronic modification of energy bands. a, Piezotronic modification of conductive band without image force. The $\Delta\varphi_{piezo}$ represents the change of Schottky barrier height produced by piezotronic effect. **b**, Piezotronic modification of conductive band with image force. The $\Delta\varphi_{m,piezo}$ represents the change of Schottky barrier height in the existence of image force produced by piezotronic effect. The x_m is the displacement between the top of barrier and the interface induced by image force.

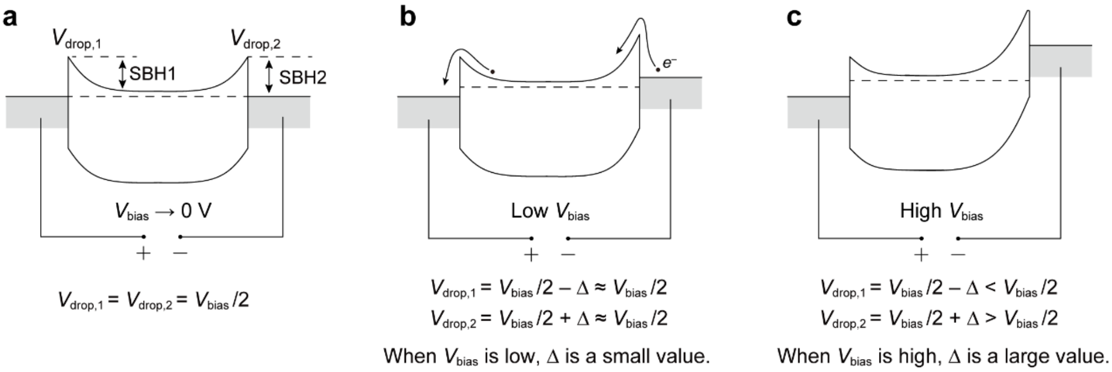

Supplementary Fig. 32 | Change of energy bands and Redistribution of voltage drops induced by high bias. a-c, Diagrams of energy bands under a bias tending to zero (**a**), under a low bias (**b**) and under a high bias (**c**). The Schottky barrier heights (corresponding voltage drops) in the left side and in the right side are respectively short for SBH1 ($V_{drop,1}$) and SBH2 ($V_{drop,2}$). The 2Δ represents the difference between $V_{drop,1}$ and $V_{drop,2}$. The bias voltage is V_{bias}.

Supplementary Note 13 | Calculation of the change of effective Schottky barrier height

According to the piezotronic effect [1], the force/strain induced piezoelectric polarization charges can effectively modulate the interface Schottky barrier at M-S contact (between metal and n-type piezoelectric semiconductor), so as to control the charge carrier transport characteristics. For the metal-semiconductor-metal (MSM) structure in the device, most of the voltage will be mainly consumed by the reverse-biased Schottky barrier [43], which dominates the piezotronic regulation.

Based on the previous studies [42], the reverse current I through the Schottky barrier follows the equation (for $V \gg 3kT/q$):

$$I \approx SA^{**}T^2 e^{-\frac{\varphi_{\mathrm{Bn}}+\Delta\varphi_{\mathrm{piezo}}-\Delta\varphi_{\mathrm{image-force}}}{kT}} \approx SA^{**}T^2 e^{-\frac{\varphi_{\mathrm{Bn}}+\Delta\varphi_{\mathrm{piezo}}}{kT}} e^{\frac{q\sqrt{q\xi_{\mathrm{m}}/4\pi\varepsilon_{\mathrm{S}}}}{kT}} \tag{21}$$

where S is the area of the Schottky contact, A^{**} is the effective Richardson constant, T is the absolute temperature, φ_{Bn} is the Schottky barrier height (SBH) without piezoelectric charges, $\Delta\varphi_{\mathrm{piezo}}$ is the change of SBH induced by the piezoelectric charges, k is the Boltzmann constant, q is the electron charge, and

$$\xi_{\mathrm{m}} = \sqrt{\frac{2qN_{\mathrm{D}}}{\varepsilon_{\mathrm{S}}}\left(V + V_{\mathrm{bi}} - \frac{kT}{q}\right)} \tag{22}$$

in which N_{D} is the donor concentration, ε_{S} is the dielectric constant of ZnO, and V_{bi} is the built-in potential.

Then we can derive the current, given by:

$$I \approx SA^{**}T^2 e^{-\frac{\varphi_{\mathrm{Bn}}+\Delta\varphi_{\mathrm{piezo}}}{kT}} e^{\frac{\sqrt[4]{q^7 N_{\mathrm{D}}(V+V_{\mathrm{bi}}-kT/q)/(8\pi^2\varepsilon_{\mathrm{S}}{}^3)}}{kT}} \tag{23}$$

The change of SBH ($\Delta\varphi_{\mathrm{piezo}}$) induced by the piezoelectric polarization charges can be derived as follows:

$$I_{\mathrm{free}} \approx SA^{**}T^2 e^{-\frac{\varphi_{\mathrm{Bn}}}{kT}} e^{\frac{\sqrt[4]{q^7 N_{\mathrm{D}}(V+V_{\mathrm{bi}}-kT/q)/(8\pi^2\varepsilon_{\mathrm{S}}{}^3)}}{kT}}$$

$$I_{\mathrm{strain}} \approx SA^{**}T^2 e^{-\frac{\varphi_{\mathrm{Bn}}+\Delta\varphi_{\mathrm{piezo}}}{kT}} e^{\frac{\sqrt[4]{q^7 N_{\mathrm{D}}(V+V_{\mathrm{bi}}-kT/q)/(8\pi^2\varepsilon_{\mathrm{S}}{}^3)}}{kT}}$$

$$\Rightarrow \Delta\varphi_{\text{piezo}} = -kT \ln(I_{\text{strain}}/I_{\text{free}})$$

(24)

where I_{strain} and I_{free} are the current measured at a fixed bias with and without external force/strain, respectively; k is the Boltzmann constant, T is the absolute temperature.

Therefore, the change of effective SBH under strain can be evaluated from the *I-V* characteristics by the formula (24) given above.

Supplementary Table 4 | Summary of pressure sensitivity of piezotronic sensors

Materials	Morphology	Pressure sensitivity (meV/MPa)	Reference
ZnO	MW	~1.759×10^3	This work
ZnO	Bulk	0.17	[44]
ZnO	Bulk	0.3	[45]
ZnO	Bulk	1.26	[46]
ZnO	NW	0.083~0.422	[6]
ZnO	NW	0.308~0.487	[47]
ZnO	NW	0.148~0.166	[48]
CdSe	NW	~0.677	[5]
GaN	NW	0.212~0.24	[7]
ZnO	Nanotube	0.771~2.255	[49]
ZnO	NW cluster	0.287~0.318	[50]
ZnO	NW cluster	<33.39	[4]
ZnO	Nanosheet	25.11~48.18	[17]
ZnO	Nanosheet	28.22~46.65	[51]
ZnO	Nanosheet	9.94~21.39	[52]
ZnO	Nanoplatelet	60.97~78.23 (Single Channel)	[8]
ZnO	Nanoplatelet	84.2~104.4 (Double Channel)	[53]

Supplementary Table 5 | Comparison of sensitivity of force/pressure sensors

Materials	Morphology	Type	Sensitivity (MPa^{-1})	Reference
ZnO	MW	Piezotronic	226.67	This work
Ga-doped MgZnO	Film	Piezotronic	31.36	[54]
InGaN/GaN MQW	Nanopillar	Piezotronic	6.11	[55]
Ag NW/rGO	Film	Piezoresistive	5800	[56]
Si	Nanorod array	Piezoresistive	0.49	[57]
PANI/MWCNT	composite	Piezoresistive	13~15	[58]
PPy foam	Film	Piezoresistive	260	[59]
Graphene/PDMS	Sponge	Piezoresistive	1.522×10^4	[60]
Pd	Nanoparticle array	Piezoresistive	130	[61]
Au/parylene-polyurethane nanomesh		Capacitive	141	[62]
Micro-patterned porous PDMS layer		Capacitive	143.5	[63]
CNF + Ag NF hybrid network		Capacitive	~1.78	[64]
PDMS/Ag/Ecoflex	Film	Capacitive	1.45	[65]
Carbon black/silicone rubber composite		Capacitive	0.2536	[66]
PDMS/Ag NW/Ecoflex	Film	Capacitive	0.57	[67]
CNT-doped PDMS/Ecoflex	Film	Capacitive	0.417	[68]

MW: Microwire as the single piezoelectric component. NW: Nanowire as the single piezoelectric component. NW cluster: Nanowire cluster as the single piezoelectric component. Nanoplatelet: Nanoplatelet as the single piezoelectric component. Twin nanoplatelet: Twin nanoplatelet as the single piezoelectric component.

Supplementary Note 14 | Weakened piezotronic modulation in the DPT for vertical force sensing

In the DPT for vertical force sensing, the slope of $\Delta I/I_0$ versus force is relatively small as the vertical force is relatively large, indicating a weakened piezotronic modulation occurred. To deeply reveal the possible regulation mechanisms of the piezotronic effect on the DPT in vertical force sensing mode, we carefully investigate the piezotronic modulation on the back-to-back Schottky barriers (two Schottky barriers connected back-to-back).

Supplementary Fig. 33a exhibits the typical current of the metal-semiconductor-metal (MSM) structure in the DPT for vertical force sensing under bias of 1 V. As can be seen that the current firstly changes approximately linearly with the vertical force increasing from 0.0 to 0.6 N, whereas the current changes slightly as the applied force is further increased. Also, based on the above derived formula (24) in **Supplementary Note 13**, the force-induced effective changes of SBH are denoted as $(\Delta SBH)_{\mathrm{eff}}$, which can be expressed as:

$$(\Delta SBH)_{\mathrm{eff}} = \Delta\varphi_{\mathrm{piezo}} \propto \ln(I_{\mathrm{force}}/I_{\mathrm{free}}) \tag{25}$$

Thus, the effective change of barrier height $(\Delta SBH)_{\mathrm{eff}}$ satisfies a linear relationship with $\ln(I_{\mathrm{force}}/I_{\mathrm{free}})$. Through the data analysis from **Supplementary Fig. 33a**, we can plot $\ln(I_{\mathrm{force}}/I_{\mathrm{free}})$ that reflects the effective change of the overall barrier height of the back-to-back Schottky barriers as a function of applied force in **Supplementary Fig. 33b**, in which the value of $\ln(I_{\mathrm{force}}/I_{\mathrm{free}})$ first changes dramatically with force in the red region (I) and then the increasing magnitude becomes small gradually in the blue region (II).

As illustrated in **Supplementary Fig. 33c**, when the applied force is relatively small corresponding to the red region in **Supplementary Fig. 33b**, the substantially change of the $\ln(I_{\mathrm{force}}/I_{\mathrm{free}})$ or $(\Delta SBH)_{\mathrm{eff}}$ mainly results from the piezotronic modification on the dominant SBH (the right Schottky barrier illustrated in **Supplementary Fig. 33c-I**) which possesses a relatively higher barrier height between

the back-to-back Schottky barriers. In this case, the positive piezoelectric polarization charges on the right side of DPT will induce a reduction of the SBH, which enables electrons to only require lower energy to cross the barrier, leading to a rapidly increased current (lower, **Supplementary Fig. 33c-I**). However, as the force is large enough corresponding to the blue region in **Supplementary Fig. 33b**, the force-induced increase of the barrier height on the left will not be negligible, while the original dominant Schottky barrier on the right continues to decrease. As shown in the lower side of **Supplementary Fig. 33c-II**, one ebbs and the other grows, leading to the piezotronic modulation of the original dominant barrier on the right that might no longer be dominant. As a result, the changes of the current or $(\Delta SBH)_{\text{eff}}$ with the increasing force gradually slow down. Therefore, the DPT in vertical force sensing mode exhibits a high sensitivity, particularly in detecting a small force, while a relatively small sensitivity in detecting a large force.

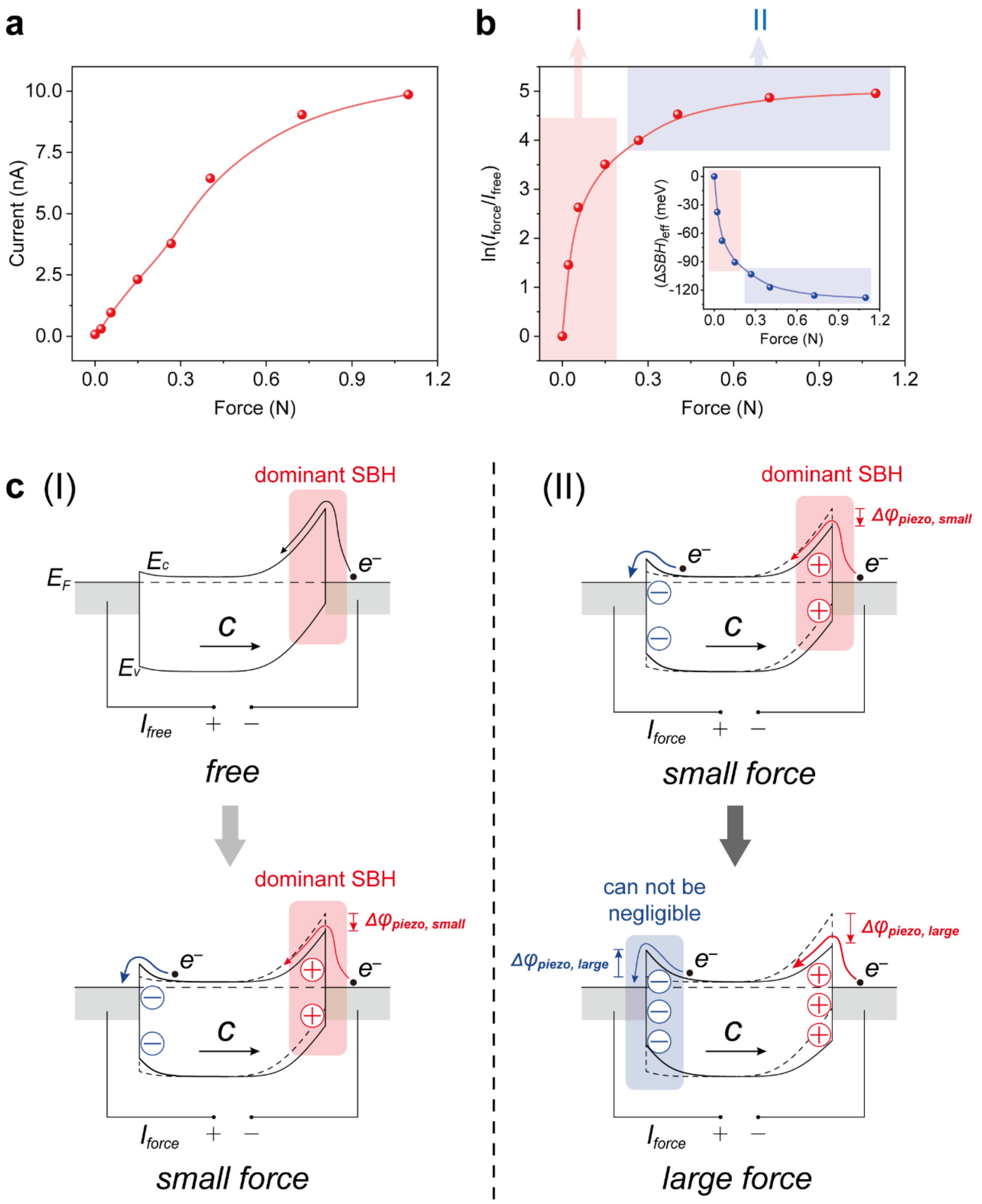

Supplementary Fig. 33 | Piezotronic modulation of the dominant Schottky barrier height (SBH) on electrical transport. a, Current as a function of applied force under bias of 1 V. **b**, $\ln(I_{\text{force}}/I_{\text{free}})$ versus vertical force curve derived from (**a**). The inset is the corresponding change of effective SBH: $(\Delta SBH)_{\text{eff}}$. **c**, Energy band diagrams of DPT with a dominant Schottky barrier showing different regulation mechanisms under (c-I) small force and (c-II) large force.

Supplementary Note 15 | Definition of the change ratio in piezotronic transistors

Both current change ratio and resistance change ratio are adopted in piezotronic devices (*e.g.* piezotronic transistors). For example, in **Figure 4** in the manuscript, resistance change ratio is used when characterizing DPT under forward bias; while current change ratio is used when characterizing DPT under reverse bias. This is totally different from sensors based on piezoresistive effect. The main reason lies on the strain-induced asymmetric modulation of electrical transport by piezotronic effect. In order to reveal this special definition of change ratio in piezotronic devices more clearly, here we will spend a lot of space to discuss (1) different regulation mechanisms of traditional piezoresistive effect and piezotronic effect, and (2) definition of change ratio in asymmetric strain-controlled piezotronic devices, and (3) examples for demonstration.

(1) Different regulation mechanisms of traditional piezoresistive effect and piezotronic effect

Both resistance change ratio and current change ratio are widely used parameters to characterize the performance of sensors, especially in traditional piezoresistive materials or devices. Usually, we use resistance change ratio to characterize the device possessing increased resistance with applied strain, while use current change ratio to characterize the device possessing increased current with applied strain. However, it is rare that both are used at the same time. With the emerging of strain-controlled devices in new principles (*e.g.* piezotronic effect), the applicable scenarios of resistance change ratio and current change ratio have also been updated.

① *Strain-induced* ***symmetric*** *modulation of electrical transport by piezoresistive effect*

In piezoresistive effect, as shown in **Supplementary Fig. 34a**, if a tensile strain increases the resistance of the device, no matter the device is forward biased or reverse biased, the forward current and reverse current of the device will both decrease with the tensile strain. As also shown in **Supplementary Fig. 34b**, if a compressive strain decreases the resistance of the device, no matter the device is forward biased or reverse biased, the forward current and reverse current of the device will both increase with the compressive strain. As a result, the electrical transport characteristics of the device is

illustrated in **Supplementary Fig. 34c**, indicating that the strain-induced modulation of electrical transport by piezoresistive effect is a symmetric effect. Apart from above case, if a tensile strain decreases the resistance and a compressive strain increases the resistance, the same conclusion will also be obtained, indicating a symmetric strain-induced modulation of electrical transport by piezoresistive effect. So, it is the strain-induced **symmetric modulation** of electrical transport by piezoresistive effect.② *Strain-induced **asymmetric** modulation of electrical transport by piezoelectric charges (piezotronic effect)*

Piezotronic effect is an interface effect that asymmetrically modulates local contacts at different terminals of the device [4], as schematically illustrated in the following **Supplementary Fig. 35**. Schottky junctions will be formed in local contacts at different terminals of the device without strain (**Supplementary Fig. 35a**). Due to the accidental error/uncertainty of device manufacturing [69], the Schottky junctions at both ends will be slightly different.

When subjecting to a tensile strain (**Supplementary Fig. 35b**), positive piezoelectric polarization charges are produced at the surface of the ZnO nano/microwire (assuming its piezoelectric *c*-axis points to the left) on the left contact of device, which will reduce the barrier height of the Schottky junction; while negative polarization charges are produced on the right contact, leading to an increasement of the barrier height of the Schottky junction. When a forward bias (potential of right electrode is higher than that of left electrode, **left** in **Supplementary Fig. 35b**) is applied, the electrical transport characteristics are mainly determined by the Schottky junction on the left terminal, which is reversely biased. Because of the decreased barrier height of the left contact, the current flowing through the device will increase and become larger than that of device without strain. Similarly, when the device is reversely biased (potential of right electrode is lower than that of left electrode, **right** in **Supplementary Fig. 35b**), under a same bias voltage, the (reverse) current of the device will be smaller than that without strain. Therefore, compared to the case without strain, the forward current of the device with tensile strain will increase; while the reverse current will decrease. With the same principle, we can also analyze the

modulation of the current of the device under a compressive strain, as shown in **Supplementary Fig. 35c**. We can see that the compressive strain will cause a decrease of the forward current (**left** in **Supplementary Fig. 35c**) and an increase of the reverse current (**right** in **Supplementary Fig. 35c**).

The corresponding *I-V* curves can be schematically plotted in the **Supplementary Fig. 35d**. The strain-induced modulation of electrical transport by piezoelectric charges (piezotronic effect) is asymmetric. The piezotronic effect modulation arises as a result of the polarization of nonmobile ions in the piezoelectric crystal [2, 3]. Thus, it is a strain-induced **asymmetric modulation** of electrical transport by piezotronic effect.

Therefore, different from the symmetric modulation by traditional piezoresistive effect, an asymmetric modulation is usually made by piezotronic effect, in which the current increases with strain under one bias, while the current decreases with strain under opposite bias.

(2) Definition of change ratio in asymmetric strain-controlled piezotronic devices

As for piezoresistive effect, if the resistances under forward bias and reverse bias both increases with applied strain, we can use the resistance change ratio $\Delta R/R_0$ (in which R_0 is the original resistance, and ΔR is the change in resistance) to characterize the piezoresistive device. However, when we apply resistance change ratio $\Delta R/R_0$ to the piezotronic devices shown in **Supplementary Fig. 35c** at the same time, the problem arises. Here, considering the situation upon a compressive strain, the forward current decreases (forward resistance increase) and the reverse current increases (reverse resistance decreases) with strain. As a result, we can get the following two formulas

$$(\frac{\Delta R}{R_0})_{forward} = \frac{R_{\varepsilon,forward} - R_0}{R_0} > 0 \tag{26}$$

$$(\frac{\Delta R}{R_0})_{reverse} = \frac{R_{\varepsilon,reverse} - R_0}{R_0} = \frac{R_{\varepsilon,reverse}}{R_0} - 1 < 0 \tag{27}$$

in which R_ε is the resistance under strain ε.

The better the performance of the piezotronic device upon compressive strain in **Supplementary Fig. 35c**, the larger $R_{\varepsilon,forward}$ and the smaller $R_{\varepsilon,reverse}$, and hence the larger $(\Delta R/R_0)_{forward}$ but the closer $(\Delta R/R_0)_{reverse}$ is to -1! We can find that in this case, the resistance change ratio $\Delta R/R_0$ can well characterize the sensing performance of the device under forward bias, but it fails to characterize the performance of the device under reverse bias. At this time, using the resistance change ratio $\Delta I/I_0$ (in which I_0 is the original current, and ΔI is the change in current) to characterize the performance of the device under reverse bias is very appropriate. For example, in this case, we can write

$$(\frac{\Delta I}{I_0})_{reverse} = \frac{I_{\varepsilon,reverse} - I_0}{I_0} = \frac{I_{\varepsilon,reverse}}{I_0} - 1 \tag{28}$$

in which I_ε is the resistance under strain ε. The better the performance of the piezotronic device upon compressive strain in **Supplementary Fig. 35c**, the larger $(\Delta R/R_0)_{forward}$ and the larger $(\Delta I/I_0)_{reverse}$.

Based on the above considerations, in order to allow a parameter to reflect the performance of the piezotronic device, we use the resistance change ratio $\Delta R/R_0$ to characterize DPT when the resistance increases with the applied strain, which is corresponding to the condition of forward bias; and use the current change ratio $\Delta I/I_0$ to characterize DPT when the current increases with the applied strain, which is corresponding to the condition of reverse bias. In fact, we would also use both resistance change ratio and current change ratio in the vertical force sensing mode, but because of its small reverse current, we only calculated the current change ratio under forward bias.

(3) Examples for demonstration

As a demonstration shown in **Supplementary Fig. 36a**, the forward current decreases with the compressive strain, while the reverse current increases with the compressive strain. In this situation, we can plot the current change ratio under reverse bias as shown in **Supplementary Fig. 36b**, and plot the resistance change ratio under forward bias as

shown in **Supplementary Fig. 36d**. As a contrast, if conversely, we will get the resistance change ratio under reverse bias in **Supplementary Fig. 36c**, and get the current change ratio under forward bias in **Supplementary Fig. 36e**. It can be seen from **Supplementary Fig. 36c** and **Supplementary Fig. 36e** that the better the device performance, the closer the change ratios are to -1, which cannot properly characterize the performance of the devices.

Therefore, due to the strain-induced asymmetric modulation of electrical transport by piezotronic effect, we use resistance change ratio when characterizing DPT under forward bias, and use current change ratio when characterizing DPT under reverse bias.

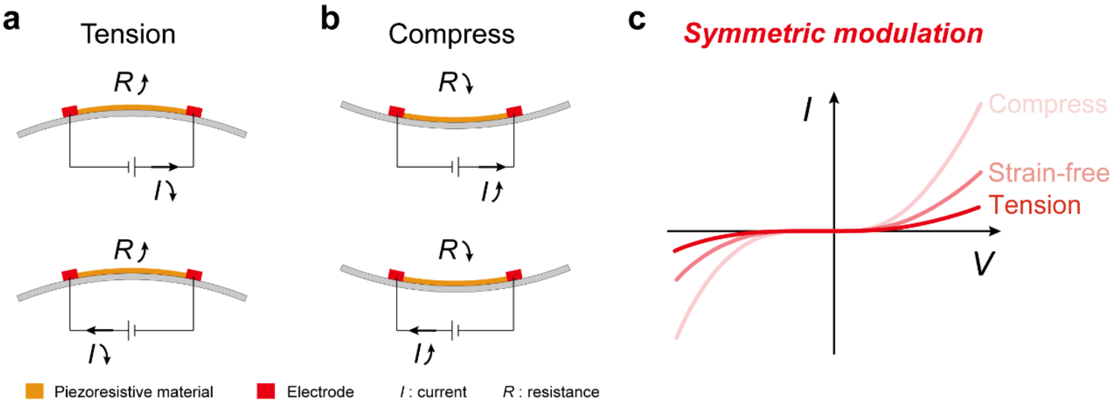

Supplementary Fig. 34 | Strain-induced symmetric modulation of electrical transport by piezoresistive effect. a, Tensile strain increases the resistances and decreases the currents both under forward bias and reverse bias. **b**, Compressive strain decreases the resistances and increase the currents both under forward bias and reverse bias. **c**, Schematic illustrations of the *I-V* curves of the device under no strain, tensile strain and compressive strain, which indicate a symmetric modulation of electrical transport by strain in piezoresistive effect.

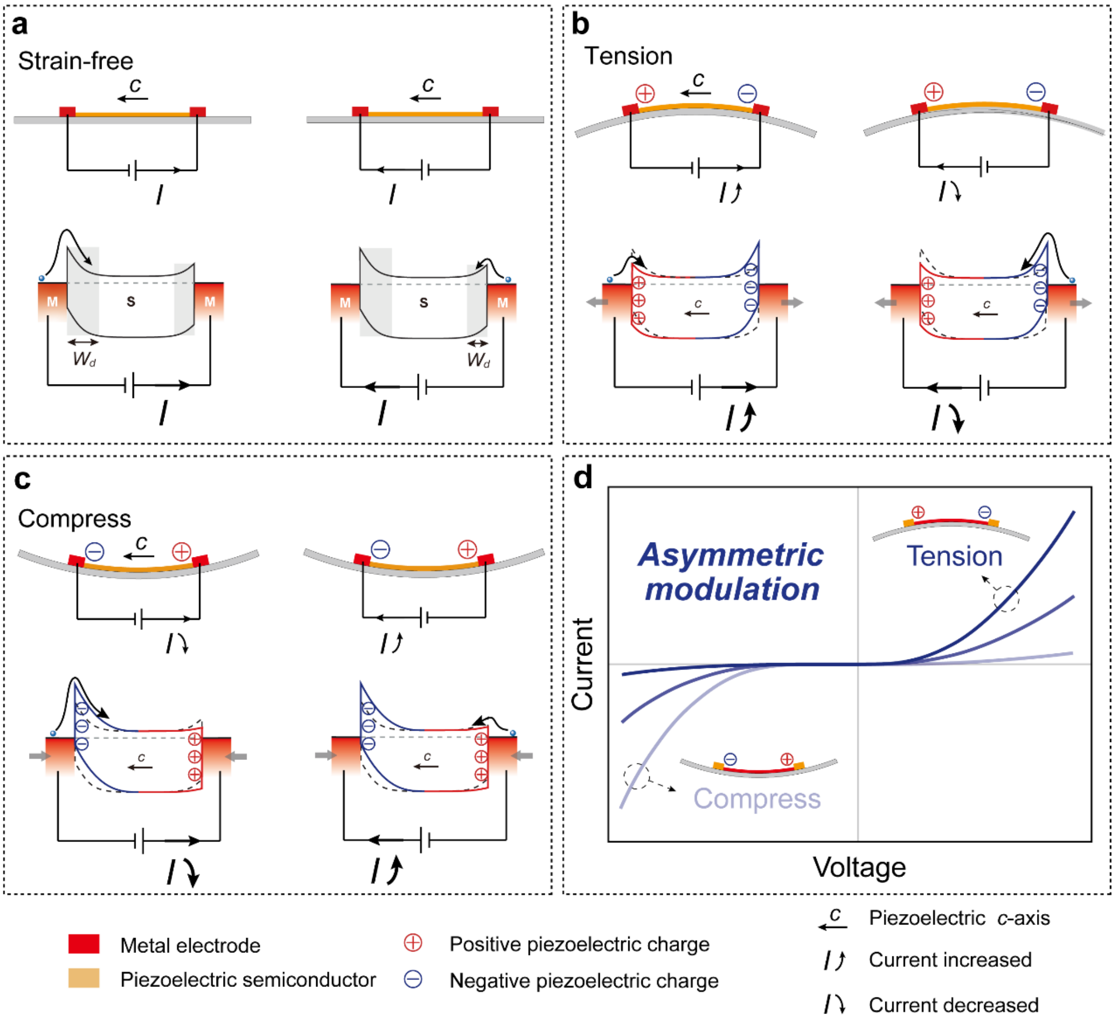

Supplementary Fig. 35 | Strain-induced asymmetric modulation of electrical transport by piezoelectric charges (piezotronic effect). a-c, Schematic diagrams of bending situations and corresponding energy bands of device with no strain (**a**), tensile strain (**b**) and compressive strain (**c**). **d**, Schematic illustrations of the *I*-*V* curves of the device under no strain, tensile strain and compressive strain, which indicate an asymmetric modulation of electrical transport by strain in piezotronic effect.

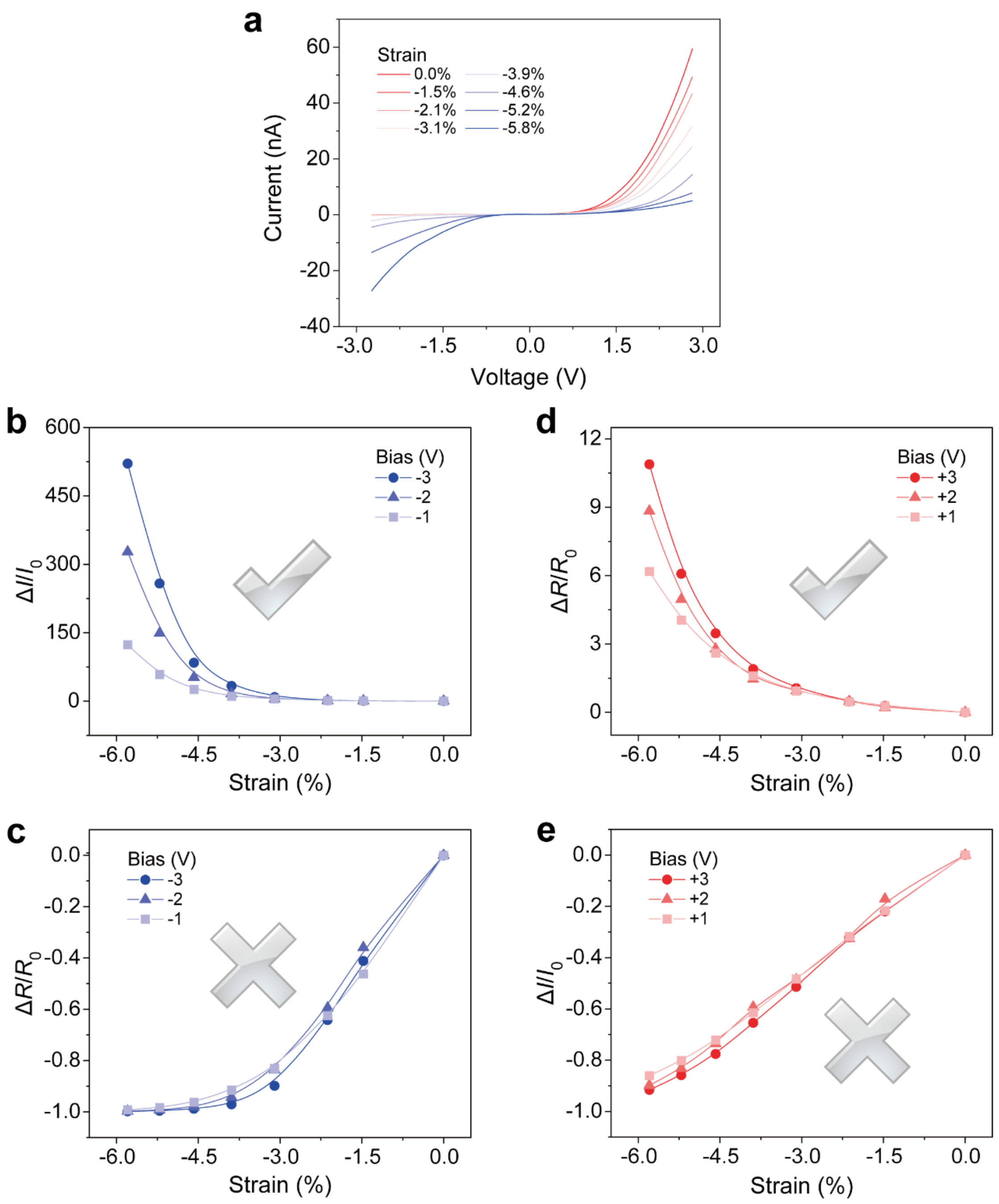

Supplementary Fig. 36 | Resistance change ratio ($\Delta R/R_0$) and current change ratio ($\Delta I/I_0$) used to characterize the piezotronic device under forward bias and reverse bias. a, Strain-induced asymmetric modulation of electrical transport by piezotronic effect. **b** and **c**, Current change ratio (**b**) and resistance change ratio (**c**) as functions of the strain under reverse bias. **d** and **e**, Resistance change ratio (**d**) and current change ratio (**e**) as functions of the strain under forward bias.

Supplementary Note 16 | Definition of the gauge factor in piezotronic transistors

Calculating gauge factors by adopting the resistance ratio under one bias while the current ratio under the other bias is a unique issue in the field of piezotronics, which is attributed to the strain-induced asymmetric modulation of electrical transport by piezotronic effect (as previously discussed in **Supplementary Note 15**). This is completely different from the traditional piezoresistive effect. In order to more clearly define the gauge factor in piezotronic transistors and reveal the reason, we give the definitions of the gauge factor in devices based on piezoresistive effect and devices based on piezotronic effect in contrast.

(1) Definition of gauge factor in devices based on piezoresistive effect

When a device is subjected to external stimuli, its electrical transport changes with the applied strain. This phenomenon is called the resistance-strain deformation effect of the sensor, which is characterized by a sensitivity coefficient named gauge factor. Gauge factor is originally used to indicate the sensitivity of the device performance in response to mechanical stimuli based on piezoresistive effect, and is now also used to indicate the strain sensing performance of piezotronic devices.

As an important parameter for evaluating the device performance, gauge factor of the strain sensor based on piezoresistive effect can be generally defined as the ratio of relative change in resistance to the applied strain based on the electromechanical performance [70, 71], which can be calculated using the following equation:

$$gauge\ factor = \frac{\Delta R/R_0}{\Delta\varepsilon} = \frac{R_\varepsilon - R_0}{R_0 \cdot \Delta\varepsilon} \tag{29}$$

where R_0 is the initial resistance under no strain; R_ε is the resistance at the strain of ε; $\Delta\varepsilon$ denotes the change of the strain applied to the sensor.

Piezoresistive effect is a change in the electrical resistance of a semiconductor or metal when mechanical strain is applied. It can be observed in a metal or a semiconductor, while the piezoresistive effect in semiconductor materials is generally much stronger than in metals. The resistance (R) of a material is defined as: $R = \rho \cdot$

l/a, where l is the length, a is the cross-sectional area of the material and ρ is electrical resistivity. In the materials with piezoresistive effect, the gauge factor can be also described as follows:

$$gauge\ factor = \frac{\Delta R/R_0}{\Delta\varepsilon} = 1 + 2v + \frac{\Delta\rho/\rho_0}{\Delta\varepsilon} \tag{30}$$

in which v is the Poisson's ratio, ρ_0 is the original electrical resistivity, and $\Delta\rho$ is the change in electrical resistivity. The first term $1 + 2v$ represents the devotion provided by the strain-induced geometric structure change, and the second term $(\Delta\rho/\rho_0)/\Delta\varepsilon$ represents the devotion provided by the strain-induced resistivity change. Generally, in piezoresistive metals, the first term determines the gauge factor; while in piezoresistive semiconductors, the second term is much larger than the first term. For example, the gauge factor of thin-film metal is about 2-5, and the gauge factor of single crystal silicon can reach 200 (a very high value in piezoresistive strain sensors).

Back to the formula (29), upon a same strain, the device with higher gauge factor means higher resistance change ratio $\Delta R/R_0$ and better device performance. This is always right in the traditional piezoresistive effect. However, with the emerging of strain-controlled devices with new principles (*e.g.* piezotronic effect), the definition of gauge factor has also been updated. For example, for some new devices with increased current ($I_\varepsilon > I_0$) with the applied strain, the resistance change ratio can be expressed as

$$\frac{R_\varepsilon - R_0}{R_0} = \frac{\frac{V}{I_\varepsilon} - \frac{V}{I_0}}{\frac{V}{I_0}} = \frac{I_0 - I_\varepsilon}{I_\varepsilon} = \frac{I_0}{I_\varepsilon} - 1 \tag{31}$$

As a result, the better the performance of the device, the smaller I_0/I_ε (tends to 0), and more strangely, the closer the resistance change ratio is to -1! So, the definition of gauge factor (formula (29)) is not suitable for the device with a new principle in some cases.

(2) Definition of gauge factor in devices based on piezotronic effect

For piezotronic devices, the strain-induced modulation of electrical transport by piezotronic effect is an asymmetric effect. As shown in **Supplementary Fig. 35**, under

the action of a tensile strain ($\Delta\varepsilon > 0$), the resistance of the device will gradually decrease under forward bias, and will gradually increases under reverse bias. In order to ensure that both the gauge factor values calculated under forward and reverse bias are positive and satisfy the rule that the higher gauge factor the better the performance, the current change ratio to the strain is utilized as gauge factor for the case when the current increases with strain:

$$gauge\ factor = \frac{\Delta I/I_0}{\Delta\varepsilon} \tag{32}$$

in which I_0 is the original current and ΔI is the change in current.

And, the resistance change ratio to the strain is utilized as gauge factor for the case when the current decreases with strain:

$$gauge\ factor = \frac{\Delta R/R_0}{\Delta\varepsilon} \tag{33}$$

Only by defining gauge factor in this way (formula (32) and formula (33)) can gauge factor characterize the strain sensing performance of piezotronic devices similar to those based on the traditional piezoresistive effect.

Supplementary Note 17 | Comparison of DPT with some other sensors

In order to exhibit the positions of the DPT and compare its performance with other sensors (including piezoresistive and capacitive sensors), we summarized performances of many sensors in **Supplementary Table 4-6** and **Supplementary Fig. 37**.

In general, based on different sensing mechanisms, parameters used to show the performance of pressure and strain sensors with different principle have different definitions. For example, piezotronic effect utilizes strain/force-induced piezoelectric polarization at interface to modulate the interface barrier height thus to control the electrical transport. As a result, a parameter defined as 'pressure sensitivity' (formula (34)) is applied to characterize the ability of mechanical stimuli on modulating the barrier height, and possesses a unit of eV/MPa. This parameter is only applicable to sensors with interface barrier modulated by strain/force. In addition, lots of sensors in literatures can only be used to sense strain or sense force, and usually characterized by different parameters, such as 'gauge factor' (with a unit of 1, defined as following formula (35)) and 'sensitivity' (with a unit of Pa^{-1}, formula (36)).

$$Pressure\ sensitivity = \frac{\Delta BH}{\Delta P} \tag{34}$$

in which ΔP is the change in pressure, and ΔBH is the barrier height change caused by ΔP.

$$Sensitivity = \frac{\Delta I/I_0}{\Delta P}\ or\ \frac{\Delta C/C_0}{\Delta P}\ or\ \frac{\Delta R/R_0}{\Delta P} \tag{35}$$

in which ΔP is the change in pressure, and ΔI, ΔC, ΔR are respectively the change in current, capacitor and resistance caused by ΔP, and I_0, C_0, R_0 are respectively the original current, capacitor and resistance.

$$Gauge\ factor = \frac{\Delta I/I_0}{\Delta \varepsilon}\ or\ \frac{\Delta R/R_0}{\Delta \varepsilon} \tag{36}$$

in which $\Delta \varepsilon$ is the change in strain, and ΔI, ΔR are respectively the change in current and resistance caused by $\Delta \varepsilon$, and I_0, R_0 are respectively the original current and resistance.

(1) Force sensing performance (pressure sensitivity and sensitivity)

Supplementary Fig. 37a gives the pressure sensitivity of DPT in comparison with previous piezotronic transistors in sensing force/pressure. The red-labeled data points are piezotronic transistors made of nano materials, and the blue-labeled data points are piezotronic transistors made of bulk materials. As can be seen, the ZnO nano/microwire-based DPT shows a pressure sensitivity of about 1.759 eV/MPa, which is a very high value in piezotronics.

Supplementary Fig. 37b shows the sensitivity of DPT in comparison with some other piezotronic sensors, piezoresistive sensors and capacitive sensors in last several years in sensing force/pressure. We can find that the sensitivity of DPT is also a high value in various sensors based on other force/pressure sensing mechanism.

(2) Strain sensing performance (pressure sensitivity and sensitivity)

Supplementary Fig. 37c and **Supplementary Fig. 37d** exhibit the gauge factor of DPT in comparison with some other piezotronic sensors, piezoresistive sensors and capacitive sensors in sensing strain. The highest gauge factor of DPT is about 8988 at a compressive strain of 5.8%, which is a high value in strain sensing.

In summary, **Supplementary Fig. 37** exhibits the positions of DPT in vertical force sensing and lateral strain sensing, which indicates the advantages of DPT with two working modes.

Supplementary Table 6 | Comparison of strain sensing works

Materials	Morphology	Type	GF	Work range	Reference
ZnO	MW, single	Piezotronic	~8988.6	-5.8%~0%	This work
ZnO	NW, single	Piezotronic	~1250	-0.8%~1.2%	[69]
ZnO	NW, single	Piezotronic	~400	0%~5%	[72]
ZnO	MW, single	Piezotronic	~1010	0%~1.14%	[73]
ZnO	NW, single	Piezotronic	~1832	-0.53%~1.31%	[74]
ZnO	NW, single	Piezotronic	~660	-0.64%~0.80%	[75]
ZnO	NW, single	Piezotronic	~437	0%~0.96%	[76]
Ag/HfO_2/ZnO	MW, single	Piezotronic	~4.8×10^5	0.00%~0.10%	[9]
ZnO	NW, array	Piezotronic	~1803	-0.15%~0%	[77]
ZnO	NW, array	Piezotronic	~1813	0%~0.8%	[78]
ZnO	NW, cluster	Piezotronic	~2×10^6	0~40 kPa	[4]
ZnO	NW, cluster	Piezotronic	~784	0~80.33 MPa	[50]
ZnO	Nanobelt	Piezotronic	~4036	-0.4%~0.3%	[79]
ZnO	Nanoplatelet	Piezotronic	~1.5×10^7	0.02~3.64 MPa	[8]
ZnO	Twin nanoplatelet	Piezotronic	2.9~9.4×10^9	24~153 kPa	[80]
ZnO	Nanoplatelet, bulk	Piezotronic	467~589	200~400 MPa	[81]
ZnO	Bulk	Piezotronic	~800	2~250 MPa	[44]
GaN	NW, single	Piezotronic	~1126	0.9~1.3 μN	[7]
Pt/Al_2O_3/GaN	Film	Piezotronic	~2.6×10^8	0~400 MPa	[82]
$ZnSnO_3$	MW, single	Piezotronic	~3740	0.00%~0.32%	[83]
CdSe	NW, single	Piezotronic	~1590	420~700 nN	[5]
InAs	NW, single	Piezotronic & Piezoresistive	~2820	0%~2.71%	[84]
ZnO	Nanobelt	Piezoresistive	~1166	-0.2%~0%	[85]
ZnO	NW, single	Piezoresistive	~14	-1.75%~1.75%	[86]

ZnO	NW, array	Piezoresistive	~7.64	0~6.2%	[87]
ZnO	Microparticle, film	Piezoresistive	>10^4	0%~0.82%, 0~10 kPa	[88]
MoS_2	Bilayer	Piezoresistive	56.5~72.5	-1.98%~1.98%, 0~200 kPa	[89]
SnS_2	Nanoflake	Piezoresistive	~3933	0%~1.25%	[90]
Ti_3C_2 MXene	Film	Piezoresistive	45.9~180.1	0.19%~2.13%, <13 kPa	[91]
SiC	NW, single	Piezoresistive	~6.9	0%~9.35%	[92]
3C-SiC/Si	Film	Piezoresistive	~5.8×10^4	0%~0.0677%	[93]
Si	NW, single	Piezoresistive	~5000	-0.163%~0%	[94]
Si	NW, single	Piezoresistive	~130	0%~3.5%	[95]
Si	Whisker	Piezoresistive	40~200	-6.7%~2.48%	[96]
Ge	NW, single	Piezoresistive	46.2~138.5	0%~2.1%	[97]
Au	NW, film	Piezoresistive	1.82~7.38	13 Pa~50 kPa	[98]
Pt	Nanocrack, film	Piezoresistive	~2000	0%~2%	[99]
Carbon	Nanotube	Piezoresistive	600~1000	0%~0.6%	[100]
Carbon	Nanotube	Piezoresistive	~2900	0%~0.5%	[101]
Carbon	Nanotube, film	Piezoresistive	0.06~0.82	0%~200%	[102]
Carbon	Nanotube, foam	Piezoresistive	0.464~2.63	1 Pa~2000 kPa	[103]
SWCNT/Graphite nanoplatelet, film		Piezoresistive	~8	0%~0.16%	[104]
PEDOT: PSS-SWCNT nanocomposite, film		Piezoresistive	~0.17	28 Pa~40 kPa	[105]
50 wt% Carbon Black/TPE, fibre		Piezoresistive	~20	0%~80%	[106]
Graphene	Ribbon	Piezoresistive	~1.9	0%~3%	[107]
Graphene	Ribbon	Piezoresistive	~2	0%~20%	[108]
Graphene	Film	Piezoresistive	~6.1	0%~1%	[109]
Graphene	Film	Piezoresistive	~300	-0.29%~0.37%	[110]
Graphene	Flake, film	Piezoresistive	~150	0.0%~1.7%	[111]

Graphene-Nanocellulose, nanopaper		Piezoresistive	1.6~7.1, 502~2427	10%~100%, 1%~6%	[112]
Pt-coated PUA	Nanofibre, array	Piezoresistive	~11.5	5~1500 Pa	[113]
Carbonized silk fabric		Piezoresistive	~9.6, ~37.5	0%~250%, 250%~500%	[114]
I_2@CuTCA MOF, nanofilm		Piezoresistive	~11200	2.5%~3.3%	[115]
DPP-TVT-PDCA/PDMS-PDCA-Fe, film		Piezoresistive	$\sim 5.75\times10^5$	0%~100%	[116]
Carbon fibre polymer composites (CFPC)		Piezoresistive	$\sim 8.5\times10^4$	0%~5%	[117]
PDMS/Ag NW/Ecoflex		Capacitive	~0.7	0%~50%	[67]
Conductive fabric/Ecoflex		Capacitive	0.83	0%~50%	[118]
Silver fiber-Cotton fiber, textile		Capacitive	0.695	0%~15%	[119]
Ag nanoparticle/spandex, fibre		Capacitive	~12	0%~40%	[120]
p-Si	Single crystal	Flexoelectronic	2650	3.51~21.08 μN	[121]
TiO_2	Single crystal	Flexoelectronic	~472	2.17-21.71 μN	[121]
Nb-$SrTiO_3$	Single crystal	Flexoelectronic	~183	3.26-14.98 μN	[121]
p-Si	Single crystal	Flexoelectronic	2189	0%~0.06%	[122]

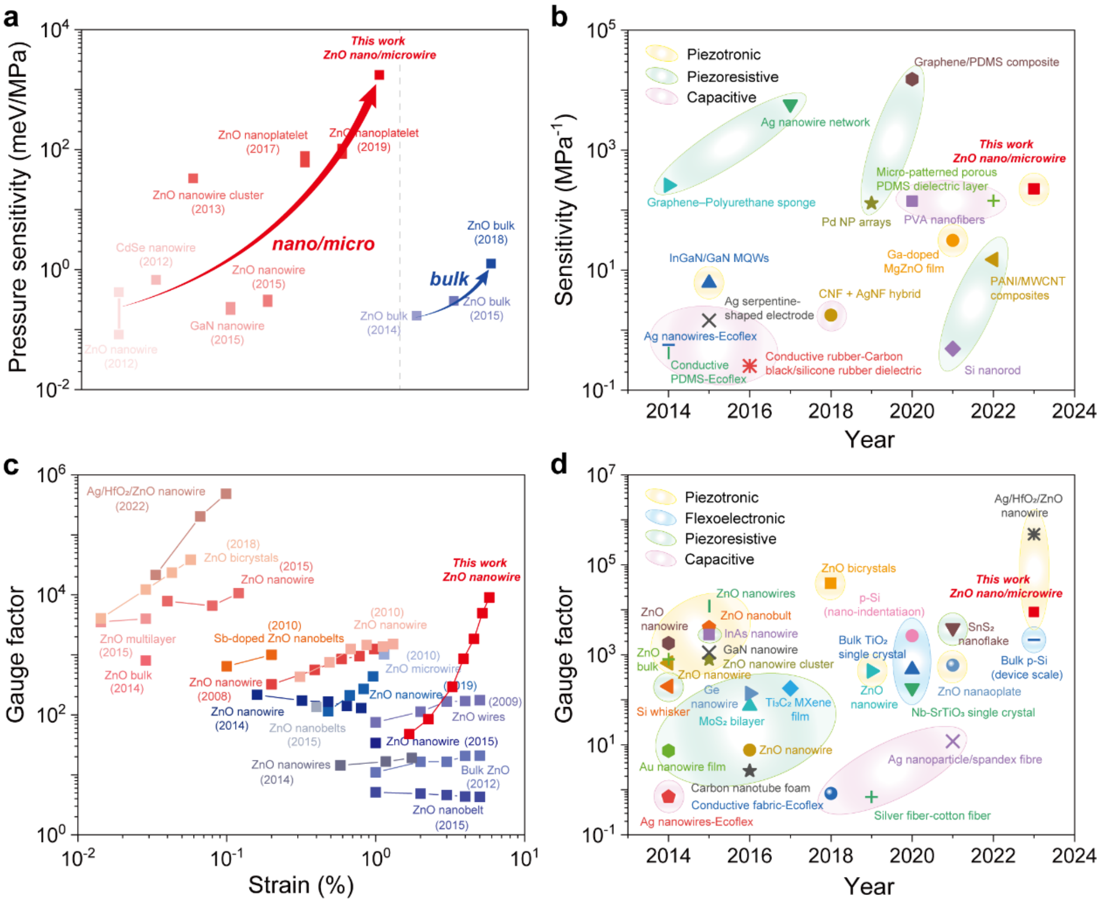

Supplementary Fig. 37 | Sensing performance of the DPT. a, Comparison of the pressure sensitivity of DPT with previous piezotronic transistors/sensors in vertical force sensing. [4-8, 44-46, 50, 53] **b**, Comparison of the pressure sensitivity of DPT in vertical force sensing with some existing piezotronic/piezoresistive/capacitive sensors. [54-68] **c** and **d**, Comparison of the gauge factor of DPT with some existing piezotronic/flexoelectronic/ piezoresistive/capacitive sensors in strain sensing as a function of the strain (**c**) [9, 44-46, 69, 72-76, 79, 85, 86, 123-126] and the published year (**d**) [7, 9, 44, 46, 50, 67, 75, 76, 78, 79, 81, 84, 87, 89-91, 96-98, 103, 118-122, 126].

Supplementary Note 18 | Definition of the on/off ratio

The on/off ratio is usually adopted to characterize sensors, which reflects the sensing performance of the device in response to the applied strain or force. The on/off ratio here is defined as the ratio of the current I_{on} in "ON" state (corresponding to the current after application of a strain or force) to the current I_{off} in "OFF" state (corresponding to the current before application of a strain or force), which can be written as

$$(on/off\ ratio)_{strain} = I_{\mathrm{on}}/I_{\mathrm{off}} = I_{after\ a\ strain}/I_{beforce\ a\ strain} \tag{37}$$

$$(on/off\ ratio)_{force} = I_{\mathrm{on}}/I_{\mathrm{off}} = I_{after\ a\ force}/I_{before\ a\ force} \tag{38}$$

Supplementary Note 19 | Crosstalk between two sensing signals in DPT and its possible solution

Since both the vertical force sensing and the lateral strain sensing utilize the deformation of ZnO nano/microwire to produce piezoelectric polarizations at interface to control the barrier height and electrical transport, we cannot distinguish in theory whether the deformation of ZnO nano/microwire is caused by the vertical force or the lateral strain. So, there exists a crosstalk between the force sensing signal and the strain sensing signal in DPT.

This work focuses on achieving a piezotronic transistor (sensor) with two sensing functionality that can switch between the two modes according to the needs of the scene, and improving the pressure sensitivity of vertical force sensing and possessing a good lateral force sensing performance. Besides this innovation, we believe that the dual-modal piezotronic transistor (DPT) will have a wider range of applications and a greater impact on the structural design of future piezotronic transistors if the two signals can be furtherly decoupled no matter by principle design or signal processing. This issue

about DPT will be a significant step in the next generation of piezotronic transistors.

At this stage we cannot solve the problem of crosstalk, but to our best understanding on piezotronic modification on energy band, we think the difference in the bending shapes of two sensing working modes (Note: In lateral strain sensing, the nano/microwire is bent or stretched and compressed as a whole; while in vertical force sensing, the middle of the nano/microwire bends downward.) is a possible breakthrough point. The different bending shapes means different piezoelectric potential distribution along the nano/microwire (**Supplementary Fig. 38**) and different energy band bending, which may cause different impedance information. Investigating the piezotronic modification of the capacitance-voltage characteristics may give us possible approach to distinguish the nano/microwire bending shapes, and decouple the vertical force and the lateral strain sensing signals by technologies like behavior-learned cognition algorithm [127]. However, its complexity needs a lot of in-depth works in the further.

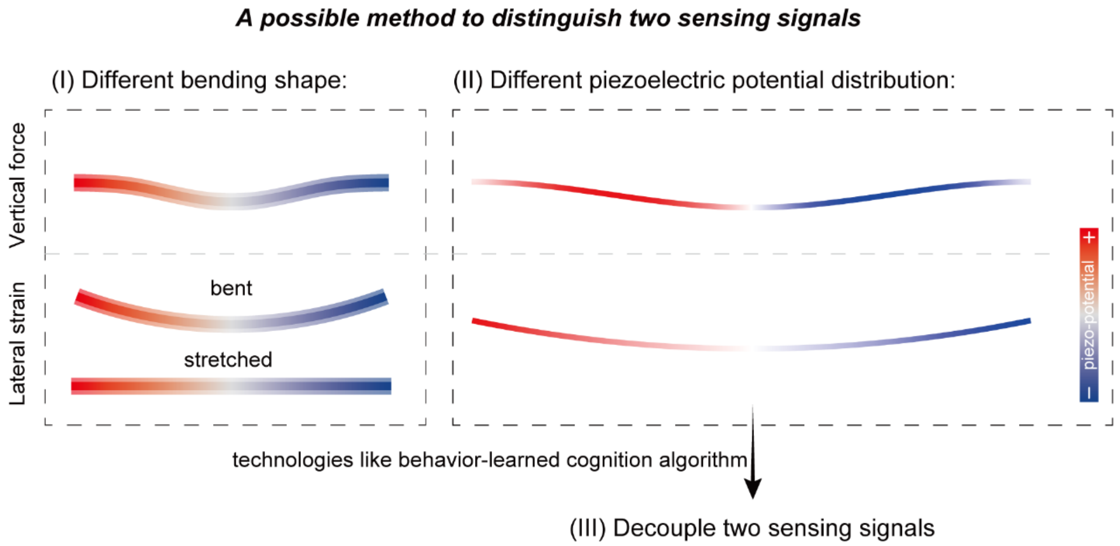

Supplementary Fig. 38 | A possible method to solve the problem of crosstalk in DPT. Different bending shape (I) will make the piezoelectric potential distribution (II) different, which may lead to different impedance (III). Through investigating the piezotronic modification of the capacitance-voltage characteristics may we obtain the possible approach to decouple the two sensing signals by technologies like behavior-learned cognition algorithm.

Supplementary References

1. Zhang, Y., Liu, Y. & Wang, Z. L. Fundamental theory of piezotronics. *Advanced Materials* **23**, 3004-3013 (2011).
2. Pan, C., Zhai, J. & Wang, Z. L. Piezotronics and piezo-phototronics of third generation semiconductor nanowires. *Chemical Reviews* **119**, 9303-9359 (2019).
3. Wu, W. & Wang, Z. L. Piezotronics and piezo-phototronics for adaptive electronics and optoelectronics. *Nature Reviews Materials* **1**, 16031 (2016).
4. Wu, W., Wen, X. & Wang, Z. L. Taxel-addressable matrix of vertical-nanowire piezotronic transistors for active and adaptive tactile imaging. *Science* **340**, 952-957 (2013).
5. Zhou, Y. S. *et al.* Vertically aligned CdSe nanowire arrays for energy harvesting and piezotronic devices. *ACS Nano* **6**, 6478-6482 (2012).
6. Han, W. *et al.* Strain-gated piezotronic transistors based on vertical zinc oxide nanowires. *ACS Nano* **6**, 3760-3766 (2012).
7. Zhao, Z. *et al.* Piezotronic effect in polarity-controlled GaN nanowires. *ACS Nano* **9**, 8578-8583 (2015).
8. Liu, S. *et al.* Ultrasensitive 2D ZnO piezotronic transistor array for high resolution tactile imaging. *Advanced Materials* **29**, 1606346 (2017).
9. Yu, Q. *et al.* Highly sensitive strain sensors based on piezotronic tunneling junction. *Nature Communications* **13**, 778 (2022).
10. Wang, L. & Wang, Z. L. Advances in piezotronic transistors and piezotronics. *Nano Today* **37**, 101108 (2021).
11. Crisler, D. F., Cupal, J. J. & Moore, A. R. Dielectric, piezoelectric, and electromechanical coupling constants of zinc oxide crystals. *Proceedings of the IEEE* **56**, 225-226 (1968).
12. Zhao, M.-H., Wang, Z.-L. & Mao, S. X. Piezoelectric characterization of individual zinc oxide nanobelt probed by piezoresponse force microscope. *Nano Letters* **4**, 587-590 (2004).
13. Fu, J. Y., Liu, P. Y., Cheng, J., Bhalla, A. S. & Guo, R. Optical measurement of the converse piezoelectric d_{33} coefficients of bulk and microtubular zinc oxide crystals.

Applied Physics Letters **90**, 212907 (2007).

14. Fan, H. J. *et al.* Template-assisted large-scale ordered arrays of ZnO pillars for optical and piezoelectric applications. *Small* **2**, 561-568 (2006).
15. Scrymgeour, D. A., Sounart, T. L., Simmons, N. C. & Hsu, J. W. P. Polarity and piezoelectric response of solution grown zinc oxide nanocrystals on silver. *Journal of Applied Physics* **101**, 014316 (2007).
16. Scrymgeour, D. A. & Hsu, J. W. P. Correlated piezoelectric and electrical properties in individual ZnO nanorods. *Nano Letters* **8**, 2204-2209 (2008).
17. Wang, L. *et al.* Ultrathin piezotronic transistors with 2 nm channel lengths. *ACS Nano* **12**, 4903-4908 (2018).
18. Lueng, C. M., Chan, H. L. W., Surya, C. & Choy, C. L. Piezoelectric coefficient of aluminum nitride and gallium nitride. *Journal of Applied Physics* **88**, 5360-5363 (2000).
19. Muensit, S. & Guy, I. L. The piezoelectric coefficient of gallium nitride thin films. *Applied Physics Letters* **72**, 1896-1898 (1998).
20. Minary-Jolandan, M., Bernal, R. A., Kuljanishvili, I., Parpoil, V. & Espinosa, H. D. Individual GaN nanowires exhibit strong piezoelectricity in 3D. *Nano Letters* **12**, 970-976 (2012).
21. Kobiakov, I. B. Elastic, piezoelectric and dielectric properties of ZnO and CdS single crystals in a wide range of temperatures. *Solid State Communications* **35**, 305-310 (1980).
22. Wang, X. *et al.* Subatomic deformation driven by vertical piezoelectricity from CdS ultrathin films. *Science Advances* **2**, e1600209 (2016).
23. Brennan, C. J., Ghosh, R., Koul, K., Banerjee, S. K., Lu, N. & Yu, E. T. Out-of-plane electromechanical response of monolayer molybdenum disulfide measured by piezoresponse force microscopy. *Nano Letters* **17**, 5464-5471 (2017).
24. Syed, N. *et al.* Printing two-dimensional gallium phosphate out of liquid metal. *Nature Communications* **9**, 3618 (2018).
25. Xue, F. *et al.* Multidirection piezoelectricity in mono- and multilayered hexagonal α-In_2Se_3. *ACS Nano* **12**, 4976-4983 (2018).

26. Ke, T.-Y. *et al.* Sodium niobate nanowire and its piezoelectricity. *The Journal of Physical Chemistry C* **112**, 8827-8831 (2008).

27. Wang, J., Stampfer, C., Roman, C., Ma, W. H., Setter, N. & Hierold, C. Piezoresponse force microscopy on doubly clamped $KNbO_3$ nanowires. *Applied Physics Letters* **93**, 223101 (2008).

28. Nguyen, T. D. *et al.* Wafer-scale nanopatterning and translation into high-performance piezoelectric nanowires. *Nano Letters* **10**, 4595-4599 (2010).

29. You, Y.-M. *et al.* An organic-inorganic perovskite ferroelectric with large piezoelectric response. *Science* **357**, 306-309 (2017).

30. Wang, Z. L. Nanopiezotronics. *Advanced Materials* **19**, 889-892 (2007).

31. Yang, R., Qin, Y., Dai, L. & Wang, Z. L. Power generation with laterally packaged piezoelectric fine wires. *Nature Nanotechnology* **4**, 34-39 (2009).

32. Feng, R. & Farris, R. J. Linear thermoelastic characterization of anisotropic poly(ethylene terephthalate) films. *Journal of Applied Polymer Science* **86**, 2937-2947 (2002).

33. Wen, X., Li, D., Tan, K., Deng, Q. & Shen, S. Flexoelectret: an electret with a tunable flexoelectriclike response. *Physical Review Letters* **122**, 148001 (2019).

34. Su, Y.-L., Gupta, K., Hsiao, Y.-L., Wang, R.-C. & Liu, C.-P. Gigantic enhancement of electricity generation in piezoelectric semiconductors by creating pores as a universal approach. *Energy & Environmental Science* **12**, 410-417 (2019).

35. Teixeira, I. *et al.* Polydimethylsiloxane mechanical properties: a systematic review. *AIMS Materials Science* **8**, 952-973 (2021).

36. Moučka, R., Sedlačík, M., Osička, J. & Pata, V. Mechanical properties of bulk Sylgard 184 and its extension with silicone oil. *Scientific Reports* **11**, 19090 (2021).

37. Peng, J., Tomsia, A. P., Jiang, L., Tang, B. Z. & Cheng, Q. Stiff and tough PDMS-MMT layered nanocomposites visualized by AIE luminogens. *Nature Communications* **12**, 4539 (2021).

38. Palchesko, R. N., Zhang, L., Sun, Y. & Feinberg, A. W. Development of polydimethylsiloxane substrates with tunable elastic modulus to study cell mechanobiology in muscle and nerve. *PLoS ONE* **7**, e51499 (2012).

39. Wang, Z., Xiang, C., Yao, X., Le Floch, P., Mendez, J. & Suo, Z. Stretchable materials of high toughness and low hysteresis. *Proceedings of the National Academy of Sciences* **116**, 5967-5972 (2019).
40. Wang, Z. Polydimethylsiloxane mechanical properties measured by macroscopic compression and nanoindentation techniques. **Master's Thesis**, University of South Florida (2011).
41. Zhang, S. L. & Li, J. C. M. Anisotropic elastic moduli and Poisson's ratios of a poly(ethylene terephthalate) film. *Journal of Polymer Science Part B: Polymer Physics* **42**, 260-266 (2004).
42. Sze, S. M. & Ng, K. K. Physics of semiconductor devices. 3rd Edition (Wiley, 2006).
43. Zhou, J. *et al.* Piezoelectric-potential-controlled polarity-reversible Schottky diodes and switches of ZnO wires. *Nano Letters* **8**, 3973-3977 (2008).
44. Baraki, R., Novak, N., Frömling, T., Granzow, T. & Rödel, J. Bulk ZnO as piezotronic pressure sensor. *Applied Physics Letters* **105**, 111604 (2014).
45. Raidl, N., Supancic, P., Danzer, R. & Hofstätter, M. Piezotronically modified double Schottky barriers in ZnO varistors. *Advanced Materials* **27**, 2031-2035 (2015).
46. Keil, P., Trapp, M., Novak, N., Frömling, T., Kleebe, H.-J. & Rödel, J. Piezotronic tuning of potential barriers in ZnO bicrystals. *Advanced Materials* **30**, 1705573 (2018).
47. Jiang, C. *et al.* 60 nm pixel-size pressure piezo-memory system as ultrahigh-resolution neuromorphic tactile sensor for in-chip computing. *Nano Energy* **87**, 106190 (2021).
48. Han, X. *et al.* A two-terminal optoelectronic synapses array based on the $ZnO/Al_2O_3/CdS$ heterojunction with strain-modulated synaptic weight. *Advanced Electronic Materials* **9**, 2201068 (2023).
49. Park, J. *et al.* Individually addressable and flexible pressure sensor matrixes with ZnO nanotube arrays on graphene. *NPG Asia Materials* **14**, 40 (2022).
50. Han, X., Du, W., Yu, R., Pan, C. & Wang, Z. L. Piezo-phototronic enhanced UV

sensing based on a nanowire photodetector array. *Advanced Materials* **27**, 7963-7969 (2015).

51. Wang, L. *et al.* 2D piezotronics in atomically thin zinc oxide sheets: interfacing gating and channel width gating. *Nano Energy* **60**, 724-733 (2019).
52. An, C. *et al.* Piezotronic and piezo-phototronic effects of atomically-thin ZnO nanosheets. *Nano Energy* **82**, 105653 (2021).
53. Liu, S. *et al.* Double-channel piezotronic transistors for highly sensitive pressure sensing. *ACS Nano* **12**, 1732-1738 (2018).
54. Lee, P. H., Brahma, S., Dutta, J., Huang, J.-L. & Liu, C.-P. Synergistic effects of Ga doping and Mg alloying over the enhancement of the stress sensitivity of a Ga-doped MgZnO pressure sensor. *Nanoscale Advances* **3**, 3909-3917 (2021).
55. Peng, M. *et al.* High-resolution dynamic pressure sensor array based on piezo-phototronic effect tuned photoluminescence imaging. *ACS Nano* **9**, 3143-3150 (2015).
56. Wei, Y., Chen, S., Dong, X., Lin, Y. & Liu, L. Flexible piezoresistive sensors based on “dynamic bridging effect” of silver nanowires toward graphene. *Carbon* **113**, 395-403 (2017).
57. Ghosh, R. *et al.* Fabrication of piezoresistive Si nanorod-based pressure sensor arrays: a promising candidate for portable breath monitoring devices. *Nano Energy* **80**, 105537 (2021).
58. Arias-Ferreiro, G. *et al.* Flexible 3D printed acrylic composites based on polyaniline/multiwalled carbon nanotubes for piezoresistive pressure sensors. *Advanced Electronic Materials* **8**, 2200590 (2022).
59. Pan, L. *et al.* An ultra-sensitive resistive pressure sensor based on hollow-sphere microstructure induced elasticity in conducting polymer film. *Nature Communications* **5**, 3002 (2014).
60. Wang, Y., Wu, H., Xu, L., Zhang, H., Yang, Y. & Wang, Z. L. Hierarchically patterned self-powered sensors for multifunctional tactile sensing. *Science Advances* **6**, eabb9083 (2020).
61. Chen, M. *et al.* An ultrahigh resolution pressure sensor based on percolative metal

nanoparticle arrays. *Nature Communications* **10**, 4024 (2019).

62. Lee, S. *et al.* Nanomesh pressure sensor for monitoring finger manipulation without sensory interference. *Science* **370**, 966-970 (2020).
63. Wang, J., Li, L., Zhang, L., Zhang, P. & Pu, X. Flexible capacitive pressure sensors with micro-patterned porous dielectric layer for wearable electronics. *Journal of Micromechanics and Microengineering* **32**, 034003 (2022).
64. An, B. W., Heo, S., Ji, S., Bien, F. & Park, J.-U. Transparent and flexible fingerprint sensor array with multiplexed detection of tactile pressure and skin temperature. *Nature Communications* **9**, 2458 (2018).
65. Zhao, X., Hua, Q., Yu, R., Zhang, Y. & Pan, C. Flexible, stretchable and wearable multifunctional sensor array as artificial electronic skin for static and dynamic strain mapping. *Advanced Electronic Materials* **1**, 1500142 (2015).
66. Guo, X., Huang, Y., Cai, X., Liu, C. & Liu, P. Capacitive wearable tactile sensor based on smart textile substrate with carbon black/silicone rubber composite dielectric. *Measurement Science and Technology* **27**, 045105 (2016).
67. Yao, S. & Zhu, Y. Wearable multifunctional sensors using printed stretchable conductors made of silver nanowires. *Nanoscale* **6**, 2345-2352 (2014).
68. Woo, S.-J., Kong, J.-H., Kim, D.-G. & Kim, J.-M. A thin all-elastomeric capacitive pressure sensor array based on micro-contact printed elastic conductors. *Journal of Materials Chemistry C* **2**, 4415-4422 (2014).
69. Zhou, J. *et al.* Flexible piezotronic strain sensor. *Nano Letters* **8**, 3035-3040 (2008).
70. Liu, K., Sakurai, M. & Aono, M. Enhancing the humidity sensitivity of Ga_2O_3/SnO_2 core/shell microribbon by applying mechanical strain and its application as a flexible strain sensor. *Small* **8**, 3599-3604 (2012).
71. Tavassolian, M., Cuthbert, T. J., Napier, C., Peng, J. & Menon, C. Textile-based inductive soft strain sensors for fast frequency movement and their application in wearable devices measuring multiaxial hip joint angles during running. *Advanced Intelligent Systems* **2**, 1900165 (2020).
72. Yang, Y., Qi, J. J., Gu, Y. S., Wang, X. Q. & Zhang, Y. Piezotronic strain sensor based on single bridged ZnO wires. *physica status solidi (RRL) – Rapid Research*

Letters **3**, 269-271 (2009).

73. Sun, K. *et al.* Growth of ultralong ZnO microwire and its application in isolatable and flexible piezoelectric strain sensor. *physica status solidi (a)* **207**, 488-492 (2010).
74. Wu, W., Wei, Y. & Wang, Z. L. Strain-gated piezotronic logic nanodevices. *Advanced Materials* **22**, 4711-4715 (2010).
75. Wang, P. *et al.* Asymmetric behavior in flexible piezoelectric strain sensors made of single ZnO nanowires. *Journal of nanoscience and nanotechnology* **14**, 6084-6088 (2014).
76. Yang, X. *et al.* Coupled ion-gel channel-width gating and piezotronic interface gating in ZnO nanowire devices. *Advanced Functional Materials* **29**, 1807837 (2019).
77. Pan, C. *et al.* High-resolution electroluminescent imaging of pressure distribution using a piezoelectric nanowire LED array. *Nature Photonics* **7**, 752-758 (2013).
78. Zhang, W., Zhu, R., Nguyen, V. & Yang, R. Highly sensitive and flexible strain sensors based on vertical zinc oxide nanowire arrays. *Sensors and Actuators A: Physical* **205**, 164-169 (2014).
79. Zhang, Z. *et al.* Highly efficient piezotronic strain sensors with symmetrical Schottky contacts on the monopolar surface of ZnO nanobelts. *Nanoscale* **7**, 1796-1801 (2015).
80. Wang, L. *et al.* Ultrasensitive vertical piezotronic transistor based on ZnO twin nanoplatelet. *ACS Nano* **11**, 4859-4865 (2017).
81. Liu, S. *et al.* Statistical piezotronic effect in nanocrystal bulk by anisotropic geometry control. *Advanced Functional Materials* **31**, 2010339 (2021).
82. Liu, S., Wang, L., Feng, X., Liu, J., Qin, Y. & Wang, Z. L. Piezotronic tunneling junction gated by mechanical stimuli. *Advanced Materials* **31**, 1905436 (2019).
83. Wu, J. M. *et al.* Ultrahigh sensitive piezotronic strain sensors based on a $ZnSnO_3$ nanowire/microwire. *ACS Nano* **6**, 4369-4374 (2012).
84. Li, X., Wei, X., Xu, T., Pan, D., Zhao, J. & Chen, Q. Remarkable and crystal-structure-dependent piezoelectric and piezoresistive effects of InAs nanowires.

Advanced Materials **27**, 2852-2858 (2015).

85. Yang, Y., Guo, W., Qi, J. & Zhang, Y. Flexible piezoresistive strain sensor based on single Sb-doped ZnO nanobelts. *Applied Physics Letters* **97**, 223107 (2010).
86. Shao, R. W. *et al.* Bandgap engineering and manipulating electronic and optical properties of ZnO nanowires by uniaxial strain. *Nanoscale* **6**, 4936-4941 (2014).
87. Lee, T., Lee, W., Kim, S.-W., Kim, J. J. & Kim, B.-S. Flexible textile strain wireless sensor functionalized with hybrid carbon nanomaterials supported ZnO nanowires with controlled aspect ratio. *Advanced Functional Materials* **26**, 6206-6214 (2016).
88. Yin, B., Liu, X., Gao, H., Fu, T. & Yao, J. Bioinspired and bristled microparticles for ultrasensitive pressure and strain sensors. *Nature Communications* **9**, 5161 (2018).
89. Park, M., Park, Y. J., Chen, X., Park, Y. K., Kim, M. S. & Ahn, J. H. MoS_2-based tactile sensor for electronic skin applications. *Advanced Materials* **28**, 2556-2562 (2016).
90. Yan, W. *et al.* Giant gauge factor of Van der Waals material based strain sensors. *Nature Communications* **12**, 2018 (2021).
91. Ma, Y. *et al.* A highly flexible and sensitive piezoresistive sensor based on MXene with greatly changed interlayer distances. *Nature Communications* **8**, 1207 (2017).
92. Shao, R., Zheng, K., Zhang, Y., Li, Y., Zhang, Z. & Han, X. Piezoresistance behaviors of ultra-strained SiC nanowires. *Applied Physics Letters* **101**, 233109 (2012).
93. Nguyen, T. *et al.* Giant piezoresistive effect by optoelectronic coupling in a heterojunction. *Nature Communications* **10**, 4139 (2019).
94. Neuzil, P., Wong, C. C. & Reboud, J. Electrically controlled giant piezoresistance in silicon nanowires. *Nano Letters* **10**, 1248-1252 (2010).
95. Lugstein, A., Steinmair, M., Steiger, A., Kosina, H. & Bertagnolli, E. Anomalous piezoresistance effect in ultrastrained silicon nanowires. *Nano Letters* **10**, 3204-3208 (2010).
96. Zheng, K. *et al.* Observation of enhanced carrier transport properties of Si <100>-

oriented whiskers under uniaxial strains. *Applied Physics Letters* **104**, 013111 (2014).

97. Greil, J., Lugstein, A., Zeiner, C., Strasser, G. & Bertagnolli, E. Tuning the electro-optical properties of germanium nanowires by tensile strain. *Nano Letters* **12**, 6230-6234 (2012).
98. Gong, S. *et al.* A wearable and highly sensitive pressure sensor with ultrathin gold nanowires. *Nature Communications* **5**, 3132 (2014).
99. Kang, D. *et al.* Ultrasensitive mechanical crack-based sensor inspired by the spider sensory system. *Nature* **516**, 222-226 (2014).
100. Cao, J., Wang, Q. & Dai, H. Electromechanical properties of metallic, quasimetallic, and semiconducting carbon nanotubes under stretching. *Physical Review Letters* **90**, 157601 (2003).
101. Stampfer, C., Jungen, A., Linderman, R., Obergfell, D., Roth, S. & Hierold, C. Nano-electromechanical displacement sensing based on single-walled carbon nanotubes. *Nano Letters* **6**, 1449-1453 (2006).
102. Yamada, T. *et al.* A stretchable carbon nanotube strain sensor for human-motion detection. *Nature Nanotechnology* **6**, 296-301 (2011).
103. Li, Y., Luo, S., Yang, M.-C., Liang, R. & Zeng, C. Poisson ratio and piezoresistive sensing: a new route to high-performance 3D flexible and stretchable sensors of multimodal sensing capability. *Advanced Functional Materials* **26**, 2900-2908 (2016).
104. Luo, S. & Liu, T. SWCNT/graphite nanoplatelet hybrid thin films for self-temperature-compensated, highly sensitive, and extensible piezoresistive sensors. *Advanced Materials* **25**, 5650-5657 (2013).
105. Roh, E., Lee, H. B., Kim, D. I. & Lee, N. E. A solution-processable, omnidirectionally stretchable, and high-pressure-sensitive piezoresistive device. *Advanced Materials* **29**, 1703004 (2017).
106. Mattmann, C., Clemens, F. & Tröster, G. Sensor for measuring strain in textile. *Sensors* **8**, 3719-3732 (2008).
107. Huang, M., Pascal, T. A., Kim, H., Goddard, W. A., III & Greer, J. R. Electronic-

mechanical coupling in graphene from in situ nanoindentation experiments and multiscale atomistic simulations. *Nano Letters* **11**, 1241-1246 (2011).

108. Wang, Y. *et al.* Super-elastic graphene ripples for flexible strain sensors. *ACS Nano* **5**, 3645-3650 (2011).
109. Lee, Y. *et al.* Wafer-scale synthesis and transfer of graphene films. *Nano Letters* **10**, 490-493 (2010).
110. Zhao, J. *et al.* Ultra-sensitive strain sensors based on piezoresistive nanographene films. *Applied Physics Letters* **101**, 063112 (2012).
111. Hempel, M., Nezich, D., Kong, J. & Hofmann, M. A novel class of strain gauges based on layered percolative films of 2D materials. *Nano Letters* **12**, 5714-5718 (2012).
112. Yan, C. *et al.* Highly stretchable piezoresistive graphene-nanocellulose nanopaper for strain sensors. *Advanced Materials* **26**, 2022-2027 (2014).
113. Pang, C. *et al.* A flexible and highly sensitive strain-gauge sensor using reversible interlocking of nanofibres. *Nature Materials* **11**, 795-801 (2012).
114. Wang, C. *et al.* Carbonized silk fabric for ultrastretchable, highly sensitive, and wearable strain sensors. *Advanced Materials* **28**, 6640-6648 (2016).
115. Pan, L. *et al.* Mechano-regulated metal-organic framework nanofilm for ultrasensitive and anti-jamming strain sensing. *Nature Communications* **9**, 3813 (2018).
116. Oh, J. Y. *et al.* Stretchable self-healable semiconducting polymer film for active-matrix strain-sensing array. *Science Advances* **5**, eaav3097 (2019).
117. Araromi, O. A. *et al.* Ultra-sensitive and resilient compliant strain gauges for soft machines. *Nature* **587**, 219-224 (2020).
118. Atalay, O. Textile-based, interdigital, capacitive, soft-strain sensor for wearable applications. *Materials* **11**, 768 (2018).
119. Zhang, Q., Wang, Y. L., Xia, Y., Zhang, P. F., Kirk, T. V. & Chen, X. D. Textile-only capacitive sensors for facile fabric integration without compromise of wearability. *Advanced Materials Technologies* **4**, 1900485 (2019).
120. Lee, J. *et al.* Stretchable and suturable fibre sensors for wireless monitoring of

connective tissue strain. *Nature Electronics* **4**, 291-301 (2021).

121. Wang, L. *et al.* Flexoelectronics of centrosymmetric semiconductors. *Nature Nanotechnology* **15**, 661-667 (2020).
122. Guo, D. *et al.* Silicon flexoelectronic transistors. *Science Advances* **9**, eadd3310 (2023).
123. Sun, X., Gu, Y., Wang, X., Zhang, Z. & Zhang, Y. Strain-modulated transport properties of Cu/ZnO-nanobelt/Cu nanojunctions. *physica status solidi (b)* **252**, 1767-1772 (2015).
124. Zhang, G., Luo, X., Zheng, Y. & Wang, B. Giant piezoelectric resistance effect of nanoscale zinc oxide tunnel junctions: first principles simulations. *Physical Chemistry Chemical Physics* **14**, 7051-7058 (2012).
125. Nakamura, K. First-principles simulation on wire diameter dependence of piezoresistivity in zinc oxide nanowires. *Japanese Journal of Applied Physics* **54**, 06FJ11 (2015).
126. Xue, F. *et al.* Influence of external electric field on piezotronic effect in ZnO nanowires. *Nano Research* **8**, 2390-2399 (2015).
127. Lee, J. H. *et al.* Heterogeneous structure omnidirectional strain sensor arrays with cognitively learned neural networks. *Advanced Materials* **35**, 2208184 (2023).
