# Supplementary material for: Dual-modal piezotronic transistor for highly sensitive vertical force sensing and lateral strain sensing
Source: Nat Commun. 2023 Oct 9;14:6315. doi: 10.1038/s41467-023-41983-3 (PMC10562489; doi:10.1038/s41467-023-41983-3)
Supplement: Supplementary file 2 — Peer Review File [file 41467_2023_41983_MOESM2_ESM.pdf]

nature portfolio

Peer Review File

Dual-modal piezotronic transistor for highly sensitive vertical force sensing and lateral strain sensing

Open Access This file is licensed under a Creative Commons Attribution 4.0 International License, which permits use, sharing, adaptation, distribution and reproduction in any medium or format, as long as you give appropriate credit to the original author(s) and the source, provide a link to the Creative Commons license, and indicate if changes were made. In the cases where the authors are anonymous, such as is the case for the reports of anonymous peer reviewers, author attribution should be to 'Anonymous Referee' followed by a clear attribution to the source work. The images or other third party material in this file are included in the article's Creative Commons license, unless indicated otherwise in a credit line to the material. If material is not included in the article's Creative Commons license and your intended use is not permitted by statutory regulation or exceeds the permitted use, you will need to obtain permission directly from the copyright holder. To view a copy of this license, visit http://creativecommons.org/licenses/by/4.0/.

REVIEWER COMMENTS

Reviewer #1 (Remarks to the Author):

The manuscript presents a novel dual-modal piezotronic transistor (DPT) that combines ZnO nanowires and PDMS with microprotrusions for highly sensitive force sensing. The electrical properties of the DPT are evaluated for both vertical force sensing and lateral strain sensing after structural optimization. While the DPT demonstrates high sensitivity in detecting small forces, the overall concept of the DPT is not particularly innovative and requires further validation and analysis. As such, based on the above comments, it is difficult to recommend the manuscript for publication at this time.

Comments:

1.The objective of this study is to develop a dual-modal piezotronic transistor; however, the structural optimization of the DPT lacks sufficient data and descriptions. For instance, the detailed parameters and schematic representation of the microprotrusion were not provided. The relationship between microprotrusion width and nanowire deformation under the same external force did not be investigated, and the impact of materials such as PDMS and PET on the nanowire deformation was not discussed, either. This leads to the most fundamental factor affecting sensitivity in the DPT structure not clear.

2.There have been many reports of multidirectional, high-sensitivity strain/pressure sensors in recent years. This work is based on the more established piezoelectric transistor principle to detect small stresses in multiple directions. Although the manuscript demonstrates the performance of DPT as a sensor in different modes, it appears to be an incremental improvement in sensitivity compared to existing work. However, this improvement alone may not guarantee publication in Nature Communications.

3.The author presents pressure-dependent simulations along with experimental data to validate them. However, in order for the data to be considered valid, it is essential to demonstrate multiple data points and their corresponding errors especially in sensor research, where accuracy is critical. As the author has not presented enough data points, it is difficult to evaluate the validity of the findings. Therefore, it is recommended that the author includes additional data points in the manuscript to support their conclusions.

Reviewer #2 (Remarks to the Author):

Comments on NCOMMS-23-11350 titled Dual-modal piezotronic transistor for highly sensitive vertical force sensing and lateral strain sensing

The authors reported a force/strain sensor based on a single ZnO nano/microwire metal-piezoelectric semiconductor-metal structure. By innovatively introduce a micro-protrusion structure, the sensor could respond to both vertical force and lateral strain. The force sensitivity is up to 221.5 N^-1 and the gauge factor is up to 7598.2. The idea is really interesting and publication in Nature Communications could be considered if the authors address the following comments.

1. In the Introduction part, the authors state that “the use of the force/strain-induced piezoelectric polarization charges and corresponding potentials produced at the interfaces so as to linearly regulate the interface barrier height……”. Why is the regulation of interface barrier height by piezoelectric polarization charges linear? Related discussion or literatures should be added for better readability.

2. For force/strain sensors, it is actually very interesting that typically force sensors should not respond to strain. Similarly, generally strain sensors should not respond to force. Could the authors discuss the crosstalk or possible relationship between vertical force sensing and lateral strain sensing of the demonstrated sensor? Maybe some useful literatures could be cited.

3. The lateral strain sensing mode is very similar to the traditional piezotronic transistor for strain sensing and piezoelectric nanogenerator, just as the authors repeatedly state. Compared to the traditional piezotronic transistor, why here is an additional PDMS layer introduced before the transfer of ZnO nano/microwire? What are its functions? If this additional PDMS layer is important to the high performance, what are the reasons? Besides, do the thicknesses of both two PDMS layers (one as substrate and the other as encapsulation) affect the force/strain sensing performance?

4. Line 148-149. How to simulate the change of ZnO nano/microwire length and calculate the strain? How to determine the accuracy since the author claim “the strain of ZnO nano/microwire can be accurately calculated”?

5. The authors systematically investigate and optimize the width of PDMS micro-protrusion structure mainly for vertical force sensing. Would the length and thickness affect the vertical force sensing? Besides, does the PDMS micro-protrusion structure affect the lateral strain sensing?

6. To my best understanding, the alignment of ZnO nano/microwire length’s direction with the PDMS substrate’s length direction is of great importance. And the position of PDMS micro-protrusion structure is also of great importance. I am wondering how do the author precisely control the alignment of ZnO nano/microwire and position of PDMS micro-protrusion structure?

7. About Fig. 3. There is an interesting phenomenon: as indicated in Fig. 3e, when responded to small forces, the sensor’s force sensitivity value (221.5 N^-1) and sensing range (0 to 0.6 N) at lower bias (+1 V) is much larger than that (89.7 N^-1 and 0 to 0.3 N) at higher bias (+3 V). Whereas, when responded to

large forces, the sensor's force sensitivity value (31.7 N^-1) and sensing range (0.6 to 1.1 N) at lower bias (+1 V) is much smaller than that (43.6 N^-1 and 0.3 to 1.1 N) at higher bias (+3 V). Could the authors discuss this interesting phenomenon?

8. About Fig. 4. As indicated in Fig. 4d, 4e and 4h, why are different methods adopted to calculate the gauge factors at forward and reverse biases? What are the resistance change ratio under reverse bias and the current change ratio under forward bias?

9. Line 111 and 122, the contents are written as "Fig. 1b-I and II" and "Fig. 1b-III". The information is wrong. Please double check the contents with Fig. 1.

10. The authors have reported several force/strain sensors based on piezotronic transistors previously. It is suggested that more detailed comparison between this work and their previous works should be added. For example, why are the performances improved? In the current manuscript, mainly only the performance is compared. I understand that the innovation of this work focuses on the realization of dual-modal sensing for vertical force and lateral strain. But I think if the mechanism of performance improvement is analyzed and discussed, then this work would have much more significant impact in the research field.

Overall, the authors innovatively develop a structure design to integrate the vertical force sensing and lateral strain sensing functions together in just one sensor. The performance of the force/strain sensor is fascinating. Though the authors have reported several works about the piezotronic transistors previously, this work makes a significant progress in the field of force/strain sensing. I would recommend publication in Nature Communications once the authors properly address the abovementioned comments.

Reviewer #3 (Remarks to the Author):

In this work, the authors proposed a dual-modal piezotronic transistor (DPT) that can simultaneously sense vertical force and horizontal strain. This design exhibits a higher sensitivity than similar work. However, there are several major flaws within this article, which needs to be addressed before further consideration.

1. The major technical flaw for this manuscript is that although this design can sense both the vertical force and lateral strain, it is not able to differentiate whether the force or the strain are applied. The author needs to present the data when both vertical force and lateral strain are applied together and present the data of cross talk between the two types the sensors.

2. The schematic diagram of the DPT device is basically used in the full text, so that the reviewer does not know what the specific device looks like. The authors should provide an optical image of the sensor. Additionally, how the external electrical connection can be made with the sensor?

3. The traditional pressure and strain sensing methods in Figure 1 have been widely known by researchers. Similar design without microprotrusion has been published even before 2010. Besides supplementary table 2, the authors should discuss the novelty of their design.

4. Supplementary Tables 2 and 3 compare the pressure sensing and strain sensing capabilities of the DPT devices fabricated in this paper with other studies. However, the advantages of DPT in pressure sensing comparison and strain sensing comparison are difficult to see directly from the table. It is recommended to draw a two-dimensional graph based on both Supplementary Table 2 and 3, with pressure sensing and strain sensing as the coordinate axes, respectively, to show the positions of the DPT and the comparison materials in this paper. Additionally, the authors only compare with piezotronic sensors and should compare with other pressure and strain sensing mechanism.

5. There are inconsistency within the figure caption. Inside the figure it is i,ii, but the main text is I, II.

6. In the Note 4, the authors should describe the simulation parameter of PET, such as the modulus and the Poisson ratio.

7. In the simulation, the author should also simulate the thickness of PDMS. The author should study the following two scenarios. First, the whole PDMS thickness is fixed, the effect of ratio of the top/bottom PDMS layer thickness on the ZnO strain should be studied. Second, once the bottom PDMS layer thickness is fixed, the effect of the top layer PDMS thickness on the ZnO strain should be studied.

8. In Fig. 3f, the sensitivity is 1.759eV/N, but the corresponding text in manuscript is 1.759eV/MPa, is this a typo or derived? If 1N corresponds to 1MPa, it means that the cross-sectional area of the microstructure protrusion is 1mm2, which is inconsistent with the ratio of 0.66 obtained by the author's optimization.

9. In the model of Note 8 and Fig. S8, the author believes that the Young's modulus of PDMS is smaller than that of PET. Thus, PMDS can deform with the bending of PET, and it is assumed that this deformation is close to a circular arc. In fact, since the Poisson's ratio of PDMS is close to 0.5, that is, its volume compressibility is small, so when PET is deformed, will PDMS compress strictly according to the shape of PET? Besides, is the deformation of ZnO along the direction of the arc? These issues require a more detailed proof.

10. In Fig. 4d-e， why use resistance ratio when characterizing DPT forward bias characteristics, and use current ratio when characterizing reverse bias characteristics, why not both use current ratio (similar to characterization of vertical force sensing)

11. In Fig. 5f, the definition of on/off ratio is not given.

Point-to-Point Response to Reviewers' comments:

We are grateful to thank the reviewers for their in-depth reviews and important/constructive questions/suggestions regarding our manuscript. In the pages that follow, we provide our responses to each of the reviewer's questions.

Reviewer #1 (Remarks to the Author):

The manuscript presents a novel dual-modal piezotronic transistor (DPT) that combines ZnO nano/microwires and PDMS with microprotrusions for highly sensitive force sensing. The electrical properties of the DPT are evaluated for both vertical force sensing and lateral strain sensing after structural optimization. While the DPT demonstrates high sensitivity in detecting small forces, the overall concept of the DPT is not particularly innovative and requires further validation and analysis. As such, based on the above comments, it is difficult to recommend the manuscript for publication at this time.

Response:

We appreciate the reviewer for the detailed and constructive comments/suggestions about our work, which promote us to condense the novelty of DPT and improve it.

Question #1

The objective of this study is to develop a dual-modal piezotronic transistor; however, the structural optimization of the DPT lacks sufficient data and descriptions. For instance, the detailed parameters and schematic representation of the microprotrusion were not provided. The relationship between microprotrusion width and nano/microwire deformation under the same external force did not be investigated, and the impact of materials such as PDMS and PET on the nano/microwire deformation was not discussed, either. This leads to the most fundamental factor affecting sensitivity in the DPT structure not clear.

Response:

We highly appreciate for the reviewer's suggestions and respond to the three questions

as follows (1), (2) and (3), respectively. In addition, we will also briefly discuss the main reason for the high sensitivity of DPT in the following section (4) (which will also be detailly discussed in the **response to Question#2**). At the same time, we have added these contents to the revised Supplementary Information and marked in red.

(1) For instance, the detailed parameters and schematic representation of the microprotrusion were not provided.

Response: We thank the reviewer for this valuable suggestion. The detailed parameters and schematic representation of the microprotrusion are really what we should provide. Here, we detail the information of the finite element method (FEM) analysis in the structural optimization of the dual-modal piezotronic transistor (DPT) in **Figure R1**. As illustrated in the schematic diagram of the exploded view layout (**Figure R1a**) and the side view (*xy* plane) of the DPT in vertical force sensing mode (**Figure R1b**), a ZnO nano/microwire is embedded into two PDMS layers on a PET substrate, with a microprotrusion bonded onto the top PDMS layer just above the ZnO nano/microwire. Because of the extremely small diameter of the ZnO nano/microwire (usually in the micron scale, such as about 6.5 μm) compared with the size of the microprotrusion (in the millimeter scale) and the PDMS layer (in the centimeter scale) in the direction of the *z*-axis (perpendicular to the *xy* plane), the influence of the morphology of ZnO nano/microwire on the deformation induced by the vertical force can be negligible in this work. Considering the above structural characteristics, it is reasonable for us to construct a 2D finite element model in plane stress condition using the software COMSOL Multiphysics to simulate the loading process of the vertical force and the corresponding shape change of the DPTs with various structure parameters.

As shown in **Figure R1c**, the model is constructed by two PDMS layers on a PET substrate, with a square PDMS as the microprotrusion just above the embedded ZnO nano/microwire. For convenience, the diameter of the ZnO nano/microwire can be ignored in the FEM model as considering its extremely small diameter compared with the thicknesses of PDMS and PET. Thus, we assume that a ZnO nano/microwire with length $L_{NW} = 1\,mm$ exists at the middle of the interface between the two PDMS layers. In order to investigate the influence of the microprotrusion structure ratio

(w/L_{NW}) on the ZnO nano/microwire deformation upon a vertical force and the sensitivity of the DPT, we model the microprotrusion with width (w) in the range from 0.25 mm to 1.25 mm, while the height remains constant $(h_{protrusion} = 1\,mm)$. Besides, the length of PDMS layers and PET substrate is $L = 30\,mm$; and the thicknesses of the top PDMS layer, the bottom PDMS layer and the PET substrate are $h_{PDMS,top} = 0.5\,mm$, $h_{PDMS,bottom} = 0.825\,mm$ and $h_{PET} = 0.15\,mm$, respectively. Under the boundary condition that the bottom edge of the PET substrate is fixed constraint, the vertical force on the top edge of the microprotrusion is applied by pressing a certain downward displacement (d) as shown in **Figure R1b**.

The above detailed parameters and schematic representation of the microprotrusion have been added into the Supplementary Information and marked in red.

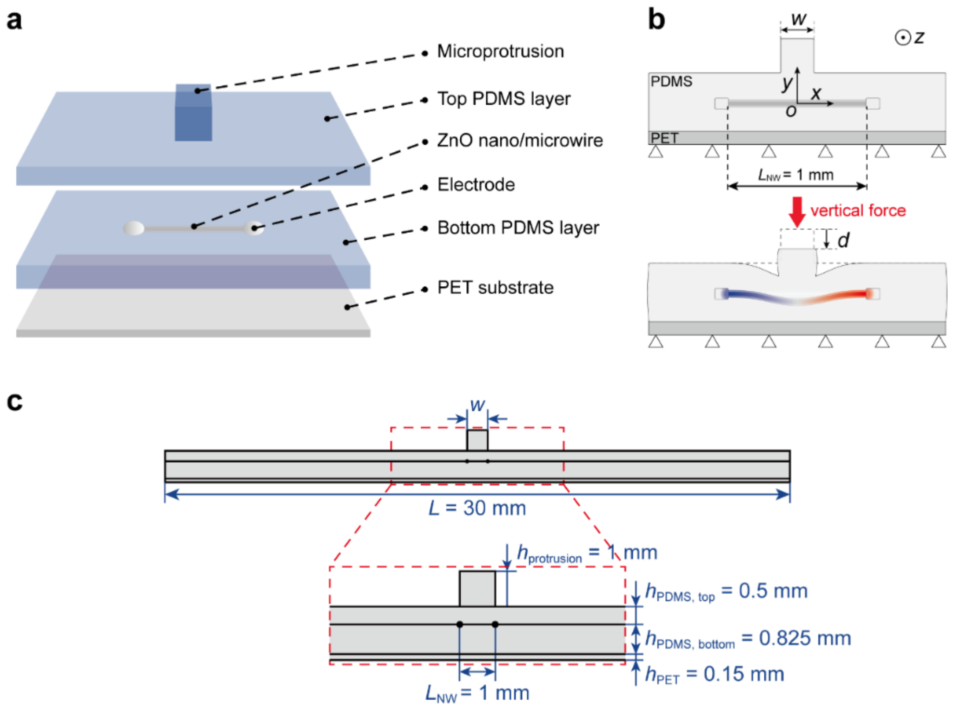

Figure R1. Schematic illustration of the dual-modal piezotronic transistor (DPT) and the 2D simulation model of DPT in the finite element method (FEM) for the structural optimization. a, **b** Schematic illustrations of the exploded view layout (**a**) and the side view at the *xy* plane (**b**) of the DPT in vertical force sensing mode. The triangle symbol represents fixed constraint. **c**, Illustrations of the FEM model with the

detailed structure parameters. Black points represent ends of the ZnO nano/microwire.

(2) The relationship between microprotrusion width and nano/microwire deformation under the same external force did not be investigated.

Response: We thank the reviewer very much for this kind reminding and suggestion. Here, we systematically investigate the downward displacement (d) of the top edge of microprotrusion and the nano/microwire deformation together with the strain induced by different external vertical force in DPTs with different microprotrusion structure ratio (w/L_{NW}).

As previously discussed in (1), the influence of the geometry of the microprotrusion along z-axis on the nano/microwire deformation under vertical force can be approximately neglected. So, the external force evenly applied to the top surface of the microprotrusion can be denoted as the contact pressure ($P = F/S$) through keeping the loaded area (or the area of the top surface) unchanged, to avoid interference of the loaded area (S) and other insignificance factors.

In order to further confirm the optimal structure ratio (w/L_{NW}) of the microprotrusion and give a convincing demonstration, we simulated the mechanical deformation process of the ZnO nano/microwire in DPTs with various microprotrusion widths (w) under uniform pressure as illustrated in **Figure R2a**. Under the application of the loading pressure varying from 0.15 MPa to 0.6 MPa on the top edge of microprotrusion as shown in **Figure R2b**, the calculated spatial distribution of displacement profiles would clearly demonstrate the shape change of the DPTs with different microprotrusion structure ratios (w/L_{NW}) in the range of 0.25~1.25.

Furthermore, the corresponding outlines of the bended nano/microwires under different conditions in **Figure R2b** could be obtained and plotted in **Figure R3a**. It can be seen from **Figure R3b** that with the application of the pressure of 0.6 MPa, a small structure ratio (for example, $w/L_{NW} = 0.25$) of the microprotrusion tends to induce the bending deformation of the nano/microwire; while a large structure ratio (for example, $w/L_{NW} = 1.25$) is more likely to cause the overall displacement of the nano/microwire. Note that the lengths of these bended nano/microwires can be estimated by the integrals derived from **Figure R3a**, which are subsequently used to

evaluate the strain of ZnO nano/microwires ($\varepsilon = \Delta l / l_0$). As demonstrated by the calculated curves of the nano/microwire strain as a function of the applied pressure in **Figure R3c**, the nano/microwire strain increases monotonously with the pressure increasing from 0 to 0.6 MPa, while the structure ratio (w/L_{NW}) around 0.75 may induce the relatively high strain level in the nano/microwire and thus a relatively excellent sensitivity. In order to further analyze the relationship between the microprotrusion structure ratio (w/L_{NW}) and the nano/microwire deformation, the nano/microwire strain as a function of the structure ratio (w/L_{NW}) under five different levels of pressure (0, 0.15, 0.30, 0.45, and 0.60 MPa) are simulated in a finer gradient of the structure ratio ranged from 0.25 to 1.25 and summarized in **Figure R3d**. It can be found that the strain level of the nano/microwire can reach a maximum value at a structure ratio (w/L_{NW}) of about 0.65~0.70 under a given pressure, which is in good agreement to the previous simulation results with application of the downward displacement (d). Also, we can find that no matter how much the loading pressure is applied, the optimal microprotrusion both possesses the structure ratio of about 0.65~0.70.

Additionally, in order to explain why the results (the optimal microprotrusion possessing a structural ratio w/L_{NW} of about 0.65~0.70) obtained by the two simulation approaches by calculating the downward displacement (d) and calculating the applied pressure are consistent with each other, we also studied the relationship between the displacement and the pressure. As shown in **Figure R4a**, the simulation results indicate that there is a good linear relationship between the downward displacement and the pressure applied on the top edge of the microprotrusion, as similar trends can be observable in DPTs with different microprotrusion structure ratios (w/L_{NW}). It is this linearity that makes the two results obtained by the two simulation approaches consistent. As a consequence, it is an appropriate approach to quantitatively apply the vertical force on the DPT with the application of the downward displacement (d), considering that the displacement (d) can be accurately controlled by a high-precision actuator in the experimental section.

Furtherly, as shown in **Figure R4b**, the downward displacement (d) increases with

the structure ratios (w/L_{NW}) slightly, and then reaches a saturated value ($d = 0.92\ mm$) under a given pressure of 0.6 MPa applied on the microprotrusion. It is worth noting that the maximum downward displacement of the top edge of microprotrusion occurs when the structure ratios is about 1.00~1.10 rather than 0.65~0.70. Actually, in vertical force sensing mode of DPT, it is not that the larger the displacement is, the better, because the larger structural ratio will make the nano/microwire move down as a whole rather than produce strain.

The above contents provide a better understand the structural characteristics of the microprotrusion in DPT and have been added into the Supplementary Information and marked in red.

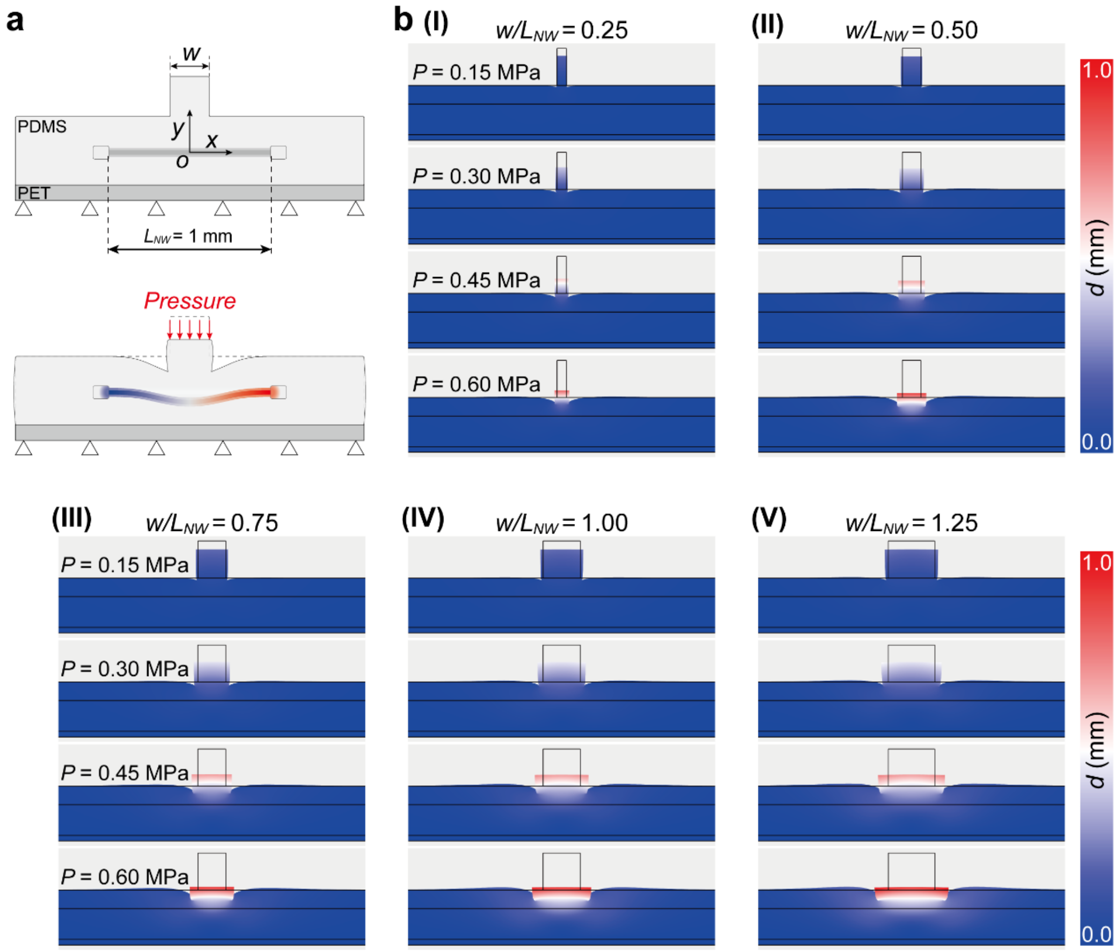

Figure R2. Simulated shapes of DPTs with various structure ratios (w/L_{NW}) under different pressures. a, Schematic illustration of the side view at the *xy* plane of the DPT unloaded (top) and loaded (bottom) with pressure. L_{NW} and w represent the

length of the ZnO nano/microwire and the width of the microprotrusion, respectively. **b**, Calculated displacement distribution profiles of the DPTs with varies of the microprotrusion structure ratios (w/L_{NW}) at different applied pressures. The loads are applied as the uniform pressure on the top edge from 0.15 MPa to 0.6 MPa and the structure ratios (w/L_{NW}) of the microprotrusion are set as 0.25~1.25. The solid line represents the original shape of DPTs before the pressure is applied. Colors represent the value of the downward deformation after compression.

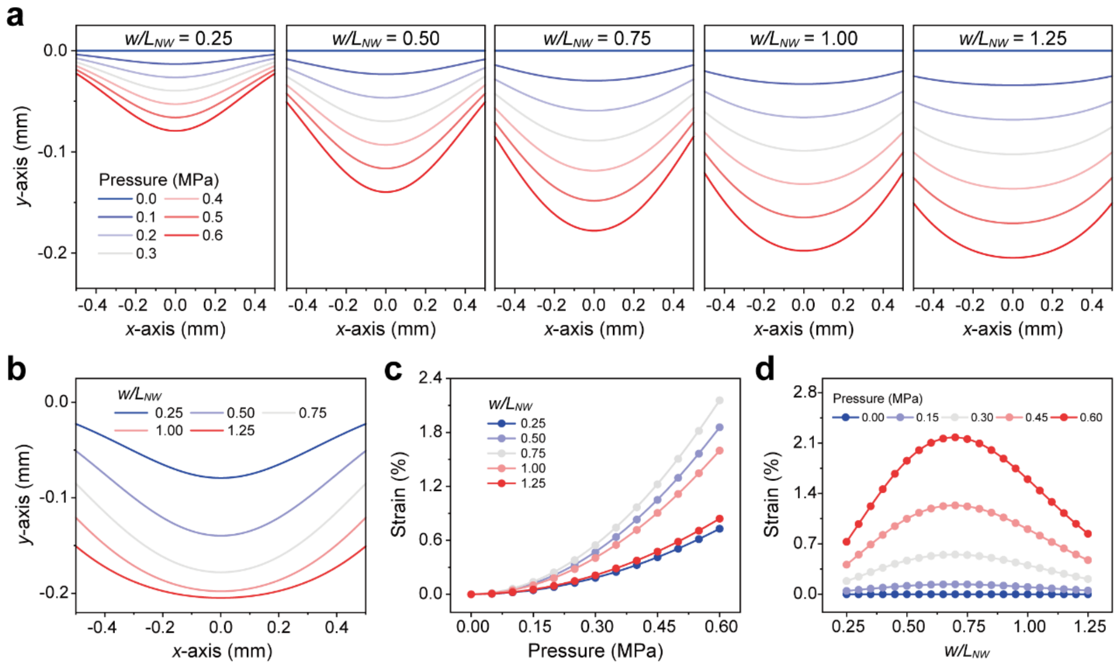

Figure R3. **Simulated results of the nano/microwire deformation in DPTs with various structure ratios (w/L_{NW}) under uniform pressures. a**, Outlines of the bended nano/microwires in DPTs with various structure ratios (w/L_{NW}) from 0.25 to 1.25 in response to different applied pressures. **b**, The corresponding outlines of nano/microwires of the DPTs with various structure ratios (w/L_{NW}) at a constant pressure of 0.6 MPa. **c**, The calculated strain of the nano/microwires in DPTs with various structure ratios (w/L_{NW}) as a function of the pressure. **d**, The curves of nano/microwire strain versus structure ratio (w/L_{NW}) under different applied pressures.

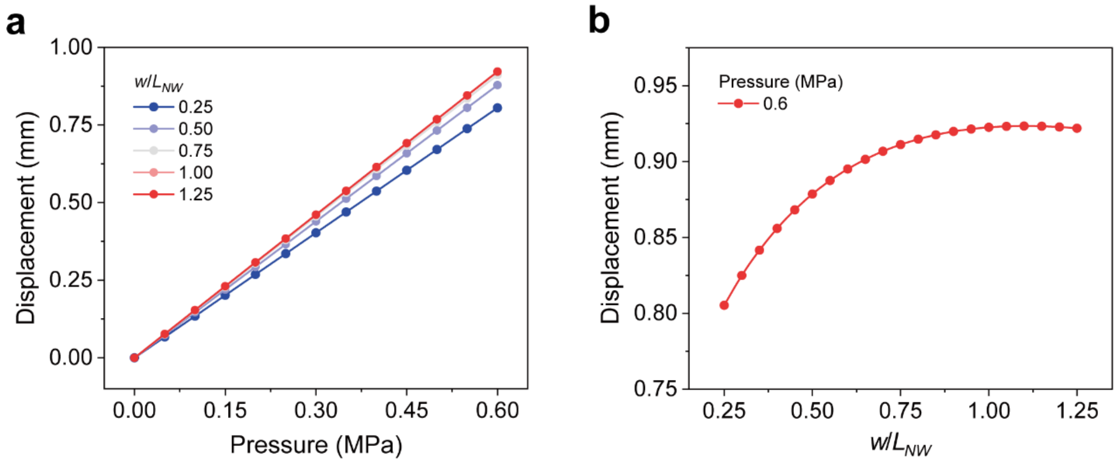

Figure R4. **The relationship between the downward displacement (d) of the top edge of microprotrusion and the applied pressure in DPTs with various structure ratios (w/L_{NW}) for vertical force sensing obtained from the FEM simulation. a**, The corresponding downward displacement (d) of the top edge of the microprotrusion as a function of the applied pressure in DPTs with various structure ratios (w/L_{NW}). **b**, The curve of downward displacement (d) versus structure ratio (w/L_{NW}) under a constant pressure of 0.6 MPa.

(3) The impact of materials such as PDMS and PET on the nano/microwire deformation was not discussed.

Response: We thank the reviewer for this nice suggestion. The impacts of the materials on DPT have been further studied. Considering the extremely small size of ZnO nano/microwire compared with the PDMS and PET, we focus on the influences of the PDMS and PET on the nano/microwire deformation as follows.

① *Impact of PDMS on the nano/microwire deformation*

As the main material in the overall structure of DPT, the mechanical properties of polydimethylsiloxane (PDMS) are directly related to the nano/microwire deformation macroscopically. PDMS, one of the most widely used elastomer materials due to its excellent flexibility, optical transparency, chemical inertness, biocompatibility and simple fabrication, demonstrates promising applications in fields such as wearable electronics, soft robots, microelectromechanical systems (MEMS), biomedicine,

microfluidics, and many others[1, 2]. Uniquely, the inherent mechanical property of PDMS, namely Young's modulus, can be further controllable through changing the degree of PDMS network's cross-linking, which can be achieved, for instance, by modifying the ratio of prepolymer to curing agent, curing time and temperature [2, 3]. Conventionally, the main property of PDMS used in the FEM (finite element method) modeling is the Young's modulus (E), which are set to be 750 kPa [4].

Here we carefully utilize the FEM simulation delving into the possible influence of the material properties (such as Young's modulus) of PDMS on the performance of the DPT for vertical force sensing. According to previous reports, modifying the cross-linker concentration and the baking time will effectively modulate the Young's modulus of PDMS (Sylgard 184) in a large range from 50 kPa to about 4 MPa [5, 6]. Based on the experimental data from the relevant literatures, the following **Table R1** gives a summary of the mechanical properties (Young's modulus E), which corresponded to a series of PDMS samples with the mixtures of Sylgard 184 base and curing agent in the $wt.$ ratios of 5:1, 10:1, 15:1, 20:1, 25:1 and 30:1 [7].

Then, we fit the given parameters to the FEM model to simulate the mechanical deformation process of the DPTs in vertical force sensing mode. **Figure R5a** shows the simulated shapes of the DPTs with a constant microprotrusion structure ratios w/L_{NW} of 1.00 and PDMS possessing different Young's moduli (E, ranged from 0.15 MPa to 3 MPa) under different downward displacement (d) of the top edge of microprotrusion. As can be seen, there is almost no difference in the shape change of the DPTs under the same downward displacement. By summarizing the data in **Figure R5a**, we can obtain the relationship between the nano/microwire strain and the downward displacement in DPTs with different Young's moduli of PDMS (**Figure R5b**), and the curves of the nano/microwire strain as a function of the Young's modulus of PDMS under different downward displacement (**Figure R5c**). Note that under the same downward displacement (d), the strain level of the nano/microwire just exhibit a slight increase due to the Young's modulus variation of PDMS, as shown in **Figure R5b** and **R5c**. It can be seen that using PDMS with different Young's moduli, we can approximately control the nano/microwire strain by controlling the downward displacement of the top

edge of microprotrusion.

Also, the plots of the corresponding pressure applied on the DPT as a function of the displacement (d) are shown in **Figure R6**. With the Young's modulus of PDMS ranging from 0.15 MPa to 3 MPa, the range of loading pressure can be dynamically changed from the low-pressure range (0~72 kPa) to the high-pressure range (0~2.1 MPa), as shown in **Figure R6a**. A strong linearity can be found between the loading pressure and the displacement. Moreover, it can be seen clearly from **Figure R6b** that the material properties of PDMS have a notable effect on the pressure of the DPT, since the applied pressure linearly increases with the Young's modulus (E) under the same displacement (d). These findings suggest that we can effectively expand the pressure working range via tuning the Young's modulus of PDMS to optimize the sensing capability of DPT, which demonstrates its feasibility in broadening the application scenarios.

It should be noted that we use the microprotrusion here with a structural ratio of 1.00 to simulate as a demonstration, mainly considering the mechanical stability of the microprotrusion particularly when the PDMS Young's modulus is relatively small (for example, 0.15 MPa); in fact, for DPTs with other structural ratios, the conclusions derived from the above discussions remain unchanged.

② *Impact of PET on the nano/microwire deformation*

Ploy(ethylene terephthalate) (PET) is also a key material for DPTs. We also investigate the influence of the material properties of PET on the sensing performance of the DPT. It is well-known that PET film is a common polymer material with good strength, flexibility, electrical insulation, and chemical stability, which is widely used in industrial applications such as photographic films, flexible electrical circuits, and packaging materials [8, 9]. In the FEM simulation, the Young's modulus ($E = 4$ GPa) of PET reported in the previous paper is adopted [8].

Figure R7a-c show the displacement distribution profiles obtained from the FEM simulation under different downward displacements (d), which exhibit the deformation of the DPTs with PET substrate (**Figure R7a**), without substrate (**Figure R7b**) and with PDMS substrate (**Figure R7c**), respectively. As demonstrated by the corresponding

outlines of nano/microwires in **Figure R7d** and **R7e**, the existence of PET substrate has a negligible effect on the nano/microwire deformation in vertical force sensing mode, since the nano/microwire strain will remain essentially unchanged in the DPTs with and without PET substrate (**Figure R7g** and **R7h**). This mainly results from the fact that the deformation of PET substrate is almost imperceptible under the application of vertical force and the quite high hardness of PET ($E = 4$ GPa). Hence, the material properties of PET substrate should not contribute to the sensing performance of the DPT. In contrast, upon application of the substrate material that much softer than PET (i.e., PDMS), the increase in the thickness of bottom PDMS layer would induce a larger bending deformation and thus yield a larger strain level of the nano/microwire in the DPT (**Figure R7f** and **R7i**). The maximum strain of the nano/microwire in the DPT with PDMS substrate can reach 1.36%, whereas that of the nano/microwire with PET substrate is about 1.21% under the same displacement of 0.8 mm, as shown in **Figure R7g** and **R7i**.

By systematically studying the influences of the microprotrusion width, the downward displacement and pressure, and the material properties on the DPT in the vertical force sensing mode, we can have in-depth understandings of the structural optimization of DPT. The above discussions have been added to the Supplementary Information to improve the quality of the paper. Thank the reviewer again for the constructive and valuable suggestions.

Table R1. Summary of Young's moduli (*E*) of the PDMS samples with different base/agent ratios [7].

Sample, Base/agent ratio	Young's modulus, *E*
PDMS, 5:1	3 MPa
PDMS, 10:1	2 MPa
PDMS, 15:1	1.5 MPa
PDMS, 20:1	0.8 MPa
PDMS, 25:1	0.45 MPa

PDMS, 30:1	0.15 MPa

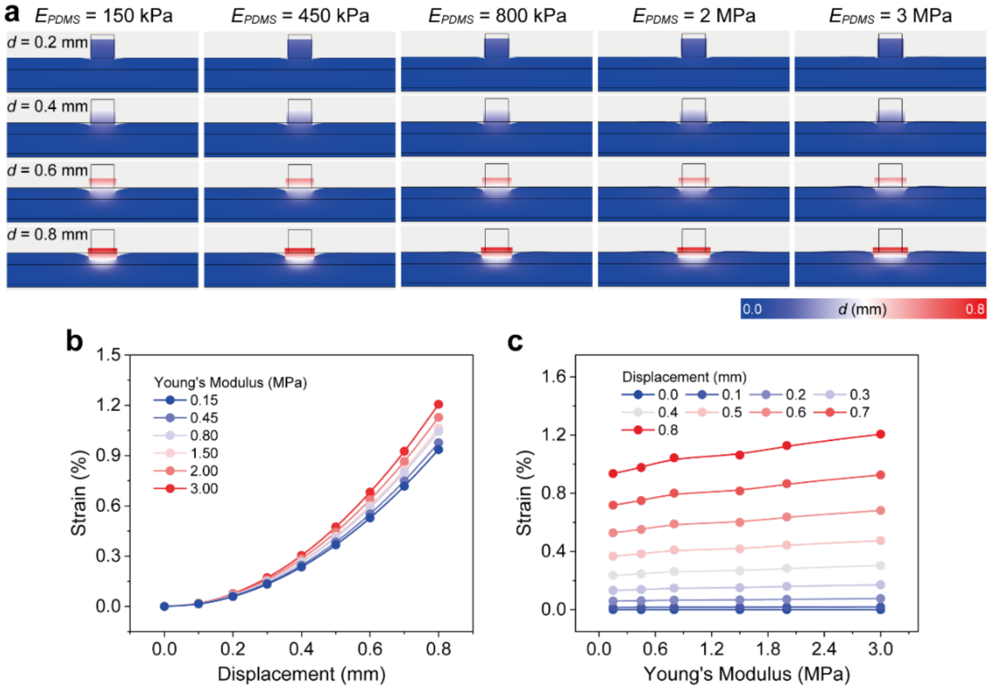

Figure R5. Impact of the material properties of PDMS on the performance of the DPT in vertical force sensing mode. a, Simulated shapes of DPTs fabricated by PDMS with various Young's moduli (E) under different downward displacement (d). The microprotrusion structure ratio in the simulations is 1.00. **b**, Calculated strain of nano/microwires in DPTs with various E as a function of d. **c**, The curves of nano/microwire strain versus E under different downward displacements.

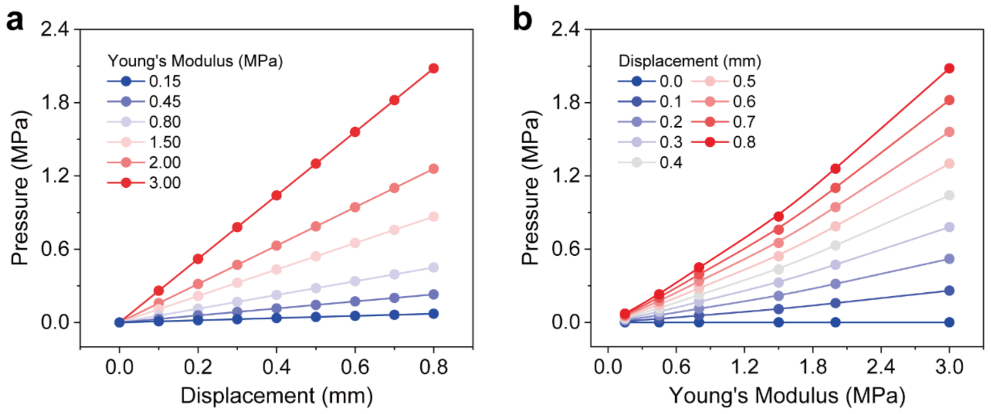

Figure R6. Impact of the material properties of PDMS on the relationship between the pressure and the downward displacement. a, Pressure applied on the top edge of microprotrusion as a function of the downward displacement (d) in the DPTs fabricated

by PDMS with various Young's moduli (E). **b**, The relationship between the pressure and the Young's modulus of PDMS in response to different downward displacements.

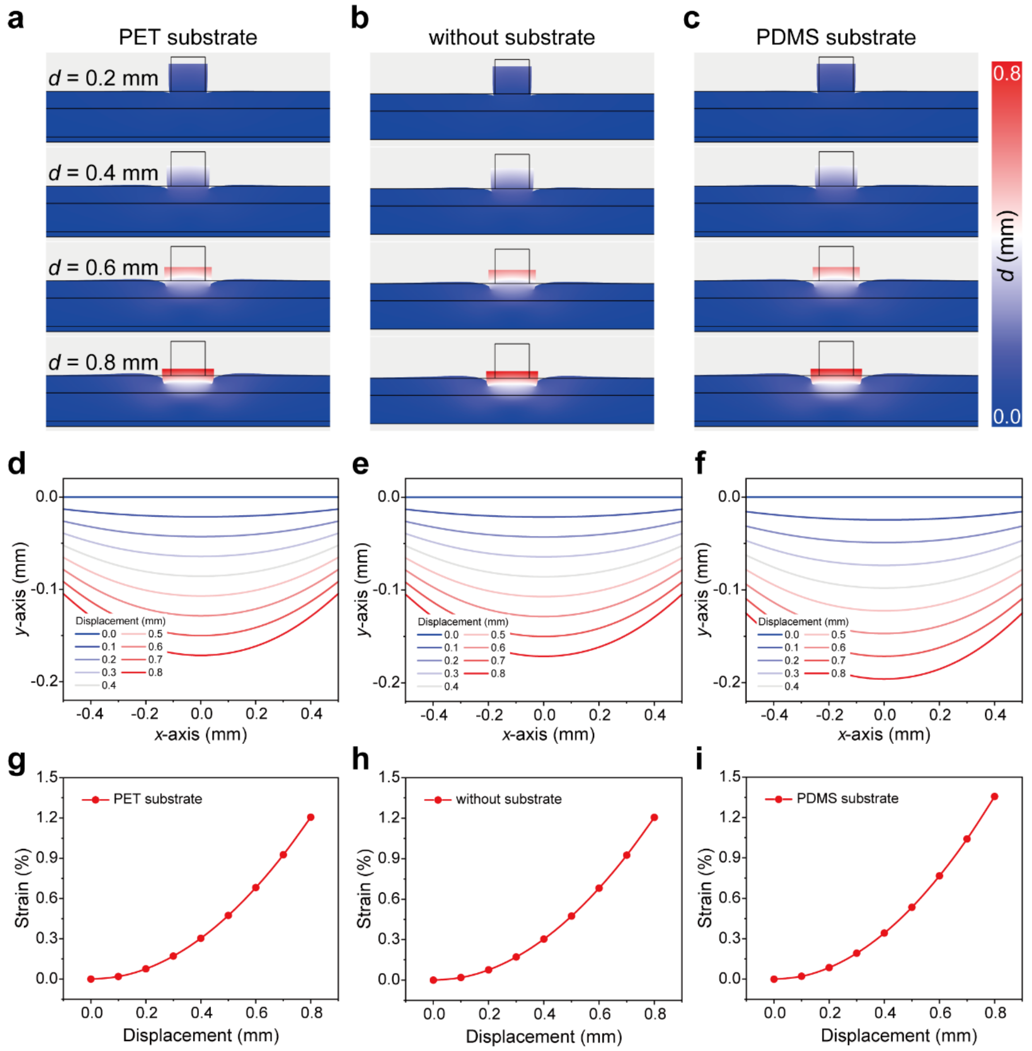

Figure R7. Impact of the PET substrate on the performance of the DPT in vertical force sensing mode. a-c, Displacement distribution profiles of the DPTs with PET substrate (**a**), without PET substrate (**b**) and with PDMS substrate (**c**) in response to different displacements (d) obtained from the FEM simulations. **d-f**, The corresponding outlines of bended nano/microwires in the DPTs with PET substrate (**d**), without PET substrate (**e**) and with PDMS substrate (**f**) under different displacements. **g-i**, The calculated nano/microwire strain as a function of displacement in the DPTs with PET substrate (**g**), without PET substrate (**h**) and with PDMS substrate (**i**), respectively.

Question #2

There have been many reports of multidirectional, high-sensitivity strain/pressure sensors in recent years. This work is based on the more established piezoelectric transistor principle to detect small stresses in multiple directions. Although the manuscript demonstrates the performance of DPT as a sensor in different modes, it appears to be an incremental improvement in sensitivity compared to existing work. However, this improvement alone may not guarantee publication in Nature Communications.

Response:

We thank the reviewer very much for this constructive comment, which promotes us to better condense the novelty of our work. We are very sorry for the lack of in-depth discussion of the novelty of the dual-modal piezotronic transistor (DPT) in the manuscript, which may have caused some misunderstandings. Actually, the design of DPT is not a simple structural optimization or parameter increase, but a new design in structure and in principle, which perfectly solves the long-term existed challenges/problems in traditional piezotronic transistors and has structural advantages in amplifying the detection of vertical force, making a qualitative leap in both performance improvement and functional expansion in piezotronics. In order to clarify the novelty of DPT in a clear and detailed way, we will discuss (1) the challenges long-term faced in piezotronics, and (2) the structural evolution of the piezotronic transistors in the past to solve the problems/challenges, and (3) the original design idea of DPT according to our thinking about the key point of the challenges in piezotronics, and (4) the achievements regarding both the performance improvement and functional expansion of piezotronic transistors realized by the innovative design of DPT. It is expected that the importance of DPT can be clarified through this process.

(1) Challenges long-term faced in piezotronics

Piezotronic transistor, utilizing piezoelectric polarizations to control the interface barrier height thus to achieve the active interaction between electronics and external stimuli, is a kind of new principle device and exhibits great potential in mechanical sensing [10]. In principle, piezotronic transistors should possess inherent high sensitivity in sensing mechanical stimuli due to its natural exponential control of output signal by

input signal. However, currently, piezotronic transistors still face many problems toward applications both in terms of functional integration and performance improvement.

As shown in **Figure R8**, a long-standing challenge is that the huge gap between the two typical structures (lateral structure and vertical structure) makes piezotronic transistor can only measure lateral strain or can only measure vertical force, which cannot meet the demand of actual applications that require the same sensor to switch between two working modes. Although there have been many reports of multidirectional, high-sensitivity strain/pressure sensors in recent years, **it is still a big challenge for piezotronic transistor to integrate vertical force sensing and lateral strain sensing into one device.**

Another long-term challenge or problem affecting sensing performance, associated with piezotronic transistor from the invention to the present, is that the traditional vertical structure of piezotronic transistor has a nano/microwire buckling effect upon vertical compressive force, which seriously limits the performance of piezotronic transistors. Due to this problem of buckling effect of nano/microwires (which will be detailly described and discussed in the following section (2)), the pressure sensitivity of piezotronic transistors is always kept within about 100 meV/MPa.

These two dilemmas have long existed since 2007 and have hindered the development and application of the piezotronic transistor. Actually, the design of DPT in this work is to simultaneously overcome above two challenges by absorbing the advantages of the traditional lateral structure but avoiding the disadvantages of the traditional vertical structure, thus to enable piezotronic transistor with two working modes and enhanced performance, which makes a qualitative leap in both function and performance of piezotronic transistors.

The key to successfully overcome the above two challenges lies in the vertical force sensing mode of DPT. Next, we will give priority to the structural evolution of the traditional vertical structure for vertical force sensing and the long-term existing problem (negative influence of buckling effect on piezotronic effect), and then derive our original design idea on the vertical force sensing mode of DPT step by step.

(2) Structural evolution of piezotronic transistors to solve the problem of buckling effect

The earliest design of using piezotronic effect to sense vertical force is a vertical structure composed of a vertical aligned wurtzite ZnO single crystal nano/microwire sandwiched and Schottky contacted by two electrodes (**Figure R9a** and Figure 1a-I). Since ZnO is a semiconductor possessing piezoelectricity, positive and negative piezoelectric polarization charges and potentials will be generated at the upper and the lower ends respectively upon a vertical compressive force according to the polarization axis (*c*-axis) shown in **Figure R9b**. These vertical-force-induced piezoelectric charges/potentials can control the electrical transport by modulating the Schottky barrier heights at interfaces formed between ZnO and electrodes. As a result, we can achieve the detection of the vertical force by measuring the change of the electrical transport. This is the basic principle of the earliest design for vertical force sensing.

However, this structure has an unavoidable problem. When a vertical compressive force is applied on the vertical nano/microwire, the nano/microwire will withstand a compressive deformation along its length direction accompanied by bending deformation, which is known as buckling effect as illustrated in **Figure R9c**. We can imagine that a large part of the vertical force is consumed to bend the nano/microwire. As a result, the net compressive deformation along the nano/microwire and the corresponding generated piezoelectric charges/potentials at two ends of the nano/microwire both decreases as the buckling effect occurs, which has a negative influence on piezotronic modification on the electrical transport.

Additionally, the buckling effect in the vertical structure will also cause an uneven piezoelectric potential distribution at the ends of the nano/microwire as simulated in the enlarged inset of **Figure R9c** due to the bending nano/microwire with one side stretched and the other compressed. This leads a nonuniform piezotronic modulation on the interface Schottky barrier. Because the interface electrical transport is mainly determined by the region with the lowest barrier height, the nonuniform modulation caused by the buckling effect will also have a negative influence on the piezotronic modification on the electrical transport.

Due to the negative impact of the buckling effect (**Figure R9d**), the early piezotronic transistors based on vertical nano/microwires (**Figure R10a**) for vertical force sensing exhibits low pressure sensitivities (CdSe nano/microwire [11]: 0.677 meV/MPa; ZnO nano/microwire [12]: 0.083-0.422 meV/MPa; GaN nano/microwire [13]: 0.212-0.240 meV/MPa).

In order to weaken the buckling effect, nano/microwire clusters encapsulated by PDMS are used in 2012 as the core unit of piezotronic transistor (**Figure R10b**) [14]. In this design, the nano/microwire is bounded by the PDMS and the nano/microwires around, which is not easy to be bent upon a vertical compressive force. The piezotronic transistor based on this structural improvement achieves a pressure sensitivity up to 33.39 meV/MPa. However, the nano/microwires in the nano/microwire clusters are not completely vertical, and there still exists buckling effect when subjected to the vertical compressive force.

In 2017, a design of replacing vertical nano/microwire or nano/microwire cluster with nanoplatelet is proposed to solve the problem of buckling effect as shown in **Figure R10c** [15]. Since the nanoplatelet has an aspect ratio (thickness-to-diameter) of about 0.42, it will not be buckle as the vertical nano/microwire subjected to a vertical force. The piezotronic transistor based on ZnO nanoplatelet exhibits pressure sensitivity of 60.97~78.23 meV/MPa, which is greatly improved from the ones based on vertical nano/microwires or nano/microwire clusters. However, due to nanoplatelets' small size both in thickness and diameter, it is very difficult to fabricate functional devices with furtherly enhanced performance. So, this design provides a possible solution to the problem of buckling effect, while it is not a feasible scheme for the time being.

The problem of the buckling effect in vertical nano/microwires, which weakens the piezotronic effect, is still not properly solved, and still restricts the performance of piezotronic transistors so far. Finding schemes to avoid the problem of buckling effect in nano/microwire-based piezotronic transistor is important for their applications in sensing.

(3) Original design idea of DPT according to our thinking about the key point of the challenges in piezotronics

From the above introduction about structural evolution of piezotronic transistors, we can find that the buckling effect mainly derived from the compression bending of the nano/microwire caused by the vertical compressive force (**Figure R11a**). A question (**Figure R11b**) arises, that is, can we utilize the elongation of nano/microwires rather than the compression of nano/microwires to achieve the detection of vertical force.

Based on this question and inspired by the suspension bridge (**Figure R11c**), we designed a structure composed of a lateral nano/microwire with two ends constrained by electrodes as illustrated in **Figure R11d-I**. If the vertical force is applied on the middle of the lateral nano/microwire, the nano/microwire will be bent downward and elongated. In this way, we can use the elongation of lateral nano/microwire to sense the applied vertical force, and perfectly solve the problem of the buckling effect in the vertical structure. This is the prototype design of DPT.

It should be noted that the downward bending here is totally different from previously discussed bending of vertical nano/microwire in the problem of the buckling effect. The vertical force induced downward bending of the lateral nano/microwire here is to elongate the nano/microwire, thus to generate piezoelectric charges/potentials. As a contrast, the vertical compressive force induced bending deformation (buckling effect) of the vertical nano/microwire decreases the net compressive deformation along the nano/microwire, weakening the generated piezoelectric charges/potentials.

In addition to the above advantage of avoiding the problem of buckling effect, this new design has another advantage. As shown in **Figure R11d-I**, based on a very simple model, we can find that this newly designed structure should have high response in the detection of the vertical force. As a small vertical force F_{vertical} is applied on the middle of the nano/microwire, a very large tensile force $F_{lateral}$ approximately equaling to $F_{vertical}/(2 \cdot \cos\theta)$ (θ is the angle between nano/microwire and vertical line) would be produced along the length of the nano/microwire. Since θ approaches 90°, $F_{lateral}$ ($\approx F_{vertical}/(2 \cdot \cos\theta)$) is a very large value. Although this estimation model is very rough, it is qualitatively estimated that a small vertical force can indeed cause the nano/microwire to tighten and produce a very large tensile force along the nano/microwire.

Our earliest device structure is illustrated in **Figure R11d-II**. However, this structure is very easy to be damaged. Even a very small vertical force will cause the nano/microwire to be broken into two sections. Then, in order to avoid very large local stresses particularly at the positions of the force point and electrodes, we used PDMS to wrap the nano/microwire and designed the structure shown in **Figure R11e**. The soft nature of PDMS allows the nano/microwire to sustain a large enough tensile stress, which can avoid the nano/microwire fracturing when subjected to very large local stresses.

To furtherly improve the performance, as illustrated in **Figure R11f**, a microprotrusion is applied to make the vertical force mainly concentrate on the middle part of the nano/microwire, thus more effectively elongating the nano/microwire rather than pressing the whole nano/microwire down.

Through the above thinking about the key point of the challenge in piezotronics and the exploration of the new design, we designed DPT possessing four features:

I. it has a basic principle of utilizing the response of lateral nano/microwire to vertical force to realize the vertical force sensing;

II. it utilizes elongation of nano/microwires (rather than compression of nano/microwires) to sense vertical force, which properly solves the problem of buckling effect in traditional vertical structure.

III. the structure of DPT inspired by suspension bridge can amplify the vertical force to the tensile force along the length of the nano/microwire;

IV. the soft nature of PDMS avoids extensive local stress, and the microprotrusion makes the vertical force concentrate on the middle of the nano/microwire, thus more effectively elongating the nano/microwire under the action of vertical force.

(4) Achievements regarding both the performance improvement and functional expansion of piezotronic transistors realized by the innovative design of DPT

The new design of DPT promotes the piezotronic transistor with both merits of performance improvement and functional expansion, as illustrated in **Figure R12**.

① *Performance improvement: high pressure sensitivity*

Based on the new design of DPT, we successfully achieve the piezotronic

transistor with a pressure sensitivity of 1.759 eV/MPa, which is 16.5 times more than that of previously reported piezotronic transistors.

Comparing the differences and analyzing the progressive relationships between the DPT in this work and the piezotronic transistors in previous works, we can find that the high performance of DPT is not a simple structural optimization or parameter increasement, but results from the advanced structural design (containing suspension-bridge-inspired structure and introduction of microprotrusion) of amplifying the vertical force to the tensile force along the length of the nano/microwire and the perfect solution to the problem of buckling effect.

② *Functional expansion: two working modes*

Fortunately, through previous discussions about the structural evolution in piezotronic transistors (Section (2)) and our research idea of DPT (section (3)), the design of DPT realizes the detection of vertical force with ultrahigh performance based on a new lateral structure; Meanwhile, the new design itself has a lateral structure similar to the traditional piezotronic transistor shown in Figure 1a-II, which makes the new design of DPT naturally suitable for lateral strain sensing. As a result, DPT with the new lateral structure that enables two working modes, one is vertical force sensing and the other is lateral strain sensing. DPT inherits all the advantages of the traditional lateral structure to detect the lateral strain, and can also detect the vertical force without the problem of buckling effect existed in the traditional vertical structure.

The design of DPT successfully overcomes the dilemma that piezotronic transistor can only be used for vertical force sensing or only be used for lateral strain sensing, and for the first time enables piezotronic transistor to possess two sensing functionality and more possibilities in practical application, which would subvert the structural design of traditional piezotronic transistors and provide new ideas for new principle devices in the field of piezotronics to promote the application of piezotronic effect.

In summary, **the design of DPT is not a simple structural optimization or parameter increase, but a qualitative leap in both performance improvement and functional expansion in piezotronics** (**Figure R12**). This is a big step in the field of piezotronics.

We thank the reviewer again for this kindly reminding and constructive suggestion. In order to improve the quality of our work, we have revised and revealed the novelty of our design more clearly in the manuscript and marked them in red. Also, the above discussions about the structural evaluation of previously reported piezotronic transistors and our original research idea about DPT have been added into the Supplementary Information.

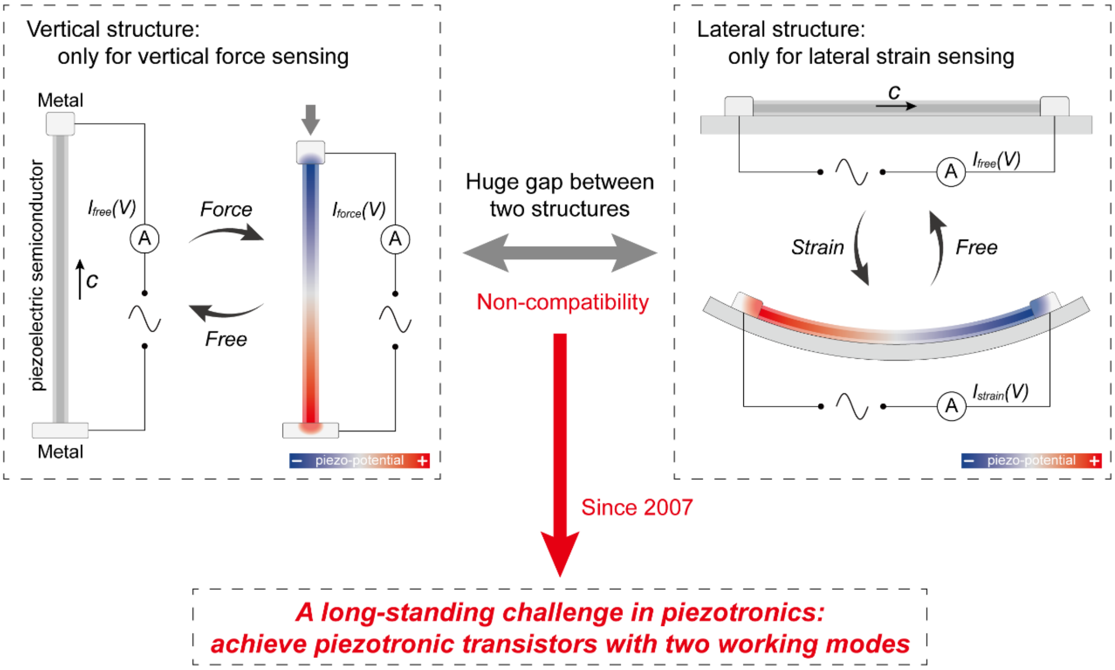

Figure R8. A long-standing challenge in piezotronics. The left dotted box shows the traditional vertical structure of piezotronic transistors composed of a vertical aligned nano/microwire sandwiched by two electrodes, which can only be used for vertical force sensing, while the right dotted box shows the traditional lateral structure of piezotronic transistors possessing a lateral nano/microwire clamped by two electrodes, which can only be used for lateral strain sensing. Since 2007, the incompatibility of the two structures (vertical structure and lateral structure) of piezotronic transistor has made it impossible to achieve a same piezotronic transistor to switch between two working modes, which is a long-standing challenge in the field of piezotronics.

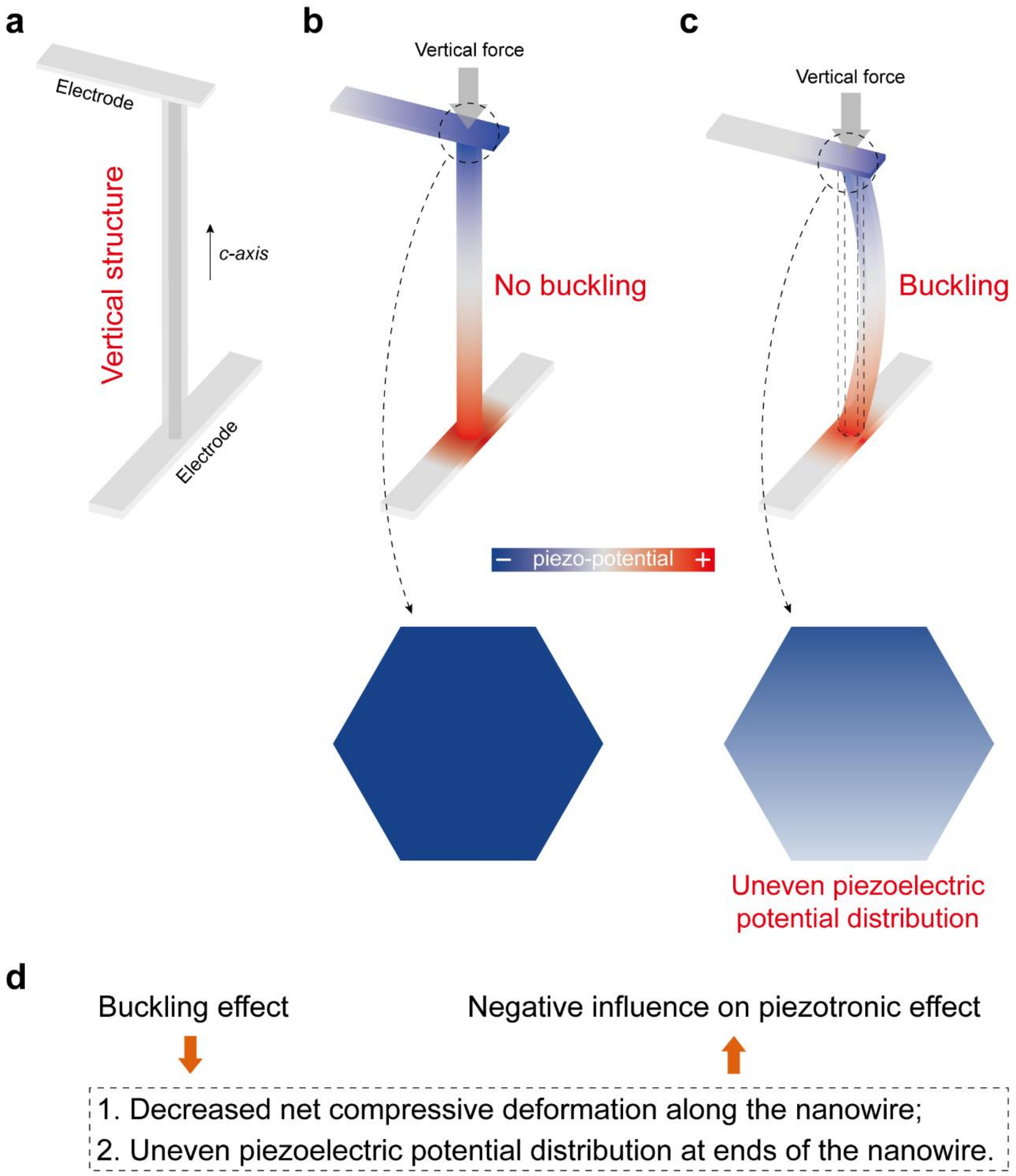

Figure R9. Problem of buckling effect in vertical structure of piezotronic transistors. a, A piezotronic transistor with a vertical structure composed of a vertical aligned nano/microwire sandwiched by two electrodes. **b**, **c**, Illustrations of vertical force induced piezoelectric potential distributions without (**b**) and with (**c**) considering the buckling effect. The enlarged insets are illustrations of the potential distributions on the upper end of the nano/microwire. **d**, Buckling effect will lead to a decreased net compressive deformation along the nano/microwire and an uneven piezoelectric potential distribution at the ends of the nano/microwire, which has negative influence on piezotronic effect.

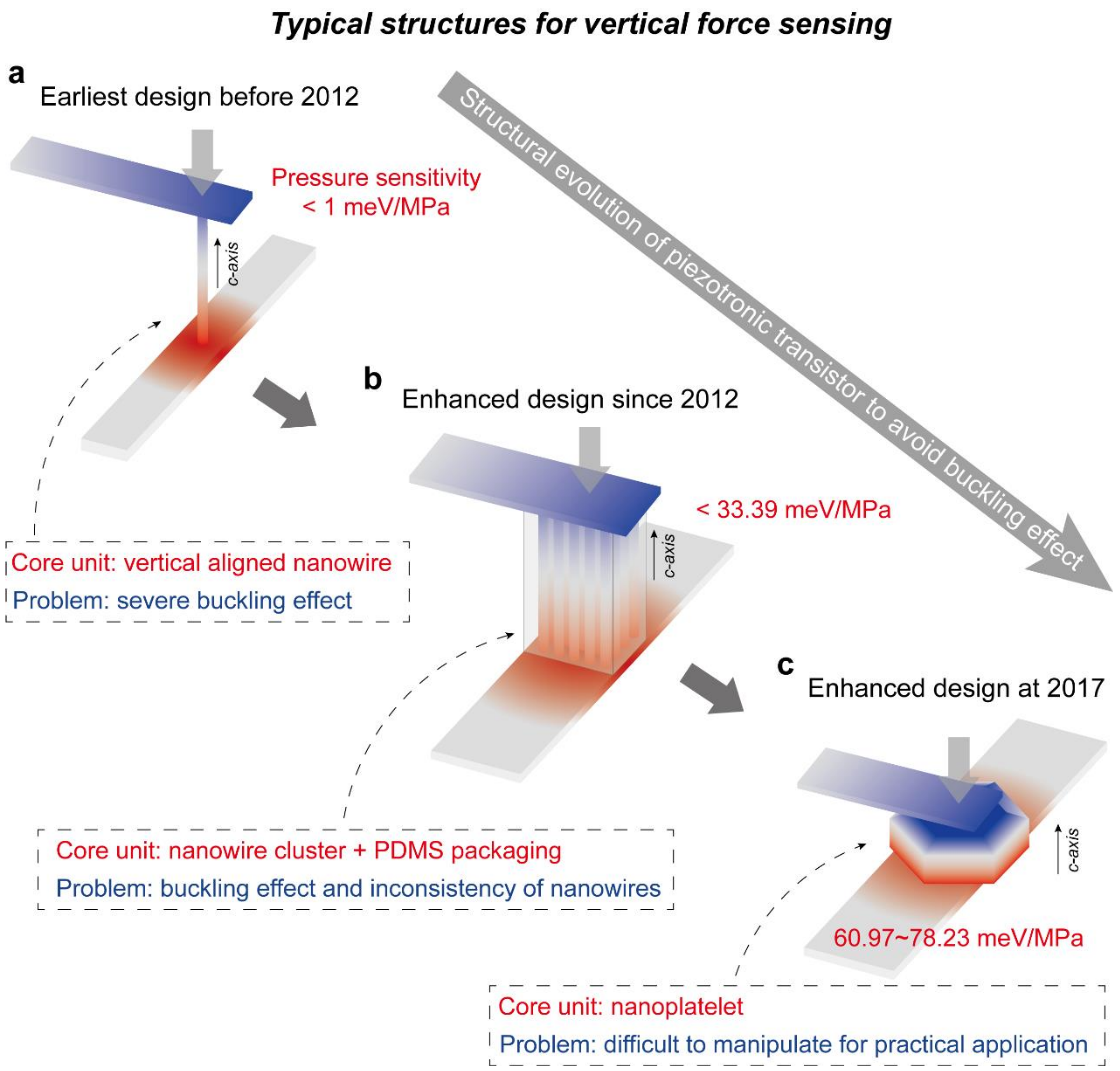

Figure R10. Structural evolution of piezotronic transistors to solve the problem of buckling effect. a, The earliest design of piezotronic transistor for vertical force sensing. It is based on the vertical aligned nano/microwire but possesses a problem of severe buckling effect, which is not conducive to piezotronic effect. **b**, Enhanced design of piezotronic transistor based on nano/microwire cluster encapsulated by PDMS. Bounded by the PDMS and the nano/microwires around, vertical nano/microwire in the cluster is not easy to be bent (buckling effect) upon a vertical compressive force, which can enhance the performance to a certain extent. The still existing buckling effect and the inconsistency of nano/microwires hinder the further improvement of performance. **c**, A design based on nanoplatelet to solve the problem of buckling effect. It is difficult to manipulate the nanoplatelets for practical application.

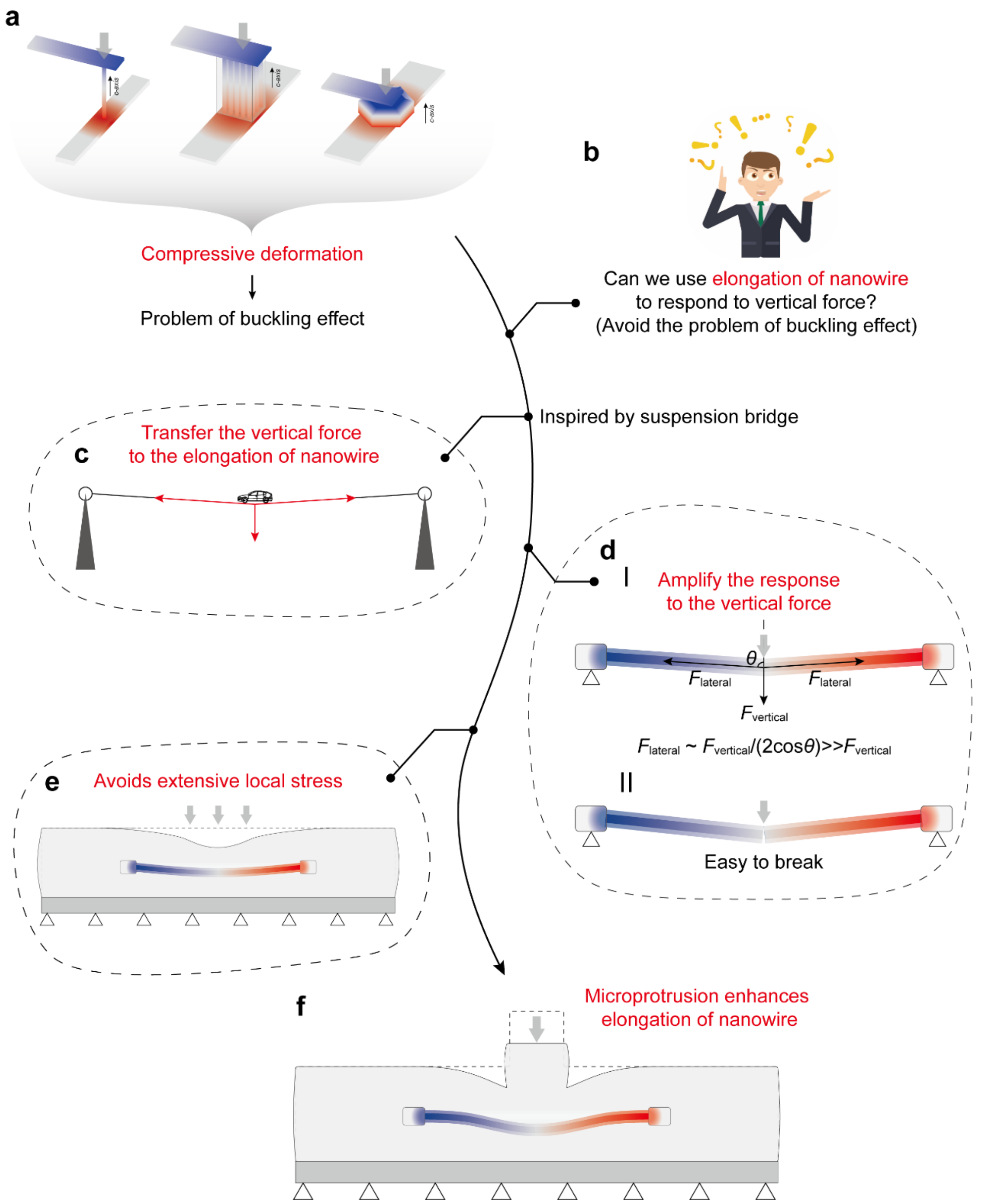

Figure R11. Our original design idea of dual-modal piezotronic transistor (DPT). **a**, Traditional vertical structures with a common feature of utilizing compressive deformation caused by vertical force to sense the vertical force. This sensing principle is easy to induce the problem of buckling effect. **b**, A question raised after investigating the structural evolution of traditional piezotronic transistors to avoid the problem of buckling effect: can we use elongation of nano/microwire to respond to vertical force? **c**, A suspension-bridge-inspired structure that can transfer the vertical force to the

elongation of wire, and thus achieve elongation of nano/microwire to respond to vertical force. **d**, The earliest structure of DPT that can amplify the response to the vertical force. Directly applying the vertical force on the lateral nano/microwire can easily lead to the fracture of the nano/microwire. **e**, Using PDMS to encapsulate the lateral nano/microwire to avoid extensive local stress. **f**, Final design of DPT. Introduction of the microprotrusion makes the vertical force concentrate on the middle of the nano/microwire, thus more effectively elongating the nano/microwire under the action of vertical force.

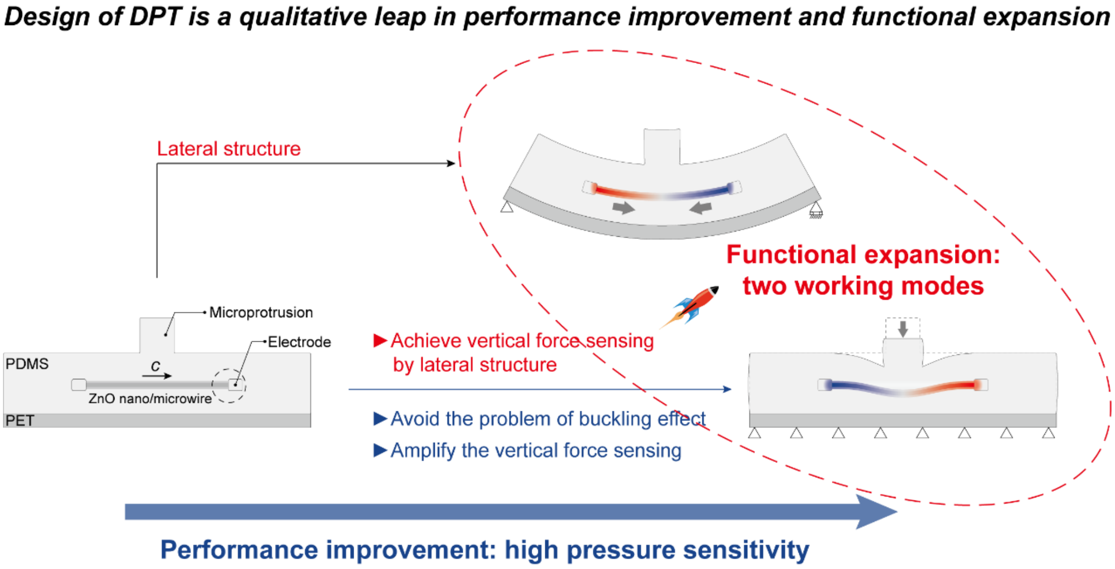

Figure R12. Novelty of the dual-modal piezotronic transistor (DPT). In terms of performance improvement (blue font marked), DPT possesses high pressure sensitivity resulting from the perfect solution to the problem of buckling effect and advanced structural designs containing the suspension-bridge-inspired structure and introduction of microprotrusion that can amplify the vertical force sensing. In terms of functional expansion (red font marked), DPT enables one piezotronic transistor with two working modes for vertical force sensing and lateral strain sensing, which benefits from the new lateral structure design of DPT.

Question #3

The author presents pressure-dependent simulations along with experimental data to validate them. However, in order for the data to be considered valid, it is essential to demonstrate multiple data points and their corresponding errors especially in sensor research, where accuracy is critical. As the author has not presented enough data points, it is difficult to evaluate the validity of the findings. Therefore, it is recommended that the author includes additional data points in the manuscript to support their conclusions.

Response:

We are grateful to the reviewer for raising this important and constructive suggestions. And, we are very sorry that the full characterizations of the strain sensing mode and the on/off ratio of DPT are ignored and insufficient due to our focus on the design of DPT for vertical force sensing before. As the reviewer's kind suggestions, and in order to give a convincing conclusion, we have carried out additional experiments, revised the **Figure 4** and the **Figure 5** with more data points, modified the corresponding contents in the revised manuscript, and marked them in red, as detailly discussed in the following **Figure R13** and **Figure R14**.

(1) DPT in lateral strain sensing mode (Figure 4 in the revised manuscript)

The description about **Figure 4** and the revised **Figure 4** are shown and marked in red as follows:

"Next, we investigated the performance of DPT as a strain sensor along the lateral direction. ……. It utilizes the bending of the substrate (a certain curvature radius *R*) to compress the ZnO nano/microwire and introduce piezoelectric polarization charges at two ends. The applied strain on DPT is controlled by changing the radius of the steel coil (inset of **Fig. 4a**).

Fig. 4b shows the lateral strain-dependent *I-V* characteristics of DPT. …… Through the analysis of **Fig. 4c** and the consideration of DPT's asymmetric modulation (**Supplementary Fig. 34**), we can obtain …… To evaluate this offsetting effect, we calculated the change of effective Schottky barrier height (SBH) as a function of the strain (**Fig. 4g**), indicating the decreased effective SBH under reverse bias and the increased effective SBH under forward bias. It can be found that the opposite regulation

of the piezotronic effect on the interface barriers is almost linearly related to the strain, particularly in the high strain range from about -2% to -6%.

In order to evaluate the performance of DPT for lateral strain sensing, we derived the gauge factor, which is defined as the ratio of relative change in current or resistance to the strain (**Supplementary Note 16**), of DPT under different biases in **Fig. 4h**. When the lateral strain is about -5.8% and the bias is -3 V, the highest gauge factor of DPT can reach 8988.6, which is a relatively high value in strain sensors (**Supplementary Note 17**, **Supplementary Fig. 36** and **Supplementary Table 6**).”

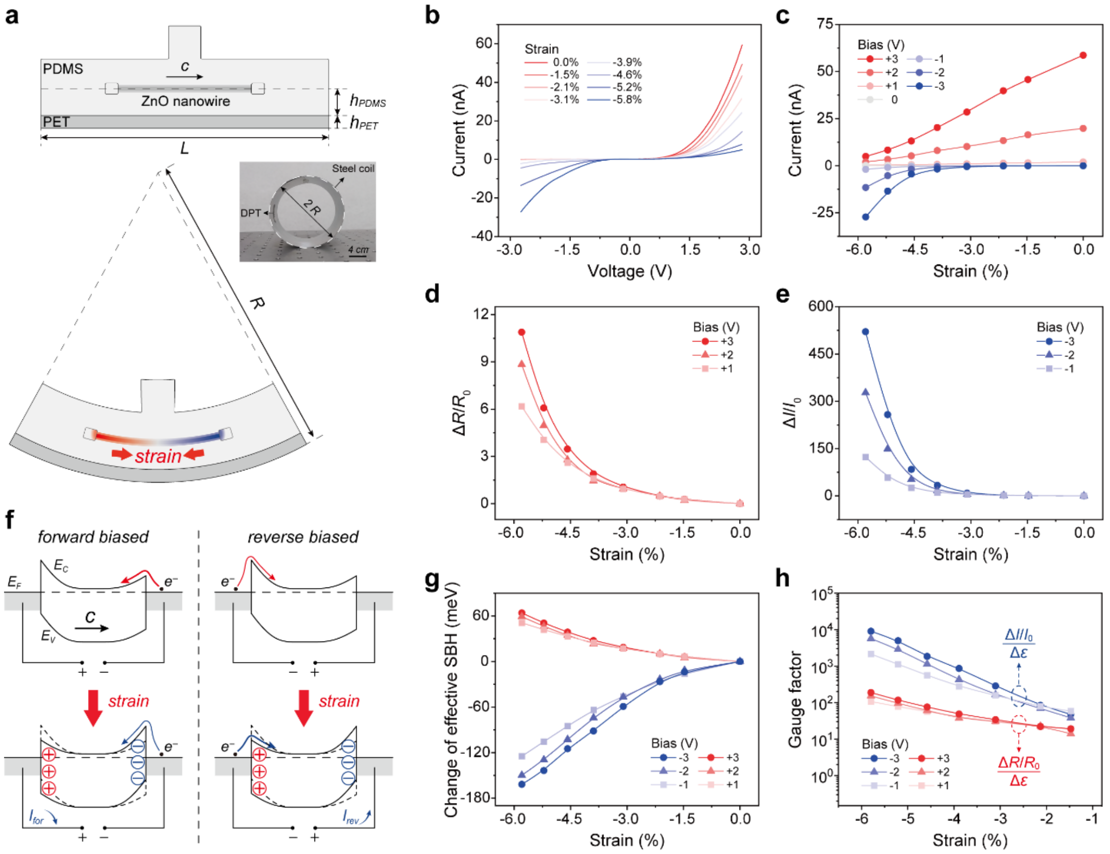

Figure R13 *(Figure 4 in the revised manuscript)*. **Piezotronic modulation of electrical characterization in the dual-modal piezotronic transistor (DPT) for lateral strain sensing.** **a**, Schematic diagrams of the DPT showing the driving mode for lateral strain sensing to evaluate the axial strain (ε) of the nano/microwire. The bending radius of the PET substrate is R. Inset: Photo image of controlling the strain applied on DPT by controlling the radius of the steel coil. **b**, Strain-dependent I-V characteristics of DPT under the sweeping bias between -3 V and +3 V. With the increase of compressive strain,

the reverse current increases rapidly, while the forward current decreases, which exhibits the asymmetric modulation of electrical transport by the piezotronic effect. **c**, Current as a function of applied strain under bias of -3, -2, -1, 0, 1, 2 and 3 V. **d**, **e** Resistance change ratio ($\Delta R/R_0$) (**d**) and current change ratio ($\Delta I/I_0$) (**e**) versus strain under various forward and reverse biases, respectively. **f**, Energy band diagrams showing the strain-induced piezotronic behavior in the DPT under opposite bias. I_{for} and I_{rev} represent forward and reverse current, respectively. **g**, Change of effective Schottky barrier height (SBH) as a function of strain as forwardly and reversely biased derived from the *I*-*V* curves in **b**. **h**, Gauge factor (GF) as a function of strain to evaluate the strain-sensing performance of DPT. The highest GF is about 8988.6.

(2) Dynamic current response of DPT to external strain and force (Figure 5 in the revised manuscript)

The description about **Figure 5** and the revised **Figure 5** are shown and marked in red as follows:

"We also measured the dynamic response characteristics of DPT ……the current signals remain nearly identical under the same stimulus and no obvious signal attenuation are observed throughout the test, …… **Fig. 5d** and **Fig. 5e** give the response time and recovery time of DPT for lateral strain sensing and vertical force sensing, respectively. In the lateral strain sensing mode, the response time of DPT slightly increases with the increased strain and the recovery time remains essentially unchanged, which have an average value of about 0.43 s. And in the vertical force sensing mode, the response and recovery time of DPT are both about 0.36 s. These values of time ……It can be found that the on/off ratio can be enhanced by increasing the stimulating signal. The maximum switching on/off ratio of DPT can reach 154.86 to vertical force and 386.57 to lateral strain. In conclusion, the DPT can work in two modes and exhibits reliable and rapid response as a strain sensor or force sensor. It should be noted that DPT cannot decouple the two sensing signals, which is the problem to be solved in the future research (**Supplementary Note 19**, **Supplementary Fig. 37**)."

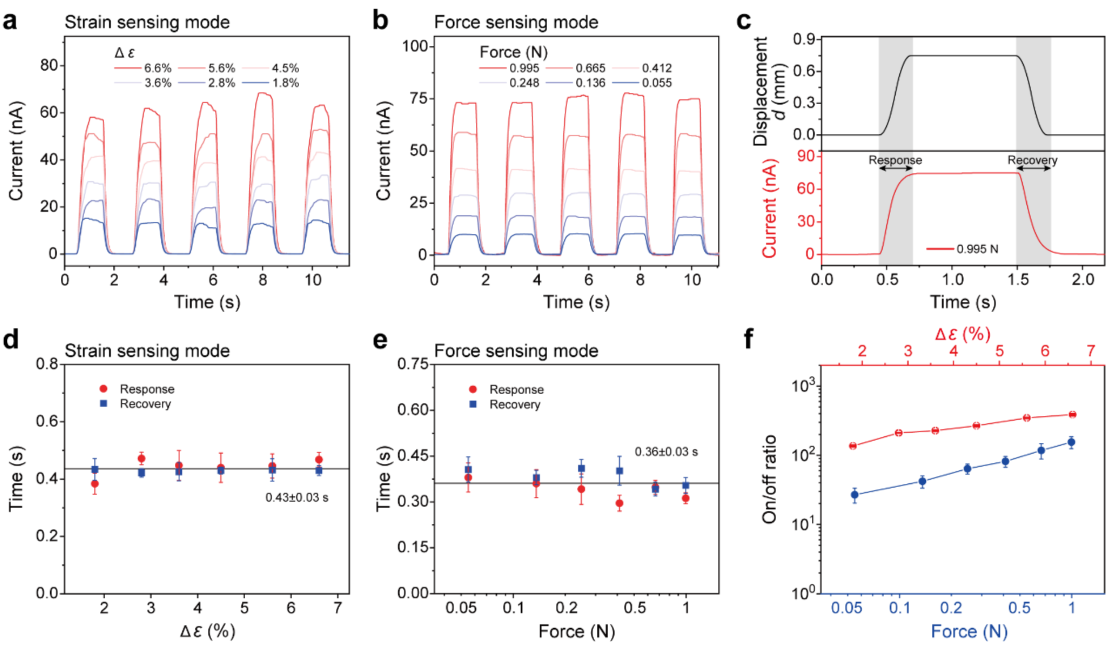

Figure R14 *(Figure 5 in the revised manuscript)*. **Dynamic current response of dual-modal piezotronic transistor (DPT) to periodic mechanical stimuli (lateral strain and vertical force). a**, **b** *I*-*t* curves of the current response to 5 on/off loading cycles under different strain changes (**a**) and forces (**b**) at a fixed bias of 3 V for strain sensing and force sensing, respectively. **c**, Detailed real-time current change with the displacement-controlled force of 0.995 N in **b**. The corresponding response time and recovery time are shown by the gray regions. **d**, **e** Statistical response time and recovery time of DPT in the lateral strain sensing mode (**d**) and the vertical force sensing mode (**e**) derived from **a** and **b**, respectively. The error bars denote standard deviations of the average value. **f**, On/off ratio of the DPT in its two working modes for lateral strain sensing and vertical force sensing derived from the current statistics in **a**, **b**.

Reference:

1. Teixeira, I. *et al.* Polydimethylsiloxane mechanical properties: a systematic review. *AIMS Materials Science* **8**, 952-973 (2021).
2. Moučka, R., Sedlačík, M., Osička, J. & Pata, V. Mechanical properties of bulk Sylgard 184 and its extension with silicone oil. *Scientific Reports* **11**, 19090 (2021).
3. Peng, J., Tomsia, A. P., Jiang, L., Tang, B. Z. & Cheng, Q. Stiff and tough PDMS-MMT layered nanocomposites visualized by AIE luminogens. *Nature Communications* **12**, 4539 (2021).

4. Wen, X., Li, D., Tan, K., Deng, Q. & Shen, S. Flexoelectret: an electret with a tunable flexoelectriclike response. *Physical Review Letters* **122**, 148001 (2019).
5. Palchesko, R. N., Zhang, L., Sun, Y. & Feinberg, A. W. Development of polydimethylsiloxane substrates with tunable elastic modulus to study cell mechanobiology in muscle and nerve. *PLoS ONE* **7**, e51499 (2012).
6. Wang, Z., Xiang, C., Yao, X., Le Floch, P., Mendez, J. & Suo, Z. Stretchable materials of high toughness and low hysteresis. *Proceedings of the National Academy of Sciences* **116**, 5967-5972 (2019).
7. Wang, Z. Polydimethylsiloxane mechanical properties measured by macroscopic compression and nanoindentation techniques. Master's Thesis, University of South Florida (2011).
8. Feng, R. & Farris, R. J. Linear thermoelastic characterization of anisotropic poly(ethylene terephthalate) films. *Journal of Applied Polymer Science* **86**, 2937-2947 (2002).
9. Zhang, S. L. & Li, J. C. M. Anisotropic elastic moduli and Poisson's ratios of a poly(ethylene terephthalate) film. *Journal of Polymer Science Part B: Polymer Physics* **42**, 260-266 (2004).
10. Wu, W. & Wang, Z. L. Piezotronics and piezo-phototronics for adaptive electronics and optoelectronics. *Nature Reviews Materials* **1**, 16031 (2016).
11. Zhou, Y. S. *et al.* Vertically aligned CdSe nanowire arrays for energy harvesting and piezotronic devices. *ACS Nano* **6**, 6478-6482 (2012).
12. Han, W. *et al.* Strain-gated piezotronic transistors based on vertical zinc oxide nanowires. *ACS Nano* **6**, 3760-3766 (2012).
13. Zhao, Z. *et al.* Piezotronic effect in polarity-controlled GaN nanowires. *ACS Nano* **9**, 8578-8583 (2015).
14. Wu, W., Wen, X. & Wang, Z. L. Taxel-addressable matrix of vertical-nanowire piezotronic transistors for active and adaptive tactile imaging. *Science* **340**, 952-957 (2013).
15. Liu, S. *et al.* Ultrasensitive 2D ZnO piezotronic transistor array for high resolution tactile imaging. *Advanced Materials* **29**, 1606346 (2017).

Reviewer #2 (Remarks to the Author):

Comments on NCOMMS-23-11350 titled Dual-modal piezotronic transistor for highly sensitive vertical force sensing and lateral strain sensing

The authors reported a force/strain sensor based on a single ZnO nano/microwire metal-piezoelectric semiconductor-metal structure. By innovatively introduce a micro-protrusion structure, the sensor could respond to both vertical force and lateral strain. The force sensitivity is up to 221.5 N^{-1} and the gauge factor is up to 7598.2. The idea is really interesting and publication in Nature Communications could be considered if the authors address the following comments.

Response:

We appreciate the reviewer's comments that "The idea is really interesting". And we also thank the reviewer for the valuable and constructive comments about our work.

Overall, the authors innovatively develop a structure design to integrate the vertical force sensing and lateral strain sensing functions together in just one sensor. The performance of the force/strain sensor is fascinating. Though the authors have reported several works about the piezotronic transistors previously, this work makes a significant progress in the field of force/strain sensing. I would recommend publication in Nature Communications once the authors properly address the abovementioned comments.

Response:

We are particularly grateful to the reviewer for the comments that "The performance of the force/strain sensor is fascinating" and "this work makes a significant progress in the field of force/strain sensing". This gives us a great incentive to move on.

Question #1

In the Introduction part, the authors state that "the use of the force/strain-induced piezoelectric polarization charges and corresponding potentials produced at the interfaces so as to linearly regulate the interface barrier height……". Why is the regulation of interface barrier height by piezoelectric polarization charges linear?

Related discussion or literatures should be added for better readability.

Response:

We thank a lot for the reviewer's nice suggestion. Here, we will give the reason why the regulation of piezoelectric polarization charges on the interface barrier height is a linear regulation in detail. It is hoped that the regulation mechanism of piezotronic effect can be clarified through the following discussion.

The metal-semiconductor contact is an important component in electronic devices, which can be simplified in terms of the charge distribution as shown in **Figure R15a** in the presence of a Schottky barrier [1-4]. The semiconductor side is assumed to be *n*-type, and the surface states and other anomalies are ignored for simplicity. Under strain, the generated piezoelectric charges at the interface not only change the height of the Schottky barrier, but also change its depletion layer width. Different from the method of changing the Schottky barrier height by introducing dopants at the semiconductor side, the piezopotential can be continuously tuned by strain for a fabricated device. Here, the piezoelectric potential distribution and related electric field in the Schottky junction can be calculated by simplifying the Poisson equation as follows:

$$-\frac{d^2\psi_i}{dx^2} = \frac{dE}{dx} = \frac{\rho(x)}{\varepsilon_s} = \frac{1}{\varepsilon_s}\left[qN_D(x) - qn(x) + qp(x) + q\rho_{piezo}(x)\right] \tag{1}$$

in which $N_D(x)$ is the donor concentration, $\rho_{piezo}(x)$ is the polarization charge density. Then W_{Dn} is defined as the width of the depletion layers on the *n*-type semiconductor side, and the electric fields in **Figure R15b** at different positions can be expressed by combining these equations:

$$E(x) = -\frac{q[N_D(W_{Dn} - x) + \rho_{piezo}(W_{piezo} - x)]}{\varepsilon_s}, \text{for } 0 \le x \le W_{piezo} \tag{2}$$

$$E(x) = -\frac{qN_D}{\varepsilon_s}(W_{Dn} - x), \text{for } W_{piezo} \le x \le W_{Dn} \tag{3}$$

Thus, the maximum electric field (E_m) at the interface (x=0) is given by

$$|E_m| = \frac{q(N_D W_{Dn} + \rho_{piezo} W_{piezo})}{\varepsilon_s} \tag{4}$$

In addition, the potential distribution $\psi_i(x)$ in **Figure R15c** can be expressed as:

$$\psi_i(x) = \psi_i(0) + \frac{q[N_D\left(W_{Dn} - \frac{x}{2}\right)x + \rho_{piezo}(W_{piezo} - \frac{x}{2})x]}{\varepsilon_s},$$

$$\text{for } 0 \leq x \leq W_{piezo} \tag{5}$$

$$\psi_i(x) = \psi_i(W_{piezo}) - \frac{qN_D}{\varepsilon_S}\left(W_{Dn} - \frac{W_{piezo}}{2}\right)W_{piezo} + \frac{qN_D}{\varepsilon_S}\left(W_{Dn} - \frac{x}{2}\right)x,$$

$$\text{for } W_{piezo} \leq x \leq W_{Dn} \tag{6}$$

Then, the built-in potential ψ_{bi} can be calculated as

$$\psi_{bi} = \frac{q}{2\varepsilon_s}[\rho_{piezo}W_{piezo}^2 + N_D W_{Dn}^2] \tag{7}$$

From the above equations, the change in the built-in potential derives from the piezoelectric charges generated through the applied strain. Here, the sign of the local piezoelectric charges is determined by tensile or compressive strain. It should be noted that the built-in potential without piezopotential can be expressed as

$$\psi_{bi0} = \frac{q}{2\varepsilon_s}N_D W_{Dn}^2 \tag{8}$$

If positive piezoelectric charges or potential is generated at interface by strain, the sign of the local piezoelectric charges is a positive value, and the built-in potential will change a value as follows

$$\psi_{bi} - \psi_{bi0} = \frac{q\rho_{piezo}W_{piezo}^2}{2\varepsilon_s} \tag{9}$$

In other words, the built-in potential (of Schottky barrier formed between metal

and n-type semiconductor just as set before) will increase as positive charges generated at interface.

Obviously, the piezoelectric potential can effectively tune the band of the junction corresponding to the Fermi levels. From equations (1) - (9), we can obtain the Fermi level (E_F) with a piezopotential at the interface of the Schottky junction as follow:

$$E_F = E_{F0} - \frac{q^2 \rho_{piezo} W_{piezo}^2}{2\varepsilon_s} \tag{10}$$

in which E_{F0} is the Fermi level in the absence of a piezopotential.

Here, the piezoelectric charges are assumed to function as a perturbation to the conduction band edge E_C at the interface of the Schottky junction. Thus, the change $\Delta\varphi_{piezo}$ in the Schottky barrier height (SBH) arising from the tuning of the piezoelectric charges can be calculated as

$$\varphi_{Bn} = \varphi_{Bn0} - \frac{q^2 \rho_{piezo} W_{piezo}^2}{2\varepsilon_s} \tag{11}$$

Thus, we can obtain that the density of the piezoelectric charges (ρ_{piezo}) linearly regulates the change of barrier height ($\Delta\varphi_{piezo}$).

$$\varphi_{Bn} - \varphi_{Bn0} = \Delta\varphi_{piezo} = -\frac{q \rho_{piezo} W_{piezo}^2}{2\varepsilon_S} \tag{12}$$

in which φ_{Bn} and φ_{Bn0} are the Schottky barrier height with and without the presence of piezoelectric charges, respectively.

Furthermore, the piezoelectric coefficient matrix of wurtzite structure ZnO can be written as [1, 2]

$$(e)_{ijk} = \begin{bmatrix} 0 & 0 & 0 & 0 & e_{15} & 0 \\ 0 & 0 & 0 & e_{15} & 0 & 0 \\ e_{31} & e_{31} & e_{33} & 0 & 0 & 0 \end{bmatrix} \tag{13}$$

As the major polarization axis of ZnO is the c-axis, normally in wurtzite crystals,

the longitudinal direction of a ZnO nano/microwire is along the *c*-axis. Based on the piezoelectric effect, the piezoelectric polarization of the ZnO nano/microwire at the contact in this case can be expressed as

$$P_z = e_{33}s_{33} = q\rho_{piezo}W_{piezo} \tag{14}$$

in which e_{33} is the piezoelectric constant, s_{33} represents the strain produced along the *c*-axis of ZnO nano/microwire.

From equation (14), it can be concluded that there is a linear correlation between piezoelectric polarization (P_z) and strain (s_{33}), as well as the density of piezoelectric polarization charges (ρ_{piezo}).

So,

$$P_z \propto s_{33} \propto \rho_{piezo} \tag{15}$$

According to equation (12), we can obtain that

$$\Delta\varphi_{piezo} \propto \rho_{piezo} \propto s_{33} \tag{16}$$

Thus, the force/strain-induced piezoelectric polarization charges and corresponding potentials produced at the interfaces linearly regulate the interface barrier height.

In order to improve the quality of the manuscript and facilitate the reference of researchers, we have added the above detailed derivation process and corresponding literatures to the revised Supplementary Information and marked them in red.

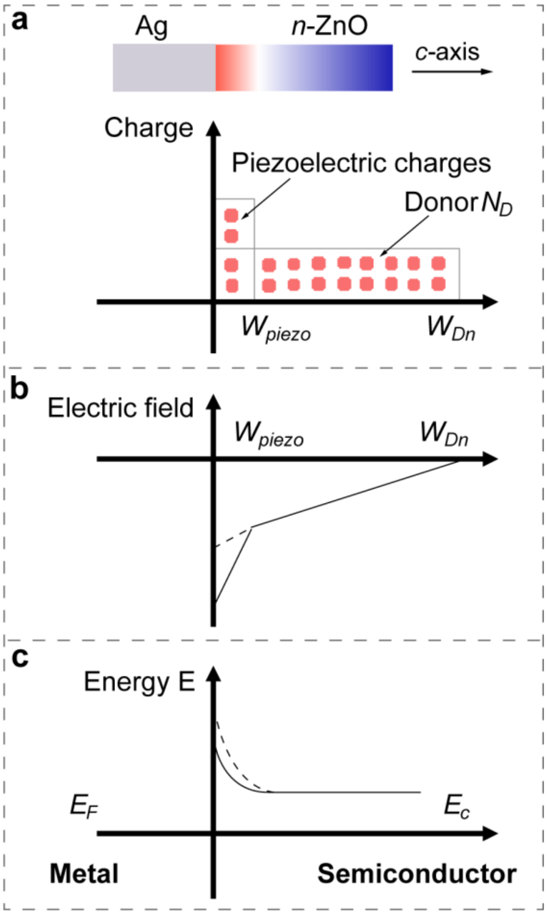

Figure R15. Ideal metal-semiconductor Schottky contact with the presence of piezoelectric charges at an applied voltage $V = 0$ (thermal equilibrium). a, Space charge distribution. **b**, **c**, Electric field (**b**) and energy band diagram (**c**) in the presence of piezoelectric charges. Dashed lines indicate the electric field and energy band in the absence of piezoelectric charges and the solid lines indicate the cases when a piezopotential is present in the semiconductor.

Question #2

For force/strain sensors, it is actually very interesting that typically force sensors should not respond to strain. Similarly, generally strain sensors should not respond to force. Could the authors discuss the crosstalk or possible relationship between vertical force sensing and lateral strain sensing of the demonstrated sensor? Maybe some useful literatures could be cited.

Response:

We thank the reviewer very much for this suggestion. It is really an interesting issue.

We have carried out a number of experiments but unfortunately found that at this stage we could not distinguish the response signals between the force sensing and the strain sensing. The main reason is that both the vertical force sensing mode and the lateral strain sensing mode utilize the deformation of ZnO nano/microwire to produce piezoelectric polarizations at interface to control the barrier height and electrical transport. We have tried our best but cannot distinguish whether the deformation of ZnO nano/microwire is caused by the vertical force or the lateral strain.

At current stage, the focus of this work is temporarily on realizing a piezotronic transistor with two working modes (vertical force sensing and lateral strain sensing) that can switch between the two working modes according to the needs of the scene. Since 2007, the incompatibility of the two structures (vertical structure and lateral structure) of piezotronic transistor has made it impossible to achieve a same piezotronic transistor to switch between two working modes, which is a long-standing challenge in the field of piezotronics. Dual-modal piezotronic transistor (DPT) in this work provides a good solution to overcome this challenge. Beside this innovation, we believe that if the two sensing signals can be furtherly decoupled no matter by principle design or signal processing, DPT will have a wider range of applications and greater significance. This issue proposed by the reviewer will be a very important step in the next stage of piezotronics. Further investigations are needed.

Although we cannot solve the problem of crosstalk at this stage, to our best understanding on piezotronic modification on energy band, we think the difference in the bending shapes of two sensing working modes (Note: In lateral strain sensing, the nano/microwire is bent or stretched and compressed as a whole; while in vertical force sensing, the middle of the nano/microwire bends downward.) is a possible breakthrough point. The different bending shapes means different piezoelectric potential distribution along the nano/microwire (**Figure R16**) and different energy band bending, which may cause different impedance information. Investigating the piezotronic modification of the capacitance-voltage characteristics may give us possible approach to distinguish the nano/microwire bending shapes, and decouple the vertical force and the lateral strain sensing signals by technologies like behavior-learned cognition algorithm [5]. However,

its complexity needs a lot of in-depth works in the further.

In order to avoid misunderstanding to readers, we have added above discussion into the revised supplementary information and corresponding content in the revised manuscript, and marked them in red.

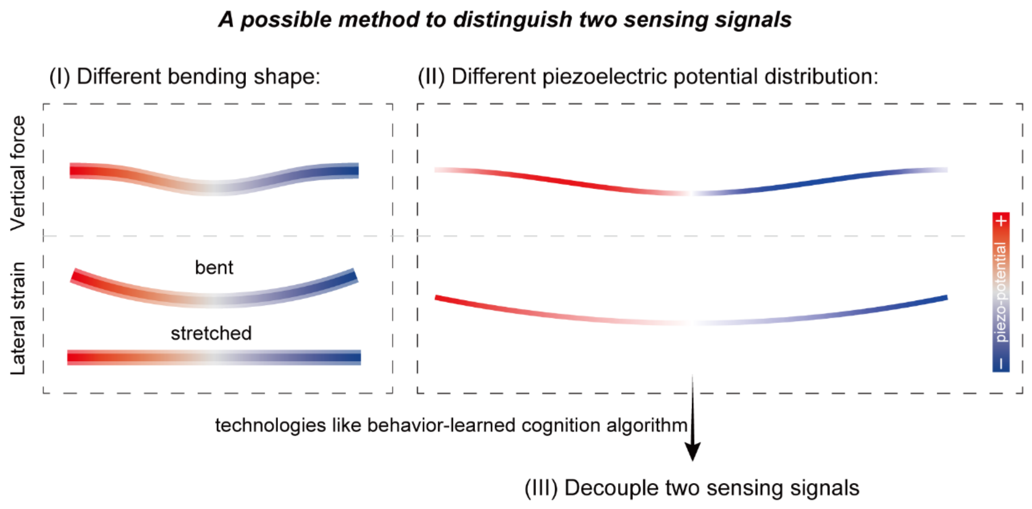

Figure R16. **A possible method to solve the problem of crosstalk in DPT.** Different bending shape (I) will make the piezoelectric potential distribution (II) different, which may lead to different impedance (III). Through investigating the piezotronic modification of the capacitance-voltage characteristics may we obtain the possible approach to decouple the two sensing signals by technologies like behavior-learned cognition algorithm.

Question #3

The lateral strain sensing mode is very similar to the traditional piezotronic transistor for strain sensing and piezoelectric nanogenerator, just as the authors repeatedly state. Compared to the traditional piezotronic transistor, why here is an additional PDMS layer introduced before the transfer of ZnO nano/microwire? What are its functions? If this additional PDMS layer is important to the high performance, what are the reasons? Besides, do the thicknesses of both two PDMS layers (one as substrate and the other as encapsulation) affect the force/strain sensing performance?

Response:

We thank the reviewer very much for these constructive comments. To clarify the role

of PDMS clearly, we divide the above valuable comments into two main questions and will answer the two questions in turn as follows, and finally make a summary.

(1) Compared to the traditional piezotronic transistor, why here is an additional PDMS layer introduced before the transfer of ZnO nano/microwire? What are its functions? If this additional PDMS layer is important to the high performance, what are the reasons?

Response: We thank the reviewer for this nice comment. The additional PDMS layer introduced before the transfer of ZnO nano/microwire has two functions. Its main function is to meet the needs of vertical force detection and provide a soft substrate for the downward deformation/bending of nano/microwires in the vertical force sensing mode. As shown in **Figure R17a** and **R17b**, the mechanism of the dual-modal piezotronic transistor (DPT) for the vertical force sensing mode is actually inspired by the suspension bridge. When the middle of the rope in suspension bridge is subjected to a vertical force, the rope will deform downward and produce a tensile force. In this way, the vertical force applied on the nano/microwire can be effectively converted into a lateral tensile force of the nano/microwire, so as to realize the sensing of the vertical force by the nano/microwire. (Note: A more in-depth and detailed discussion about the research idea on the design of DPT is in the follow-up response to **Question #10**.) In other words, the vertical force sensing depends on the downward deformation/bending of the nano/microwire, which induces the elongation of the nano/microwire. In a structure with a bottom PDMS layer as shown in **Figure R17c**, the vertical force can downward bend and elongate the nano/microwire due to the soft nature of the bottom PDMS. While, in a structure without bottom PDMS as shown in **Figure R17d**, the nano/microwire would not response to the vertical force. It can be found that the bottom PDMS plays an important role in the vertical force sensing mode of DPT and is a necessary condition for DPT to possess high-performance vertical force sensing, which is also the main reason why we design the bottom PDMS.

Another additional function of the bottom PDMS is to produce greater strain on the nano/microwire when the substrate bends and protects the nano/microwire from being easily damaged in the lateral strain sensing mode of DPT. In the traditional piezotronic device with a lateral structure, the piezoelectric nano/microwire lies flat on

a flexible substrate (PET), with both ends tightly bonded to the substrate as illustrated in upper side of **Figure R18a** [6]. Upon the application of external stimuli, the mechanical bending of the substrate will create the uniaxial strain and the corresponding piezoelectric potential along the *c*-axis of the nano/microwire (the lower side of **Figure R18a**). The strain of nano/microwire can be expressed by

$$\varepsilon = -\frac{h_{PET}/2}{R} \tag{17}$$

in which R is the curvature radius of the bending PET substrate. Similar to the traditional lateral structure, the strain of nano/microwire in the DPT is directly related to the thicknesses of the PET substrate (h_{PET}) and the bottom PDMS layer ($h_{PDMS,bottom}$) for lateral strain sensing (**Figure R18b**), which can be expressed as the following formula.

$$\varepsilon = -\frac{h_{PDMS,bottom} + h_{PET}/2}{R} \tag{18}$$

By comparing formulas (17) and (18), we can see that the strain induced in the nano/microwire by bending substrate can be considerably large due to the existence of the bottom PDMS layer compared with the traditional lateral structure. Also, according to our years of experimental experience in piezotronics, since the nano/microwire is not directly fixed on PET, but have the bottom PDMS as a buffer layer, the nano/microwire is more resistant to fracture.

It should be noted that the introduction of the bottom PDMS is mainly due to the consideration of the vertical force sensing mode of DPT, and its function in the lateral strain sensing mode of DPT is incidental.

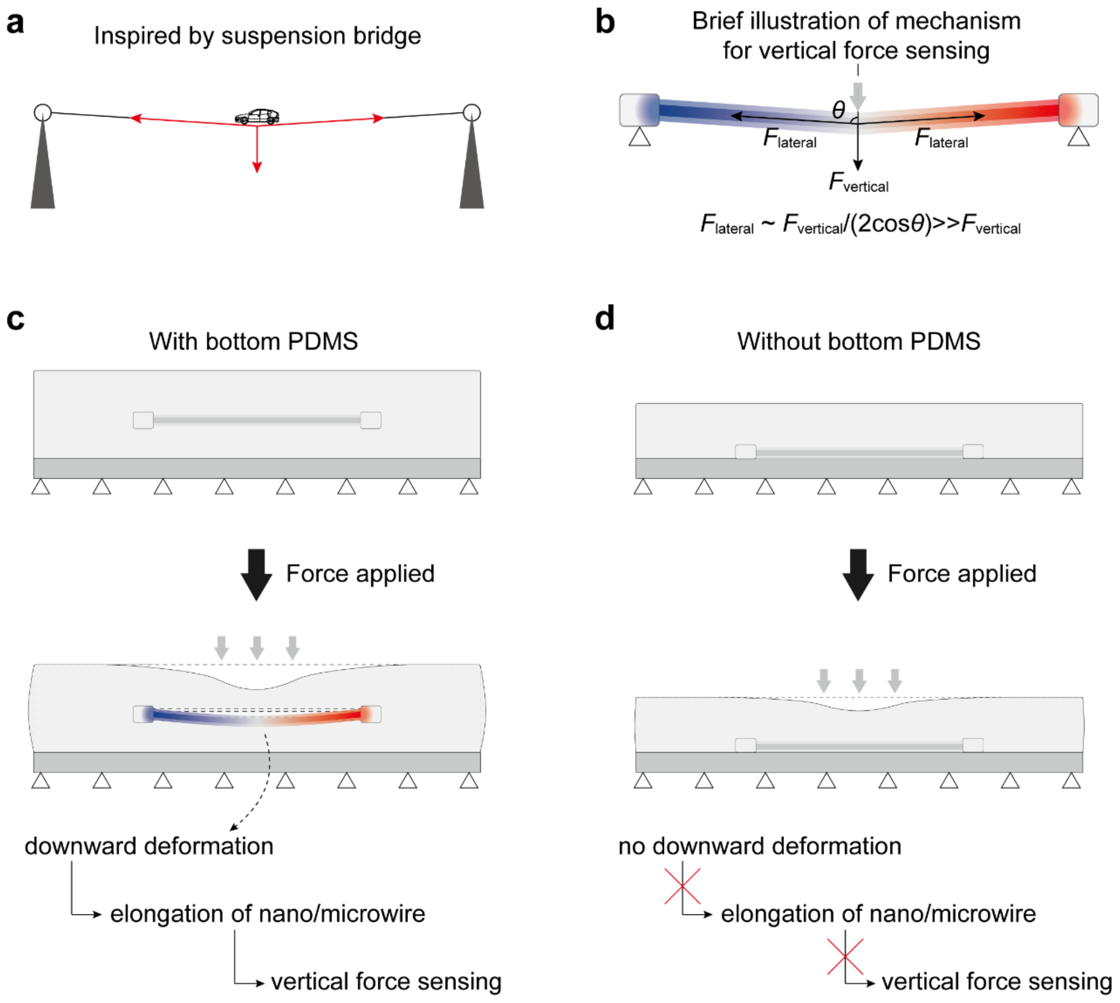

Figure R17. Main function of the bottom PDMS in vertical force sensing mode of DPT. a, b, Mechanism for vertical force sensing of DPT (**b**) is inspired by the suspension bridge (**a**), in which the vertical force can be transformed to the elongation and tensile force of the rope or the nano/microwire. **c**, In the structure with a bottom PDMS layer, the vertical force can downward bend and elongate the nano/microwire due to the soft nature of the bottom PDMS. **d**, In the structure without bottom PDMS, the nano/microwire would not response to the vertical force. It should be noted that to better exhibit the role of the bottom PDMS, the microprotrusion in DPT is not drawn.

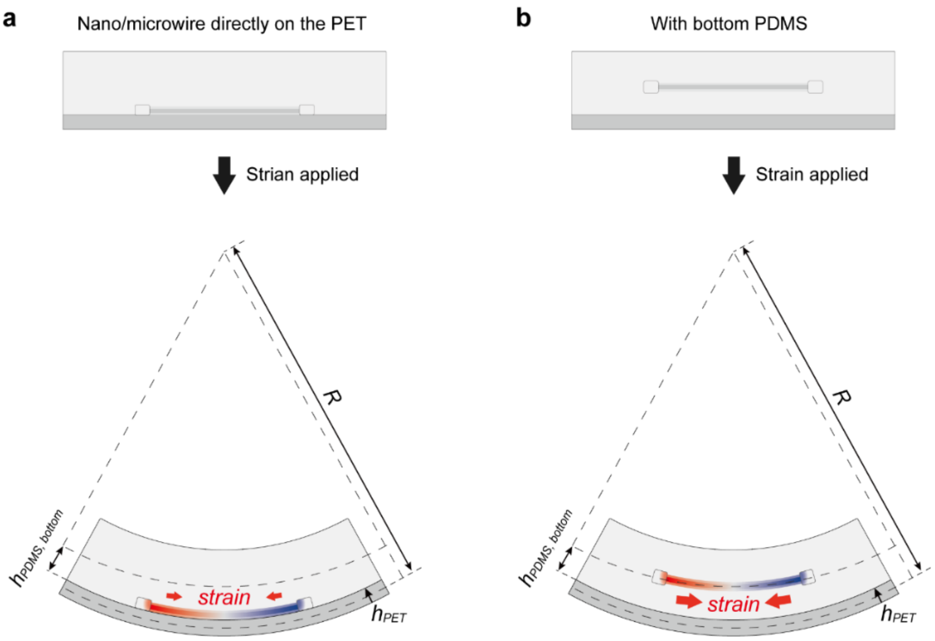

Figure R18. The additional function of the bottom PDMS in the lateral strain sensing mode of DPT. a, In the structure with nano/microwire directly on the PET, a small lateral strain will be produced along the length direction of the nano/microwire under the application of a compressive stimuli/strain. **b**, In the structure with a bottom PDMS layer, a relatively large strain will be induced in the nano/microwire compared with that in the structure without bottom PDMS layer in (**a**). R represents the curvature radius of the bending PET substrate. $h_{PDMS,bottom}$ and h_{PET} are the thickness of the bottom PDMS and PET substrate, respectively.

(2) Besides, do the thicknesses of both two PDMS layers (one as substrate and the other as encapsulation) affect the force/strain sensing performance?

Response: We thank the reviewer very much for this interesting and valuable suggestions. As discussed above, the introduction of bottom PDMS (the one as substrate) is mainly due to the consideration of the vertical force sensing mode of DPT. We will focus on the influence of the thickness of both two PDMS layers on the vertical force sensing performance of DPT. (In fact, in the lateral strain sensing mode, the thickness of the bottom PDMS layer mainly affects the magnitude of the nano/microwire strain when the PET is bent, as expressed in above formula (18); the top PDMS layer is mainly used as encapsulation and its thickness has little effect on the lateral strain sensing.)

① *Influence of the bottom PDMS thickness on the vertical force sensing performance*

To investigate the influence of the thicknesses of PDMS layers on the sensing performance of the DPT, the finite element method (FEM) simulation is established to analyze the structure of the DPT in vertical force sensing mode. **Figure R19a** schematically shows the side view (xy plane) of the DPT with two soft PDMS layers, in which $h_{PDMS,bottom}$ and $h_{PDMS,top}$ represent the thicknesses of the bottom PDMS layer (as substrate) and the top PDMS layer (as encapsulation), respectively. Meanwhile, other structural parameters are set as constant values, such as the microprotrusion (1 mm in width and 1 mm in height), the ZnO microwire (1 mm in length) and the PET substrate (0.15 mm in thickness and 30 mm in length).

With a constant thickness of the top PDMS layer ($h_{PDMS,top} = 0.5\ mm$), the displacement distribution profiles of the DPTs and the corresponding outlines of bended nano/microwires in response to different displacements (d) applied on the DPTs with various thicknesses of the bottom PDMS layers ($h_{PDMS,bottom}$) ranged from 0.425 mm to 1.625 mm are demonstrated in **Figure R19b** and **R19c**, respectively. It can be obviously observed in **Figure R19c** that the magnitude of the overall displacement of the nano/microwire in the vertical direction (the y-axis) gradually increases with thicker bottom PDMS. Also, it can be found that the downward bending degree of the nano/microwire increases as the bottom PDMS thickness increases. As expected, the tendency of decrease in the bending deformation and the overall displacement of the nano/microwire with respect to the reduced thickness of the bottom PDMS layer should be attributed to the enhanced substrate clamping effect in the thinner PDMS layer. The obtained curves of nano/microwire strain versus downward displacement (d) with the thickness in the range of 0.225 mm to 1.625 mm are plotted in **Figure R19d**, which also indicates that the strain level of the nano/microwire increases with the bottom PDMS thickness. Under the given displacement ($d = 0.8\ mm$), the nano/microwire strain increases approximately linearly and then tends to a saturated value (~1.6%) when the thickness of the bottom PDMS layer exceeds 1.225 mm, as shown in **Figure R19e**. It is worth noting that although the strain increases with the increase of the bottom PDMS thickness, the thickness should not be too large considering the practical

application of the device.

② *Influence of the top PDMS thickness on the vertical force sensing performance*

The influence of the top PDMS layer on the vertical force sensing performance is also studied by using the FEM model of the DPT with a constant thickness of the bottom PDMS layer ($h_{PDMS,bottom} = 0.825\ mm$), while the thickness of the top PDMS layer ($h_{PDMS,top}$) is variable from 0.15 mm to 1 mm (**Figure R20**). **Figure R20b** shows the displacement distribution profiles obtained under different downward displacements (d), which indicates the device deformation with the top PDMS layers at the thicknesses ($h_{PDMS,top}$) of 0.15 mm, 0.50 mm and 1.00 mm, respectively. It is worth noting that a thinner encapsulation layer (i.e., the top PDMS layer) leads to a diminution of the distance between the nano/microwire and the microprotrusion, and hence induces a larger bending deformation of the microwire under vertical force condition, as shown in **Figure R20c**.

Figure R20d gives the calculated strain of the bended nano/microwire as a function of the downward displacement (d) in the DPTs with different thicknesses of the top PDMS layers ($h_{PDMS,top}$). It can be seen clearly from **Figure R20e** that when a 0.15-mm-thick PDMS layer is employed for encapsulation, the maximum strain level of the microwire under the downward displacement d of 0.8 mm can reach about 3.0%, which then begins to drop dominantly with increasing thickness of the top PDMS layer. On the other hand, the top PDMS layer is introduced as encapsulation for insulation and protection, which should achieve a robust thickness in order to maintain the physical stability of the ZnO nano/microwire. Therefore, utilizing the PDMS layer with appropriate thickness can effectively control the force transfer process to induce the sufficiently large deformation in the nano/microwire, while maintaining high sensitivity as well as excellent reliability of the DPT for vertical force sensing.

Actually, a function of the top and bottom PDMS layers together is to protect the lateral nano/microwire from breaking when subjected to the vertical force. Our earliest device design is based on the structure of suspended nano/microwires just like the one in **Figure R17b**, but through a large number of experiments, it is found that the lack of top and bottom PDMS layers will cause the local stress of the device to be too large and

very easy to damage.

In summary, the introduction of the bottom PDMS layer is mainly to meet the needs of vertical force sensing of DPT, which can improve the nano/microwire strain caused by external force or strain to a certain extent; the introduction of the top PDMS is mainly to encapsulate and protect the nano/microwire, with the best thickness to improve the vertical force sensing performance of DPT.

We thank the reviewer for this very constructive suggestion again. To improve the quality of the manuscript and provide a reference for readers, we have added the above discussions into the Supplementary Information and marked them in red.

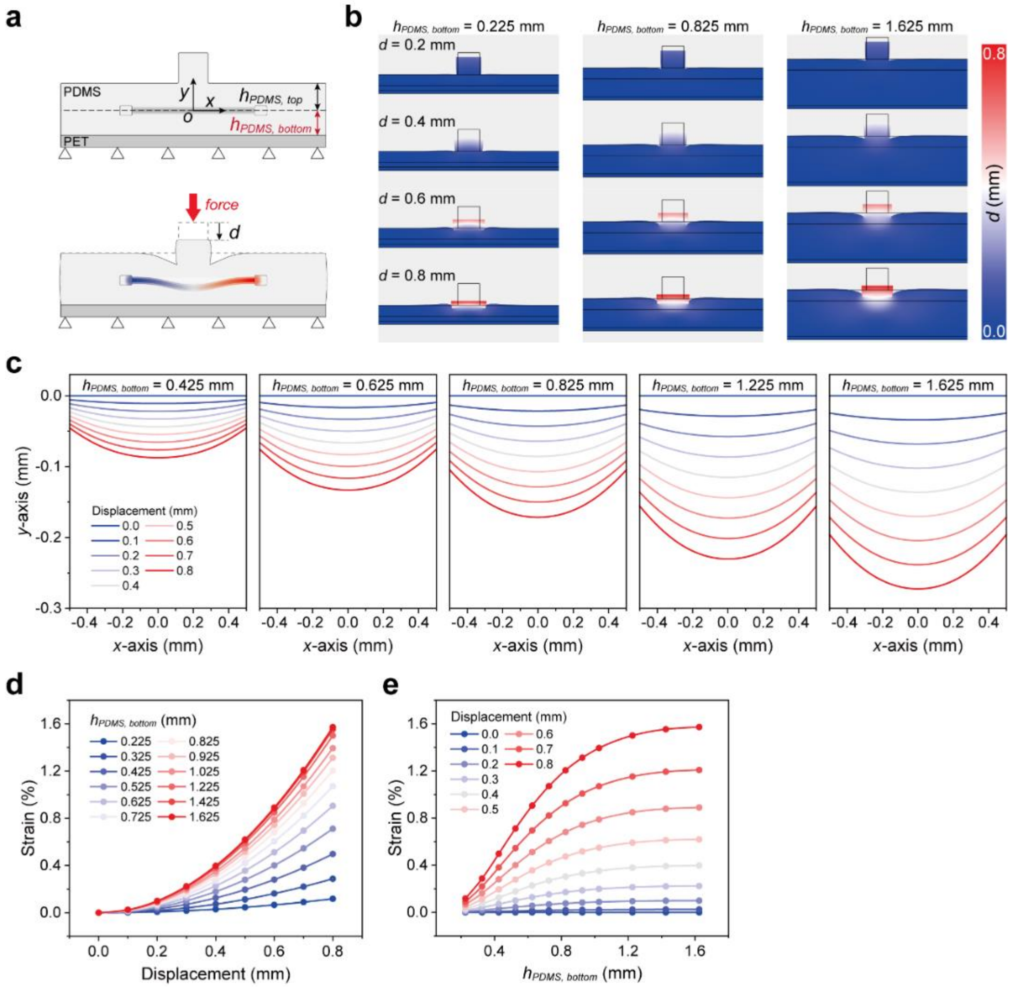

Figure R19. Influence of the thickness of bottom PDMS layer ($h_{PDMS,bottom}$) on the performance of the DPT for vertical force sensing by FEM simulations. a, Schematic illustration of the DPT with the detailed structure of two PDMS layers in

vertical force sensing mode. $h_{PDMS,top}$ and $h_{PDMS,bottom}$ represent the thicknesses of the top PDMS layer and the bottom PDMS layer, respectively. **b**, **c** Displacement distribution profiles of the DPTs (**b**) and the corresponding outlines of bended nano/microwires (**c**) with various $h_{PDMS,bottom}$ in response to different downward displacements (d). **d**, Calculated strain of nano/microwires of the DPTs with different $h_{PDMS,bottom}$ as a function of d. **e**, The curves of nano/microwire strain versus $h_{PDMS,bottom}$ under different d.

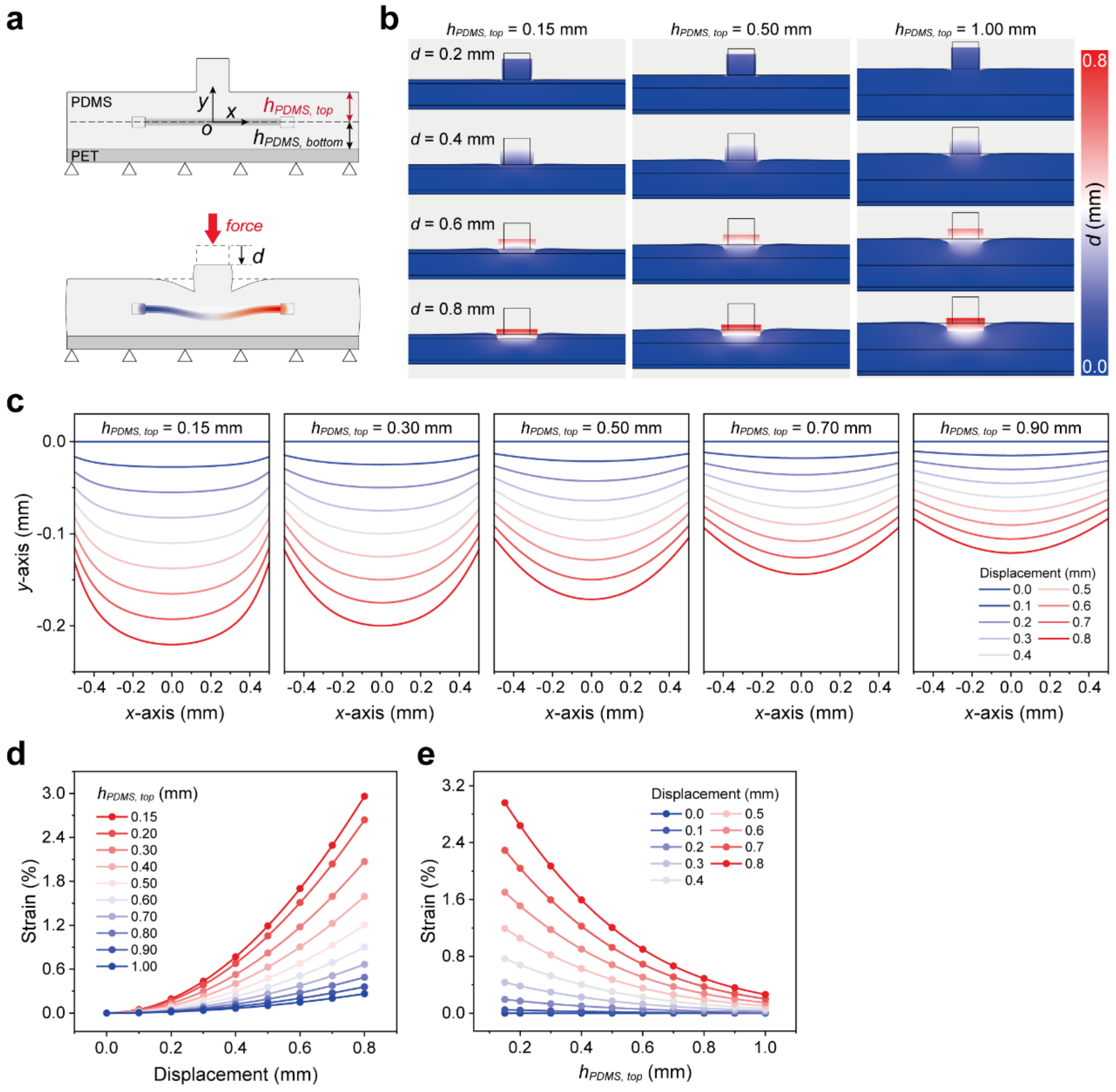

Figure R20. Impact of the thickness of top PDMS layer ($h_{PDMS,top}$) on the performance of the DPT for vertical force sensing by FEM simulations. a, Schematic illustration of the DPT with the detailed structure of two PDMS layers in vertical force sensing mode. $h_{PDMS,top}$ and $h_{PDMS,bottom}$ represent the thicknesses of

the top and bottom PDMS layer, respectively. **b**, **c** Displacement distribution profiles of the DPTs (**b**) and the corresponding outlines of bended microwires (**c**) with various $h_{PDMS,top}$ in response to different downward displacements (d). **d**, The calculated strain of nano/microwires of the DPTs with different $h_{PDMS,top}$ as a function of d. **e**, The curves of nano/microwire strain versus $h_{PDMS,top}$ under different d.

Question #4

Line 148-149. How to simulate the change of ZnO nano/microwire length and calculate the strain? How to determine the accuracy since the author claim "the strain of ZnO nano/microwire can be accurately calculated"?

Response:

We thank the reviewer very much for the comment and suggestion. In order to simulate the bending deformation of ZnO nano/microwire in response to the applied force, we establish a 2D finite element model of the DPT under plane stress condition, and assume that a nano/microwire with original length $L_{NW} = 1\ mm$ is embedded in the interface between two PDMS layers. According to the FEM simulation result, we can obtain the information relating to the mechanical deformation of the DPT under vertical force condition, such as the outline curve of bended microwire in the displacement distribution profile.

With application of the definite integral, if a smooth curve with equation $y = f(x)$, $a \leq x \leq b$, is traversed exactly once as x increases from a to b, then its length l can be determined from the following formula:

$$l = \int_a^b \sqrt{1 + \left(\frac{dy}{dx}\right)^2}\, dx \tag{19}$$

Considering the very small size of the nano/microwire compared to PDMS and PET, we can estimate the change of the nano/microwire length (Δl) based on the integral for the curve length derived from the outline of bended nano/microwire. So, the axial strain of the nano/microwire along length direction can be calculated as

$$\varepsilon = \frac{\Delta l}{l_0} = \frac{l - l_0}{l_0}$$

(20)

where l_0 and l represent the original length of the microwire in the initial state ($l_0 = 1\ mm$) and the calculated length of the nano/microwire under bending, respectively.

For the convenience of calculation, we will not consider the negligible effect of the diameter of the ZnO nano/microwire in the FEM simulations, indicating the calculated result of the nano/microwire strain is just an approximation to the quantitative evaluation. To make it more appropriate, we have carefully revised the sentences in the manuscript, which should be replaced by "the strain of ZnO nano/microwire can be approximately evaluated", and then marked it in red. Thanks again for the reviewer's kind reminder.

Question #5

The authors systematically investigate and optimize the width of PDMS micro-protrusion structure mainly for vertical force sensing. Would the length and thickness affect the vertical force sensing? Besides, does the PDMS micro-protrusion structure affect the lateral strain sensing?

Response:

We thank the reviewer very much for the detailed and nice suggestions. In order to reveal the impact of the microprotrusion on the device performance more comprehensively, we have carried out more detailed simulations on the microprotrusion as follows.

(1) Would the length and thickness affect the vertical force sensing?

Thanks for the reviewer's nice suggestion. Considering the extremely small diameter of the ZnO nano/microwire (usually in the micron scale, such as about 6.5 μm) compared with the length of the PDMS microprotrusion (millimeter scale), the influence of the microprotrusion length on the microwire deformation under vertical force condition is negligible in this work. However, the height (also can be called thickness) of the PDMS microprotrusion ($h_{protrusion}$) actually has an important impact

on the performance of the DPT for vertical force sensing. Here, the microprotrusions with various heights ranging from 0.2 mm to 2.0 mm are introduced in the FEM models of the DPTs while keeping other structural parameters unchanged. We model the microprotrusion with width $w = 1\,mm$, and the structure ratio remains constant ($w/L_{NW} = 1$). Besides, the thicknesses of the PET substrate, the top and bottom PDMS layers are set as $h_{PET} = 0.15\,mm$, $h_{PDMS,top} = 0.5\,mm$ and $h_{PDMS,bottom} = 0.825\,mm$, respectively.

For the DPT in vertical force sensing mode, the applied force can be controlled by changing the downward displacement (d) on the top edge of the microprotrusion. Note that the DPT with higher microprotrusion could allow it to adapt to much larger downward displacement (d), whereas the displacements (d) are relatively limited for those with smaller microprotrusion heights ($h_{protrusion}$). So, we define the ratio between the downward displacement (d) and the microprotrusion height ($h_{protrusion}$) as a new parameter named downward displacement level ($d/h_{protrusion}$) in the loading process for the DPT with different microprotrusion heights. As demonstrated by the FEM analysis results in **Figure R21a-b**, the DPT with the microprotrusion height ($h_{protrusion}$) of 0.25 mm will exhibit extremely small bending deformation of the nano/microwire with application of a low displacement range ($d = 0{\sim}0.2\,mm$). When the microprotrusion height ($h_{protrusion}$) rises up to 2.0 mm, higher range of the downward displacement ($d = 0{\sim}1.6\,mm$) will lead to an obvious increase in the overall displacement of the nano/microwire and then induce a larger bending deformation (**Figure R21b**).

Figure R22a shows the typical strain curves of the nano/microwires in the DPTs with varying microprotrusion heights ($h_{protrusion}$) from 0.25 mm to 2.0 mm, which indicates that the nano/microwire strain shows a conspicuous monotonic increase as a function of the displacement level ($d/h_{protrusion}$). It can be seen from **Figure R22b** that, when the height of the microprotrusion is relatively small ($h_{protrusion} \leq 1.0\,mm$), higher microprotrusion could induce larger bending deformation in the nano/microwire, corresponding to a rapidly increased strain level. However, as the microprotrusion height ($h_{protrusion}$) further increases, a substantial amount of the downward

displacement (d) would be inevitably dissipated to compress the microprotrusion with the additional height, which causes the strain growth of the bended microwire to slow down gradually (**Figure R22b**).

Moreover, to evaluate the mechanical properties of the DPTs with different microprotrusion heights ($h_{protrusion}$), we systematically investigate the corresponding pressure applied on the DPTs under different levels of displacements (d). It is worth noting that the DPT exhibits a good linear relationship between the applied pressure and the displacement level ($d/h_{protrusion}$) for all cases, while the maximum measurable pressure rises rapidly at first and the trend is subsequently moderated with the increase of the microprotrusion height ($h_{protrusion}$), as shown in **Figure R22c-d**. On the other hand, when a relatively small pressure (0.2 MPa) is applied, the strain level of the nano/microwire will decrease drastically due to a slight increase in the microprotrusion height ($h_{protrusion}$), falling below 0.18% from 0.22% (**Figure R22e**). It can be obviously observed in **Figure R22f** that the tendency of decrease in the bending deformation of the microwire should be attributed to the decline of the induced displacement level ($d/h_{protrusion}$) with increasing microprotrusion height ($h_{protrusion}$) under a pressure of 0.2 MPa, indicating the small pressure is insufficient to drive the microprotrusion with a large height to bend the nano/microwire.

Therefore, these findings suggest the increase of the microprotrusion height ($h_{protrusion}$) could achieve a larger pressure response range but a much lower sensitivity with the minimum detectable pressure.

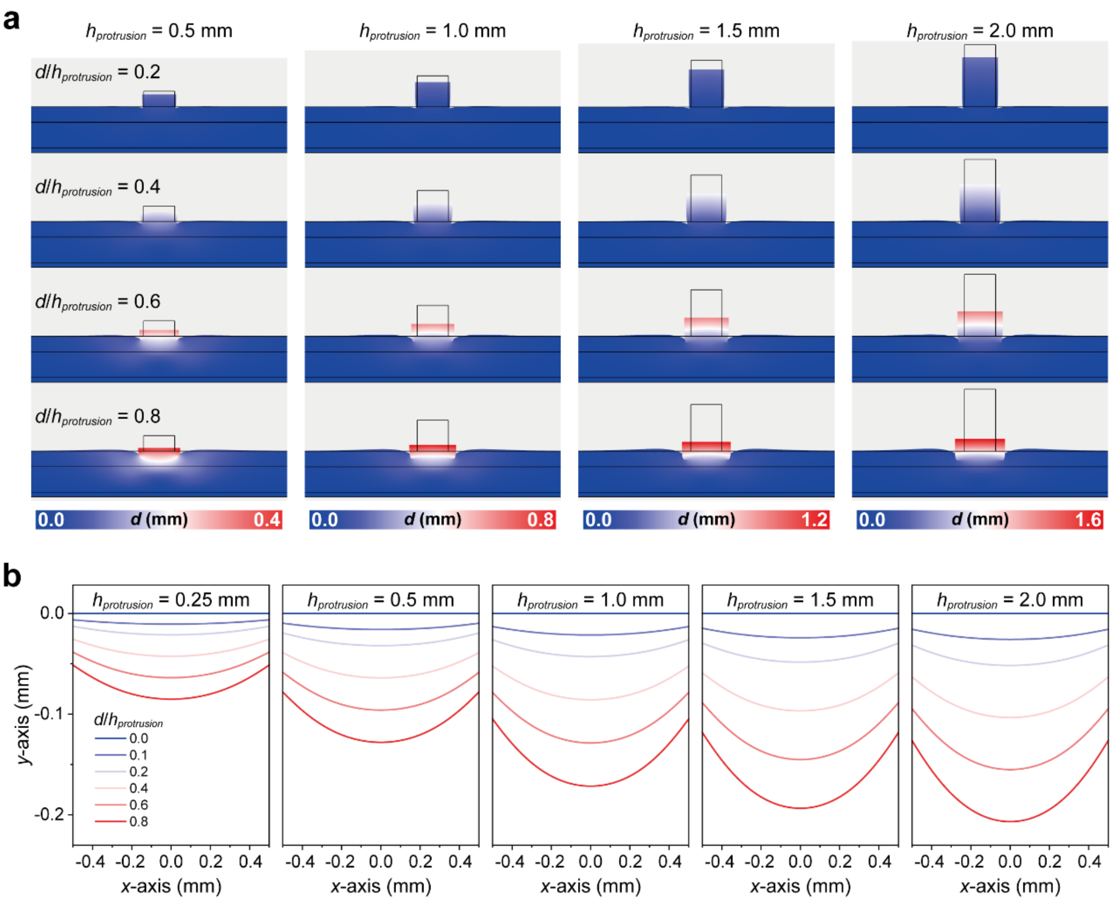

Figure R21. The FEM simulation results for the DPTs with different microprotrusion heights ($h_{protrusion}$) in vertical force sensing mode. a, b, Displacement distribution profiles of the DPTs (**a**) and the corresponding outlines of bended microwires (**b**) with various heights of the microprotrusions ($h_{protrusion}$) ranging from 0.25 mm to 2.00 mm under different downward displacement levels ($d/h_{protrusion}$ = 0~0.8).

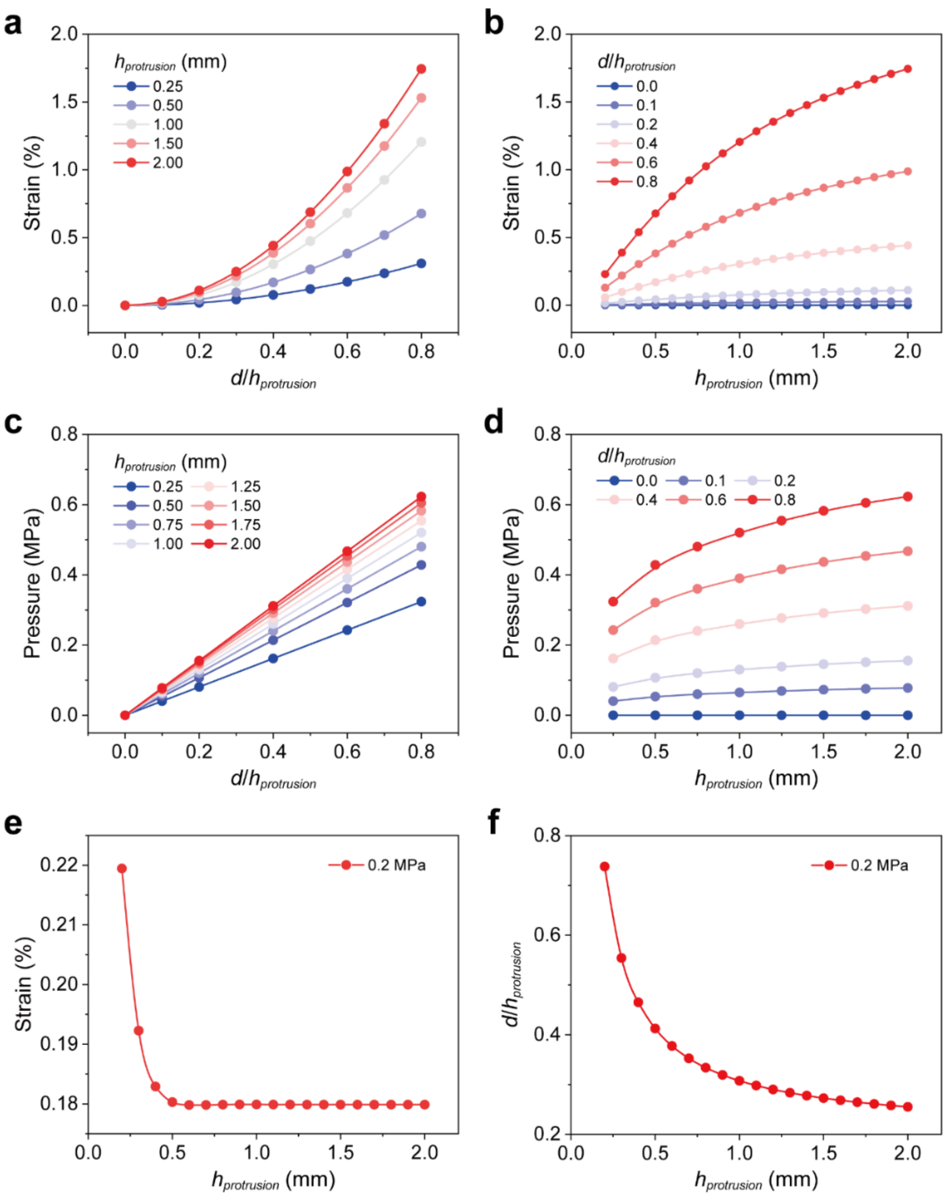

Figure R22. Impact of the microprotrusion height on the DPT performance for vertical force sensing by the FEM simulation. a, Curves of nano/microwire strain versus the displacement level ($d/h_{protrusion}$) for DPTs with various microprotrusion heights ($h_{protrusion}$). **b**, Dependence of the simulated strain with respect to the microprotrusion height under different displacement levels. **c**, Corresponding pressure applied on the top edge of the microprotrusion as a function of the displacement level with different microprotrusion heights. **d**, The relationship between the applied pressure and the microprotrusion height in response to different displacement levels. **e**, **f**, Dependence of the calculated strain (**e**) and the corresponding displacement level (**f**) with respect to the microprotrusion height under a pressure of 0.2 MPa.

(2) Besides, does the PDMS micro-protrusion structure affect the lateral strain sensing?

We thank for the reviewer's suggestion. For the traditional piezotronic transistor with the lateral structure, the induced strain in the ZnO nano/microwire can be directly sensed through the bending of the flexible substrate. In fact, considering that the ratio of the microprotrusion area to the substrate one is negligible, the microprotrusion in the DPT should not be necessary factors that affect the overall deformation of the device in lateral strain sensing mode. To evaluate the influence of the introduction of the microprotrusion structure on the mechanical property and the sensing performance, further simulations are carefully performed to qualitatively verify the sensitivity of the DPT in response to a lateral strain.

The typical 2D finite element models are constructed for the simulations with the consideration of the microprotrusion structure. As the demonstration of the loading process in the DPT for lateral strain sensing, we create the proper boundary condition to the neutral layer in the PET substrate to induce the bending deformation. Here we introduce an assumption that the length of the substrate (L) remains unchanged while the distance between the two ends of the substrate ($L - \Delta L$) decreases, leading to various curvature of the compressible PDMS layers in the model (**Figure R23a**). It can be quantitatively observed that the upper part of the PDMS layer at the center position could be gradually compressed as the increasing curvature of the substrate, as demonstrated by the strain distribution (ε_{11}) profiles under different bending conditions in **Figure R23b** and **R23c** for the DPTs with and without the microprotrusion, respectively. Note that in comparison to the piezotronic transistor without the microprotrusion (**Figure R23e**), the existence of the microprotrusion in the DPT would create the localized high magnitude strain around the surface of the PDMS layer, which is more inclined to contribute to the inhomogeneous deformation of the nano/microwire under bending, as shown in **Figure R23d**. Subsequently, the calculated strain curves of the nano/microwires corresponding to the DPTs with and without the microprotrusion under the same loading conditions are obtained in **Figure R23f**. It can be found that when subjected to the bending deformation at the same curvature, the nano/microwire strain for the DPT with the microprotrusion may slightly increase in the simulated strain

range (0%~4%) compared to that without the microprotrusion, which should benefit from the stress concentration behavior around the microprotrusion. The above results validate that, as expected, the addition of the microprotrusion would not significantly affect the sensitivity of the DPT to the lateral strain.

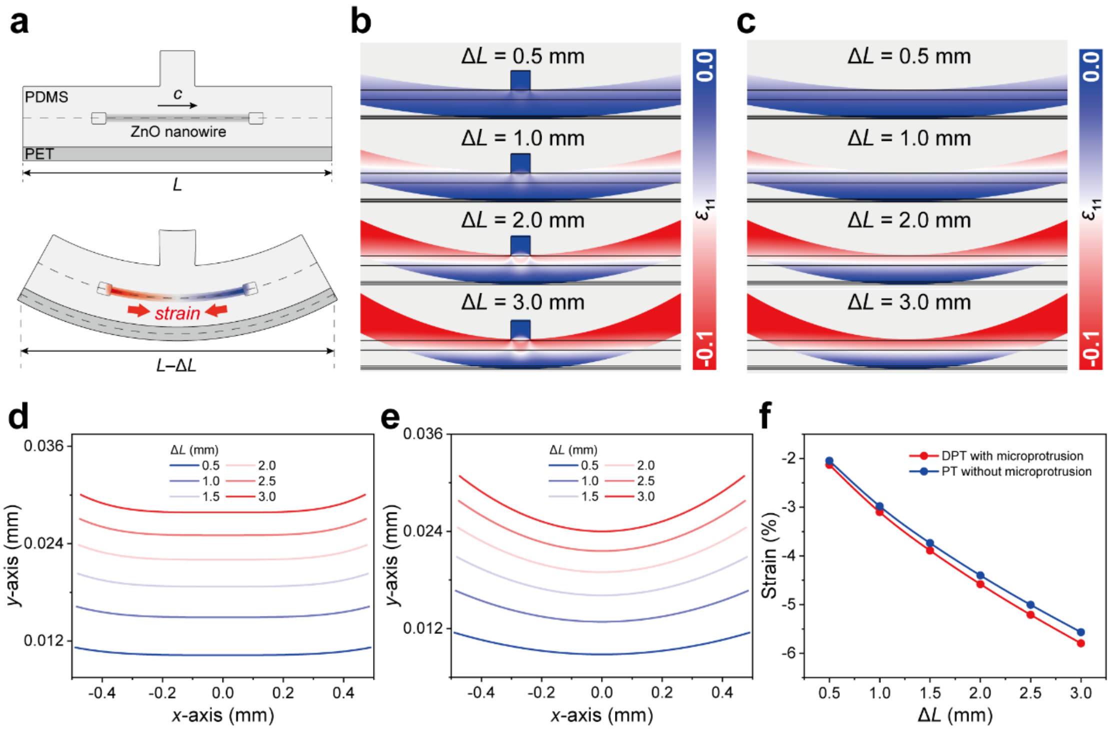

Figure R23. Impact of the structure of the microprotrusion on the performance of the DPT for lateral strain sensing by the FEM simulation. a, Schematic diagrams of the side view of the DPT in lateral strain sensing mode. The original length of the substrate is L; the distance change between the two ends of the bending substrate is ΔL. **b**, **c**, Strain distribution (ε_{11}) profiles under bending of the DPT with (**b**) and without (**c**) the microprotrusion in response to different distance changes ΔL. **d**, **e**, Corresponding outlines of bended nano/microwires in the case with (**d**) and without (**e**) the microprotrusion under bending derived from **b** and **c**, respectively. **f**, Calculated nano/microwire strain as a function of the distance change (ΔL) applied under lateral strain condition for the DPT with and without the microprotrusion.

Question #6

To my best understanding, the alignment of ZnO nano/microwire length's direction with the PDMS substrate's length direction is of great importance. And the position of PDMS micro-protrusion structure is also of great importance. I am wondering how do the author precisely control the alignment of ZnO nano/microwire and position of PDMS micro-protrusion structure?

Response:

We thank the reviewer very much for this suggestion. Precisely controlling the alignment of nano/microwire and the position of microprotrusion are importance to the performance of DPT. Here, we introduce the step-by-step fabrication of DPT in detail.

In this work, the device is constructed on the flexible PET substrate (0.15 mm in thickness) with a soft adherent layer of PDMS. Before the preparation of PDMS layer, a clean PET film (50×50 mm^2) should first be tightly attached to the bottom of the lab dish. The mixture of PDMS (a prepolymer and a curing agent default mixing weight ratio 10:1) is then poured onto the PET substrate (**Figure R24a**), followed by 30 min of degassing in vacuum desiccator. When the thermal curing process is done, the PDMS layer with uniform thickness (~0.825 mm) would be formed on a PET film.

For the formation of the device, a single ZnO nano/microwire (its length is usually larger than 1.5 mm) could be physically picked up and transferred onto the as-fabricated PDMS-PET substrate with tweezers (**Figure R24b**). Owing to the geometrical shape, the ZnO nano/microwire would lay down on the flat surface of the substrate, while the growth direction of the ZnO nano/microwire (the piezoelectric polarization *c*-axis) is naturally parallel to the flat substrate horizontally.

Then, the PDMS-PET substrate together with the ZnO nano/microwire would be placed on the microscope stage (with calibration ruler under the substrate) as shown in **Figure R25a**. By rotating the PDMS-PET substrate with the ZnO nano/microwire, we can align the nano/microwire very accurately with the calibration ruler under the microscope (**Figure R25b**). Furtherly cut the substrate into the desired shape with dimension of 30 mm × 10 mm based on the calibration ruler (**Figure R25c**). In this way,

the alignment of ZnO nano/microwire length's direction with the PDMS substrate's length direction is achieved.

To realize Schottky contact, the source/drain Ag electrodes are deposited on both ends of the nano/microwire by RF-magnetron sputtering, which is furtherly fixed by silver paste, to improve the interface property. Furthermore, the additional 0.5 mm of PMDS layer was also deposited on the ZnO nano/microwire as a top dielectric layer after curing at 100 °C for 1 hour (**Figure R24c**). Finally, the as-prepared PDMS microprotrusion could be mechanically released from the mold, and then carefully transferred and bonded onto the top of the device under the microscope, considering the transparent encapsulation of the whole device (**Figure R24d**).

We thank the reviewer for this suggestion again. And the above fabrication process has been added into the revised Supplementary Information and marked in red.

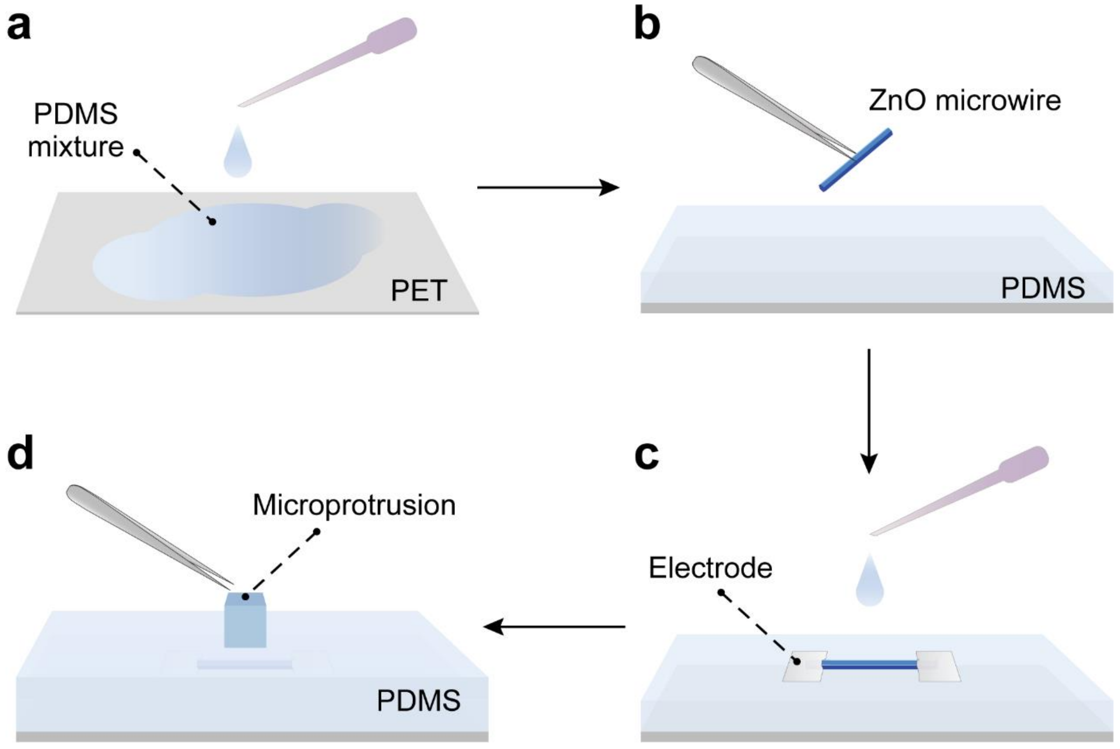

Figure R24. Schematic diagram showing the fabrication process of the DPT.

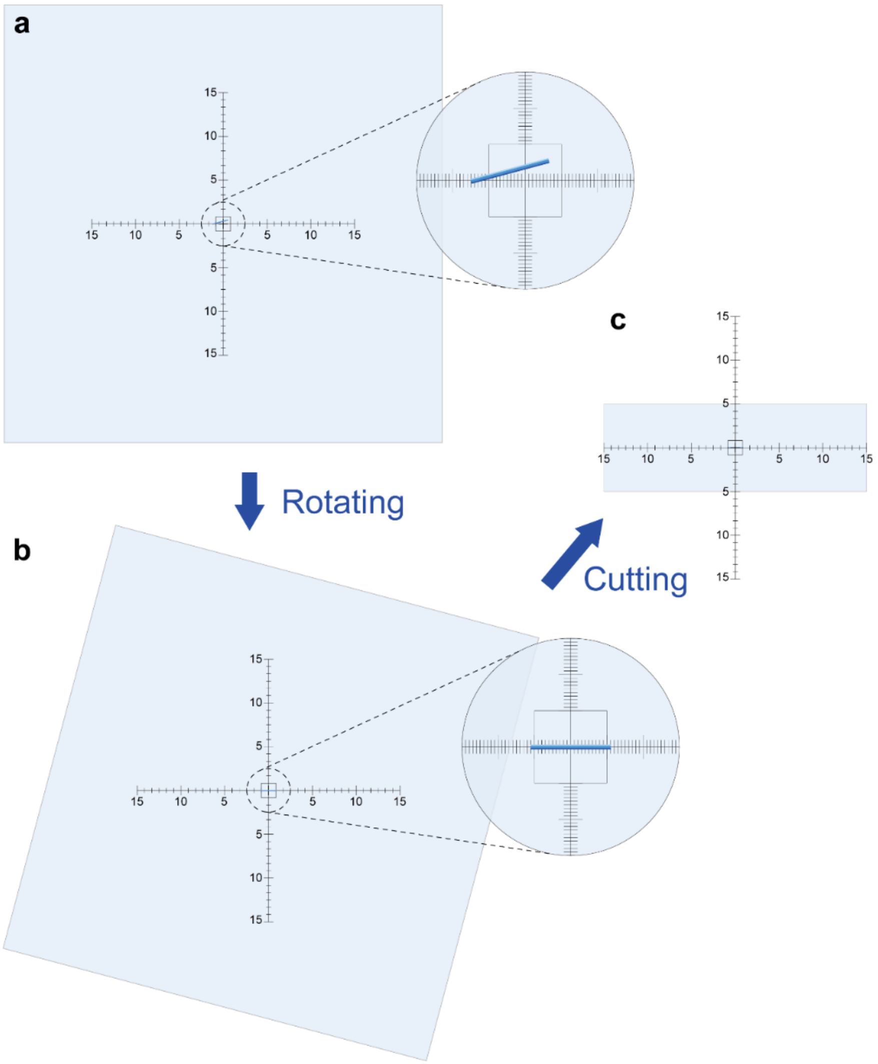

Figure R25. Schematic illustration of the ZnO nano/microwire alignment. a, The PDMS-PET substrate is placed on the calibration ruler under microscope. **b**, Aligning the nano/microwire to the calibration ruler by rotating the PDMS-PET substrate. **c**, Cutting the substrate with aligned ZnO nano/microwire into the desired shape based on the calibration ruler. The insets show the enlarged images of the central region indicated by dashed lines.

Question #7

About Fig. 3. There is an interesting phenomenon: as indicated in Fig. 3e, when responded to small forces, the sensor's force sensitivity value (221.5 N^{-1}) and sensing range (0 to 0.6 N) at lower bias (+1 V) is much larger than that (89.7 N^{-1} and 0 to 0.3 N) at higher bias (+3 V). Whereas, when responded to large forces, the sensor's force

sensitivity value (31.7 N^{-1}) and sensing range (0.6 to 1.1 N) at lower bias (+1 V) is much smaller than that (43.6 N^{-1} and 0.3 to 1.1 N) at higher bias (+3 V). Could the authors discuss this interesting phenomenon?

Response:

We thank the reviewer very much for this comment and constructive suggestion. It is really an interesting phenomenon, which also attracted our attention. Since there involve three regulations induced by piezoelectric polarization and bias voltage, the whole regulation process is very complicated. The three regulations can be labeled as: (1) opposite regulation induced by asymmetric polarization; (2) image force induced by high bias; (3) redistribution of voltage drops induced by high bias. In order to clarify the whole regulation process clearly and thus explain this interesting phenomenon, we need to analyze above three regulations in detail and show what kind of changes in the energy band of the device will be caused by the introduction of piezoelectric polarization and electric field.

(1) Opposite regulation induced by asymmetric polarization

As shown in **Figure R26**, we illustrate the energy bands of device under different bias and different mechanical stimuli. **Figure R26a-c** respectively shows the energy bands without force (**Figure R26a**), under small force (**Figure R26b**) and large force (**Figure R26c**). As can be seen, there are two Schottky barriers (short for SB) in series, through which electrical carriers (mainly electrons in this work) must pass. Here, we define the left one as SB1 and the right one as SB2. As shown in **Figure R26a**, since the SB2 is larger than SB1, the electrical transport characteristics of device are mainly controlled by SB2. In other words, SB2 plays a dominant regulatory role. As the applied force gradually increases (**Figure R26b**), a negative piezoelectric polarization charge is generated at SB1, resulting in an increased Schottky barrier height (SBH1); whereas a positive piezoelectric polarization charge is generated at SB2, resulting in a decreased Schottky barrier height (SBH2). As the force becomes large enough (**Figure R26c**), the asymmetric polarization and opposite regulation (SBH1 gradually increase, SBH2 gradually decrease) make SB1 and SB2 gradually become similar, and SB2 gradually loses its dominant regulatory role. At this time, SB1 and SB2 both affect the electrical

transport, rather than SB1 alone dominates in the case of applying a small force.

We also illustrate the corresponding energy bands of device under low bias (**Figure R26d-f**) and high bias (**Figure R26g-i**). Since SB1 and SB2 are in series, voltage drops of the two barriers ($V_{drop,1}$ for SB1 and $V_{drop,2}$ for SB2) add up to the bias voltage V_{bias}, which can be expressed as following formula (21).

$$V_{bias} = V_{drop,1} + V_{drop,2} \tag{21}$$

Similar to **Figure R26a-c**, the opposite regulation will always make SB2 lower with $V_{drop,2}$ smaller (**Figure R26d-f** under low bias) and SB1 higher with $V_{drop,2}$ larger **(Figure R26g-i** under high bias) as increasing force. This is the reason why the piezotronic modulation gradually decreases with the increase of force under a same bias (as shown in Figure 3e in the manuscript).

It should be noted that we only analyzed the piezotronic effect affected by the piezoelectric polarization charge up to now, and did not involve the influences of high bias. Next, we will focus on the analysis of two effects induced by high bias on piezotronic effect as follows.

(2) Image force induced by high bias

On the basis of the aforementioned opposite regulation induced by asymmetric polarization, another effect must be considered as the bias voltage increases, that is, the Schottky effect, especially at SB2 which plays the dominant role when the force is relatively small.

Based on semiconductor theory, there will exist an image force at Schottky barrier in the presence of an electric field, which makes the Schottky barrier not that 'sharp' and lead the Schottky barrier height lower. This effect is known as the Schottky effect or Schottky-barrier lowering. As the voltage bias (electric field) across Schottky barrier increases, the image force will increase and the Schottky barrier height lowering $\Delta\varphi_{\text{image force}}$ will become obvious, which can be derived from the following formula.

$$\Delta\varphi_{image\ force} = \sqrt{\frac{qE}{4\pi\varepsilon_s}} \tag{22}$$

in which q is the carrier charge, E represents the electric field at the Schottky barrier, ε_s is the dielectric constant of semiconductor.

Returning to the situation in this work, as shown in **Figure R26e** and **Figure R26h**, under the action of a small force, due to the weak opposite regulation, SB2 maintains its dominant regulatory role no matter under low bias or high bias, and the voltage drop $V_{drop,2}$ on it is approximately equal to the bias voltage V_{bias}. As the bias increases (from **Figure R26e** to **Figure R26h**), a gradually increased electric field falls on SB2, making Schottky effect become obvious.

In order to clarify the influence of image force induced by high bias on the piezotronic regulation, we illustrate the piezotronic modification of the energy band (conductive band) without image force (**Figure R27a**) and with image force (**Figure R27b**). In addition to the previously mentioned image force will reduce the Schottky barrier height, the image force will also cause the barrier to shift away from the interface between the metal and the semiconductor by a distance of x_m (**Figure R27b**). This will cause the piezoelectric polarization charge to stagger from the top position of barrier and reduce the piezotronic effect. In other words, the existence of the image force induced by high bias weakens the regulation of the piezotronic effect on the energy band. This is the main reason why piezotronic regulation weakens with the increase of bias when the force is small. It should be noted that the piezotronic regulation weakening here not only comes from the image force, but also is related to the narrower depletion layer width and the stronger electrostatic shielding of the polarization charges by the free carriers under high bias.

For the case of large force, the image force will also exist. However, due to the obvious opposite regulation, the voltage drop of SB2 is not high (and the electric field is not high), and the influence of image force is limited. In this case, another effect induced by high bias needs to be considered.

(3) Redistribution of voltage drops induced by high bias

Upon a large force, opposite regulation previously discussed in (1) will make SB2 gradually lose its dominant regulatory role as illustrated in **Figure R26c** or **Figure R26f**. At this time, the SBH1 and SBH2 are appropriate, and their voltage drops ($V_{drop,1}$ and

$V_{drop,2}$) are also appropriate. As the bias become high (**Figure R26i**) from low (**Figure R26f**), there is an easily overlooked effect, that is, redistribution of voltage drops between two Schottky barriers induced by high bias, will become important.

Energy bands of two Schottky barriers with similar barrier height in series under different biases are illustrated in **Figure R28**. When the bias tends to 0 V (**Figure R28a**), the forward biased SB1 and the reverse biased SB2 are similar in barrier height, and it can be considered that their voltage drops ($V_{drop,1}$ for SB1 and $V_{drop,2}$ for SB2) are approximately equal to $V_{bias}/2$. In this case, both SB1 and SB2 determinate the electrical transport of device. As the bias gradually increase to a small value (**Figure R28b**), SB1 will decrease, and its voltage drop $V_{drop,1}$ will decrease to a relatively small value $V_{bias}/2 - \Delta$, compared with that ($V_{bias}/2 + \Delta$) of SB2. Since the bias is very low, the difference 2Δ of voltage drops between the two is small, and the voltage drops of the two is approximately equal to $V_{bias}/2$. In this case, SB1 and SB2 still determinate the electrical transport of device together. Therefore, **when the bias is low, both SB1 and SB2 control the device transport.** Since the opposite regulation exists between SB1 and SB2, the overall performance of piezotronic effect (sensitivity value) is relatively weak.

As the bias furtherly increases to a high value, SBH1 will furtherly decrease (finally tend to 0), and 2Δ is a considerable large value. At this time, the voltage drop of SB2 is $V_{drop,2}$ far larger than $V_{bias}/2$ and tends to V_{bias}. Usually, the value of $V_{drop,2}/(V_{drop,1} + V_{drop,2})$ is more than 75%, and gradually increase with applied bias. In other words, **when bias is high, the reverse biased SB2 will play a dominant regulatory role** in controlling the device transport. So, the piezotronic modification of SB2 will totally determine the device, resulting a high overall performance of piezotronic effect (force sensitivity value).

It should be noted that the image force also exists when large force is applied. The influence of image force induced by high bias and the influence of redistribution of voltage drops induced by high bias offset each other, resulting in only a slightly enhanced force sensitivity value at bias of 3 V compared with that at 1 V. Maybe in some case, it is entirely possible that the force sensitivity value at 3V is smaller than or

equal to that at 1 V.

In summary, through the above qualitative analysis, the opposite regulation induced by asymmetric polarization, the image force induced by high bias and the redistribution of voltage drops induced by high bias all affect the overall piezotronic regulation process, resulting in the interesting phenomenon. With the increase of bias, what happens to the interface engineering by polarizations including piezoelectric polarization, flexoelectric polarization and ferroelectric polarization? This question is an important and cutting-edge research topic in the field of piezotronics and flexotronics (maybe also in ferroelectric electronics), which has rarely been discussed before. More in-depth investigations are needed in the further.

Thanks again for this very constructive suggestion. We have added the above discussion into the revised Supplementary Information (marked in red) for the reference of experts in related fields.

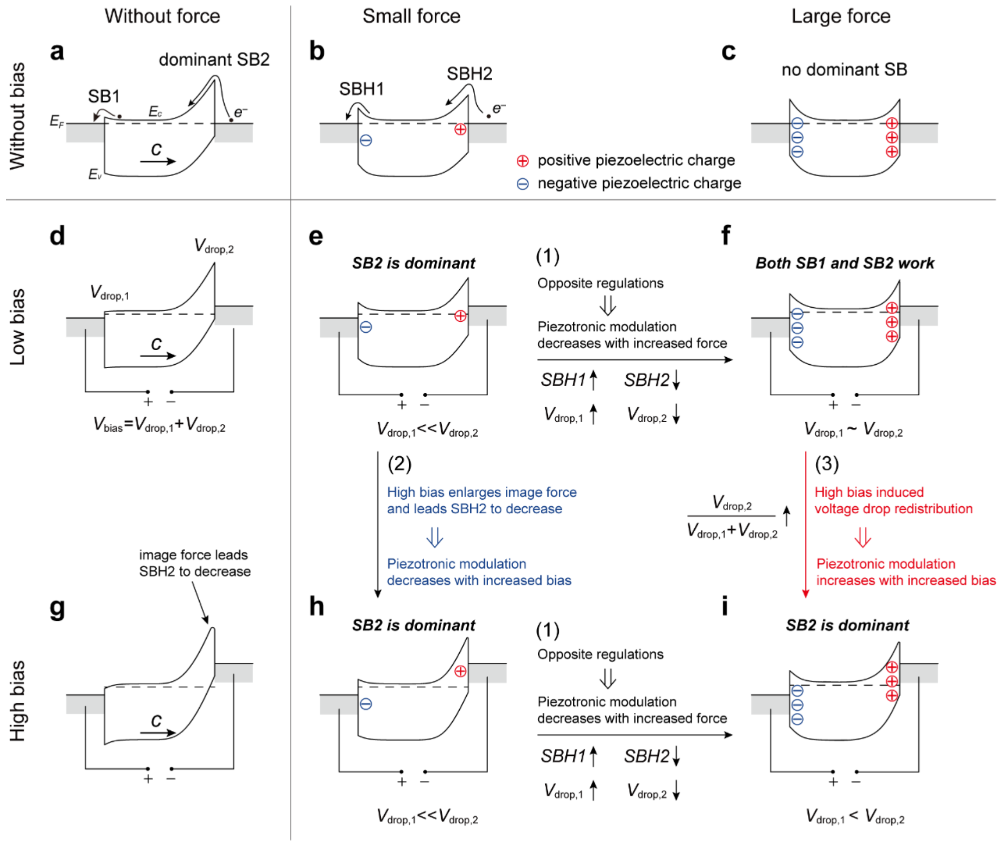

Figure R26. Energy bands of device under different bias and different force. a-c, The energy bands under no bias without force (**a**), with a small force (**b**) and a large

force (**c**). **d-f,** The energy bands under low bias without force (**d**), with a small force (**e**) and a large force (**f**). **g-i,** The energy bands under high bias without force (**g**), with a small force (**h**) and a large force (**i**). The left Schottky barrier, Schottky barrier height and corresponding voltage drop are respectively short for SB1, SBH1 and $V_{drop,1}$. The right Schottky barrier, Schottky barrier height and corresponding voltage drop are respectively short for SB2, SBH2 and $V_{drop,2}$. The bias voltage is V_{bias}.

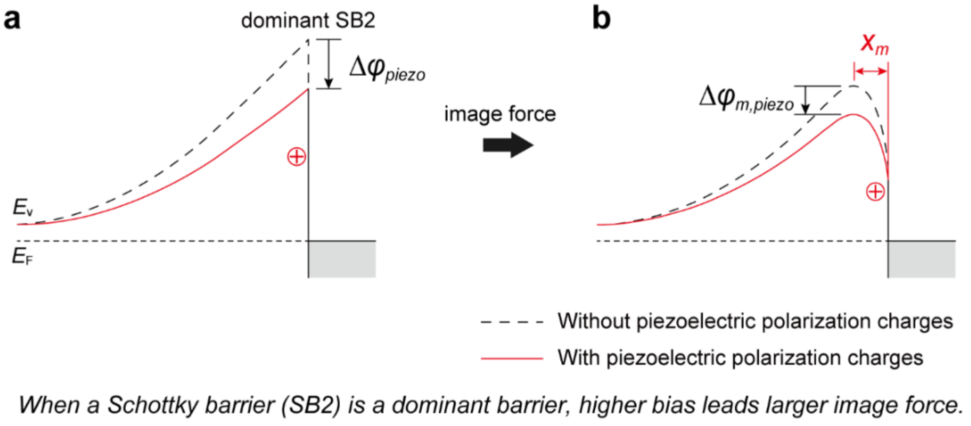

Figure R27. Influence of the image force induced by high bias on piezotronic modification of energy bands. a, Piezotronic modification of conductive band without image force. The $\Delta\varphi_{piezo}$ represents the change of Schottky barrier height produced by piezotronic effect. **b**, Piezotronic modification of conductive band with image force. The $\Delta\varphi_{m,piezo}$ represents the change of Schottky barrier height in the existence of image force produced by piezotronic effect. The x_m is the displacement between the top of barrier and the interface induced by image force.

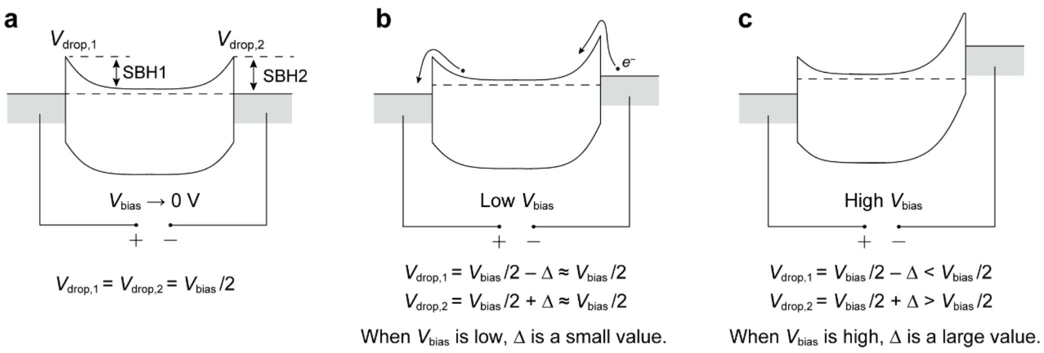

Figure R28. Change of energy bands and Redistribution of voltage drops induced

by high bias. a-c, Diagrams of energy bands under a bias tending to zero (**a**), under a low bias (**b**) and under a high bias (**c**). The Schottky barrier heights (corresponding voltage drops) in the left side and in the right side are respectively short for SBH1 ($V_{drop,1}$) and SBH2 ($V_{drop,2}$). The 2Δ represents the difference between $V_{drop,1}$ and $V_{drop,2}$. The bias voltage is V_{bias}.

Question #8

About Fig. 4. As indicated in Fig. 4d, 4e and 4h, why are different methods adopted to calculate the gauge factors at forward and reverse biases? What are the resistance change ratio under reverse bias and the current change ratio under forward bias?

Response:

We thank the reviewer very much for this important comment. Next, we will firstly discuss the reason of adopting different methods to calculate the gauge factors in piezotronics, and then calculate the resistance change ratio under reverse bias and the current change ratio under forward bias to help demonstrate the reason.

(1) Why are different methods adopted to calculate the gauge factors at forward and reverse biases?

Response: We thank for the reviewer's nice comment. Calculating gauge factors by adopting the resistance ratio under one bias while the current ratio under the other bias is a unique issue in the field of piezotronics, which is attributed to the strain-induced asymmetric modulation of electrical transport by piezoelectric charges (piezotronic effect). This is completely different from the traditional piezoresistive effect. In order to clearly reveal the reason, we will discuss in three parts: ① definition of gauge factor in devices based on piezoresistive effect; ② difference between piezoresistive effect and piezotronic effect; ③ definition of gauge factor in asymmetric strain-controlled devices. It is hoped that this discussion can clearly reveal the reason.

① *Definition of gauge factor in devices based on piezoresistive effect*

When a device is subjected to external stimuli, its electrical transport changes with the applied strain. This phenomenon is called the resistance-strain deformation effect of the sensor, which is characterized by a sensitivity coefficient named gauge factor.

Gauge factor is originally used to indicate the sensitivity of the device performance in response to mechanical stimuli based on piezoresistive effect, and is now also used to indicate the strain sensing performance of piezotronic devices.

As an important parameter for evaluating the device performance, gauge factor of the strain sensor based on piezoresistive effect can be generally defined as the ratio of relative change in resistance to the applied strain based on the electromechanical performance [7, 8], which can be calculated using the following equation:

$$gauge\ factor = \frac{\Delta R/R_0}{\Delta\varepsilon} = \frac{R_\varepsilon - R_0}{R_0 \cdot \Delta\varepsilon} \tag{23}$$

where R_0 is the initial resistance under no strain; R_ε is the resistance at the strain of ε; $\Delta\varepsilon$ denotes the change of the strain applied to the sensor.

Piezoresistive effect is a change in the electrical resistance of a semiconductor or metal when mechanical strain is applied. It can be observed in a metal or a semiconductor, while the piezoresistive effect in semiconductor materials is generally much stronger than in metals. The resistance (R) of a material is defined as: $R = \rho \cdot l/a$, where l is the length, a is the cross-sectional area of the material and ρ is electrical resistivity. In the materials with piezoresistive effect, the gauge factor can be also described as follows:

$$gauge\ factor = \frac{\Delta R/R_0}{\Delta\varepsilon} = 1 + 2v + \frac{\Delta\rho/\rho_0}{\Delta\varepsilon} \tag{24}$$

in which v is the Poisson's ratio, ρ_0 is the original electrical resistivity, and $\Delta\rho$ is the change in electrical resistivity. The first term $1 + 2v$ represents the devotion provided by the strain-induced geometric structure change, and the second term $(\Delta\rho/\rho_0)/\Delta\varepsilon$ represents the devotion provided by the strain-induced resistivity change. Generally, in piezoresistive metals, the first term determines the gauge factor; while in piezoresistive semiconductors, the second term is much larger than the first term. For example, the gauge factor of thin-film metal is about 2-5, and the gauge factor of single crystal silicon can reach 200 (a very high value in piezoresistive strain sensors).

Back to the formula (23), upon a same strain, the device with higher gauge factor

means higher resistance change ratio $\Delta R/R_0$ and better device performance. This is always right in the traditional piezoresistive effect. However, with the emerging of strain-controlled devices with new principles (*e.g.* piezotronic effect), the definition of gauge factor has also been updated. For example, for some new devices with increased current ($I_\varepsilon > I_0$) with the applied strain, the resistance change ratio can be expressed as

$$\frac{R_\varepsilon - R_0}{R_0} = \frac{\frac{V}{I_\varepsilon} - \frac{V}{I_0}}{\frac{V}{I_0}} = \frac{I_0 - I_\varepsilon}{I_\varepsilon} = \frac{I_0}{I_\varepsilon} - 1 \tag{25}$$

As a result, the better the performance of the device, the smaller I_0/I_ε (tends to 0), and more strangely, the closer the resistance change ratio is to -1! So, the definition of gauge factor (formula (23)) is not suitable for the new principle devices in some cases.

② *Difference between piezoresistive effect and piezotronic effect*

Piezotronic effect, utilizing strain-induced piezoelectric charges/potentials at interfaces to tune the electrical transport of interfaces, is a new effect firstly proposed by Zhong Lin Wang in 2007 [9]. Piezotronic effect usually exhibits the strain-induced asymmetric modulation of electrical transport, which is different from the strain-induced symmetric modulation of piezoresistive effect.

In piezoresistive effect, as shown in **Figure R29a**, if a tensile strain increases the resistance of the device, no matter the device is forward biased or reverse biased, the forward current and reverse current of the device will both decrease with the tensile strain. As also shown in **Figure R29b**, if a compressive strain decreases the resistance of the device, no matter the device is forward biased or reverse biased, the forward current and reverse current of the device will both increase with the compressive strain. As a result, the electrical transport characteristics of the device is illustrated in **Figure R29c**, indicating that the strain-induced modulation of electrical transport by piezoresistive effect is a symmetric effect. Apart from above case, if a tensile strain decreases the resistance and a compressive strain increases the resistance, the same conclusion will also be obtained, indicating a symmetric strain-induced modulation of

electrical transport by piezoresistive effect. So, it is a strain-induced **symmetric modulation** of electrical transport by piezoresistive effect.

Piezotronic effect is an interface effect that asymmetrically modulates local contacts at different terminals of the device [4], as schematically illustrated in the following **Figure R30**. Schottky junctions will be formed in local contacts at different terminals of the device without strain (**Figure R30a**). Due to the accidental error/uncertainty of device manufacturing [6], the Schottky junctions at both ends will be slightly different.

When subjecting to a tensile strain (**Figure R30b**), positive piezoelectric polarization charges are produced at the surface of the ZnO nano/microwire (assuming its piezoelectric *c*-axis points to the left) on the left contact of device, which will reduce the barrier height of the Schottky junction; while negative polarization charges are produced on the right contact, leading to an increasement of the barrier height of the Schottky junction. When a forward bias (potential of right electrode is higher than that of left electrode, **left** in **Figure R30b**) is applied, the electrical transport characteristics are mainly determined by the Schottky junction on the left terminal, which is reversely biased. Because of the decreased barrier height of the left contact, the current flowing through the device will increase and become larger than that of device without strain. Similarly, when the device is reversely biased (potential of right electrode is lower than that of left electrode, **right** in **Figure R30b**), under a same bias voltage, the (reverse) current of the device will be smaller than that without strain. Therefore, compared to the case without strain, the forward current of the device with tensile strain will increase; while the reverse current will decrease. With the same principle, we can also analyze the modulation of the current of the device under a compressive strain, as shown in **Figure R30c**. We can see that the compressive strain will cause a decrease of the forward current (**left** in **Figure R30c**) and an increase of the reverse current (**right** in **Figure R30c**).

The corresponding *I-V* curves can be schematically plotted in the **Figure R30d**. The strain-induced modulation of electrical transport by piezoelectric charges (piezotronic effect) is asymmetric. The piezotronic effect modulation arises as a result

of the polarization of nonmobile ions in the piezoelectric crystal [2, 3]. Thus, it is a strain-induced **asymmetric modulation** of electrical transport by piezotronic effect.

③ *Definition of gauge factor in asymmetric strain-controlled devices*

For piezotronic devices, the strain-induced modulation of electrical transport by piezotronic effect is an asymmetric effect. As shown in **Figure R30d**, under the action of a tensile strain ($\Delta\varepsilon > 0$), the resistance of the device will gradually decrease under forward bias, and will gradually increases under reverse bias. In order to ensure that both the gauge factor values calculated under forward and reverse bias are positive and satisfy the rule that the higher gauge factor the better the performance, the current change ratio to the strain is utilized as gauge factor for the case when the current increases with strain:

$$gauge\ factor = \frac{\Delta I/I_0}{\Delta\varepsilon} \tag{26}$$

in which I_0 is the original current and ΔI is the change in current.

And, the resistance change ratio to the strain is utilized as gauge factor for the case when the current decreases with strain:

$$gauge\ factor = \frac{\Delta R/R_0}{\Delta\varepsilon} \tag{27}$$

Only by defining gauge factor in this way (formula (26) and formula (27)) can gauge factor characterize the strain sensing performance of piezotronic devices similar to those based on the traditional piezoresistive effect.

(2) What are the resistance change ratio under reverse bias and the current change ratio under forward bias?

Response: We thank the reviewer for this valuable suggestion. As a demonstration shown in **Figure R31a**, the forward current decreases with the compressive strain, while the reverse current increases with the compressive strain. In this situation, we can plot the current change ratio under reverse bias as shown in **Figure R31b**, and plot the resistance change ratio under forward bias as shown in **Figure R31c**. As a contrast, if conversely, we will get the resistance change ratio under reverse bias in **Figure R31d**,

and get the current change ratio under forward bias in **Figure R31e**. It can be seen from **Figure R31d-e** that the better the device performance, the closer the change ratios are to -1, which cannot properly characterize the performance of the devices.

In summary, due to the strain-induced asymmetric modulation of electrical transport by piezotronic effect, in order to satisfy the rule that the higher gauge factor the better the performance, we use resistance change ratio when analyzing DPT forward bias characteristics, and use current change ratio when analyzing reverse bias characteristics.

To improve the readability and quality of the manuscript, we have added the above discussions to Supplementary Information and marked them in red.

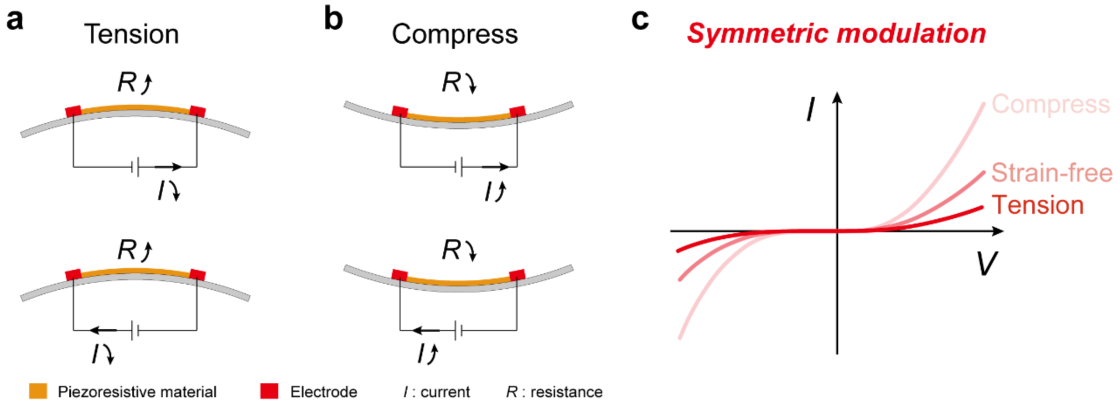

Figure R29. Strain-induced symmetric modulation of electrical transport by piezoresistive effect. a, Tensile strain increases the resistances and decreases the currents both under forward bias and reverse bias. **b**, Compressive strain decreases the resistances and increases the currents both under forward bias and reverse bias. **c**, Schematic illustrations of the *I*-*V* curves of the device under no strain, tensile strain and compressive strain, which indicate a symmetric modulation of electrical transport by strain in piezoresistive effect.

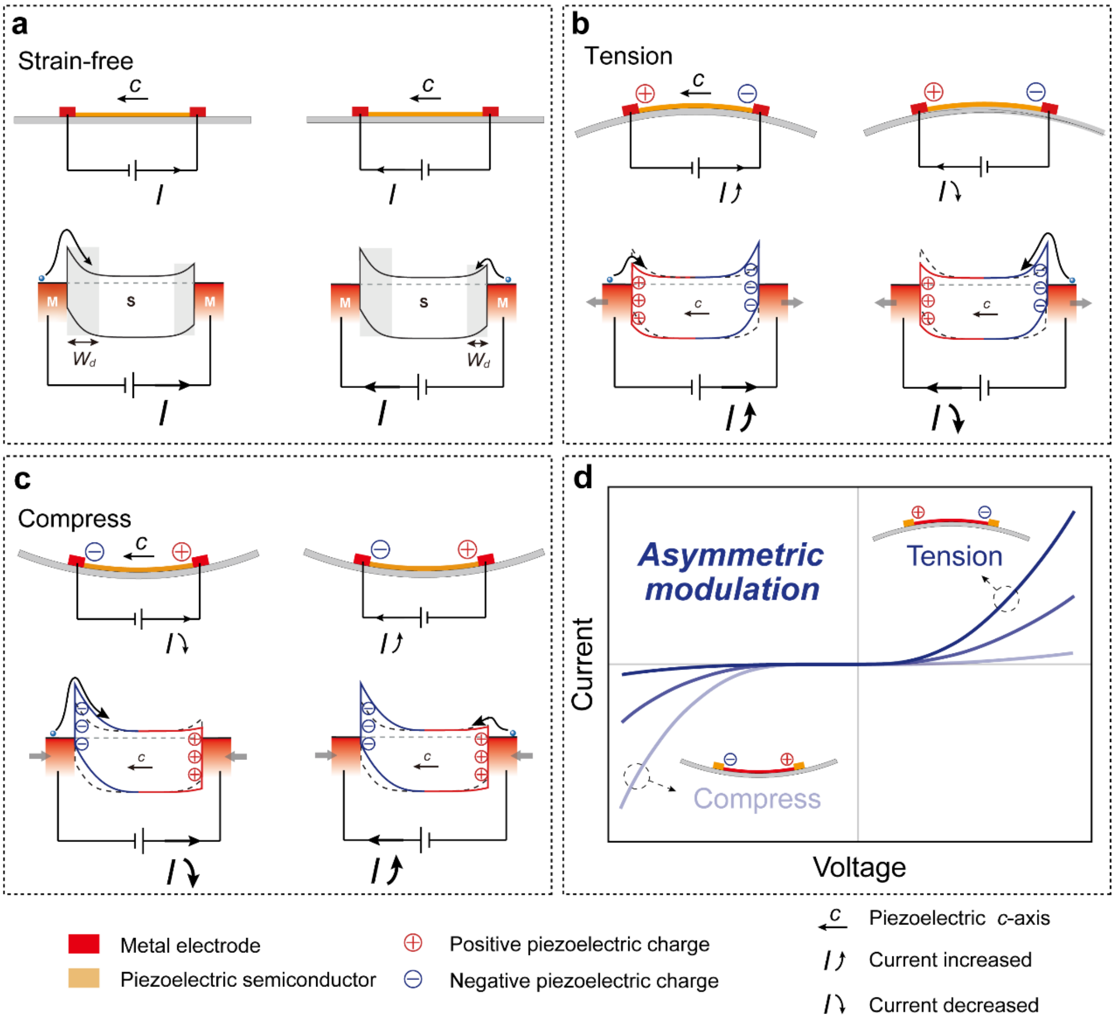

Figure R30. Strain-induced asymmetric modulation of electrical transport by piezoelectric charges (piezotronic effect). a-c, Schematic diagrams of bending situations and corresponding energy bands of device with no strain (**a**), tensile strain (**b**) and compressive strain (**c**). **d**, Schematic illustrations of the *I-V* curves of the device under no strain, tensile strain and compressive strain, which indicate an asymmetric modulation of electrical transport by strain in piezotronic effect.

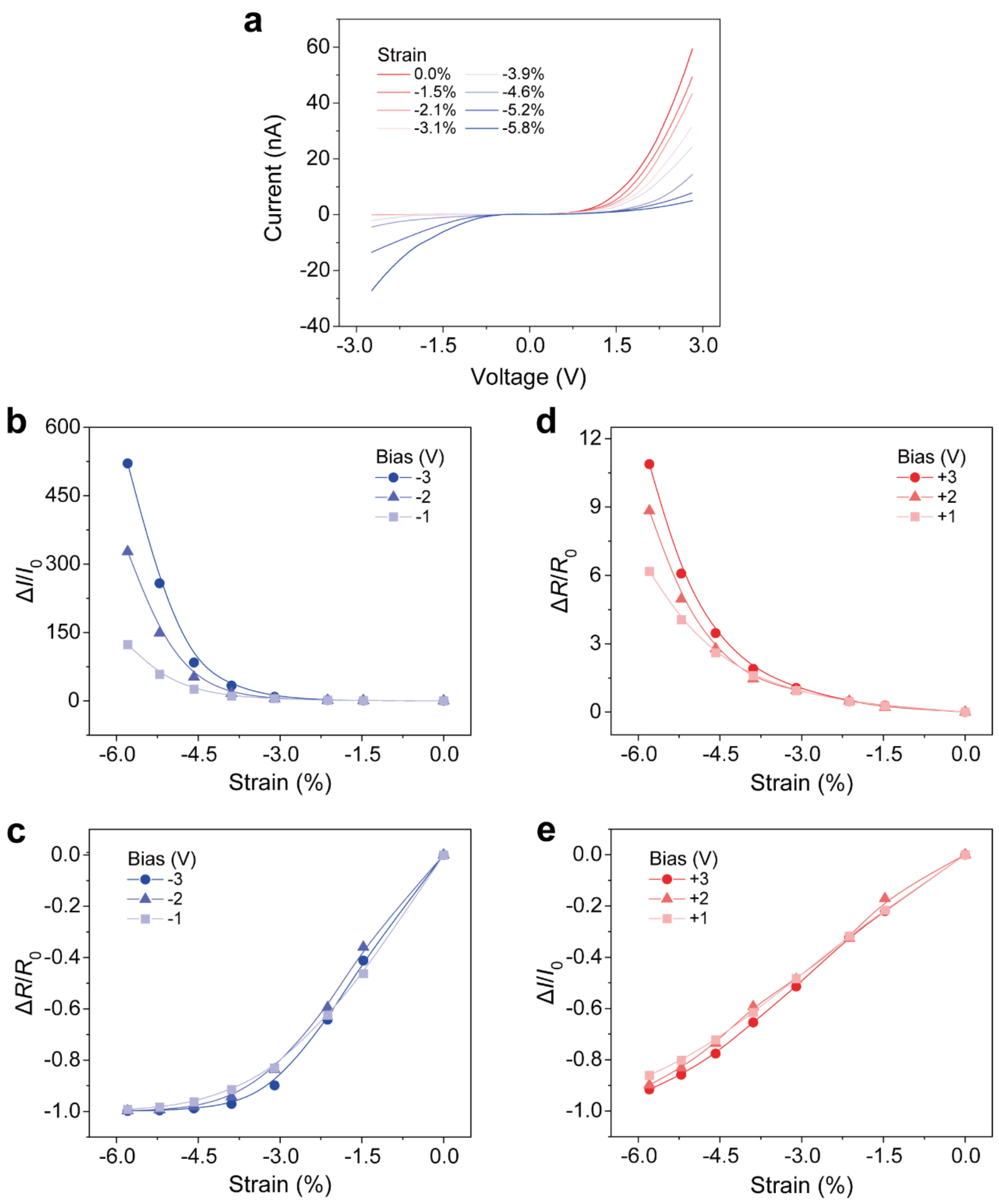

Figure R31. Resistance change ratio and current change ratio used to characterize the piezotronic device under forward bias and reverse bias. a, Strain-induced asymmetric modulation of electrical transport by piezotronic effect. **b**, **c**, Current change ratio (**b**) and resistance change ratio (**c**) as functions of the strain under reverse bias. **d**, **e**, Resistance change ratio (**d**) and current change ratio (**e**) as functions of the strain under forward bias.

Question #9

Line 111 and 122, the contents are written as “Fig. 1b-I and II” and “Fig. 1b-III”. The information is wrong. Please double check the contents with Fig. 1.

Response:

We are grateful to the reviewer for kindly reminding us of this error in Figure 1. We have carefully checked the full manuscript and supplementary information, and revised all similar errors. Now, all the labels like “(i)”, “(ii)”, “(iii)” in figures have been replaced by new labels like “(I)”, “(II)”, “(III)”. For example, Figure 1 in the revised manuscript is changed as the following **Figure R32**.

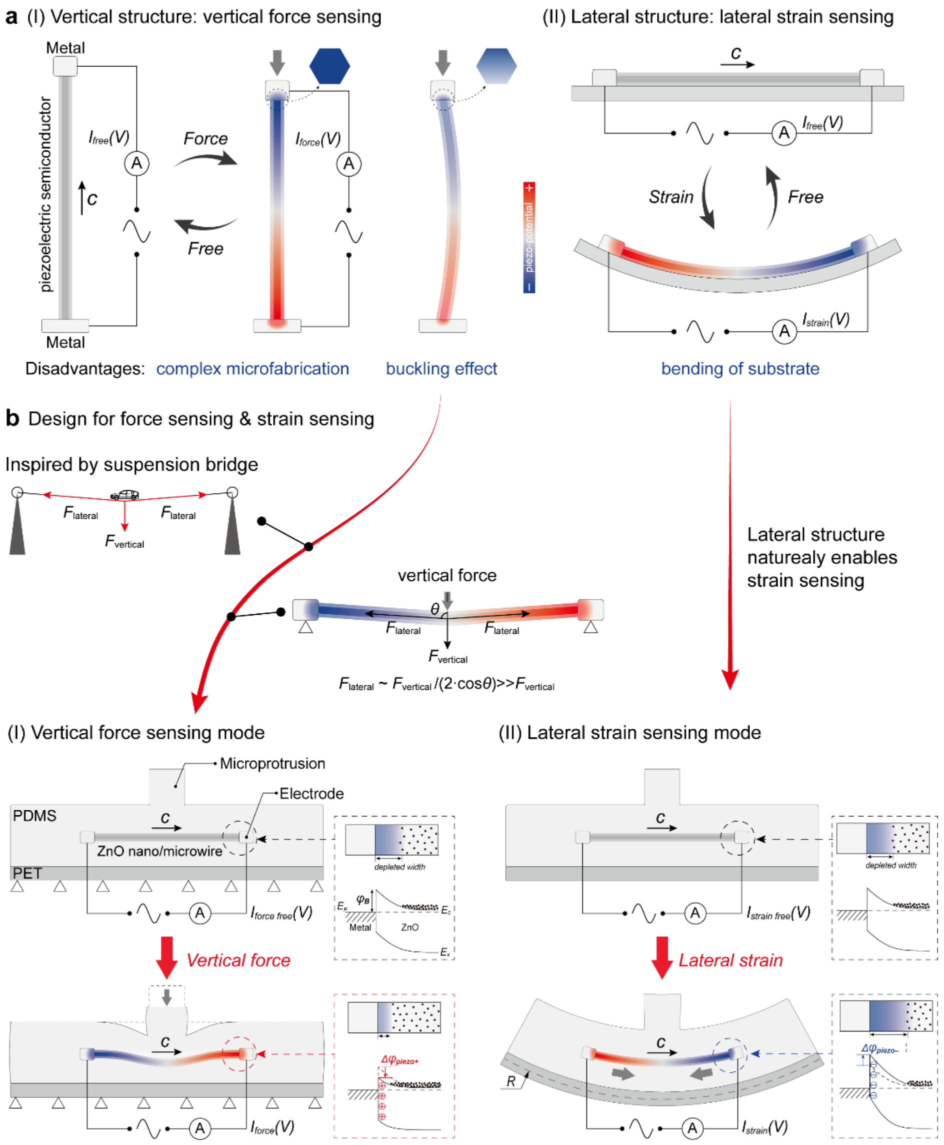

Figure R32. The new Figure 1 in the revised manuscript.

Question #10

The authors have reported several force/strain sensors based on piezotronic transistors previously. It is suggested that more detailed comparison between this work and their previous works should be added. For example, why are the performances improved? In the current manuscript, mainly only the performance is compared. I understand that the innovation of this work focuses on the realization of dual-modal sensing for vertical force and lateral strain. But I think if the mechanism of performance improvement is analyzed and discussed, then this work would have much more significant impact in the research field.

Response:

We appreciate the reviewer very much for this valuable comment and nice suggestion, which promotes us to better condense the key points of this work and reminds us where our work can be further improved. As for the dual-modal piezotronic transistor (DPT) proposed in this work, its vertical force sensing mode exhibits a pressure sensitivity of 1.759 eV/MPa, which is an ultra-high value in piezotronic transistors and is a big step in piezotronics; while its lateral strain sensing mode possesses a high gauge factor, which is at the upper middle level in piezotronics. In fact, the performance improvement of DPT is mainly reflected on its the vertical force sensing mode. Next, based on the vertical force sensing mode, we will detailly analyze the comparison between DPT and previous works, and introduce our original design idea of DPT to reveal the reasons for the high performance of DPT in detection of vertical force first. Then, we will briefly discuss the comparison between the lateral strain sensing mode of DPT and previous works, and give the reason for its relatively good performance. It is hoped that by showing our original design idea of this work, DPT can have more significant impact and inspiration.

(1) Performance improvement of DPT on vertical force sensing mode

① *Structural evolution of previous piezotronic transistors for vertical force sensing*

At the earliest, the design of utilizing piezotronic transistor to sense vertical force is shown in **Figure R33a**. A vertical aligned piezoelectric semiconductor nano/microwire is the core unit to detect the vertical force. As a vertical force is applied

on the vertical nanowire, the nano/microwire will deform along its length and generate piezoelectric charges and corresponding piezoelectric potentials at the Schottky barrier formed between the nano/microwire and metal electrodes. The vertical force induced piezoelectric potentials regulate the Schottky barrier height to control the carrier transport of nano/microwire, thereby realizing the detection of the vertical force.

However, when the vertical nano/microwire is subjected to a vertical force, a buckling effect will occur as shown in **Figure R33b-c**. The buckling effect will cause two negative influences on the piezotronic effect. On the one hand, due to the compression caused buckling of the nano/microwire, the deformation of the nano/microwire along its length direction caused by the vertical force becomes smaller, and the piezoelectric charge generated at both ends of the nano/microwire becomes less. In fact, part of the vertical force is wasted on bending the nano/microwire. On the other hand, as shown in upper side of **Figure R33c**, the buckling of the nano/microwire will lead to one side of the nano/microwire being stretched and the other side being compressed, which makes the potential distribution at the end of the nano/microwire very uneven as shown in the bottom side of **Figure R33c**. This uneven piezoelectric potential distribution has negative influence on piezotronic effect, because the electrical transport of the interface (Schottky barrier) is mainly determined by the region with the lowest barrier on the interface. Therefore, as the core part of piezotronic transistor, the vertical nano/microwire has a buckling effect under the action of vertical force, which greatly weakens the performance of piezotronic effect. Piezotronic transistors based on vertical nano/microwires, which are compressed by vertical force, usually possess low pressure sensitivities (CdSe nano/microwire [10]: 0.677 meV/MPa; ZnO nano/microwire [11]: 0.083-0.422 meV/MPa; GaN nano/microwire [12]: 0.212-0.240 meV/MPa).

In order to solve the problem of buckling effect in the vertical nano/microwire, the firstly considered solution is to replace the single vertical nano/microwire with a nano/microwire cluster as shown in **Figure R33d**, and use PDMS to enhance the stability of the mechanical structure. In this case, the nano/microwires in the cluster are not easy to buckle under the vertical force. Based on this design, the pressure sensitivity of the piezotronic transistor (ZnO nano/microwire cluster [4]) can be up to 33.39

meV/MPa.

Another solution is to replace the vertical nano/microwire or nano/microwire cluster with a nanoplatelet as shown in **Figure R33e**. As the aspect ratio (thickness-to-diameter) of the nanoplatelet is very small, approximately 0.42, the nanoplatelet will not buckle like nano/microwire upon a vertical force. And, the deformation of the nanoplatelet caused by the vertical force is strictly along the direction of the thickness or the polarization axis (*c*-axis in ZnO) of the nanoplatelet, which perfectly avoids the problem of buckling effect. The corresponding piezotronic transistor based on this design increases the pressure sensitivity to 60.97~78.23 meV/MPa [13].

Furtherly, based on the unique geometric characteristics of the nanoplatelet, that is, the end surface (up or down surface) is large, we changed the upper and lower electrode structure to a double lower electrode structure. In this case, both positive piezoelectric charges/potentials will be generated at the two Schottky barriers formed between the nanoplatelet and bottom electrodes when subjected to vertical force. Since the piezoelectric charges/potentials of the same polarity (positive in **Figure R33f**) synergistically regulate (both decrease) the two Schottky barrier heights, the opposite regulation discussed previously (in the section (1) of the **response to Question #7**) can be avoided, which is beneficial to improving the performance of piezotronic transistor. This design furtherly improves the pressure sensitivity to 84.2~104.4 meV/MPa [14].

Additionally, we have two other works that only explore the two-dimensional scale and the macroscopic bulk scale of the nanoplatelets, and do not involve the updating of the vertical structure and performance improvement. As shown in **Figure R33g**, in order to explore the performance of piezotronic transistor with thinner piezoelectric semiconductor, we have also grown a kind of ZnO nanosheet with a thickness of about 2 nm (Note: we call it nanosheet to distinguish it from nanoplatelet), and fabricated 2D (two-dimensional) piezotronic transistor [15, 16]. Due to the influence of encapsulation, the vertical force is difficult to be effectively applied to the nanosheet, and the pressure sensitivity is smaller than that of the nanoplatelet-based piezotronic transistor in **Figure R33f**. Also, since the nanoplatelet is not easy to operate, we used it to study the regulation of piezoelectric polarization charge on the bulk with a large

number of interfaces (**Figure R33h**), which is called statistical piezotronic effect [17]. Of course, as expected, because of some nanoplatelets with *c*-axis up and the other with *c*-axis down, the pressure sensitivity is also lower than that of piezotronic transistor based on a single nanoplatelet in **Figure R33f**.

Through the above introduction about structural evolution of piezotronic transistors for vertical force sensing, the problem of buckling effect mainly results from the compression bending of the vertical nano/microwire upon vertical force. Although the nanoplatelet can solve the problem of nano/microwire, it is not easy to operate the nanoplatelet due to its small size both in length (thickness) and diameter, and is not a feasible scheme for the time being. Then, a question arises in **Figure R33i**, that is, how to use the elongation of nano/microwires (which can avoid buckling effect caused by compression for nano/microwires) rather than the compression of nano/microwires (which would induce the problem of buckling effect that long-time limits the performance of piezotronic transistors for vertical force sensing) to achieve the detection of vertical force.

② *Our original design idea of DPT for vertical force sensing*

Through in-depth consideration of above question, and inspired by the suspension bridge (**Figure R34a**), we think of constraining the two ends of a lateral nanowire, and then using a vertical force to press the middle of the nano/microwire and thus elongate the nano/microwire as shown in **Figure R34b**. In this way, we can use the elongation of lateral nano/microwire to sense the vertical force, and perfectly solve the problem of the buckling effect in the vertical nano/microwire structure. This is the prototype design of DPT. It is worth noting that the downward bending here (**Figure R34b**) is totally different from previously discussed bending of vertical nano/microwire in the problem of the buckling effect (**Figure R33c**). The vertical force induced downward bending of the lateral nano/microwire here is to elongate the nano/microwire, thus to generate piezoelectric charges/potentials. While, the vertical compressive force caused bending deformation (buckling effect) of the vertical nano/microwire decreases the net compressive deformation along the nano/microwire, weakening the generated piezoelectric charges/potentials.

In addition to the above advantage of avoiding the buckling effect of vertical nano/microwires under compression, we can use a very simple model to estimate the lateral force $F_{lateral}$ produced along the nano/microwire as shown in **Figure R34b-I**. Assuming that the nano/microwire is similar to the rope, a small vertical force $F_{vertical}$ will produce a very large tensile force $F_{lateral}$ (approximately equaling to $F_{vertical}/(2 \cdot \cos\theta)$, in which θ is the angle approaching 90°). Since θ approaches 90°, $F_{lateral}$ possesses a very large value. Although this estimation model is very rough, it can be utilized to estimate the qualitative relationship between $F_{lateral}$ and $F_{vertical}$, and the fact that a small vertical force can cause the nano/microwire to tighten and induce a big lateral force to elongate the nano/microwire. In other words, another advantage of the suspension-bridge-inspired design (**Figure R34b-I**) is amplifying the response (elongation and corresponding piezoelectric charges/potentials) of nano/microwire to the vertical force.

Actually, in our earliest design, we found that the nano/microwire would produce a very large tensile deformation along its length direction when subjected to a very small vertical force, and was very easy to break into two sections (**Figure R34b-II**).

Next, in order to avoid very large local stresses particularly at the positions of the force point and electrodes, we designed a structure as shown in **Figure R34c**, and used PDMS to wrap the nano/microwire. The soft nature of PDMS allows the nanowire to sustain a large enough tensile stress, which can avoid the nano/microwire fracturing when subjected to very large local stresses. Through this design, we successfully achieve devices that are not easy to be damaged and possess the response of lateral nano/microwire to the vertical force. However, there is a defect in this structure, that is, when subjected to the vertical force, the introduction of PDMS will disperse the vertical force on the whole nano/microwire, causing the whole nano/microwire to move down instead of downward bending to cause elongation.

In order to further enhance the influence of the vertical force, a microprotrusion as shown in **Figure R34d** is added to make the vertical force mainly concentrate on the middle part of the nano/microwire, thus more effectively elongating the nano/microwire rather than pressing the whole nano/microwire down. Based on this

structure, DPT proposed in this work achieves the pressure sensitivity of 1.759 eV/MPa, which is 16.5 times more than that of previously reported piezotronic transistors (Supplementary Table 4 in the revised Supplementary Information).

③ *Features of DPT leading to performance improvement on vertical force sensing*

By comparing the differences and progressive relationships between this work and the previous works, we can see that the DPT possesses the following four features:

I. it has a basic principle of utilizing the response of lateral nano/microwire to vertical force to realize the vertical force sensing;

II. it utilizes elongation of nano/microwires (rather than compression of nano/microwires) to sense vertical force, which properly solves the problem of buckling effect in traditional vertical structure.

III. the structure of DPT inspired by suspension bridge can amplify the vertical force to the tensile force along the length of the nano/microwire;

IV. the soft nature of PDMS avoids the nano/microwire to bear local extensive stress, and the microprotrusion makes the vertical force concentrate on the middle of the nano/microwire and thus more effectively elongate the nano/microwire under the action of vertical force.

All these features together make DPT not easy to be damaged and exhibit high pressure sensitivity in response to the vertical force.

(2) Performance improvement of DPT on lateral strain sensing mode

① *Impact of the DPT design on the performance of vertical force sensing*

From the above discussion (the section (1)), it can be seen that the design idea of DPT is mainly to meet the needs of vertical force sensing. Actually, as previously discussed in the **response to Question #3**, the advantage of DPT in lateral strain sensing mode lies on the amplified strain ε along the nano/microwire that can be estimated by the following formula

$$\varepsilon = -\frac{h_{PDMS,bottom} + h_{PET}/2}{R} \tag{28}$$

in which R is the curvature radius of the bending PET substrate, $h_{PDMS,bottom}$ is the

thickness of the bottom PDMS layer, and h_{PET} the thickness of the PET.

We can find that the introduction of the bottom PDMS layer will increase the strain produced under the same bending amount of PET. However, the performance improvement caused by this structure design is not large. As a result, its performance is only upper-middle level in piezotronic transistors. It is worth noting that, compared with the traditional lateral structure (**Figure R18a**), this DPT structure design (introduction of the bottom PDMS layer, **Figure R18b**) can induce greater strain on the nano/microwire without damaging it, which is in fact the biggest advantage of the design of DPT on lateral strain sensing.

Therefore, through the above discussion of the research idea and the structural evolution in piezotronic transistor, the mechanism of performance improvement is analyzed in detail, which can provide an in-depth understanding of the dual-modal piezotronic transistor. We thank the reviewer very much for this very nice suggestion, which also enables us to study, understand and present this work from another perspective. To improve the quality of this manuscript, we have added above discussion in the supplementary information and marked them in red.

② *Previous structure of piezotronic transistors for vertical force sensing*

The traditional structure (lateral structure) of piezotronic transistors for vertical force sensing has remained almost unchanged since 2007, as exhibited in **Figure R18a**.

Our previous work using this lateral structure is to propose piezotronic tunneling junction for highly sensitive strain sensing [18], which can achieve a high gauge factor of 4.8×10^5. It completely used the traditional lateral structure, and its novelty lies in the proposal of piezotronic tunneling junction based nanodevices, which utilizes the strain-induced piezoelectric potential to control the tunneling barrier height and width in parallel, and hence to synergistically modulate the electrical transport process and improve the performance. One reason why DPT does not use the piezotronic tunneling junction is that the quantitative regulation mechanism of piezotronic tunneling junction is still not clear, which will interfere with our research on DPT. The application of piezotronic tunneling junctions to DPT may have a higher response, which maybe an interesting research topic in the future. In fact, the design of DPT is a new structure of

piezotronic transistor, which can be used to replace most of previous piezotronic devices based on nano/microwires.

In summary, the high performance of DPT for vertical force sensing is due to **advanced structural design** (containing suspension-bridge-inspired structure and introduction of microprotrusion), **perfect solution to the problem of buckling effect** (existed in traditional vertical structure), and **introduction of microprotrusion**, while the performance of DPT for lateral strain sensing is at the upper middle level in the field of piezotronics, which results from the **introduction of the bottom PDMS layer**.

We thank the reviewer again for this constructive and nice suggestion. To improve the quality of this work, we have revised the manuscript and added above discussions about the structural evaluation of previously reported piezotronic transistors and our original research idea into the revised Supplementary Information and marked them in red.

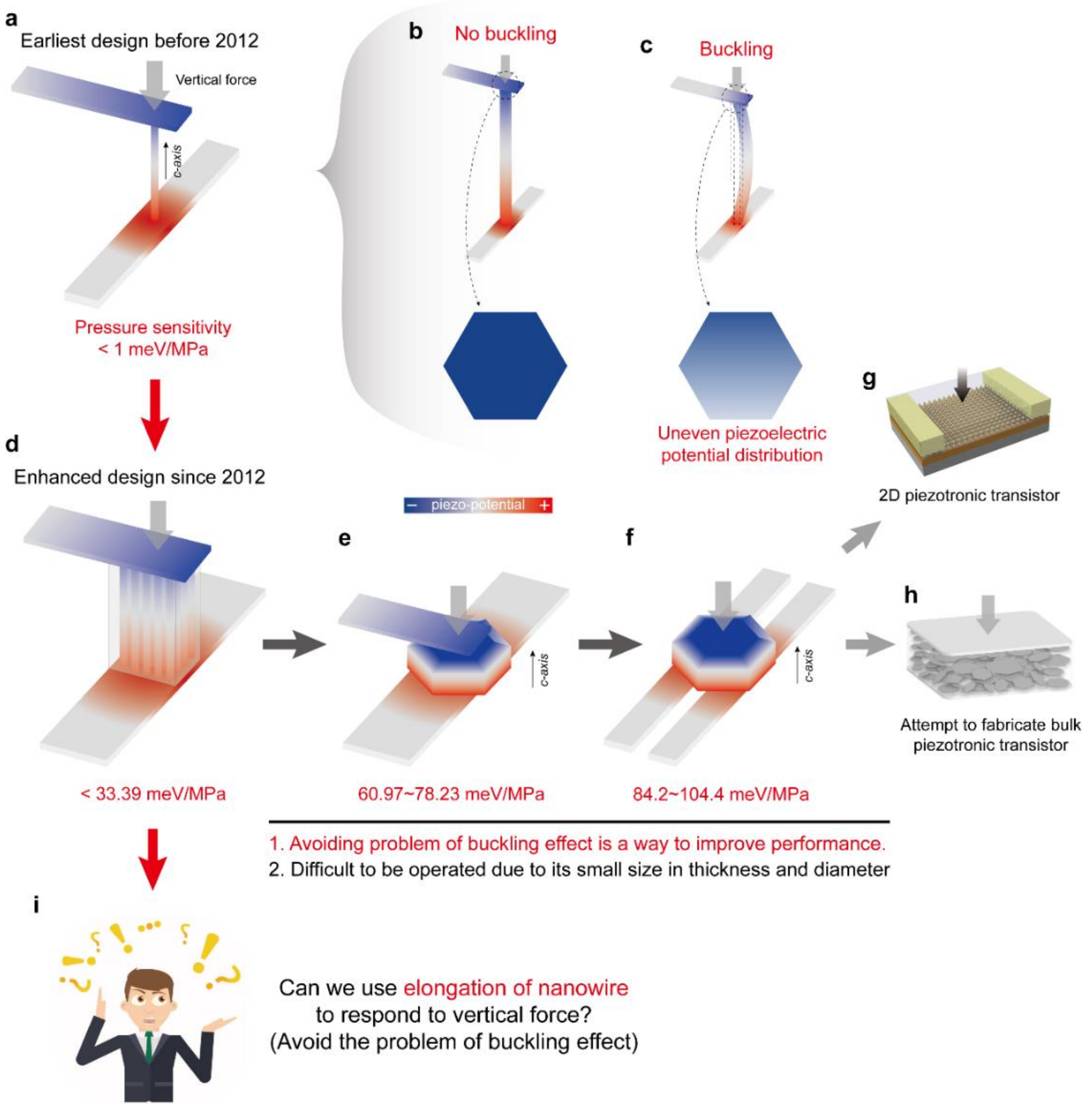

Figure R33. **Structural evolution of piezotronic transistors for vertical force sensing. a**, A piezotronic transistor with a vertical structure composed of a vertical aligned nano/microwire sandwiched by two electrodes. **b**, **c**, Illustrations of vertical force induced piezoelectric potential distributions without (**b**) and with (**c**) considering the buckling effect. The enlarged insets are illustrations of the potential distributions on the upper end of the nano/microwire. **d**, An enhanced design of piezotronic transistor based on nano/microwire cluster encapsulated by PDMS. Bounded by the PDMS and the nano/microwires around, vertical nano/microwire in the cluster is not easy to be bent (buckling effect) upon a vertical compressive force, which can enhance the performance to a certain extent. The still existing buckling effect and the inconsistency of nano/microwires hinder the further improvement of performance. **e**, A design based on nanoplatelet to solve the problem of buckling effect. **f**, A design based on

nanoplatelet with two bottom electrodes to achieve synergistically regulations of two Schottky barrier by same piezoelectric polarization charges/potentials. It is difficult to manipulate the nanoplatelets for practical application. **g**, **h**, Attempts towards two-dimensional piezotronic transistors (**g**) and bulk statistical piezotronic transistor (**h**) based on ZnO nanoplatelets. The pressure sensitivity of these two kinds of piezotronic transistors is not as good as that based on a single nanoplatelet in (**e**) and (**f**), but the special piezotronic channel-gating mechanism at two-dimension and statistical piezotronic effect are found. **i**, A question raised after investigating the structural evolution of traditional piezotronic transistors to avoid the problem of buckling effect: can we use elongation of nano/microwire to respond to vertical force?

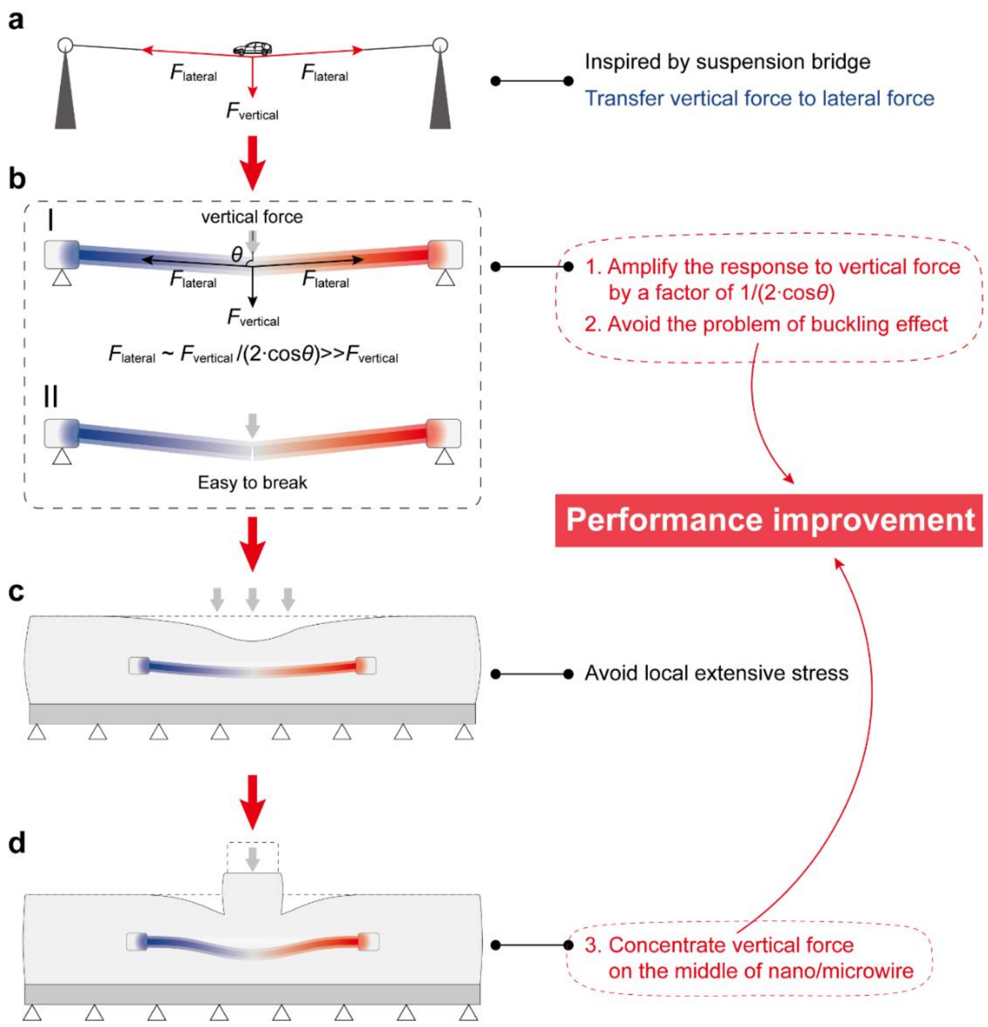

Figure R34. Origin of the design idea of dual-modal piezotronic transistor (DPT). **a**, A suspension-bridge-inspired structure that can transform the vertical force to the

elongation of rope, and thus achieve elongation of rope to respond to vertical force. **b**, The earliest structure of DPT that can amplify the response to the vertical force. This DPT structure can amplify the response to vertical force by a factor of $1/(2 \cdot \cos\theta)$ and avoid the problem of buckling effect caused by compression deformation of vertical nano/microwires. Directly applying the vertical force on the lateral nano/microwire can easily lead to the fracture of the nano/microwire. **c**, Using PDMS to encapsulate the lateral nano/microwire to avoid extensive local stress. **d**, Final design of DPT. Introduction of the microprotrusion makes the vertical force concentrate on the middle of the nano/microwire, thus more effectively elongating the nano/microwire.

Reference:

1. Zhang, Y., Liu, Y. & Wang, Z. L. Fundamental theory of piezotronics. *Advanced Materials* **23**, 3004-3013 (2011).
2. Pan, C., Zhai, J. & Wang, Z. L. Piezotronics and piezo-phototronics of third generation semiconductor nanowires. *Chemical Reviews* **119**, 9303-9359 (2019).
3. Wu, W. & Wang, Z. L. Piezotronics and piezo-phototronics for adaptive electronics and optoelectronics. *Nature Reviews Materials* **1**, 16031 (2016).
4. Wu, W., Wen, X. & Wang, Z. L. Taxel-addressable matrix of vertical-nanowire piezotronic transistors for active and adaptive tactile imaging. *Science* **340**, 952-957 (2013).
5. Lee, J. H. *et al.* Heterogeneous structure omnidirectional strain sensor arrays with cognitively learned neural networks. *Advanced Materials* **35**, 2208184 (2023).
6. Zhou, J. *et al.* Flexible piezotronic strain sensor. *Nano Letters* **8**, 3035-3040 (2008).
7. Liu, K., Sakurai, M. & Aono, M. Enhancing the humidity sensitivity of Ga_2O_3/SnO_2 core/shell microribbon by applying mechanical strain and its application as a flexible strain sensor. *Small* **8**, 3599-3604 (2012).
8. Tavassolian, M., Cuthbert, T. J., Napier, C., Peng, J. & Menon, C. Textile-based inductive soft strain sensors for fast frequency movement and their application in wearable devices measuring multiaxial hip joint angles during running. *Advanced Intelligent Systems* **2**, 1900165 (2020).

9. Wang, Z. L. Nanopiezotronics. *Advanced Materials* **19**, 889-892 (2007).
10. Zhou, Y. S. *et al.* Vertically aligned CdSe nanowire arrays for energy harvesting and piezotronic devices. *ACS Nano* **6**, 6478-6482 (2012).
11. Han, W. *et al.* Strain-gated piezotronic transistors based on vertical zinc oxide nanowires. *ACS Nano* **6**, 3760-3766 (2012).
12. Zhao, Z. *et al.* Piezotronic effect in polarity-controlled GaN nanowires. *ACS Nano* **9**, 8578-8583 (2015).
13. Liu, S. *et al.* Ultrasensitive 2D ZnO piezotronic transistor array for high resolution tactile imaging. *Advanced Materials* **29**, 1606346 (2017).
14. Liu, S. *et al.* Double-channel piezotronic transistors for highly sensitive pressure sensing. *ACS Nano* **12**, 1732-1738 (2018).
15. Wang, L. *et al.* Ultrathin piezotronic transistors with 2 nm channel lengths. *ACS Nano* **12**, 4903-4908 (2018).
16. Wang, L. *et al.* 2D piezotronics in atomically thin zinc oxide sheets: interfacing gating and channel width gating. *Nano Energy* **60**, 724-733 (2019).
17. Liu, S. *et al.* Statistical piezotronic effect in nanocrystal bulk by anisotropic geometry control. *Advanced Functional Materials* **31**, 2010339 (2021).
18. Yu, Q. *et al.* Highly sensitive strain sensors based on piezotronic tunneling junction. *Nature Communications* **13**, 778 (2022).

Reviewer #3 (Remarks to the Author):

In this work, the authors proposed a dual-modal piezotronic transistor (DPT) that can simultaneously sense vertical force and horizontal strain. This design exhibits a higher sensitivity than similar work. However, there are several major flaws within this article, which needs to be addressed before further consideration.

Response:

We highly appreciate the reviewer for acknowledging the importance of our work and commenting that "This design exhibits a higher sensitivity than similar work". And we also thank the reviewer for the detailed and valuable comments/suggestions about our work, which reminds us what work is not done well, and promotes us to condense the

novelty of DPT and improve the research details better.

Question #1

The major technical flaw for this manuscript is that although this design can sense both the vertical force and lateral strain, it is not able to differentiate whether the force or the strain are applied. The author needs to present the data when both vertical force and lateral strain are applied together and present the data of cross talk between the two types the sensors.

Response:

We thank the reviewer very much for this valuable and constructive suggestion. It is an interesting and important issue considering the crosstalk between the vertical force and lateral strain sensing signals. Because both the vertical force sensing and the lateral strain sensing utilize the deformation of ZnO nano/microwire to produce piezoelectric polarizations at interface to control the barrier height and electrical transport, we cannot distinguish in theory whether the deformation of ZnO nano/microwire is caused by the vertical force or the lateral strain. As the reviewer suggested, we have tried to carry out a number of experiments but unfortunately found that the two response signals are difficult to be decupled. As shown in **Figure R35a**, with a fixed lateral strain, an applied vertical force will increase the current; while, as the fixed lateral strain become bigger, the *I*-*t* curve will decrease as a whole. Judging from the electrical transport signal alone, we cannot decouple the two signals for the time being.

Since 2007, achieving a piezotronic transistor (sensor) with two working modes that can switch between the two working modes according to the needs of the scene is a long-time challenge in the field of piezotronics. This work focuses on overcoming the above challenge, and improving the pressure sensitivity of vertical force sensing and possessing a good lateral force sensing performance (Note: please see the **response to Question #3** for detailed discussion). Besides this innovation, we believe that the dual-modal piezotronic transistor (DPT) will have a wider range of applications and a greater impact on the structural design of future piezotronic transistors if the two signals can be furtherly decoupled no matter by principle design or signal processing. This issue about DPT provided by the reviewer will be a significant step in the next generation of

piezotronic transistors.

At current stage, although we cannot solve the problem of crosstalk when both vertical force and lateral strain are applied together, we think that the difference in the bending shape of nano/microwires in the two working modes is a possible breakthrough. In lateral strain sensing, **the nano/microwire as a whole** is bent or stretched and compressed; while in vertical force sensing, **the middle of the nano/microwire** bends downward. Based on our understanding on piezotronic modification of energy band, this difference in bending shape (**Figure R35b**) will make the piezoelectric potential distribution together with the energy band bending along the nano/microwire different, which may lead to different impedance. Through investigating the piezotronic modification of the capacitance-voltage characteristics may we obtain the possible approach to decouple the two sensing signals by technologies like behavior-learned cognition algorithm [1]. However, its complexity requires lots of further in-depth investigations.

To avoid misunderstanding and expectations beyond experiments to readers, we put forward the unsolved problem of crosstalk and prospect of decoupling the two signals, and added corresponding descriptions and above discussion in the revised manuscript and the revised supplementary information.

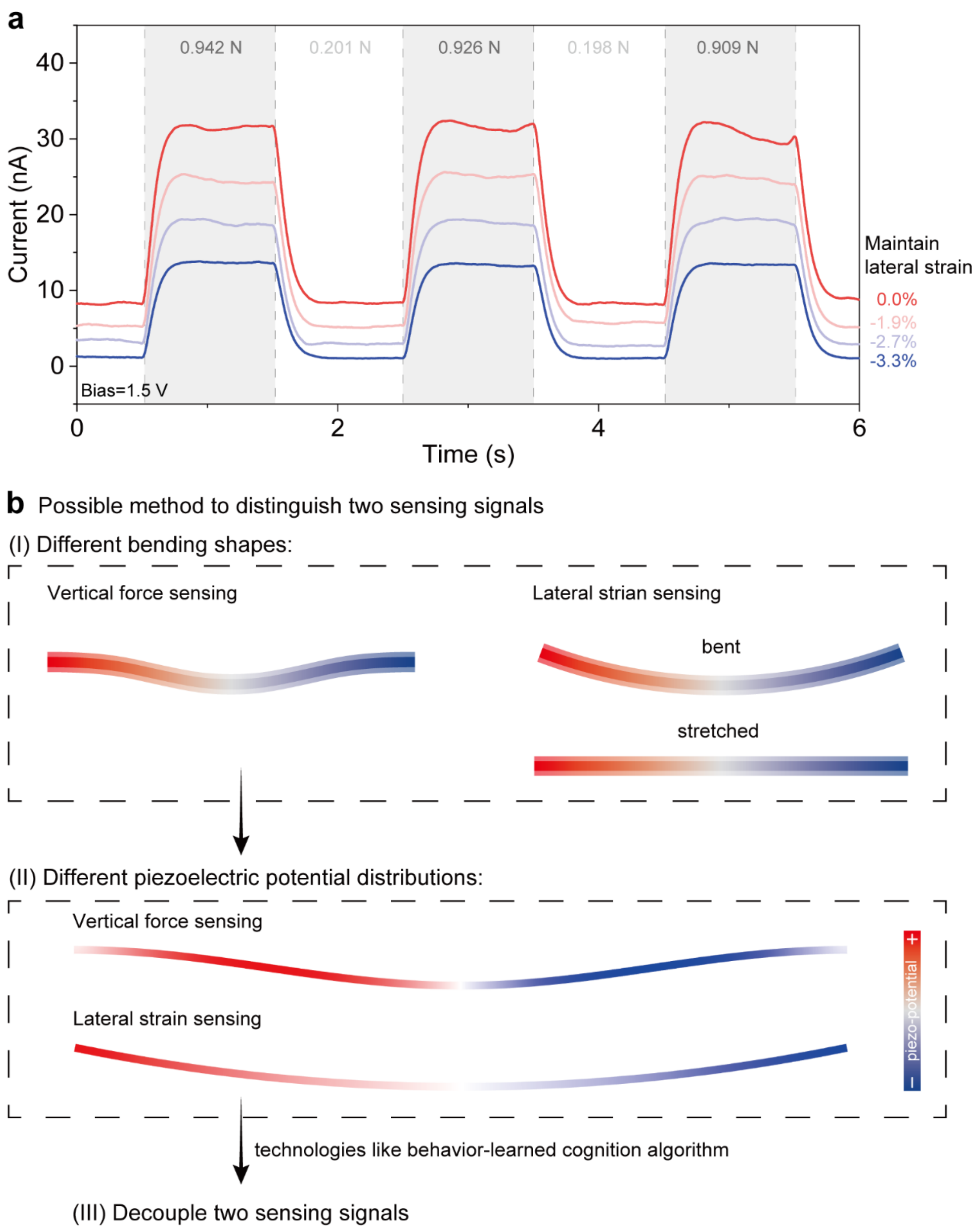

Figure R35. Dynamic current response of DPT to periodic vertical force with various fixed strains and problem of crosstalk. a, Dynamic current response to periodic vertical force (~0.2 N to ~0.9 N) with various fixed strains (0.0%, -1.9%, -2.7% and -3.3%). It is difficult to decouple the effect of vertical force and lateral strain, by judging from the electrical transport alone. **b**, A possible method to distinguish the two sensing signals. Different bending shape (I) will make the piezoelectric potential distribution (II) different, which may lead to different impedance (III).

Question #2

The schematic diagram of the DPT device is basically used in the full text, so that the reviewer does not know what the specific device looks like. The authors should provide an optical image of the sensor. Additionally, how the external electrical connection can be made with the sensor?

Response:

We thank very much for the reviewer's constructive suggestions. **Figure R36** shows the DPT device and the external electrical connection in detail. The specific home-made experiment setup together with the optical image of the DPT is shown in **Figure R36a.** The data acquisition card (National Instruments) is used for data acquisition and input the data to the computer. The synthesized function generator (Stanford Model DS 345) is applied to produce a drive voltage on DPT. With the help of shielded junction box, the voltage signal is also imported into the data acquisition card. Meanwhile, the low-noise current preamplifier (Stanford Model SR570) is used to measure the current flow through the DPT and also import the current value into the data acquisition card. In the specific test process for vertical force sensing, a linear actuator is controlled by the computer to apply a displacement on the DPT, and a force senor under the substrate readouts the force applied on DPT at the same time. The illustration of electrical connection is also provided in **Figure R36b** for a more intuitive display. And the circuit diagram is shown in **Figure R36c**.

Thank the reviewer again for this valuable suggestion. The above optical image of device and specific electrical connection have been added into the revised Supplementary Information and marked in red.

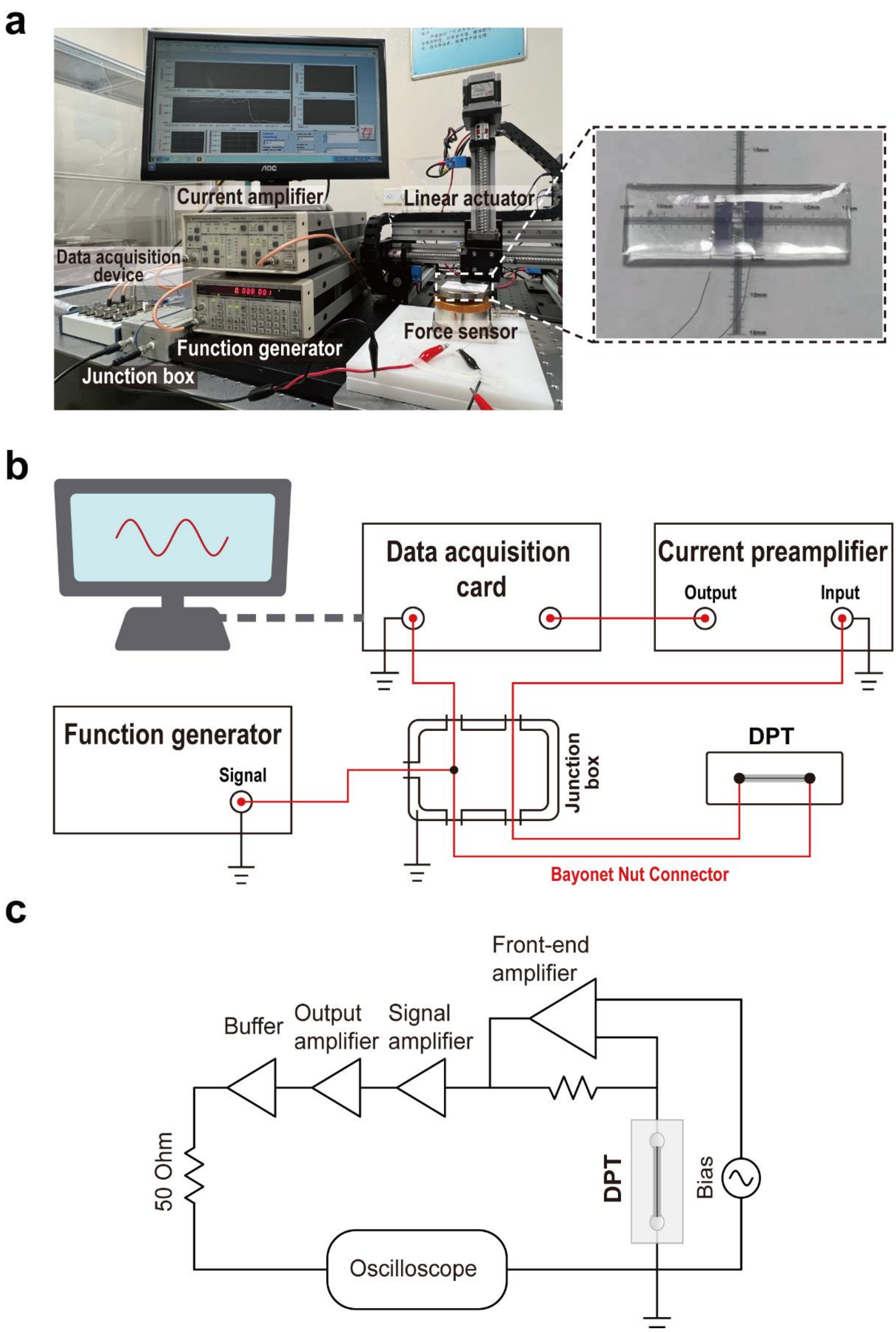

Figure R36. Experimental setup. a, Home-made experiment setup composed of functional generator, current preamplifier, junction box, data acquisition card and computer. Inset: Optical image of DPT. b, Illustration of external electrical connection. **c**, Circuit diagram of measurement.

Question #3

The traditional pressure and strain sensing methods in Figure 1 have been widely known by researchers. Similar design without microprotrusion has been published even before 2010. Besides supplementary table 2, the authors should discuss the novelty of their design.

Response:

We appreciate the reviewer for this constructive comment and suggestion very much. And, we are sorry that the novelty of our design on the dual-modal piezotronic transistor (DPT) is not discussed more clearly and revealed in-depth in the manuscript. Although the structure of DPT, especially in lateral strain sensing mode (Figure 1b-II) is very similar to the traditional lateral structure (for lateral strain sensing only, Figure 1a-II), DPT in Figure 1b utilizes a lateral nano/microwire to achieve not only lateral strain sensing (a long-standing usage since 2007 in piezotronics) but also vertical force sensing (key point, never achieved before in piezotronics). Through the design of DPT, a new lateral structure (DPT) can be used to sense vertical force, which is the key point in this work and makes a big change/improvement in the field of piezotronics. Actually, DPT is not a simple structural optimization including the introduction of microprotrusion, but a new design in structure and in principle, which can perfectly solve the long-term existed challenges/problems in traditional piezotronic transistors and has structural advantages in amplifying the detection of vertical force, leading to a qualitative leap in both performance improvement and functional expansion in piezotronics. The main novelty of DPT lies on the functional expansion of piezotronic transistors, making it possess two working modes for lateral strain sensing and vertical force sensing. Another novelty of DPT is to achieve performance improvement of piezotronic transistors through utilizing the elongation of the lateral nano/microwire (rather than the compression of the vertical nano/microwire) caused by vertical force to realize the detection of the vertical force.

In order to explain the novelty of DPT more clearly, here we will further describe (1) structural evolution of traditional piezotronic transistors for vertical force sensing, and exhibit (2) origin of the design idea of DPT, and finally show (3) novelty of the

design of DPT. It is expected that the novelty of DPT can be fully revealed through this discussion.

(1) Structural evolution of traditional piezotronic transistors for vertical force sensing

In 2007, the earliest design of utilizing piezotronic effect to sense vertical force is illustrated in **Figure R37a** or Figure 1a, in which a vertical aligned ZnO single crystal nano/microwire sandwiched and Schottky contacted by two electrodes acts as the core unit. As ZnO is a kind of piezoelectric semiconductor, according to the direction of its piezoelectric polarization axis (*c*-axis) as shown in the **Figure R37a**, positive and negative piezoelectric polarization charges and corresponding piezoelectric potentials will be produced at the upper end and the lower end of ZnO nano/microwire respectively when compressed by a vertical force. Based on the piezotronic effect, these piezoelectric charges/potentials will modulate the Schottky barrier heights at two ends, and hence control the electrical transport of the nano/microwire. Since linear relationships between the force, the piezoelectric potential and the Schottky barrier height, we can achieve detection of the applied vertical force through measuring the change of *I-V* characteristics. However, as subjected to a vertical compressive force, the nano/microwire will not only sustain a compressive deformation along its length direction but also a bending deformation, which is known as buckling effect as shown in **Figure R37b**. In this case, part of the vertical force will be wasted to bend the nano/microwire. In other words, the buckling effect will weaken the nano/microwire's compressive deformation along its length direction and the corresponding piezoelectric potentials generated at two ends of nano/microwire, resulting in a negative influence on the performance of piezotronic effect. Apart from above, the buckling effect will also cause the bending nano/microwire with one side stretched and the other compressed, making an uneven piezoelectric potential distribution at the end of the nano/microwire as illustrated in the inset of **Figure R37b**, and hence a nonuniform piezotronic modulation on the Schottky barrier. Since the electrical transport of the interface is determined by the region that possesses the lowest barrier height, the buckling effect induced nonuniform modulation also has a negative influence on the

performance of piezoelectric effect.

As a result of above two negative influences of buckling effect, early piezotronic transistors based on vertical nano/microwires for vertical force sensing exhibits low pressure sensitivities (CdSe nano/microwire [2]: 0.677 meV/MPa; ZnO nano/microwire [3]: 0.083-0.422 meV/MPa; GaN nano/microwire [4]:0.212-0.240 meV/MPa). This design kept unchanged from 2007 to 2012.

In 2012, nanowire clusters encapsulated by PDMS are utilized to act as the core unit of piezotronic transistor as shown in **Figure R37c**, which can inhibit the buckling effect to some extent. Nano/microwire in the cluster bounded by the nano/microwires around and the PDMS is not easy to buckle under the action of vertical compressive force. Based on this improvement of mechanical structure, the piezotronic transistor exhibits a relatively high pressure sensitivity up to 33.39 meV/MPa (ZnO nanowire cluster [5]).

In 2017, another strategy of replacing vertical nano/microwire or nano/microwire cluster with nanoplatelet is proposed to solve the problem of buckling effect as shown in **Figure R37d** [6]. The nanoplatelet with an aspect ratio (thickness-to-diameter) of approximately 0.42 will not buckle as the vertical nano/microwire upon a vertical compressive force. The vertical compressive force induced compressive deformation is strictly along the direction of the nanoplatelet thickness that is also the piezoelectric polarization axis (*c*-axis in ZnO). The piezotronic transistor based on ZnO nanoplatelet possesses pressure sensitivity of 60.97~78.23 meV/MPa, which is a big improvement compared with the ones based on vertical nano/microwires or nano/microwire clusters. However, due to nanoplatelets' small size both in thickness and diameter, fabricating piezotronic transistor based on nanoplatelets is very difficult. Additionally, how to design a device structure to achieve the vertical force/pressure evenly applied on the upper surface of the nanoplatelet is another problem that persuades further improvement of pressure sensitivity.

It should be noted that, piezotronic transistors based on vertical aligned nano/microwires, nano/microwires clusters and nanoplatelets all utilize the compressive deformation caused by vertical compressive force to produce piezoelectric

charges/potentials and thus to control electrical transport. This is totally different from the design of DPT, which will be discussed later in the following section (2). For the vertical aligned nano/microwire or nano/microwire cluster, there inevitably exists the problem of buckling effect upon vertical compressive forces; whereas for the nanoplatelet, although there is no problem of buckling effect, its unique geometric characteristics makes it difficult to furtherly improve the performance. Finding schemes to avoid the problem of buckling effect in nano/microwire -based piezotronic transistor is important for their applications in sensing.

(2) Origin of the design idea of DPT

From above discussions about methods/developments of improving the performance of piezotronic transistors, we can find that buckling effect during compressive deformation caused by the vertical compressive force has negative influences on piezotronic effect. A question arises (**Figure R38a**), that is, can we utilize the elongation of nano/microwires rather than the compression of nano/microwires to achieve the detection of vertical force. Inspired by the suspension bridge (**Figure R38b**), we designed a new structure composed of a lateral nano/microwire with two ends constrained by electrodes as shown in **Figure R38c-I**. When a vertical force is applied on the middle of the lateral nano/microwire, the nano/microwire will be bent downward and elongated. This kind of structure utilizing the response of lateral nano/microwire to the applied vertical force can transform the vertical force to lateral force, and thus to realize vertical force sensing.

It is worth noting that the bending of lateral nano/microwire here is totally different from the previously discussed bending of vertical nano/microwire. In the former case, the two ends of the nano/microwire are constrained, and the vertical force elongates the nano/microwire and thus induce piezoelectric charges/potentials through downward bending the nano/microwire. As a comparison, in the latter case, the bending of nano/microwire caused by the vertical compressive force will reduce the net compressive deformation along the length of the nano/microwire and decrease the generations of piezoelectric charges/potentials. So, from the perspective of solving the existing problem in piezotronics, this new design does not have the problem of buckling

effect in traditional piezotronic transistors.

In addition, applying a very simple model (**Figure R38c-I**) to estimate, we can find that this newly designed structure should have high response in the detection of the vertical force. As a small vertical force $F_{vertical}$ is applied on the middle of the nano/microwire, a very large tensile force $F_{lateral}$ approximately equaling to $F_{vertical}/(2 \cdot \cos\theta)$ (θ is the angle between nano/microwire and vertical line) would be produced along the length of the nano/microwire. Since θ approaches 90°, $F_{lateral}$ ($\approx F_{vertical}/(2 \cdot \cos\theta)$) is a very large value. Although this estimation model is very rough, it is qualitatively estimated that a small vertical force can indeed cause the nanowire to tighten and produce a very large tensile force along the axial direction. So, from the perspective of structural design and principle, this new design exhibits a function of amplifying the vertical force on the nano/microwire by a factor of $1/(2 \cdot \cos\theta)$ and should have a high response to vertical force.

Our earliest design is illustrated in **Figure R38c-II**. By applying a very small vertical force, we found that the nano/microwire would produce a very large tensile deformation along its length direction together with large local stresses particularly at the positions of the force point and electrodes, and was very likely to break into two sections. In other words, the device is very easy to damage.

Next, we designed a structure as shown in **Figure R38d**, and used PDMS to wrap the nano/microwire. The soft nature of PDMS allows the nano/microwire to sustain a large enough tensile stress and avoids the nano/microwire fracturing when subjected to very large local stresses (especially at the positions of the force point and electrodes). Based on this design, we successfully achieve devices based on the response of lateral nano/microwire to the vertical force.

In order to furtherly enhance the influence of the vertical force, a microprotrusion as shown in **Figure R38e** is added to make the vertical force mainly concentrate on the middle part of the nano/microwire, thus more effectively elongating the nano/microwire rather than pressing the whole nano/microwire down.

By comparing the differences and progressive relationships between this work and the previous works, we can see that the DPT has the following features:

I. it has a basic principle of utilizing the response of lateral nano/microwire to vertical force to realize the vertical force sensing;

II. it utilizes elongation of nano/microwires (rather than compression of nano/microwires) to sense vertical force, which properly solves the problem of buckling effect in traditional vertical structure.

III. the suspension-bridge-inspired structure of DPT can amplify the vertical force to the lateral/tensile force along the nano/microwire;

IV. the soft nature of PDMS avoids local extensive stress;

V. the introduction of microprotrusion makes the vertical force concentrate on the middle of the nano/microwire, thus more effectively elongating the nano/microwire under the action of vertical force.

As shown in **Figure R38**, considering that the lateral nano/microwire structure can be intrinsically used to sense lateral strain, the above feature I results in the functional expansion of DPT, and makes DPT possessing two working modes for both vertical force sensing and lateral strain sensing. Additionally, the features II, III and V together make DPT exhibit high pressure sensitivity to vertical force compared with previous designs (Supplementary Table 4 in revised Supplementary Information).

From the above discussion about the origin of the design idea of DPT, it can be found that, **besides the microprotrusion, the suspension-bridge-inspired lateral structure plays a critical role in the functional expansion and performance improvement of DPT and is totally different from traditional lateral structure.**

(3) Novelty of the design of DPT

The new design of DPT promotes piezotronic transistor with both merits of performance improvement and functional expansion. **Figure R39** summaries the novelty of the new design of DPT.

① *Performance improvement: high pressure sensitivity*

Based on the above discussed features II, III and V of the design, the DPT proposed in this work achieves the pressure sensitivity of 1.759 eV/MPa, which is 16.5 times more than that of previously reported piezotronic transistors.

As illustrated by the blue part in **Figure R39**, high performance of DPT is not only

due to the **introduction of microprotrusion**, but also results from the perfect **solution to the problem of buckling effect**, and **advanced structural design** (containing suspension-bridge-inspired structure and introduction of microprotrusion) of amplifying vertical force to tensile force along the length of the nano/microwire.

② *Functional expansion: two working modes (main novelty of DPT)*

More importantly, in the past, traditional piezotronic transistor based on vertical structure can only be used for vertical force sensing while based on lateral structure can only be used for lateral strain sensing. These two structures can detect vertical force or lateral strain separately, while not meet the demand of actual applications that require the same sensor to switch between two working modes. This dilemma has hindered the development and application of piezotronic transistors for a long time. Fortunately, the design of DPT realizes the detection of vertical force with ultrahigh performance based on a new lateral structure; Meanwhile, similar to the traditional lateral structure shown in Figure 1a-II, the new design itself has a lateral structure with all the advantages of a lateral structure, which makes the new design of DPT naturally suitable for lateral strain sensing. As a result, DPT with the new lateral structure that enables two working modes, one is vertical force sensing and the other is lateral strain sensing. DPT inherits all the advantages of traditional lateral structure for lateral strain sensing, and can also sense vertical force without the problem of buckling effect in traditional vertical structure.

As illustrated by the red part in **Figure R39**, the design of DPT successfully overcomes the dilemma that piezotronic transistor can only be used for vertical force sensing or only be used for lateral strain sensing, and for the first time enables piezotronic transistor to possess two working modes and more possibilities in practical application, and subverts the structural design of traditional piezotronic transistors, which can provide new ideas for new principle devices in the field of piezotronics and promote the application of piezotronic effect.

In summary, through the above introductions of structural evolution of traditional piezotronic transistors and our design idea of DPT, **DPT enables the piezotronic transistor to possess two working modes for the first time and achieves higher**

performance by solving problem that exists in previous designs, which makes a qualitative leap in both function and performance of piezotronic transistors.

We are very grateful to the reviewer for this very constructive suggestion, which promotes us to better condense the novelty of our design. To improve the quality of this manuscript, we have revised and added descriptions of the novelty of DPT in the manuscript and marked them in red, and also added the above discussions in the revised Supplementary Information (red marked).

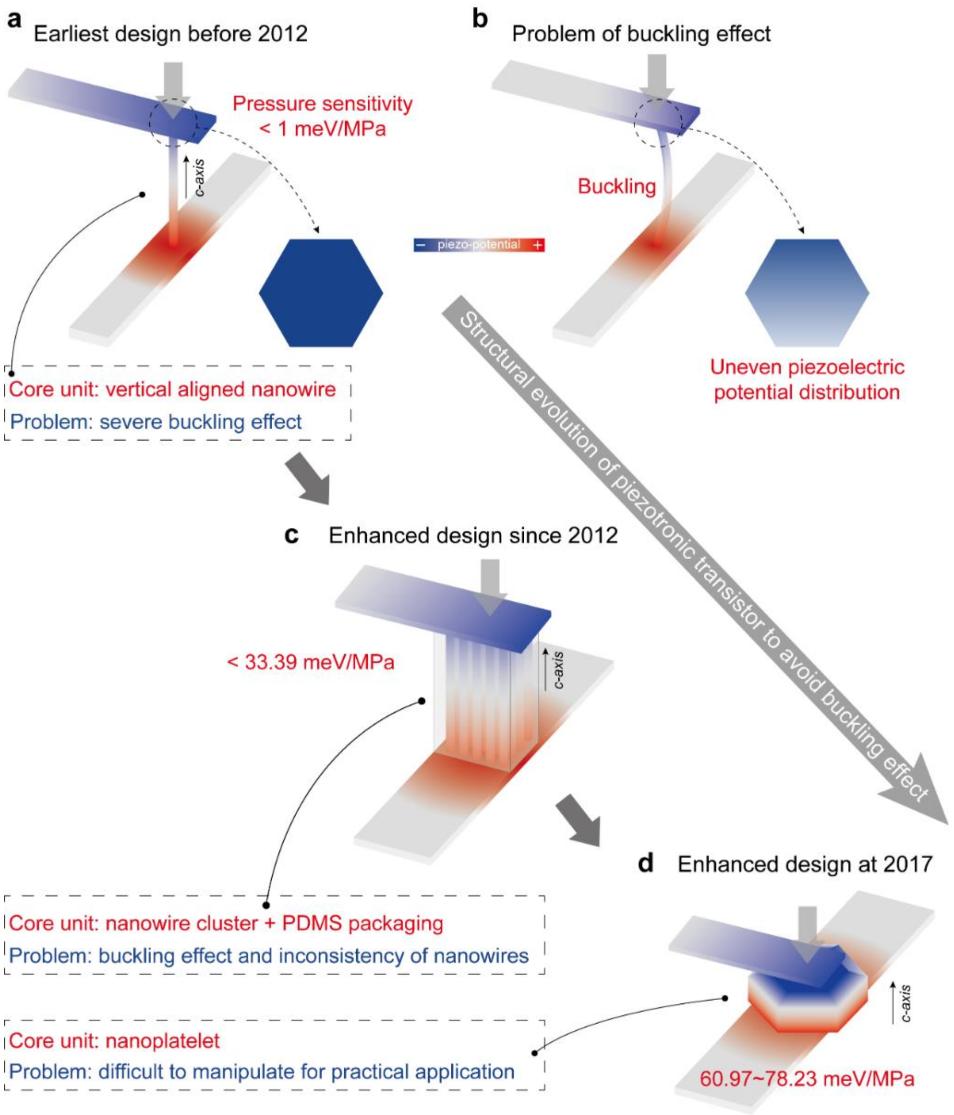

Figure R37. **Structural evolution of traditional piezotronic transistors for vertical force sensing. a**, A piezotronic transistor with a vertical structure composed of a vertical aligned nano/microwire sandwiched by two electrodes. **b**, Illustrations of vertical force induced piezoelectric potential distributions with considering the buckling effect. The

enlarged insets are illustrations of the potential distributions on the upper end of the nano/microwire. **c**, An enhanced design of piezotronic transistor based on nano/microwire cluster encapsulated by PDMS. Bounded by the PDMS and the nano/microwires around, vertical nano/microwire in the cluster is not easy to be bent (buckling effect) upon a vertical compressive force, which can enhance the performance to a certain extent. The still existing buckling effect and the inconsistency of nano/microwires hinder the further improvement of performance. **d**, A design based on nanoplatelet to solve the problem of buckling effect. It is difficult to manipulate the nanoplatelets for practical application.

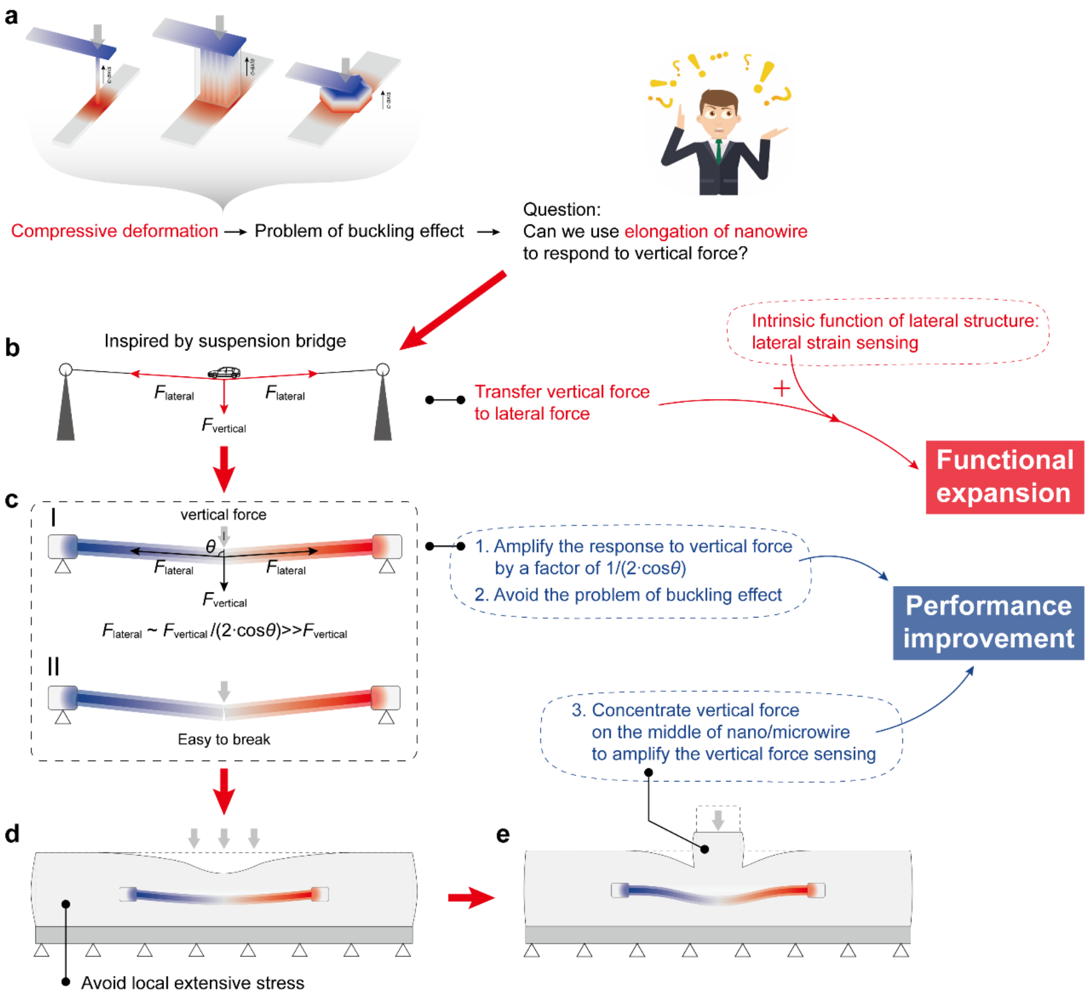

Figure R38. Origin of the design idea of the dual-modal piezotronic transistor (DPT). a, Traditional vertical structures with a common feature of utilizing compressive deformation caused by vertical force to sense vertical force. This sensing principle is easy to induce the problem of buckling effect. A question arises, that is, can

we use elongation of nano/microwires to respond to the vertical force? **b**, A suspension-bridge-inspired structure that can transfer the vertical force to the elongation of wire, and thus achieve elongation of nano/microwire to respond to vertical force. **c**, The earliest structure of DPT that can amplify the response to the vertical force. Directly applying the vertical force on the lateral nano/microwire can easily lead to the fracture of the nano/microwire. **d**, Using PDMS to encapsulate the lateral nano/microwire to avoid extensive local stress. **e**, Final design of DPT. Introduction of the microprotrusion makes the vertical force concentrate on the middle of the nano/microwire, thus more effectively elongating the nano/microwire under the action of vertical force.

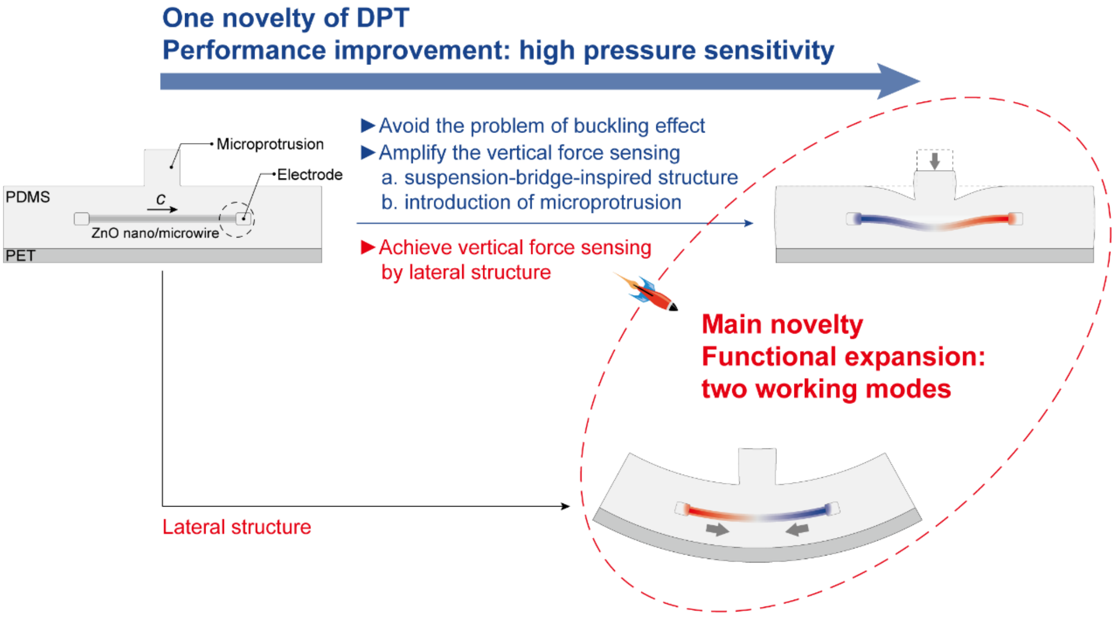

Figure R39. Novelty of the design of the dual-modal piezotronic transistor (DPT). One novelty (blue part): DPT possesses high pressure sensitivity resulting from the perfect solution to the problem of buckling effect and advanced structural designs containing the suspension-bridge-inspired structure and introduction of microprotrusion that can amplify the vertical force sensing. **Main novelty (red part)**: DPT enables one piezotronic transistor with two working modes for vertical force sensing and lateral strain sensing, which benefits from the new lateral structure design of DPT.

Question #4

Supplementary Tables 2 and 3 compare the pressure sensing and strain sensing capabilities of the DPT devices fabricated in this paper with other studies. However, the advantages of DPT in pressure sensing comparison and strain sensing comparison are difficult to see directly from the table. It is recommended to draw a two-dimensional graph based on both Supplementary Table 2 and 3, with pressure sensing and strain sensing as the coordinate axes, respectively, to show the positions of the DPT and the comparison materials in this paper. Additionally, the authors only compare with piezotronic sensors and should compare with other pressure and strain sensing mechanism.

Response:

We are very grateful for the reviewer's valuable and constructive suggestions. Here, we will try to draw graphs to exhibit the positions of the DPT and compare the performance of DPT with other sensors (including piezoresistive sensors and capacitive sensors).

In general, based on different sensing mechanisms, parameters used to show the performance of pressure and strain sensors with different principle have different definitions. For example, piezotronic effect utilizes strain/force-induced piezoelectric polarization at interface to modulate the interface barrier height thus to control the electrical transport. As a result, a parameter defined as 'pressure sensitivity' (formula (29)) is applied to characterize the ability of mechanical stimuli on modulating the barrier height, and possesses a unit of eV/MPa. This parameter is only applicable to sensors with interface barrier modulated by strain/force. In addition, lots of sensors in literatures can only be used to sense strain or sense force, and usually characterized by different parameters, such as 'gauge factor' (with a unit of 1, defined as following formula (30)) and 'sensitivity' (with a unit of Pa^{-1}, formula (31)).

$$Pressure\ sensitivity = \frac{\Delta BH}{\Delta P} \tag{29}$$

in which ΔP is the change in pressure, and ΔBH is the barrier height change caused by ΔP.

$$Sensitivity = \frac{\Delta I/I_0}{\Delta P} \ or \ \frac{\Delta C/C_0}{\Delta P} \ or \ \frac{\Delta R/R_0}{\Delta P} \tag{30}$$

in which ΔP is the change in pressure, and ΔI, ΔC, ΔR are respectively the change in current, capacitor and resistance caused by ΔP, and I_0, C_0, R_0 are respectively the original current, capacitor and resistance.

$$Gauge\ factor = \frac{\Delta I/I_0}{\Delta \varepsilon} \ or \ \frac{\Delta R/R_0}{\Delta \varepsilon} \tag{31}$$

in which $\Delta \varepsilon$ is the change in strain, and ΔI, ΔR are respectively the change in current and resistance caused by $\Delta \varepsilon$, and I_0, R_0 are respectively the original current and resistance.

As the reviewer suggested, we tried to draw a two-dimensional graph based on both Supplementary Table 2 and Table 3 with pressure sensing and strain sensing as the coordinate axes, but found it difficult to obtain the two-dimensional graph due to the fact that few literatures provide both parameters regarding vertical force/pressure sensing and lateral strain sensing. In order to do our best to intuitively exhibit the position of DPT in comparison with other sensors, we have summarized their performance parameters and plotted four graphs as shown in the following **Figure R40**.

(1) Force sensing performance (pressure sensitivity and sensitivity)

Figure R40a gives the pressure sensitivity of DPT in comparison with previous piezotronic transistors in sensing force/pressure. The red-labeled data points are piezotronic transistors made of nano materials, and the blue-labeled data points are piezotronic transistors made of bulk materials. As can be seen, the ZnO nano/microwire-based DPT shows a pressure sensitivity of about 1.759 eV/MPa, which is a very high value in piezotronics.

Figure R40b shows the sensitivity of DPT in comparison with some other piezotronic sensors, piezoresistive sensors and capacitive sensors in last several years in sensing force/pressure. We can find that the sensitivity of DPT is also a high value in various sensors based on other force/pressure sensing mechanism.

(2) Strain sensing performance (pressure sensitivity and sensitivity)

Figure R40c and **R40d** exhibit the gauge factor of DPT in comparison with some other piezotronic sensors, piezoresistive sensors and capacitive sensors in sensing strain. The highest gauge factor of DPT is about 8988 at a compressive strain of 5.8%, which is a high value in strain sensing. In fact, as previously discussed in **response to Question#3**, the novelty of DPT mainly focus on its new design for vertical force sensing and the resulting in performance (vertical force sensing) improvement and functional expansion; therefore, the gauge factor of DPT is in the upper state among strain sensors with various principle, which indicates that DPT is also a good lateral strain sensor in addition to being a good vertical force sensor.

In summary, **Figure R40** exhibits the positions of DPT in vertical force sensing and lateral strain sensing separately, which indicates the advantages of DPT with two working modes.

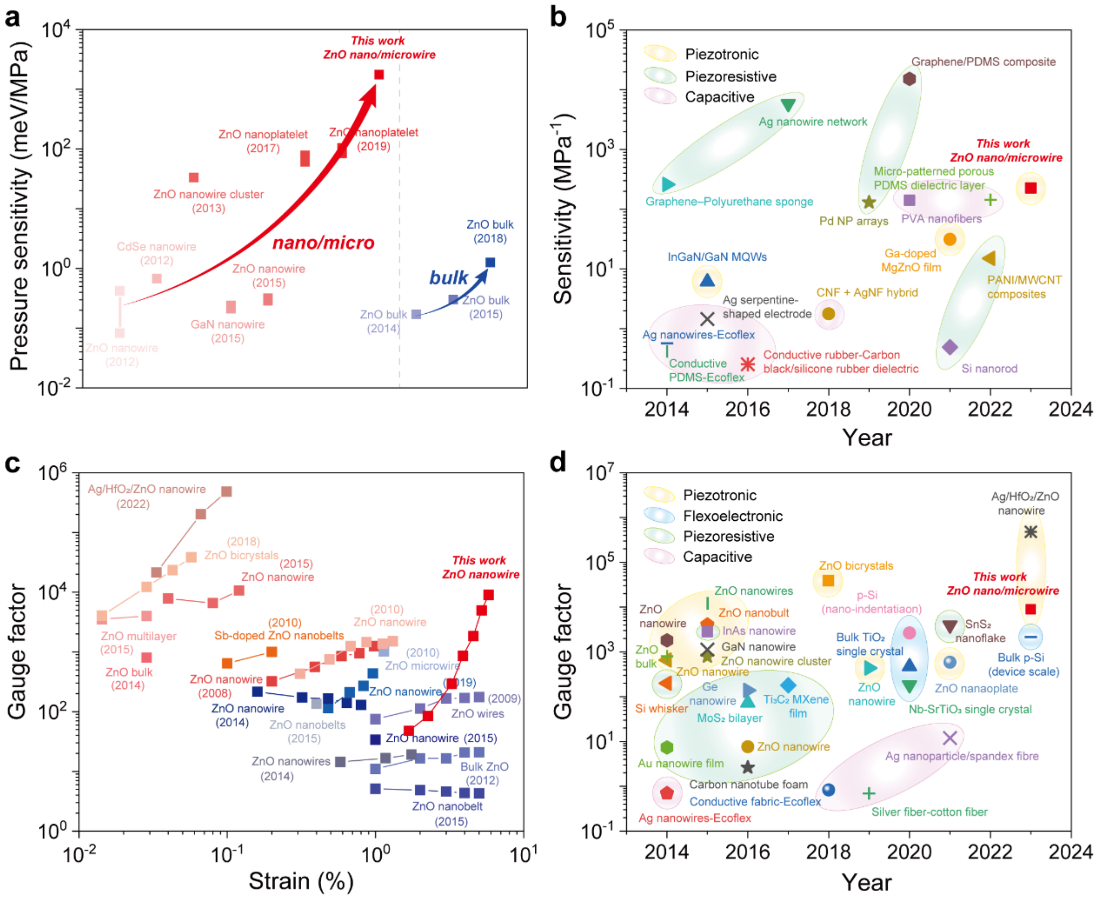

Figure R40. Sensing performance of the DPT. a, Comparison of the pressure sensitivity of DPT with previous piezotronic transistors/sensors in vertical force sensing[2-11]. **b**, Comparison of the pressure sensitivity of DPT in vertical force sensing with some existing piezotronic/ piezoresistive/ capacitive sensors[12-26]. **c**, **d**, Comparison of the gauge factor of DPT with some existing piezotronic/ flexoelectronic/ piezoresistive/ capacitive sensors in strain sensing as a function of the strain (**c**) [7-9, 27-40] and the published year (**d**) [4, 7, 9, 10, 25, 32-34, 39-56].

Question #5

There are inconsistency within the figure caption. Inside the figure it is i,ii, but the main text is I, II.

Response:

We highly appreciate for the reviewer's kindly reminding about the error in Figure 1. We have carefully checked the whole manuscript and revised all similar errors. Now, all the labels like "(ii)", "(iii)" in figures have been replaced by "(II)", "(III)". For

example, Figure 1 in the revised manuscript is changed as the following **Figure R41**.

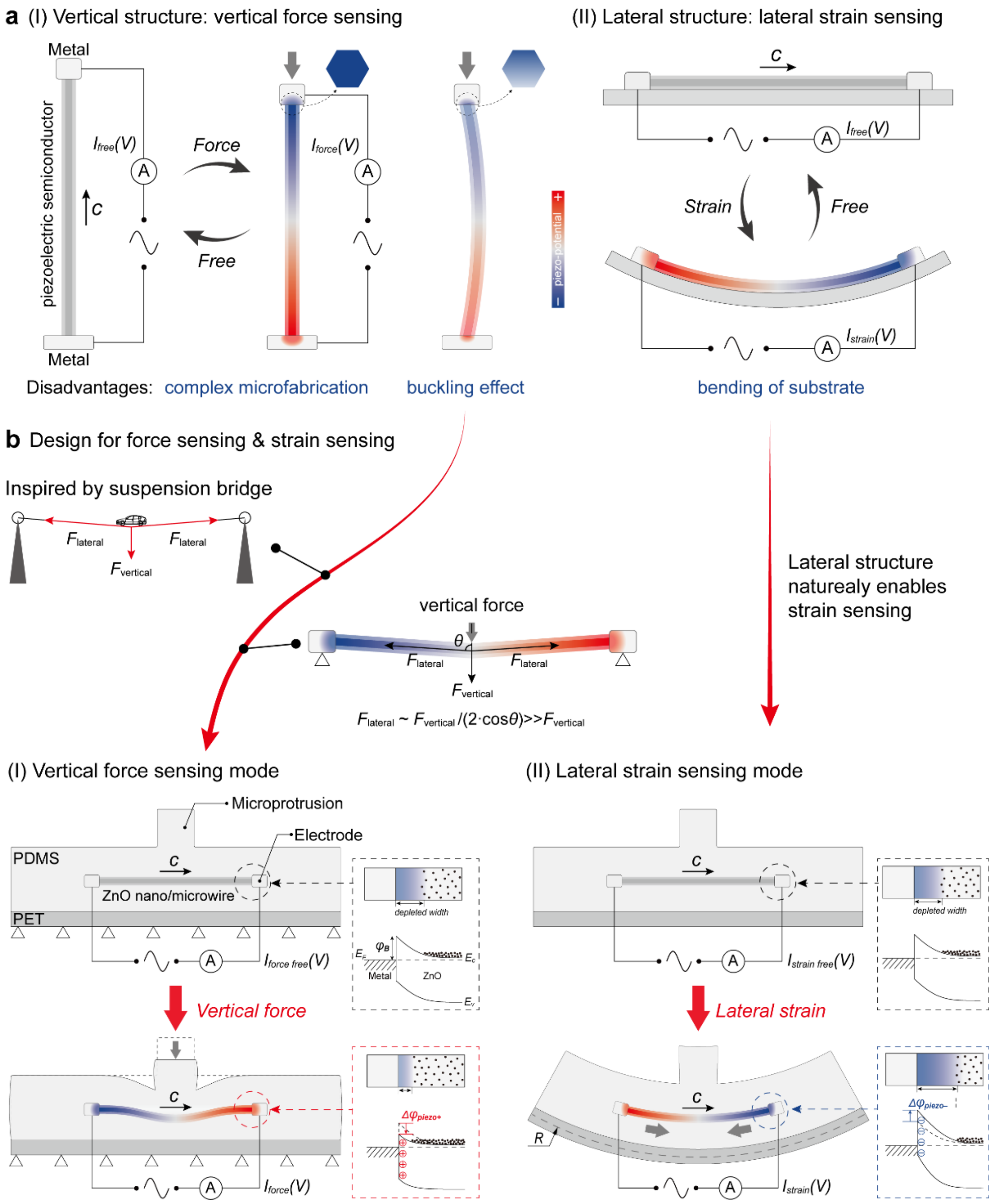

Figure R41. The new Figure 1 in the revised manuscript.

Question #6

In the Note 4, the authors should describe the simulation parameter of PET, such as the modulus and the Poisson ratio.

Response:

We thank the reviewer very much for this constructive suggestion. The detailed

simulation parameters of PET are really what we should provide. Here, we list the main parameters of the material properties for the mechanical simulation using the material library database in the software of COMSOL Multiphysics (**Table R2**). And we have added this information into the supplementary information and marked them in red.

Additionally, we also briefly studied the impact of the PET on the nano/microwire deformation in the vertical force sensing mode of DPT. Note that PET has much more stiffness ($E = 4\ GPa$) than its soft counterparts (such as PDMS, $E = 750\ kPa$), and thus the PET substrate is hard to deform under vertical force condition. To further investigate the effect of the PET substrate, the DPTs with and without the PET substrate are simulated to compare the mechanical properties in vertical force sensing mode. As demonstrated by the simulation results in **Figure R42**, with almost identical nano/microwire strain for the DPTs with and without PET substrate, the addition of the PET substrate has little effect on the sensitivity of the DPT in response to the vertical force.

Table R2. Material parameters of the mechanical properties for PET, PDMS and ZnO in the finite element method (FEM) simulations.

Materials	Young's modulus, E	Poisson's ratio, ν	Reference
PET	4 GPa	0.35	**[57]**
PDMS	750 kPa	0.49	**[58]**
ZnO	210 GPa	0.33	**[59]**

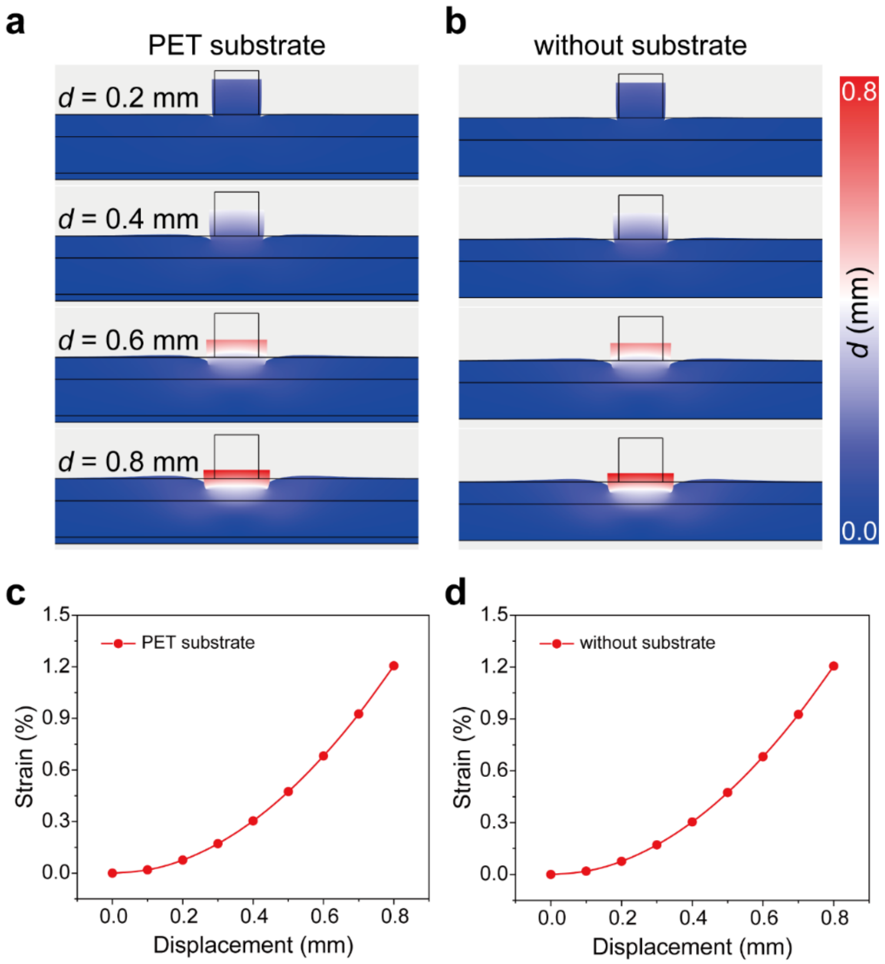

Figure R42. Impact of the PET substrate on the DPT performance in vertical force sensing mode. a, **b**, Displacement distribution profiles of DPTs with (**a**) and without PET substrate (**b**) in response to different displacements (*d*) obtained from the FEM simulation. **c**, **d**, The corresponding nano/microwire strain as a function of the displacement in DPTs with (**c**) and without (**d**) PET substrate, respectively.

Question #7

In the simulation, the author should also simulate the thickness of PDMS. The author should study the following two scenarios. First, the whole PDMS thickness is fixed, the effect of ratio of the top/bottom PDMS layer thickness on the ZnO strain should be studied. Second, once the bottom PDMS layer thickness is fixed, the effect of the top layer PDMS thickness on the ZnO strain should be studied.

Response:

We thank the reviewer very much for the valuable suggestions, which in fact can

improve our understandings about the role of PDMS structure on the dual-modal piezotronic transistor (DPT). According to the structural design of the DPT, the thickness of PDMS layer will directly affect the sensitivity to the vertical stimulus on the bending deformation of the nano/microwire, which also plays a crucial role in improving the sensing performance. In order to delve into the PDMS thickness effect in the DPT for vertical force sensing, the FEM simulations have been performed with a series of controlled experiments as the reviewer suggested and with an additional scenario (Condition: the thickness of the top PDMS layer is fixed, and the thickness of the bottom PDMS layer changes.).

(1) First, the whole PDMS thickness is fixed, the effect of ratio of the top/bottom PDMS layer thickness on the ZnO strain should be studied.

Response: Thanks for this constructive suggestion provided by the reviewer. The FEM model of the DPT with various thicknesses of the top PDMS layers ($h_{PDMS,top}$) ranging from 0.1 mm to 1.0 mm are constructed (**Figure R43a**), while keeping the total PDMS thickness fixed ($h_{PDMS} = 1.325\ mm$). Meanwhile, other structural parameters are set as constant values in the manuscript, including the dimensions of the ZnO nano/microwire ($L_{NW} = 1\ mm$ in length), the microprotrusion ($1\ mm \times 1\ mm$ in height × width) and the PET substrate ($0.15\ mm \times 30\ mm$ in thickness × length).

With application of the displacement boundary conditions (d) on the top edge of the microprotrusion, the side views of the DPTs in the initial state and the vertical force sensing mode are schematically shown in **Figure R43a**. As demonstrated by the FEM simulation results in **Figure R43b**, it is evident that the PDMS thickness ratio of the top layer to the total ($h_{PDMS,top}/h_{PDMS}$) would not affect the overall mechanical property and the corresponding displacement distribution of the DPT, which could be attributed to the identical thickness of the total PDMS layer. Nevertheless, due to the influence of the position of the nano/microwire, the DPTs with various thickness ratios of the PDMS layers ($h_{PDMS,top}/h_{PDMS}$) will induce different magnitudes of bending deformation in the nano/microwires, which continuously decline with the increase of thickness ratio, as shown in **Figure R43c**. It can be also seen clearly from the calculated

strain curves of the nano/microwires in **Figure R43d** and **R43e** that, under the application of a certain displacement ($d = 0.8\,mm$), the nano/microwire strain will decrease from 3.63% at the thickness ratio of 0.08 down to only 0.05% at the ratio of 0.05, which is drastically reduced by ~98.5%. Therefore, it is expected to induce much higher strain level in the nano/microwire with the relatively small thickness ratio ($h_{PDMS,top}/h_{PDMS}$), when the position of the nano/microwire is closer to the local region surrounding the deformed microprotrusion (**Figure R43b**).

(2) Second, once the bottom PDMS layer thickness is fixed, the effect of the top layer PDMS thickness on the ZnO strain should be studied.

Response: We thank the reviewer for this valuable suggestion, which can promote us to understand the impact of the top PDMS layer on the sensing performance. The DPT with a constant thickness of the bottom PDMS layer ($h_{PDMS,bottom} = 0.825\,mm$) is applied in the FEM simulations, while the thickness of the top PDMS layer ($h_{PDMS,top}$) is variable from 0.15 mm to 1 mm (**Figure R44**). **Figure R44b** shows the displacement distribution profiles obtained under different downward displacements (d), which indicates the device deformation with the top PDMS layers at the thicknesses ($h_{PDMS,top}$) of 0.15 mm, 0.50 mm and 1.00 mm, respectively. It can be found that a thinner encapsulation layer (i.e., the top PDMS layer) leads to a diminution of the distance between the nano/microwire and the microprotrusion, and hence induces a larger bending deformation of the nano/microwire under vertical force condition, as shown in **Figure R44c**.

The calculated nano/microwire strain as a function of the downward displacement (d) in the DPTs with different thicknesses of the top PDMS layers ($h_{PDMS,top}$) is shown in **Figure R44d**. And the dependence of the nano/microwire strain with respect to the thickness of the top PDMS layers ($h_{PDMS,top}$) is also shown in **Figure R44e**, in which we can find that when a 0.15-mm-thick PDMS layer is employed for encapsulation, the maximum strain level of the nano/microwire under a given downward displacement d of 0.8 mm can reach about 3.0%, which then begins to drop dominantly with increasing thickness of the top PDMS layer. On the other hand, the top PDMS layer is introduced

as encapsulation for insulation and protection, which should achieve a robust thickness in order to maintain the physical stability of the ZnO nano/microwire. Therefore, utilizing the PDMS layer with appropriate thickness can effectively control the force transfer process to induce the sufficiently large deformation in the nano/microwire, while maintaining a high sensitivity and excellent reliability of the DPT for vertical force sensing.

(3) An additional scenario: as the top PDMS layer thickness is fixed, the effect of the bottom PDMS layer thickness on the ZnO strain is also studied.

As shown in **Figure R45b** and **R45c**, the displacement distribution profiles of DPTs and the corresponding outlines of bended nano/microwires are simulated in response to different displacements (d) applied on the DPTs with a constant top PDMS layer thickness ($h_{PDMS,top} = 0.5\ mm$) and with various of bottom PDMS layer thicknesses ($h_{PDMS,bottom}$) ranged from 0.225 mm to 1.625 mm. It can be obviously seen in **Figure R45c** that the magnitude of the overall displacement of the nano/microwire in the vertical direction gradually increases with thicker bottom PDMS layer. Also, it can be found that the downward bending degree of the nano/microwire increases as the bottom PDMS thickness ($h_{PDMS,bottom}$) increases. As expected, the tendency of decrease in the bending deformation and the overall displacement of the nano/microwire with respect to the reduced thickness of the bottom PDMS layer ($h_{PDMS,bottom}$) should be attributed to the enhanced substrate clamping effect in the thinner PDMS layer. The obtained curves of nano/microwire strain versus downward displacement (d) with the thickness in the range of 0.225 mm to 1.625 mm are plotted in **Figure R45d**, which also indicates that the strain level of the nano/microwire increases with the bottom PDMS thickness ($h_{PDMS,bottom}$). Under the given displacement ($d = 0.8\ mm$), the nano/microwire strain increases approximately linearly and then tends to a saturated value (~1.6%) when the thickness of the bottom PDMS layer ($h_{PDMS,bottom}$) exceeds 1.225 mm, as shown in **Figure R45e**. It should be noted that although the strain increases with the increase of the bottom PDMS thickness ($h_{PDMS,bottom}$), the thickness should not be too large considering the practical application of the device.

Apart from above effect of the PDMS layer on the device performance, a very important function of the top and bottom PDMS layers together is actually to protect the lateral nano/microwire from breaking when subjected to the vertical force. Our earliest device design is based on the structure of suspended nano/microwires just like the one in **Figure R38c**, but through a large number of experiments, it is found that the lack of top and bottom PDMS layers will cause the local stress of the device to be too large and very easy to damage.

We thank again for the reviewer's very valuable suggestions. And we have added the above discussions into the revised Supplementary Information and marked them in red.

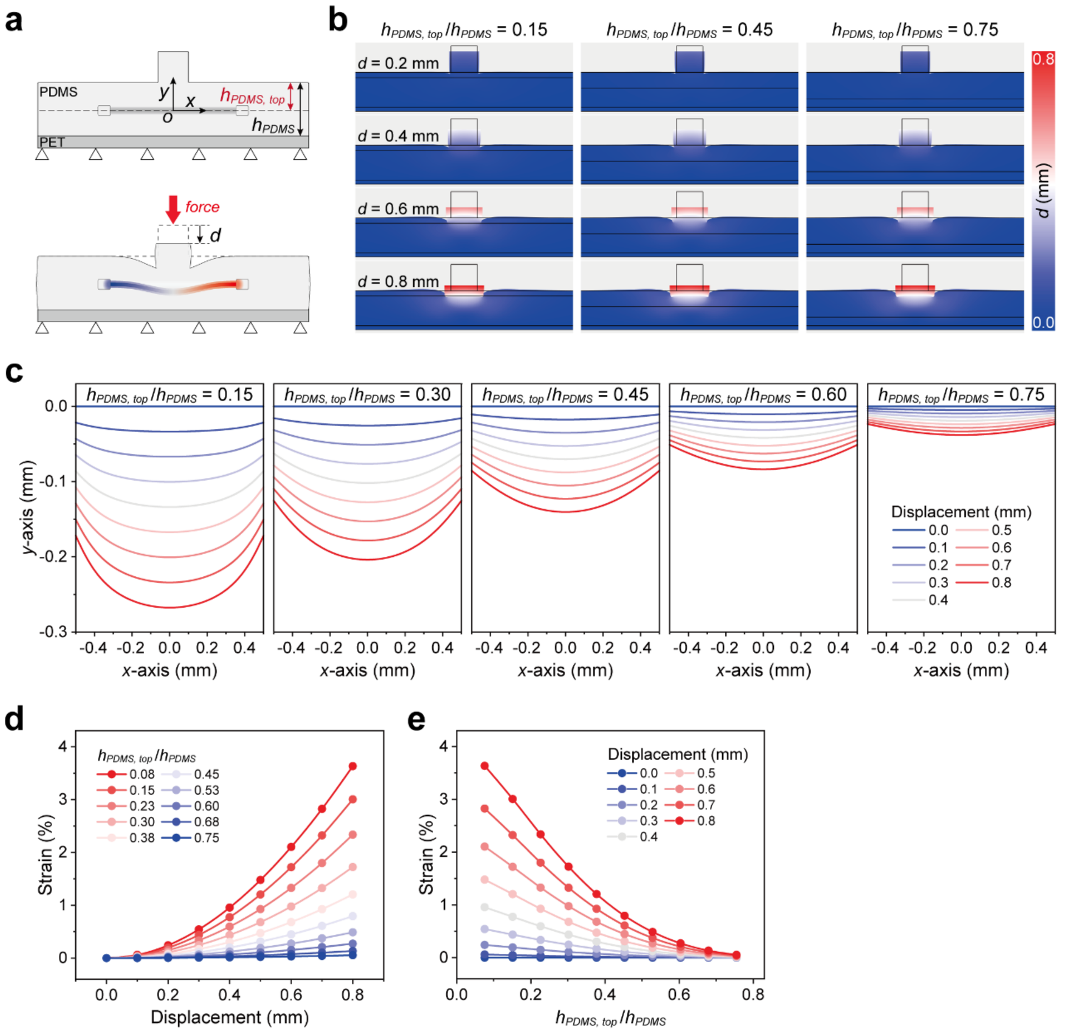

Figure R43. Impact of the PDMS thickness ratio of the top layer to the total ($h_{PDMS,top}/h_{PDMS}$) on the DPT performance for vertical force sensing by FEM

simulations. **a**, Schematic illustration of the DPT with the detailed structure of two PDMS layers in vertical force sensing mode. $h_{PDMS,top}$ and h_{PDMS} represent the thicknesses of the top and the total PDMS layer, respectively. **b**, **c** Displacement distribution profiles of the DPTs (**b**) and the corresponding outlines of bended nano/microwires (**c**) with various $h_{PDMS,top}/h_{PDMS}$ in response to different displacements (d). **d**, The calculated strain of nano/microwires of the DPTs with different $h_{PDMS,top}/h_{PDMS}$ as a function of d. **e**, The curves of nano/microwire strain versus $h_{PDMS,top}/h_{PDMS}$ under different d.

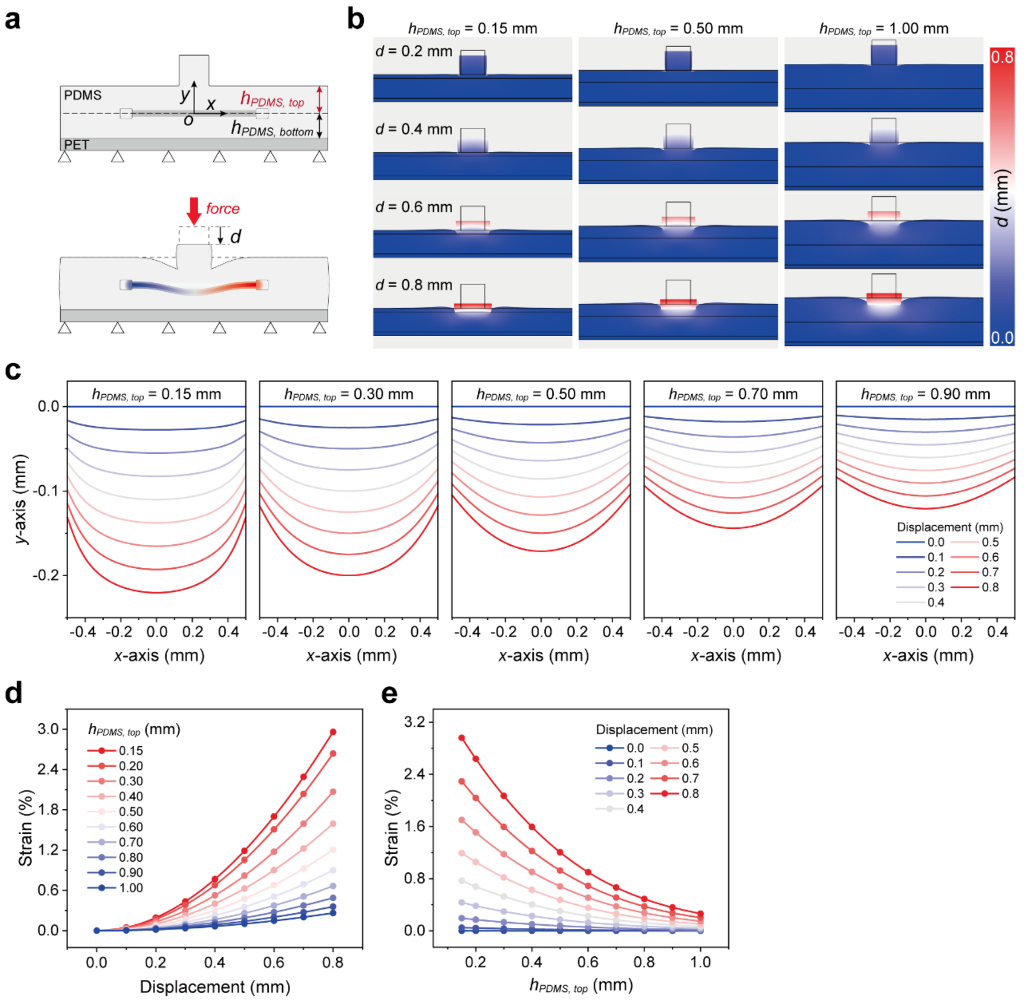

Figure R44. Impact of the top PDMS layer thickness ($h_{PDMS,top}$) on the DPT performance for vertical force sensing by FEM simulations. **a**, Schematic illustration of the DPT with the detailed structure of two PDMS layers in vertical force sensing mode. $h_{PDMS,top}$ and $h_{PDMS,bottom}$ represent the thicknesses of the top and the bottom PDMS layer, respectively. **b**, **c**, Displacement distribution profiles of the

DPTs (**b**) and the corresponding outlines of bended nano/microwires (**c**) with various $h_{PDMS,top}$ in response to different displacements (d). **d**, The calculated strain of nano/microwires of the DPTs with different $h_{PDMS,top}$ as a function of d. **e**, The curves of nano/microwire strain versus $h_{PDMS,top}$ under different displacements d.

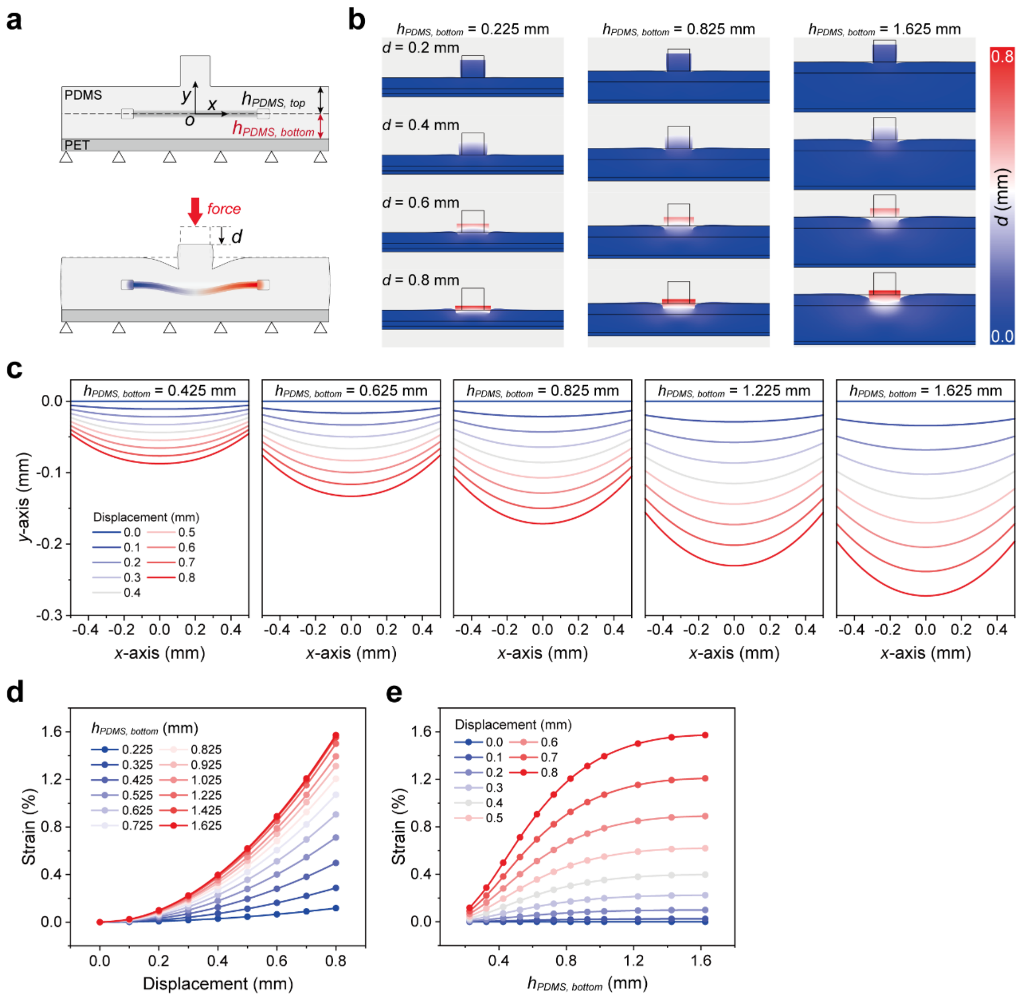

Figure R45. Impact of the bottom PDMS layer thickness ($h_{PDMS,bottom}$) on the DPT performance for vertical force sensing by FEM simulations. a, Schematic illustration of DPT with detailed structure of two PDMS layers in vertical force sensing mode. $h_{PDMS,top}$ and $h_{PDMS,bottom}$ represent the thicknesses of the top PDMS layer and the bottom PDMS layer, respectively. **b**, **c** Displacement distribution profiles of the DPTs (**b**) and the corresponding outlines of bended nano/microwires (**c**) with various $h_{PDMS,bottom}$ in response to different downward displacements (d). **d**, The calculated strain of nano/microwires of the DPTs with different $h_{PDMS,bottom}$ as a function of d. **e**, The curves of nano/microwire strain versus $h_{PDMS,bottom}$ under different d.

Question #8

In Fig. 3f, the sensitivity is 1.759eV/N, but the corresponding text in manuscript is 1.759eV/MPa, is this a typo or derived? If 1N corresponds to 1MPa, it means that the cross-sectional area of the microstructure protrusion is 1mm2, which is inconsistent with the ratio of 0.66 obtained by the author's optimization.

Response:

We thank the reviewer for this kindly reminder. We are glad to answer these two questions one by one as follows.

(1) In Fig. 3f, the sensitivity is 1.759eV/N, but the corresponding text in manuscript is 1.759eV/MPa, is this a typo or derived?

Response: We thank the reviewer for giving us this detailed comment. The pressure sensitivity of 1.759 eV/MPa is a derived value from the sensitivity of 1.759 eV/N. In the experiments, to apply a controlled force or downward displacement (d) on the top surface of the microprotrusion in DPT for vertical force sensing, a linear actuator together with a commercial force sensor is vertically pressed on the top surface of the microprotrusion in DPT, which could precisely control d and simultaneously measure the applied force F. The dimensions of the DPT device are shown in **Figure R46a**, where the microprotrusion is manufactured by casting a PDMS pillar with a cross-sectional area S of 1×1 mm^2. With this configuration, the relation between the force F and the corresponding pressure P applied onto the contact area S (or the top surface of the microprotrusion) can be calculated by the formula $P = F/S$, which means 1 N is equivalent to 1 MPa.

(2) If 1N corresponds to 1MPa, it means that the cross-sectional area of the microstructure protrusion is 1mm2, which is inconsistent with the ratio of 0.66 obtained by the author's optimization.

Response: We thank the reviewer for this comment. It is really a nice question! In fact, in our earliest design, in order to achieve the microprotrusion structure ratio w/L_{NW} of 0.66, we matched nano/microwires by manufacturing microprotrusions with different sizes. However, since the microprotrusion is made of PDMS and its size is small (in about 1 millimeter), the error introduced by manufacturing microprotrusions

was relatively large. Later, in order to solve this problem, we found that by controlling the size of the mask to accurately control the electrode spacing (space between source and drain electrodes, deposited by RF-magnetron sputtering on the ZnO nano/microwire) is a more feasible method that possess a higher accuracy. Therefore, we fabricated a PDMS microprotrusion with a cross-sectional area of 1 mm × 1 mm, and then controlled the electrode spacing to achieve various microprotrusion structure ratios w/L_{NW}. **Figure R46b** shows the DPTs with various channel lengths on the ZnO nano/microwires and the corresponding structure ratios w/L_{NW}, through adjusting the accurate distance of the source/drain electrodes. Note that when the electrode distance of the ZnO nano/microwire (the channel length) is 1.5 mm, the microprotrusion structure ratio w/L_{NW} of the DPT could exactly be the optimal value 0.66, which is thus adopted as the practical parameter in the experimental section.

The above discussions can improve the readability of the manuscript and the repeatability of this work. So, we have added the above discussions into the revised Supplementary Information and marked them in red.

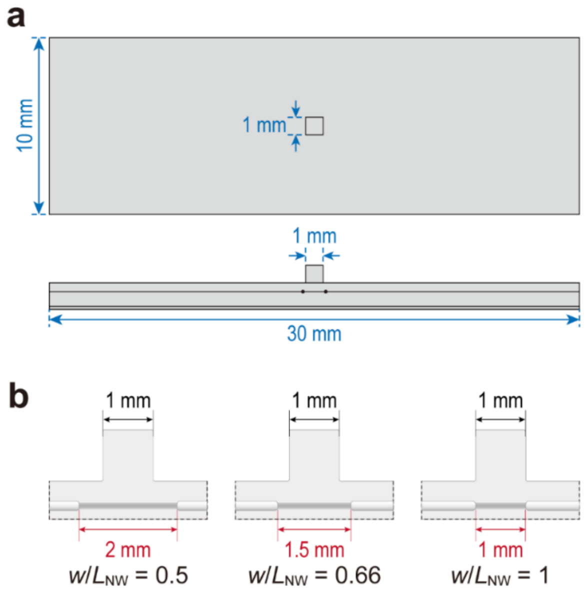

Figure R46. Dimension of DPTs and method to achieve the structure ratio of 0.66. **a**, Overview of the DPT with the top view (top) and the side view (bottom). **b**, Schematic illustration of the DPTs with various channel lengths on the ZnO nano/microwires and the corresponding structure ratios w/L_{NW}.

Question #9

In the model of Note 8 and Fig. S8, the author believes that the Young's modulus of PDMS is smaller than that of PET. Thus, PMDS can deform with the bending of PET, and it is assumed that this deformation is close to a circular arc. In fact, since the Poisson's ratio of PDMS is close to 0.5, that is, its volume compressibility is small, so when PET is deformed, will PDMS compress strictly according to the shape of PET? Besides, is the deformation of ZnO along the direction of the arc? These issues require a more detailed proof.

Response:

We appreciate the reviewer for this comment and kindly reminder. Under the suggestion provided by the reviewer, we carefully checked our experiments and found that the shape of PET is not an ideal circular arc, indeed as the reviewer speculated. Under a displacement ΔL (**Figure R47a**), the shape of the PET as well as the ZnO nano/microwire cannot remain a circular arc, due to the small volume compressibility of PDMS. So, our original strain calculation is an approximate method, which has a big error. In order to accurately calibrate the strain applied to the nano/microwires, we redesigned the driving mode of the strain, and applied FEM simulation to obtain the strain with consideration of the PDMS Poisson's ratio of 0.49.

As shown in **Figure R47b**, we fix the DPT onto the inner surface of a steel coil, and then apply strain to the DPT by changing the diameter of the steel coil. In this case, we can confirm that the shape of the PET is a circular arc. By measuring the distance change ΔL between the two ends of the PET or the radius of the steel coil in this case, combined with the FEM simulation, we can get a relatively accurate strain. It should be noted that the boundary condition of PET is a circular arc in the FEM simulation. **Figure R47c** shows the comparison between the new method (using steel coil and FEM simulation) and the original method (using formula in **Figure R47a**). As can be seen that a larger strain will be produced when the PET is constrained as a circular arc.

In order to ensure the accuracy of DPT characterization, we re-fabricated new DPT devices, and used the method in **Figure R47b** to drive the DPT to strain, and adopted the newly calibrated strain. **Figure 4** and **Figure 5** have been both updated by new

experiments in the revised manuscript.

Although the novelty of this work mainly lies on the vertical force sensing (**response to Question #3**), we are very grateful to the reviewer and thank the reviewer for this very important comment to help us optimize the experiment to avoid errors.

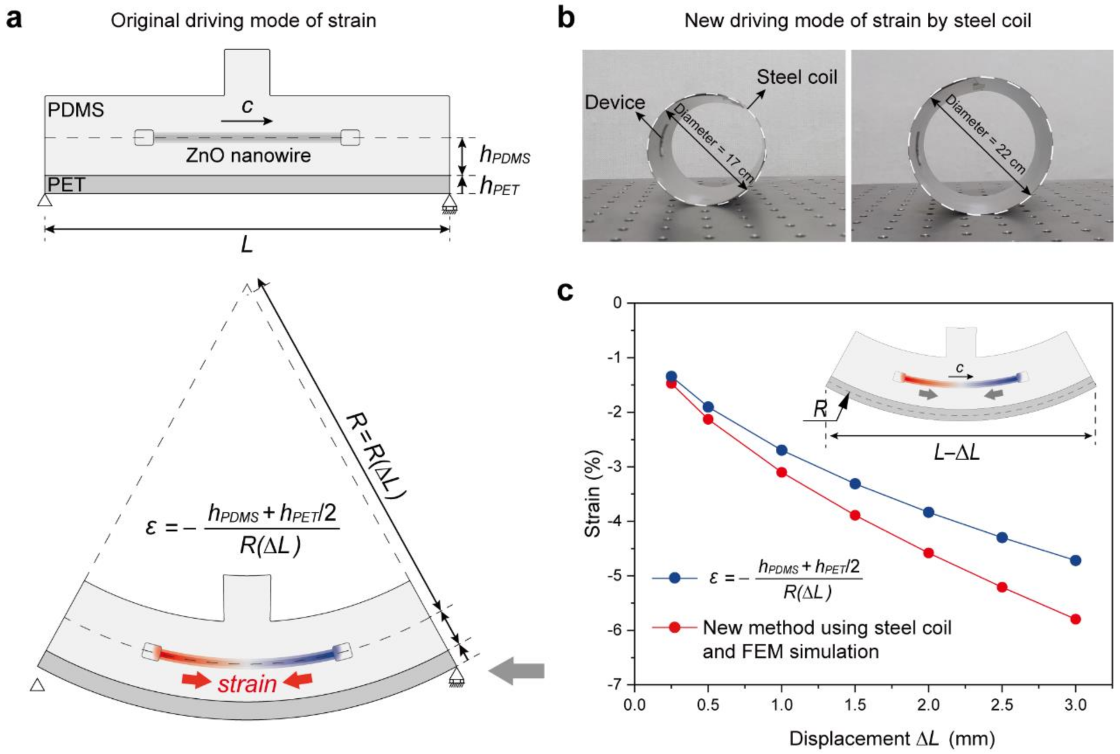

Figure R47. Methods to calculate the applied strain. a, Original driving mode of strain on DPT and the method to calculate the strain by the theory formula. **b**, New driving mode of strain by steel coil. Through controlling the radius of the steel coil to control the strain applied on the DPT. In this new driving mode, the PET substrate can be constrained as a circle arc. **c**, Comparison between the new method (using steel coil (**b**) and FEM simulation) and the original method (using formula (**a**)).

Question #10

In Fig. 4d-e, why use resistance ratio when characterizing DPT forward bias characteristics, and use current ratio when characterizing reverse bias characteristics, why not both use current ratio (similar to characterization of vertical force sensing)

Response:

We thank very much for the reviewer's valuable and constructive comment/suggestion. Actually, the reason why we use resistance ratio and current ratio respectively under forward bias and reverse bias to characterize DPT is due to the strain-induced **asymmetric** modulation of electrical transport by piezotronic effect. In order to reveal the reason more clearly, here we will discuss (1) different regulation mechanisms of traditional piezoresistive effect and piezotronic effect, and (2) definition of change ratio in asymmetric strain-controlled piezotronic devices, and (3) examples for demonstration.

(1) Different regulation mechanisms of traditional piezoresistive effect and piezotronic effect

Both resistance ratio and current ratio are widely used parameters to characterize the performance of sensors, especially in traditional piezoresistive materials or devices. Usually, we use resistance ratio to characterize the device possessing increased resistance with applied strain, while use current ratio to characterize the device possessing increased current with applied strain. However, it is rare that both are used at the same time. With the emerging of strain-controlled devices in new principles (*e.g.* piezotronic effect), the applicable scenarios of resistance ratio and current ratio have also been updated.

① *Strain-induced* ***symmetric*** *modulation of electrical transport by piezoresistive effect*

In piezoresistive effect, as shown in **Figure R48a**, if a tensile strain increases the resistance of the device, no matter the device is forward biased or reverse biased, the forward current and reverse current of the device will both decrease with the tensile strain. As also shown in **Figure R48b**, if a compressive strain decreases the resistance of the device, no matter the device is forward biased or reverse biased, the forward current and reverse current of the device will both increase with the compressive strain. As a result, the electrical transport characteristics of the device is illustrated in **Figure R48c**, indicating that the strain-induced modulation of electrical transport by piezoresistive effect is a symmetric effect. Apart from above case, if a tensile strain decreases the resistance and a compressive strain increases the resistance, the same conclusion will also be obtained, indicating a symmetric strain-induced modulation of

electrical transport by piezoresistive effect. So, it is a strain-induced symmetric modulation of electrical transport by piezoresistive effect.

② *Strain-induced **asymmetric** modulation of electrical transport by piezoelectric charges (piezotronic effect)*

Piezotronic effect is an interface effect that asymmetrically modulates local contacts at different terminals of the device [5], as schematically illustrated in the following **Figure R49**. Schottky junctions will be formed in local contacts at different terminals of the device without strain (**Figure R49a**). Due to the accidental error/uncertainty of device manufacturing [37], the Schottky junctions at both ends will be slightly different.

When subjecting to a tensile strain (**Figure R49b**), positive piezoelectric polarization charges are produced at the surface of the ZnO nano/microwire (assuming its piezoelectric *c*-axis points to the left) on the left contact of device, which will reduce the barrier height of the Schottky junction; while negative polarization charges are produced on the right contact, leading to an increasement of the barrier height of the Schottky junction. When a forward bias (potential of right electrode is higher than that of left electrode, **left** in **Figure R49b**) is applied, the electrical transport characteristics are mainly determined by the Schottky junction on the left terminal, which is reversely biased. Because of the decreased barrier height of the left contact, the current flowing through the device will increase and become larger than that of device without strain. Similarly, when the device is reversely biased (potential of right electrode is lower than that of left electrode, **right** in **Figure R49b**), under a same bias voltage, the (reverse) current of the device will be smaller than that without strain. Therefore, compared to the case without strain, the forward current of the device with tensile strain will increase; while the reverse current will decrease. With the same principle, we can also analyze the modulation of the current of the device under a compressive strain, as shown in **Figure R49c**. We can see that the compressive strain will cause a decrease of the forward current (**left** in **Figure R49c**) and an increase of the reverse current (**right** in **Figure R49c**).

The corresponding *I-V* curves can be schematically plotted in the **Figure R49d**. The strain-induced modulation of electrical transport by piezoelectric charges

(piezotronic effect) is asymmetric. The piezotronic effect modulation arises as a result of the polarization of nonmobile ions in the piezoelectric crystal [60, 61]. Thus, it is a strain-induced asymmetric modulation of electrical transport by piezotronic effect.

Therefore, different from the symmetric modulation by traditional piezoresistive effect, an asymmetric modulation is usually made by piezotronic effect, in which the current increases with strain under one bias, while the current decreases with strain under opposite bias.

(2) Definition of change ratio in asymmetric strain-controlled piezotronic devices

As for piezoresistive effect, if the resistances under forward bias and reverse bias both increases with applied strain, we can use the resistance ratio $\Delta R/R_0$ (in which R_0 is the original resistance, and ΔR is the change in resistance) to characterize the piezoresistive device. However, when we apply resistance ratio $\Delta R/R_0$ to the piezotronic devices shown in **Figure R49c** at the same time, the problem arises. Here, considering the situation upon a compressive strain, the forward current decreases (forward resistance increase) and the reverse current increases (reverse resistance decreases) with strain. As a result, we can get the following two formulas

$$(\frac{\Delta R}{R_0})_{forward} = \frac{R_{\varepsilon,forward} - R_0}{R_0} > 0 \tag{32}$$

$$(\frac{\Delta R}{R_0})_{reverse} = \frac{R_{\varepsilon,reverse} - R_0}{R_0} = \frac{R_{\varepsilon,reverse}}{R_0} - 1 < 0 \tag{33}$$

in which R_ε is the resistance under strain ε.

The better the performance of the piezotronic device upon compressive strain in **Figure R49c**, the larger $R_{\varepsilon,forward}$ and the smaller $R_{\varepsilon,reverse}$, and hence the larger $(\Delta R/R_0)_{forward}$ but the closer $(\Delta R/R_0)_{reverse}$ is to -1! We can find that in this case, the resistance ratio $\Delta R/R_0$ can well characterize the sensing performance of the device under forward bias, but it fails to characterize the performance of the device under reverse bias. At this time, using the resistance ratio $\Delta I/I_0$ (in which I_0 is the original current, and ΔI is the change in current) to characterize the performance of the device under reverse bias is very appropriate. For example, in this case, we can write

$$(\frac{\Delta I}{I_0})_{reverse} = \frac{I_{\varepsilon,reverse} - I_0}{I_0} = \frac{I_{\varepsilon,reverse}}{I_0} - 1$$

(34)

in which I_ε is the resistance under strain ε. The better the performance of the piezotronic device upon compressive strain in **Figure R49c**, the larger $(\Delta R/R_0)_{forward}$ and the larger $(\Delta I/I_0)_{reverse}$.

Based on the above considerations, in order to allow a parameter to reflect the performance of the piezotronic device, we use the resistance ratio $\Delta R/R_0$ to characterize DPT when the resistance increases with the applied strain, which is corresponding to the condition of forward bias; and use the current ratio $\Delta I/I_0$ to characterize DPT when the current increases with the applied strain, which is corresponding to the condition of reverse bias. In fact, we would also use both resistance ratio and current ratio in the vertical force sensing mode, but because of its small reverse current, we only calculated the current ratio under forward bias.

(3) Examples for demonstration

As a demonstration shown in **Figure R50a**, the forward current decreases with the compressive strain, while the reverse current increases with the compressive strain. In this situation, we can plot the current ratio under reverse bias as shown in **Figure R50b**, and plot the resistance ratio under forward bias as shown in **Figure R50d**. As a contrast, if conversely, we will get the resistance ratio under reverse bias in **Figure R50c**, and get the current ratio under forward bias in **Figure R50e**. It can be seen from **Figure R50c** and **R50e** that the better the device performance, the closer the change ratios are to -1, which cannot properly characterize the performance of the devices.

Therefore, due to the strain-induced asymmetric modulation of electrical transport by piezotronic effect, we use resistance ratio when characterizing DPT under forward bias, and use current ratio when characterizing DPT under reverse bias. In order to improve the readability and quality of the manuscript, we have added the above discussions to the supplementary information for researchers' reference.

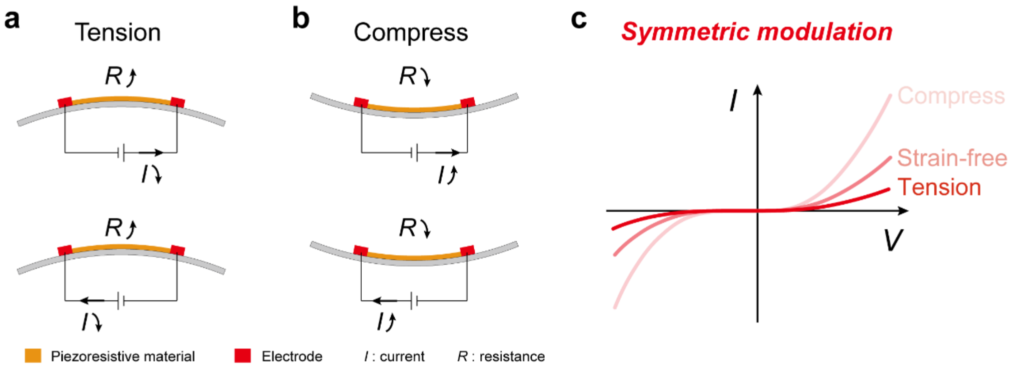

Figure R48. Strain-induced symmetric modulation of electrical transport by piezoresistive effect. a, Tensile strain increases the resistances and decreases the currents both under forward bias and reverse bias. **b**, Compressive strain decreases the resistances and increase the currents both under forward bias and reverse bias. **c**, Schematic illustrations of the *I*-*V* curves of the device under no strain, tensile strain and compressive strain, which indicate a symmetric modulation of electrical transport by strain in piezoresistive effect.

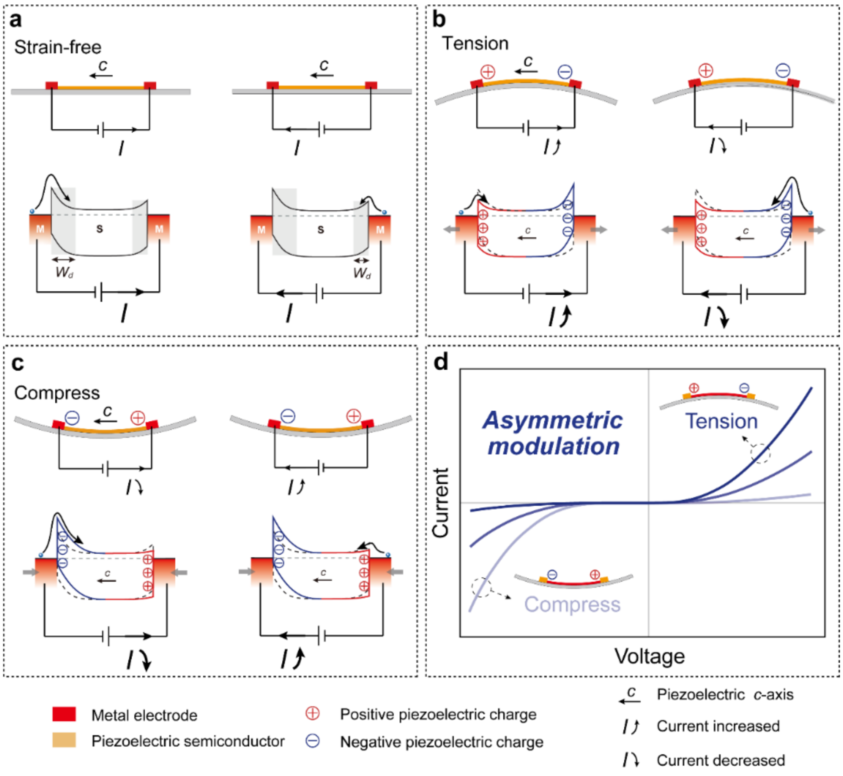

Figure R49. Strain-induced asymmetric modulation of electrical transport by piezoelectric charges (piezotronic effect). a-c, Schematic diagrams of bending situations and corresponding energy bands of device with no strain (**a**), tensile strain (**b**)

and compressive strain (**c**). **d**, Schematic illustrations of the I-V curves of the device under no strain, tensile strain and compressive strain, which indicate an asymmetric modulation of electrical transport by strain in piezotronic effect.

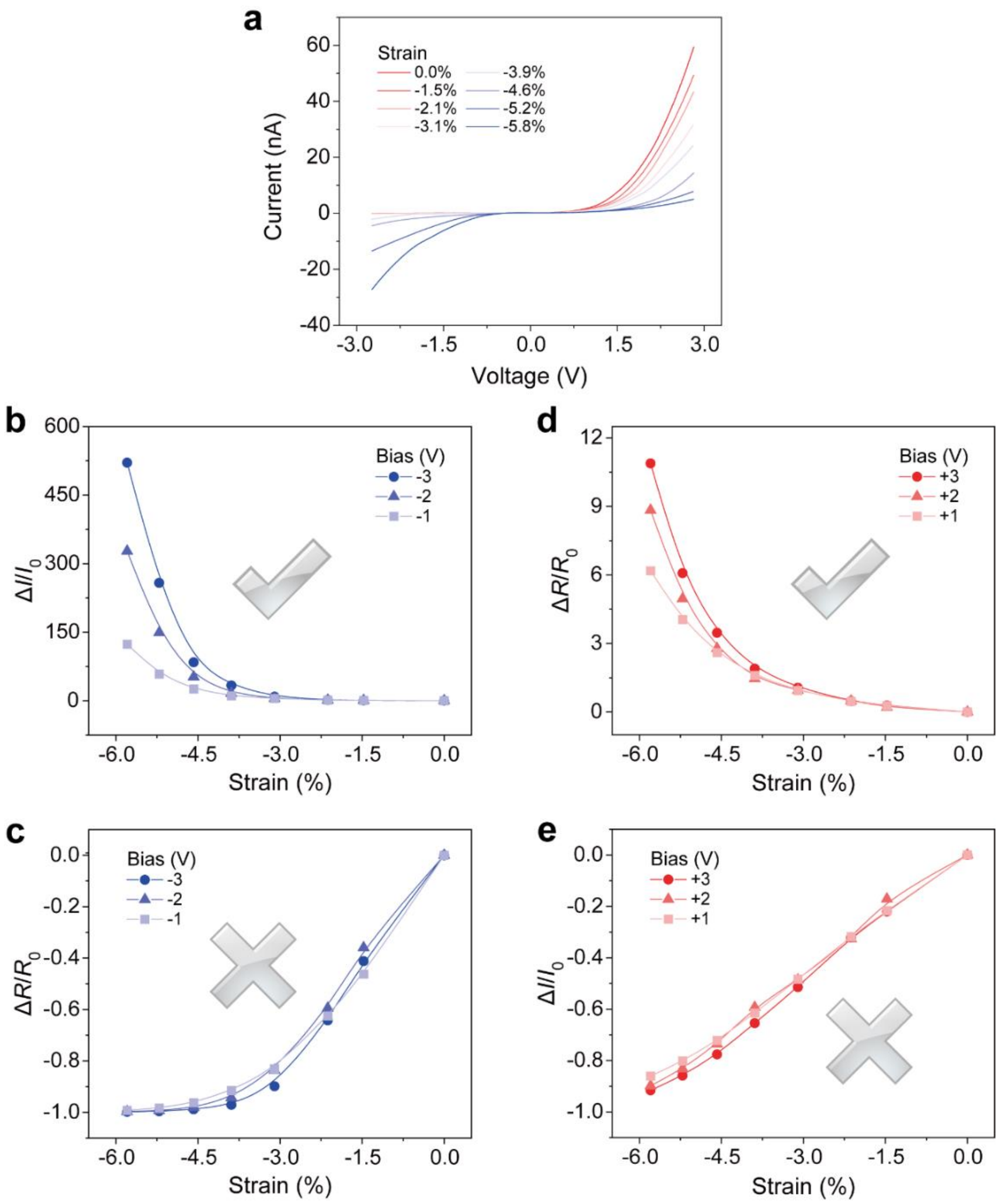

Figure R50. Resistance ratio ($\Delta R/R_0$) and current ratio ($\Delta I/I_0$) used to characterize the piezotronic device under forward bias and reverse bias. **a**, Strain-induced asymmetric modulation of electrical transport by piezotronic effect. **b**, **c**, Current ratio (**b**) and resistance ratio (**c**) as functions of the strain under reverse bias. **d**, **e**, Resistance ratio (**d**) and current ratio (**e**) as functions of the strain under forward bias.

Question #11

In Fig. 5f, the definition of on/off ratio is not given.

Response:

We appreciate for the reviewer's kindly reminder. The on/off ratio is usually adopted to characterize sensors, which reflects the sensing performance of the device in response to the applied strain or force. In **Figure 5f**, the on/off ratio is defined as the ratio of the current I_{on} in "ON" state (corresponding to the current after application of a strain or force) to the current I_{off} in "OFF" state (corresponding to the current before application of a strain or force), which can be written as

$$(on/off\ ratio)_{strain} = I_{\mathrm{on}}/I_{\mathrm{off}} = I_{after\ a\ strain}/I_{before\ a\ strain} \tag{35}$$

$$(on/off\ ratio)_{force} = I_{\mathrm{on}}/I_{\mathrm{off}} = I_{after\ a\ force}/I_{before\ a\ force} \tag{36}$$

To improve the readability and quality of the manuscript, we have added the above discussions to the revised Supplementary Information.

At the same time, in order to avoid misunderstanding, and make the manuscript more rigorous and readable, we have added the definition of on/off ratio in the revised manuscript and marked them in red.

Reference:

1. Lee, J. H. *et al.* Heterogeneous structure omnidirectional strain sensor arrays with cognitively learned neural networks. *Advanced Materials* **35**, 2208184 (2023).
2. Zhou, Y. S. *et al.* Vertically aligned CdSe nanowire arrays for energy harvesting and piezotronic devices. *ACS Nano* **6**, 6478-6482 (2012).
3. Han, W. *et al.* Strain-gated piezotronic transistors based on vertical zinc oxide nanowires. *ACS Nano* **6**, 3760-3766 (2012).
4. Zhao, Z. *et al.* Piezotronic effect in polarity-controlled GaN nanowires. *ACS Nano* **9**, 8578-8583 (2015).
5. Wu, W., Wen, X. & Wang, Z. L. Taxel-addressable matrix of vertical-nanowire

piezotronic transistors for active and adaptive tactile imaging. *Science* **340**, 952-957 (2013).

6. Liu, S. *et al.* Ultrasensitive 2D ZnO piezotronic transistor array for high resolution tactile imaging. *Advanced Materials* **29**, 1606346 (2017).
7. Baraki, R., Novak, N., Frömling, T., Granzow, T. & Rödel, J. Bulk ZnO as piezotronic pressure sensor. *Applied Physics Letters* **105**, 111604 (2014).
8. Raidl, N., Supancic, P., Danzer, R. & Hofstätter, M. Piezotronically modified double Schottky barriers in ZnO varistors. *Advanced Materials* **27**, 2031-2035 (2015).
9. Keil, P., Trapp, M., Novak, N., Frömling, T., Kleebe, H.-J. & Rödel, J. Piezotronic tuning of potential barriers in ZnO bicrystals. *Advanced Materials* **30**, 1705573 (2018).
10. Han, X., Du, W., Yu, R., Pan, C. & Wang, Z. L. Piezo-phototronic enhanced UV sensing based on a nanowire photodetector array. *Advanced Materials* **27**, 7963-7969 (2015).
11. Liu, S. *et al.* Double-channel piezotronic transistors for highly sensitive pressure sensing. *ACS Nano* **12**, 1732-1738 (2018).
12. Lee, P. H., Brahma, S., Dutta, J., Huang, J.-L. & Liu, C.-P. Synergistic effects of Ga doping and Mg alloying over the enhancement of the stress sensitivity of a Ga-doped MgZnO pressure sensor. *Nanoscale Advances* **3**, 3909-3917 (2021).
13. Peng, M. *et al.* High-resolution dynamic pressure sensor array based on piezo-phototronic effect tuned photoluminescence imaging. *ACS Nano* **9**, 3143-3150 (2015).
14. Wei, Y., Chen, S., Dong, X., Lin, Y. & Liu, L. Flexible piezoresistive sensors based on “dynamic bridging effect” of silver nanowires toward graphene. *Carbon* **113**, 395-403 (2017).
15. Ghosh, R. *et al.* Fabrication of piezoresistive Si nanorod-based pressure sensor arrays: a promising candidate for portable breath monitoring devices. *Nano Energy* **80**, 105537 (2021).
16. Arias-Ferreiro, G. *et al.* Flexible 3D printed acrylic composites based on

polyaniline/multiwalled carbon nanotubes for piezoresistive pressure sensors. *Advanced Electronic Materials* **8**, 2200590 (2022).

17. Pan, L. *et al.* An ultra-sensitive resistive pressure sensor based on hollow-sphere microstructure induced elasticity in conducting polymer film. *Nature Communications* **5**, 3002 (2014).
18. Wang, Y., Wu, H., Xu, L., Zhang, H., Yang, Y. & Wang, Z. L. Hierarchically patterned self-powered sensors for multifunctional tactile sensing. *Science Advances* **6**, eabb9083 (2020).
19. Chen, M. *et al.* An ultrahigh resolution pressure sensor based on percolative metal nanoparticle arrays. *Nature Communications* **10**, 4024 (2019).
20. Lee, S. *et al.* Nanomesh pressure sensor for monitoring finger manipulation without sensory interference. *Science* **370**, 966-970 (2020).
21. Wang, J., Li, L., Zhang, L., Zhang, P. & Pu, X. Flexible capacitive pressure sensors with micro-patterned porous dielectric layer for wearable electronics. *Journal of Micromechanics and Microengineering* **32**, 034003 (2022).
22. An, B. W., Heo, S., Ji, S., Bien, F. & Park, J.-U. Transparent and flexible fingerprint sensor array with multiplexed detection of tactile pressure and skin temperature. *Nature Communications* **9**, 2458 (2018).
23. Zhao, X., Hua, Q., Yu, R., Zhang, Y. & Pan, C. Flexible, stretchable and wearable multifunctional sensor array as artificial electronic skin for static and dynamic strain mapping. *Advanced Electronic Materials* **1**, 1500142 (2015).
24. Guo, X., Huang, Y., Cai, X., Liu, C. & Liu, P. Capacitive wearable tactile sensor based on smart textile substrate with carbon black /silicone rubber composite dielectric. *Measurement Science and Technology* **27**, 045105 (2016).
25. Yao, S. & Zhu, Y. Wearable multifunctional sensors using printed stretchable conductors made of silver nanowires. *Nanoscale* **6**, 2345-2352 (2014).
26. Woo, S.-J., Kong, J.-H., Kim, D.-G. & Kim, J.-M. A thin all-elastomeric capacitive pressure sensor array based on micro-contact printed elastic conductors. *Journal of Materials Chemistry C* **2**, 4415-4422 (2014).
27. Sun, X., Gu, Y., Wang, X., Zhang, Z. & Zhang, Y. Strain-modulated transport

properties of Cu/ZnO-nanobelt/Cu nanojunctions. *physica status solidi (b)* **252**, 1767-1772 (2015).

28. Zhang, G., Luo, X., Zheng, Y. & Wang, B. Giant piezoelectric resistance effect of nanoscale zinc oxide tunnel junctions: first principles simulations. *Physical Chemistry Chemical Physics* **14**, 7051-7058 (2012).
29. Shao, R. W. *et al.* Bandgap engineering and manipulating electronic and optical properties of ZnO nanowires by uniaxial strain. *Nanoscale* **6**, 4936-4941 (2014).
30. Nakamura, K. First-principles simulation on wire diameter dependence of piezoresistivity in zinc oxide nanowires. *Japanese Journal of Applied Physics* **54**, 06FJ11 (2015).
31. Yang, Y., Qi, J. J., Gu, Y. S., Wang, X. Q. & Zhang, Y. Piezotronic strain sensor based on single bridged ZnO wires. *physica status solidi (RRL) – Rapid Research Letters* **3**, 269-271 (2009).
32. Yang, X. *et al.* Coupled ion-gel channel-width gating and piezotronic interface gating in ZnO nanowire devices. *Advanced Functional Materials* **29**, 1807837 (2019).
33. Zhang, Z. *et al.* Highly efficient piezotronic strain sensors with symmetrical Schottky contacts on the monopolar surface of ZnO nanobelts. *Nanoscale* **7**, 1796-1801 (2015).
34. Wang, P. *et al.* Asymmetric behavior in flexible piezoelectric strain sensors made of single ZnO nanowires. *Journal of nanoscience and nanotechnology* **14**, 6084-6088 (2014).
35. Sun, K. *et al.* Growth of ultralong ZnO microwire and its application in isolatable and flexible piezoelectric strain sensor. *physica status solidi (a)* **207**, 488-492 (2010).
36. Wu, W., Wei, Y. & Wang, Z. L. Strain-gated piezotronic logic nanodevices. *Advanced Materials* **22**, 4711-4715 (2010).
37. Zhou, J. *et al.* Flexible piezotronic strain sensor. *Nano Letters* **8**, 3035-3040 (2008).
38. Yang, Y., Guo, W., Qi, J. & Zhang, Y. Flexible piezoresistive strain sensor based on single Sb-doped ZnO nanobelts. *Applied Physics Letters* **97**, 223107 (2010).

39. Xue, F. *et al.* Influence of external electric field on piezotronic effect in ZnO nanowires. *Nano Research* **8**, 2390-2399 (2015).
40. Yu, Q. *et al.* Highly sensitive strain sensors based on piezotronic tunneling junction. *Nature Communications* **13**, 778 (2022).
41. Zhang, W., Zhu, R., Nguyen, V. & Yang, R. Highly sensitive and flexible strain sensors based on vertical zinc oxide nanowire arrays. *Sensors and Actuators A: Physical* **205**, 164-169 (2014).
42. Liu, S. *et al.* Statistical piezotronic effect in nanocrystal bulk by anisotropic geometry control. *Advanced Functional Materials* **31**, 2010339 (2021).
43. Li, X., Wei, X., Xu, T., Pan, D., Zhao, J. & Chen, Q. Remarkable and crystal-structure-dependent piezoelectric and piezoresistive effects of InAs nanowires. *Advanced Materials* **27**, 2852-2858 (2015).
44. Lee, T., Lee, W., Kim, S.-W., Kim, J. J. & Kim, B.-S. Flexible textile strain wireless sensor functionalized with hybrid carbon nanomaterials supported ZnO nanowires with controlled aspect ratio. *Advanced Functional Materials* **26**, 6206-6214 (2016).
45. Park, M., Park, Y. J., Chen, X., Park, Y. K., Kim, M. S. & Ahn, J. H. MoS_2-based tactile sensor for electronic skin applications. *Advanced Materials* **28**, 2556-2562 (2016).
46. Yan, W. *et al.* Giant gauge factor of Van der Waals material based strain sensors. *Nature Communications* **12**, 2018 (2021).
47. Ma, Y. *et al.* A highly flexible and sensitive piezoresistive sensor based on MXene with greatly changed interlayer distances. *Nature Communications* **8**, 1207 (2017).
48. Zheng, K. *et al.* Observation of enhanced carrier transport properties of Si ⟨100⟩-oriented whiskers under uniaxial strains. *Applied Physics Letters* **104**, 013111 (2014).
49. Greil, J., Lugstein, A., Zeiner, C., Strasser, G. & Bertagnolli, E. Tuning the electro-optical properties of germanium nanowires by tensile strain. *Nano Letters* **12**, 6230-6234 (2012).
50. Gong, S. *et al.* A wearable and highly sensitive pressure sensor with ultrathin gold nanowires. *Nature Communications* **5**, 3132 (2014).

51. Li, Y., Luo, S., Yang, M.-C., Liang, R. & Zeng, C. Poisson ratio and piezoresistive sensing: a new route to high-performance 3D flexible and stretchable sensors of multimodal sensing capability. *Advanced Functional Materials* **26**, 2900-2908 (2016).
52. Atalay, O. Textile-based, interdigital, capacitive, soft-strain sensor for wearable applications. *Materials* **11**, 768 (2018).
53. Zhang, Q., Wang, Y. L., Xia, Y., Zhang, P. F., Kirk, T. V. & Chen, X. D. Textile-only capacitive sensors for facile fabric integration without compromise of wearability. *Advanced Materials Technologies* **4**, 1900485 (2019).
54. Lee, J. *et al.* Stretchable and suturable fibre sensors for wireless monitoring of connective tissue strain. *Nature Electronics* **4**, 291-301 (2021).
55. Wang, L. *et al.* Flexoelectronics of centrosymmetric semiconductors. *Nature Nanotechnology* **15**, 661-667 (2020).
56. Guo, D. *et al.* Silicon flexoelectronic transistors. *Science Advances* **9**, eadd3310 (2023).
57. Feng, R. & Farris, R. J. Linear thermoelastic characterization of anisotropic poly(ethylene terephthalate) films. *Journal of Applied Polymer Science* **86**, 2937-2947 (2002).
58. Wen, X., Li, D., Tan, K., Deng, Q. & Shen, S. Flexoelectret: an electret with a tunable flexoelectriclike response. *Physical Review Letters* **122**, 148001 (2019).
59. Su, Y.-L., Gupta, K., Hsiao, Y.-L., Wang, R.-C. & Liu, C.-P. Gigantic enhancement of electricity generation in piezoelectric semiconductors by creating pores as a universal approach. *Energy & Environmental Science* **12**, 410-417 (2019).
60. Wu, W. & Wang, Z. L. Piezotronics and piezo-phototronics for adaptive electronics and optoelectronics. *Nature Reviews Materials* **1**, 16031 (2016).
61. Pan, C., Zhai, J. & Wang, Z. L. Piezotronics and piezo-phototronics of third generation semiconductor nanowires. *Chemical Reviews* **119**, 9303-9359 (2019).

REVIEWER COMMENTS

Reviewer #1 (Remarks to the Author):

In this manuscript, the authors have developed a dual-modal piezotronic transistor (DPT) using a combination of piezoelectric nano/microwire and a flexible polymer with protrusions. This innovative design allows the DPT to operate in two modes: highly sensitive vertical force sensing and lateral strain sensing. In vertical force sensing mode, the DPT shows impressive force sensitivity and can detect even small forces with high precision. In lateral strain sensing mode, the DPT can respond to significant compression strain with a high on/off ratio and gauge factor. This design offers a unique solution by combining both vertical force sensing and lateral strain sensing in a single nanodevice, presenting a practical approach for creating versatile multimodal devices. So, I would recommend this manuscript for possible publication in Macromolecules after minor revision.

Comments:

1. To provide readers with more diverse examples of research, I recommend "doi.org/10.1002/eom2.12384", “doi.org/10.1002/smll.202105811” and “doi.org/10.1016/j.nanoen.2020.105610” as examples for ‘force/strain sensors’ in line 45.

2. In line 91, it becomes evident that a comparison with various references has been conducted to assess the performance of the sensor in relation to prior research. However, the list of references comprises articles that are not recent, thus making an objective assessment of whether comparable levels of performance align with current research trends challenging. Therefore, in pursuit of a more quantifiable comparison, it is suggested that the authors consider selecting references from sensors crafted using the same material and published within the last five years. This approach would enable a more precise and accurate evaluation of performance in relation to your research.

3. Figure 2 focuses on the outcomes of finite element method (FEM) modeling conducted for the structural optimization of the dual-modal piezotronic transistor (DPT). Various parameters were employed as input values based on the architecture of the DPT. Notably, the value of "d," representing the downward displacement of the upper surface of the microprotrusion, was systematically varied in an incremental manner. It is observed that the largest value of "d" was set at 0.8 mm. However, practically, it is conceivable that values greater than 0.8 mm could also be applied. Therefore, the reason behind constraining the maximum value to 0.8 mm is curious.

4. In the context of the experimental setup, in lines 190 to 192, it is discernible that a dynamometer was positioned beneath the dual-modal piezotronic transistor (DPT) to measure force. Notably, the choice to

measure force beneath the DPT raises concerns regarding the potential influence of damping effects from components such as the polydimethylsiloxane (PDMS) constituting the DPT, which could compromise the accuracy of force measurements. It is curious as to whether the utilization of devices such as load cells, directly positioned at the protrusion site where direct application of pressure occurs, was considered to enable more precise force measurements.

Reviewer #2 (Remarks to the Author):

The authors have carefully addressed the comments and answered the questions. The quality of manuscript has been improved significantly. I would recommend publication of the manuscript in its current form.

Reviewer #3 (Remarks to the Author):

I appreciate the author's meticulous response to the reviewer's comments. The discussion of the novel design, comparison of related work, setup of the experimental platform, and the more comprehensive simulation design, along with the revisions regarding PET deformation modeling, have significantly enhanced the quality and readability of the manuscript. In my opinion, the manuscript now meets the standard for publication in Nature Communications.

Point-to-Point Response to Reviewers' comments:

We thank the reviewers for their in-depth reviews and important/constructive questions/suggestions regarding our manuscript.

Reviewer #1 (Remarks to the Author):

In this manuscript, the authors have developed a dual-modal piezotronic transistor (DPT) using a combination of piezoelectric nano/microwire and a flexible polymer with protrusions. This innovative design allows the DPT to operate in two modes: highly sensitive vertical force sensing and lateral strain sensing. In vertical force sensing mode, the DPT shows impressive force sensitivity and can detect even small forces with high precision. In lateral strain sensing mode, the DPT can respond to significant compression strain with a high on/off ratio and gauge factor. This design offers a unique solution by combining both vertical force sensing and lateral strain sensing in a single nanodevice, presenting a practical approach for creating versatile multimodal devices. So, I would recommend this manuscript for possible publication in Macromolecules after minor revision.

Response:

We highly appreciate the reviewer for acknowledging the importance of our work and commenting that "This design offers a unique solution by combining both vertical force sensing and lateral strain sensing in a single nanodevice, presenting a practical approach for creating versatile multimodal devices." We also thank the reviewer for the detailed and valuable suggestions about our work, and thank the reviewer for the time and effect in helping us to significantly improve the quality of our manuscript.

Question #1

To provide readers with more diverse examples of research, I recommend "doi.org/10.1002/eom2.12384", "doi.org/10.1002/smll.202105811" and "doi.org/10.1016/j.nanoen.2020.105610" as examples for 'force/strain sensors' in line 45.

Response:

We thank the reviewer very much for this nice suggestion, which is beneficial to improve the manuscript. We have carefully studied the papers provided by the reviewer and some other useful literatures, which are cited as examples for 'force/strain sensors' in line 45.

The main text revised in the manuscript is listed as following for your convenience (all changes made in the revised manuscript are marked in red):

"According to different working principles, force/strain sensors can be mainly classified into piezoresistive[6], capacitive[5, 8], piezoelectric[3, 9-11], and piezotronic[4] types, *etc*.

……

3. Yang, F. *et al.* Wafer-scale heterostructured piezoelectric bio-organic thin films. *Science* **373**, 337-342 (2021).
4. Wu, W., Wen, X. & Wang, Z. L. Taxel-addressable matrix of vertical-nanowire piezotronic transistors for active and adaptive tactile imaging. *Science* **340**, 952-957 (2013).
5. Su, Q. *et al.* A stretchable and strain-unperturbed pressure sensor for motion interference–free tactile monitoring on skins. *Sci. Adv.* **7**, eabi4563 (2021).
6. Ma, Y. *et al.* A highly flexible and sensitive piezoresistive sensor based on MXene with greatly changed interlayer distances. *Nat. Commun.* **8**, 1207 (2017).
7. Chen, Y., Gao, Z., Zhang, F., Wen, Z. & Sun, X. Recent progress in self-powered multifunctional e-skin for advanced applications. *Exploration* **2**, 20210112 (2022).
8. Lee, J. *et al.* Stretchable and suturable fibre sensors for wireless monitoring of connective tissue strain. *Nat. Electron.* **4**, 291-301 (2021).
9. Liu, D.-S. *et al.* Piezoionic-powered graphene strain sensor based on solid polymer electrolyte. *Nano Energy* **81**, 105610 (2021).
10. Lee, S. *et al.* Ambient humidity-induced phase separation for fiber morphology engineering toward piezoelectric self-powered sensing. *Small* **18**, 2105811 (2022).
11. Kim, D. *et al.* High-performance piezoelectric yarns for artificial intelligence-enabled wearable sensing and classification. *EcoMat* **5**, e12384 (2023)."

Question #2

In line 91, it becomes evident that a comparison with various references has been conducted to assess the performance of the sensor in relation to prior research. However, the list of references comprises articles that are not recent, thus making an objective assessment of whether comparable levels of performance align with current research trends challenging. Therefore, in pursuit of a more quantifiable comparison, it is suggested that the authors consider selecting references from sensors crafted using the same material and published within the last five years. This approach would enable a more precise and accurate evaluation of performance in relation to your research.

Response:

We thank the reviewer very much for this valuable suggestion. We have carefully reviewed the relevant researches on the piezotronics using the same material in the recent five years and summarized the corresponding parameters of pressure sensitivity in **Supplementary Table 4**. This summarization enables a more precise and accurate evolution of this work and may make it have more significant impact in piezotronics. In order to improve the quality of the research, we have cited these additional literatures in the revised manuscript (marked in red) and the revised Supplementary Information (marked in red) for comparison of other researchers in related fields.

The main text revised in the manuscript is listed as following for your convenience (all changes made in the revised manuscript are marked in red):

"Its corresponding pressure sensitivity is about 1.759 eV/MPa, more than 16.5 times of previously reported piezotronic transistors[4, 21, 27-33].

……

21. Wang, L. *et al.* 2D piezotronics in atomically thin zinc oxide sheets: interfacing gating and channel width gating. *Nano Energy* **60**, 724-733 (2019).

……

27. Liu, S. *et al.* Double-channel piezotronic transistors for highly sensitive pressure sensing. *ACS Nano* **12**, 1732-1738 (2018).

28. Han, X., Du, W., Yu, R., Pan, C. & Wang, Z. L. Piezo-phototronic enhanced UV sensing based on a nanowire photodetector array. *Adv. Mater.* **27**, 7963-7969 (2015).
29. Zhao, Z. *et al.* Piezotronic effect in polarity-controlled GaN nanowires. *ACS Nano* **9**, 8578-8583 (2015).
30. Jiang, C. *et al.* 60 nm pixel-size pressure piezo-memory system as ultrahigh-resolution neuromorphic tactile sensor for in-chip computing. *Nano Energy* **87**, 106190 (2021).
31. Han, X. *et al.* A two-terminal optoelectronic synapses array based on the ZnO/Al_2O_3/CdS heterojunction with strain-modulated synaptic weight. *Adv. Electron. Mater.* **9**, 2201068 (2023).
32. An, C. *et al.* Piezotronic and piezo-phototronic effects of atomically-thin ZnO nanosheets. *Nano Energy* **82**, 105653 (2021).
33. Park, J. *et al.* Individually addressable and flexible pressure sensor matrixes with ZnO nanotube arrays on graphene. *NPG Asia Mater.* **14**, 40 (2022)."

The main content revised in the Supplementary Information is also listed as following for your convenience (all changes made in the revised Supplementary Information are marked in red):

"Supplementary Table 4 | Summary of pressure sensitivity of piezotronic sensors

Materials	Morphology	Pressure sensitivity (meV/MPa)	Reference
ZnO	MW	~1.759×10^3	This work
ZnO	Bulk	0.17	[44]
ZnO	Bulk	0.3	[45]
ZnO	Bulk	1.26	[46]
ZnO	NW	0.083~0.422	[6]
ZnO	NW	0.308~0.487	[47]
ZnO	NW	0.148~0.166	[48]
CdSe	NW	~0.677	[5]

GaN	NW	0.212~0.24	[7]
ZnO	Nanotube	0.771~2.255	[49]
ZnO	NW cluster	0.287~0.318	[50]
ZnO	NW cluster	<33.39	[4]
ZnO	Nanosheet	25.11~48.18	[17]
ZnO	Nanosheet	28.22~46.65	[51]
ZnO	Nanosheet	9.94~21.39	[52]
ZnO	Nanoplatelet	60.97~78.23 (Single Channel)	[8]
ZnO	Nanoplatelet	84.2~104.4 (Double Channel)	[53]

……

17. Wang, L. *et al.* Ultrathin piezotronic transistors with 2 nm channel lengths. *ACS Nano* **12**, 4903-4908 (2018).

……

47. Jiang, C. *et al.* 60 nm pixel-size pressure piezo-memory system as ultrahigh-resolution neuromorphic tactile sensor for in-chip computing. *Nano Energy* **87**, 106190 (2021).

48. Han, X. *et al.* A two-terminal optoelectronic synapses array based on the ZnO/Al_2O_3/CdS heterojunction with strain-modulated synaptic weight. *Advanced Electronic Materials* **9**, 2201068 (2023).

49. Park, J. *et al.* Individually addressable and flexible pressure sensor matrixes with ZnO nanotube arrays on graphene. *NPG Asia Materials* **14**, 40 (2022).

50. Han, X., Du, W., Yu, R., Pan, C. & Wang, Z. L. Piezo-phototronic enhanced UV sensing based on a nanowire photodetector array. *Advanced Materials* **27**, 7963-7969 (2015).

51. Wang, L. *et al.* 2D piezotronics in atomically thin zinc oxide sheets: interfacing gating and channel width gating. *Nano Energy* **60**, 724-733 (2019).

52. An, C. *et al.* Piezotronic and piezo-phototronic effects of atomically-thin ZnO nanosheets. *Nano Energy* **82**, 105653 (2021).”

We thank the reviewer again for this constructive suggestion that helps to improve the quality of our manuscripts.

Question #3

Figure 2 focuses on the outcomes of finite element method (FEM) modeling conducted for the structural optimization of the dual-modal piezotronic transistor (DPT). Various parameters were employed as input values based on the architecture of the DPT. Notably, the value of "d", representing the downward displacement of the upper surface of the microprotrusion, was systematically varied in an incremental manner. It is observed that the largest value of "d" was set at 0.8 mm. However, practically, it is conceivable that values greater than 0.8 mm could also be applied. Therefore, the reason behind constraining the maximum value to 0.8 mm is curious.

Response:

We thank the reviewer for this detailed and valuable comment. We constrained the maximum value of the downward displacement (d) to 0.8 mm, mainly based on DPT structure design. In the FEM simulation, the 2D model of the DPT was constructed to optimize the DPT structure in vertical force sensing mode, which could systematically simulate the loading process with application of a certain downward displacement (d) on the top edge of the microprotrusion. Note that the downward displacement (d) should be generally limited by the height of the microprotrusion ($h_{protrusion}$). Under the application of the downward displacements (d) varying from 0.0 to 1.0 mm as illustrated in **Figure R1a**, the calculated spatial distribution of displacement profiles could clearly demonstrate the shape change of the DPT with a constant height of the microprotrusion ($h_{protrusion} = 1\ mm$). It is worth noting that the surface of the microprotrusion would be gradually compressed as the increasing displacement (d), which is more inclined to induce the bulging deformation of the surrounding PDMS layer. According to the enlarged images of the central region around the microprotrusion in **Figure R1b**, when the downward displacement (d) exceeds 0.9 mm, the horizontal height of the microprotrusion surface would be lower than the level of the surrounding area in the top PDMS layer due to the bulging deformation. These

findings suggest that the actual loading area might be larger than the efficient area of the microprotrusion surface, which would spread the pressure on the top PDMS layer surrounding, leading to the increased risk of the inaccurate measurement as well as the sensibility loss. Therefore, in order to avoid the potential problems in the excessively high displacement range ($d > 0.8\,mm$) mentioned above, we constrained the maximum value of the downward displacement (d) to 0.8 mm and investigated the sensing performance of the DPT under an appropriate displacement range theoretically and experimentally.

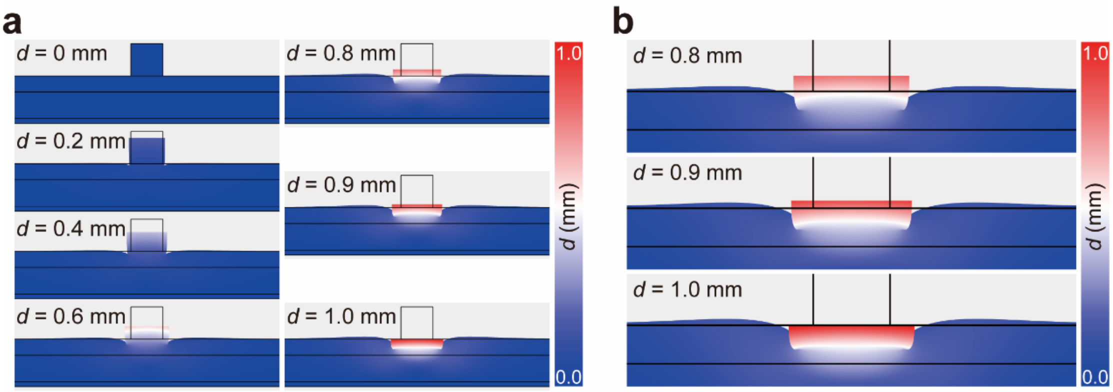

Figure R1. Simulation results of the DPT with the microprotrusion height $h_{protrusion}$ of 1.0 mm in response to different downward displacements (d). a, Displacement distribution profiles of the DPT under downward displacements (d) varying from 0.0 to 1.0 mm. **b**, Enlarged images of the central region around the microprotrusion and the surface of the PDMS layer with application of a large displacement range from 0.8 mm to 1.0 mm in **a** (right).

Question #4

In the context of the experimental setup, in lines 190 to 192, it is discernible that a dynamometer was positioned beneath the dual-modal piezotronic transistor (DPT) to measure force. Notably, the choice to measure force beneath the DPT raises concerns regarding the potential influence of damping effects from components such as the polydimethylsiloxane (PDMS) constituting the DPT, which could compromise the accuracy of force measurements. It is curious as to whether the utilization of devices

such as load cells, directly positioned at the protrusion site where direct application of pressure occurs, was considered to enable more precise force measurements.

Response:

We appreciate the reviewer very much for this constructive suggestion and kindly reminder. According to the suggestion provided by the reviewer, we carefully checked the experimental method to accurately calibrate the force applied to the DPT. In order to quantitatively evaluate the potential influence of the damping effects from PDMS on the force measurement, we redesigned a home-made experimental setup as shown in **Figure R2a**, which could measure the vertical force in different positions. With this configuration, the dynamometer with a flat measurement head was vertically mounted on the linear actuator, which could precisely control the displacement of the microprotrusion surface and directly measure the applied force on the DPT. Meanwhile, the force senor under the substrate could measure the force beneath the DPT as the original one. By measuring the force on the microprotrusion (F_1) and the force beneath the DPT (F_2) simultaneously, we can get a relatively accurate force. It should be noted that the force sensor is zeroed after the DPT is placed on it, so F_2 does not include the weight of the DPT. As demonstrated by the test results in **Figure R2b** and **Figure R2c**, the average values of force difference ($\Delta F = F_1 - F_2$) can be controlled within 2 mN and the vertical forces are basically identical with the maximum error $\Delta F/F_2$ less than 3%, which indicates that the PDMS damping effects might actually have little effect on the accuracy of the force measurement in this work. Nevertheless, it is important to utilize more precise force measurement system to avoid interference of other insignificance factors in our future studies. The above discussions have been added to the **Methods** in the revised manuscript as well as the Supplementary Information (**Supplementary Note 11**, **Supplementary Fig. 29**), and marked in red to improve the quality of the paper. Thank the reviewer again for this constructive and valuable suggestion.

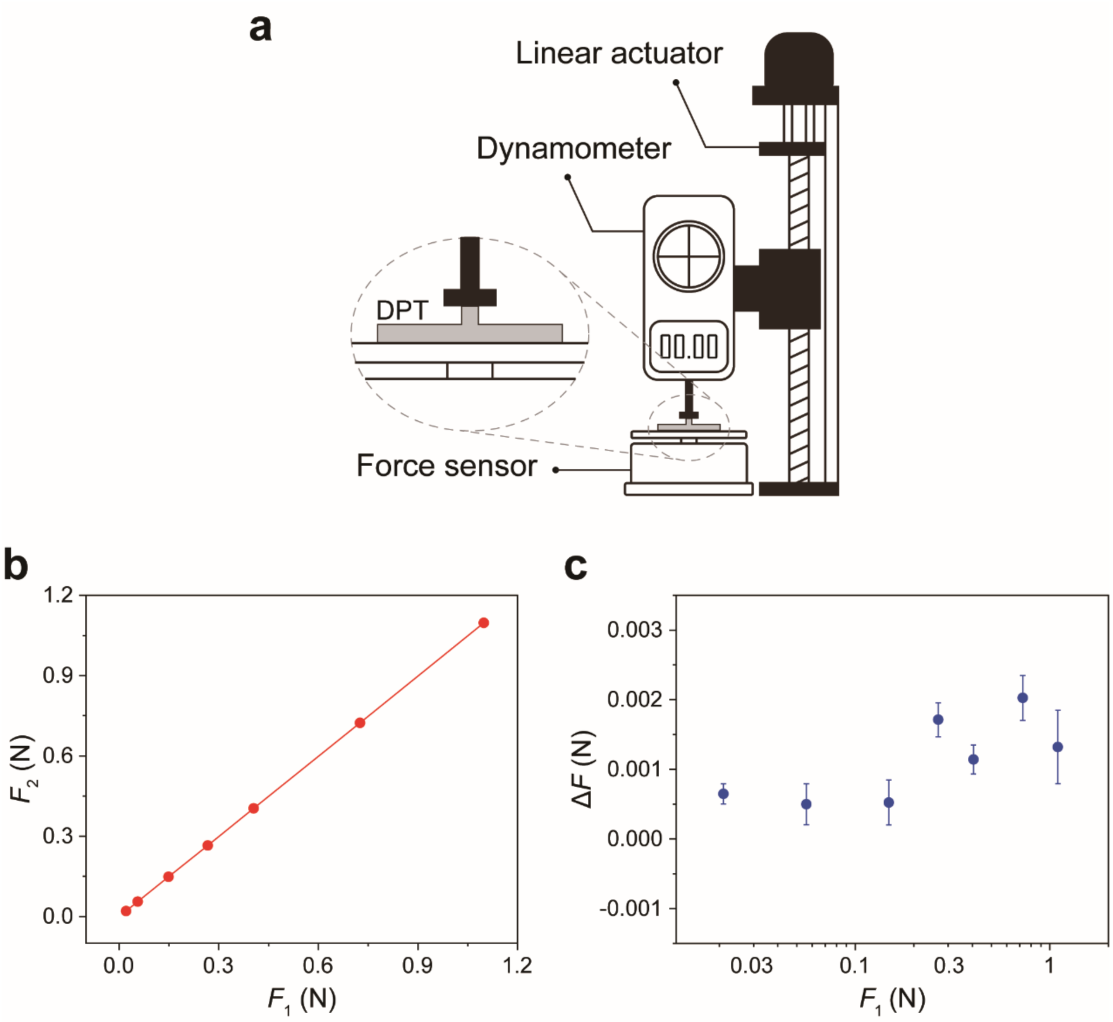

Figure R2. The relationship of the vertical force on DPTs measured in different positions. a, Schematic illustration of the force measurement system for the DPT in vertical force sensing mode, composed mainly of a force sensor under the substrate of the DPT and a dynamometer vertically mounted on the linear actuator. **b**, Curve of force beneath the DPT (F_2) versus force on the microprotrusion (F_1) measured simultaneously. **c**, Difference value of the measured force ($\Delta F = F_1 - F_2$) from statistics results in **b**.

Reviewer #2 (Remarks to the Author):

The authors have carefully addressed the comments and answered the questions. The quality of manuscript has been improved significantly. I would recommend publication of the manuscript in its current form.

Response:

We highly appreciate the reviewer's detailed, very positive and valuable comments regarding our manuscript during the review process. According to these important comments and suggestions, we have gained a better understanding of the detailed regulations in DPT, a better showing of the mechanism for the performance improvement and the novelty of the DPT design, and many more valuable details that significantly help us to improve the manuscript. We thank the reviewer very much again for the time and effect in improving the quality of our manuscript.

Reviewer #3 (Remarks to the Author):

I appreciate the author's meticulous response to the reviewer's comments. The discussion of the novel design, comparison of related work, setup of the experimental platform, and the more comprehensive simulation design, along with the revisions regarding PET deformation modeling, have significantly enhanced the quality and readability of the manuscript. In my opinion, the manuscript now meets the standard for publication in Nature Communications.

Response:

We highly appreciate the reviewer's very constructive and valuable comments during the review process. The reviewer's comments and suggestions greatly helped us. And according to your suggestions, the quality of this work has been significantly enhanced including better revealing the novel design of DPT, its comparison of related works, its experimental setup and lots of details for readability. We thank the reviewer very much again for the time and effect in improving the quality of our manuscript.

REVIEWERS' COMMENTS

Reviewer #1 (Remarks to the Author):

I appreciate your thoroughly addressing my suggestions. I am particularly grateful for the addition of visual aids such as figures and graphs, which greatly aided comprehension. Through the revision process, I believe that the reliability and quality of the paper have been enhanced. In my view, this manuscript is suitable for publication in Nature Communications.

Point-to-Point Response to Reviewer's comments:

We thank the reviewer for the in-depth reviews and important/constructive questions/suggestions regarding our manuscript.

Reviewer #1 (Remarks to the Author):

I appreciate your thoroughly addressing my suggestions. I am particularly grateful for the addition of visual aids such as figures and graphs, which greatly aided comprehension. Through the revision process, I believe that the reliability and quality of the paper have been enhanced. In my view, this manuscript is suitable for publication in Nature Communications.

Response:

We highly appreciate the reviewer's detailed, responsible and valuable reviewing of our manuscript during the review process. According to the reviewer's comments and suggestions, the quality of the paper has been improved for better readability, which also helps us gain a better understanding of our work. We thank the reviewer very much again for the time and effect in improving the quality of our manuscript.
